# Integrated transcriptomics identifies ER stress–associated apoptosis in post-resuscitation AKI and supports early Dl-3-n-butylphthalide–associated renoprotection in a porcine TCA model

**DOI:** 10.3389/fphar.2026.1841271

**Published:** 2026-06-04

**Authors:** Kechun Zhou, Wang Du, Yi Chen, Yufeng Hu, Pin Lan

**Affiliations:** 1 Department of Emergency Medicine, The Fifth Affiliated Hospital of Wenzhou Medical University, Lishui Central Hospital, Lishui Hospital of Zhejiang University, Lishui, Zhejiang, China; 2 Department of Emergency Medicine, Second Affiliated Hospital of Zhejiang University School of Medicine, Hangzhou, China; 3 Key Laboratory of The Diagnosis and Treatment of Severe Trauma and Burns of Zhejiang Province, Hangzhou, China; 4 Clinical Research Center for Emergency and Critical Care Medicine of Zhejiang Province, Hangzhou, China

**Keywords:** acute kidney injury, apoptosis, dl-3-n-butylphthalide, endoplasmic reticulum stress, traumatic cardiac arrest

## Abstract

**Background:**

Dl-3-n-butylphthalide (NBP) is a small-molecule compound derived from celery seeds with anti-inflammatory, antioxidant, and anti-apoptotic properties. Although NBP has shown protective effects in various kidney diseases, its role in traumatic cardiac arrest (TCA)-induced acute kidney injury (AKI) remains unclear. This study aimed to investigate the effects of NBP on AKI following TCA in pigs and to determine whether these effects were associated with modulation of ERS/UPR-associated apoptotic readouts.

**Methods:**

We integrated ischemia–reperfusion injury (IRI)-related bulk transcriptomic/microarray datasets with single-cell RNA sequencing (scRNA-seq) data. Differential expression analysis, Hallmark GSEA, and GSVA/ssGSEA were performed to quantify unfolded protein response (UPR) and apoptosis, focusing on proximal tubule (PT) cells via PT-state stratification, pseudo-bulk differential analysis, and pseudotime inference. For *in vivo* validation, healthy male Bama minipigs were randomized to Sham, TCA, or TCA+NBP groups. NBP (2.5 mg/kg, i.v.) was administered within 120 min after return of spontaneous circulation (ROSC). Serum creatinine (Cr) and blood urea nitrogen (BUN) were measured at 1, 2, 4, and 24 h; kidneys harvested at 24 h underwent H&E and TUNEL staining, immunohistochemistry (KIM-1, NGAL), and Western blotting (PERK, CHOP, caspase-12, caspase-3).

**Results:**

Integrated bulk, single-cell, PT-state, pseudotime, and PT pseudo-bulk analyses prioritized a PT-enriched PERK–ATF4–CHOP-associated stress-apoptosis module, predominantly within injury-associated PT states. Along PT injury-state progression, HSPA5/GRP78, PERK/EIF2AK3, ATF4, CHOP/DDIT3, and apoptosis-related effectors showed coordinated transcript-level remodeling, suggesting engagement of an ERS/UPR-associated stress-apoptosis program rather than establishing pathway causality. Compared with TCA, NBP significantly reduced Cr and BUN, alleviated histopathologic injury, decreased KIM-1/NGAL expression, reduced TUNEL-positive cells and caspase-3 abundance, and was associated with lower PERK, CHOP, and caspase-12 protein expression.

**Conclusion:**

A PT-enriched PERK-ATF4-CHOP-associated ERS/UPR stress-apoptosis module was prioritized in injury-associated proximal tubule states during IRI-related AKI. In a porcine TCA model, NBP was associated with reduced early AKI severity within a 24 h observation window, accompanied by lower PERK/CHOP/caspase-related ERS/UPR-associated and apoptosis-related readouts.

## 1 Introduction

Cardiac arrest (CA) remains a leading cause of mortality and long-term disability worldwide. Despite iterative updates to resuscitation guidelines and continuous optimization of prehospital-to-in-hospital systems of care, overall survival—particularly after out-of-hospital cardiac arrest—remains disappointingly low (Perman et al., 2024). Within the CA spectrum, traumatic cardiac arrest (TCA) represents a biologically and operationally distinct entity, most commonly arising from severe polytrauma, hemorrhagic shock, and lethal thoracoabdominal injury, and it continues to carry an exceptionally high case-fatality rate (Abrard et al., 2025). Population-based studies indicate that although TCA accounts for a minority of out-of-hospital arrests, its etiologic architecture and prognostic determinants diverge substantially from non-traumatic CA, with consistently poorer outcomes (Wolthers et al., 2023). Notably, however, the dissemination of damage-control resuscitation, rapid hemorrhage control, and protocolized trauma systems has coincided with improving survival in selected high-performing trauma centers (Schober et al., 2024). Against this evolving backdrop, a central unmet need is not only to achieve return of spontaneous circulation (ROSC), but to mitigate the downstream cascade of secondary organ injury that ultimately governs in-hospital mortality and functional recovery.

Even after successful ROSC, patients frequently transition into post–cardiac arrest syndrome driven by systemic ischemia–reperfusion injury (IRI), culminating in multiple organ dysfunction—a dominant determinant of inpatient death (Czerwinska et al., 2019). Acute kidney injury (AKI) is among the most prevalent and clinically consequential complications in this setting. Beyond serving as an epiphenomenon of global hypoperfusion, AKI can amplify systemic inflammation and metabolic derangements, destabilize hemodynamics, and further impair organ perfusion, thereby linking it mechanistically to increased mortality, prolonged intensive care unit length of stay, and adverse long-term renal outcomes (Geri et al., 2015; Tujjar et al., 2015). Yet current management of post-resuscitation AKI remains largely supportive—hemodynamic optimization, fluid stewardship, and renal replacement therapy—underscoring the absence of a clear, targetable renoprotective intervention with translational traction.

At the mechanistic level, an early burst of reactive oxygen species (ROS) following reperfusion constitutes a key initiating event in renal injury, precipitating mitochondrial dysfunction, propagating inflammatory signaling, and triggering tubular epithelial cell injury and death (Su et al., 2023). In this manuscript, endoplasmic reticulum stress (ERS, also referred to as ER stress) denotes disruption of ER proteostasis induced by ischemia, hypoxia, calcium disequilibrium, and oxidative stress. The unfolded protein response (UPR) refers to the adaptive signaling network activated by ERS, mainly involving the PERK, IRE1, and ATF6 branches (Inagi, 2009; Bravo et al., 2013). Among these branches, PERK–ATF4–CHOP signaling can shift from adaptive proteostasis regulation toward pro-apoptotic signaling under severe or sustained stress. In kidney IRI models, canonical ERS/UPR markers such as BiP/GRP78 and CHOP are induced, accompanied by activation of ER-associated caspase-12 and downstream caspase-3 cleavage (Zhang et al., 2020). We therefore use “ERS/UPR-associated apoptosis” to describe apoptosis temporally and biologically linked to ERS/UPR activation, without implying definitive pathway causality. These observations position ERS/UPR-associated apoptotic signaling as a plausible therapeutic leverage point in renal IRI and motivate focused evaluation of druggable nodes along this pathway.

A major barrier to mechanistic resolution is the cellular complexity of the kidney and the pronounced spatial and state-dependent heterogeneity of injury responses. Conventional bulk transcriptomics often collapses this heterogeneity and obscures cell-of-origin signals. Single-cell and single-nucleus RNA sequencing have therefore emerged as high-resolution platforms to reconstruct renal cell atlases and delineate disease-associated cellular states. For example, Park and colleagues generated a reference mouse kidney single-cell transcriptomic map that has become foundational for assigning cellular provenance in kidney disease (Park et al., 2018). In AKI/IRI, single-nucleus profiling further demonstrates that proximal tubule (PT) cells occupy multiple injury, repair, and maladaptive repair states that evolve dynamically over time (Gerhardt et al., 2021). Integrating bulk and single-cell datasets offers a pragmatic synthesis: statistical robustness at the tissue level paired with cellular localization and state resolution, thereby enabling more credible identification of key pathways and intervention points (Stuart et al., 2019). Accordingly, a “single-cell + bulk” integrative framework to define the central pathways and cellular sources underlying TCA-associated IRI-AKI—and to align these pathways with experimentally testable molecular axes—provides a rational route to strengthen mechanistic inference and translational relevance.

Dl-3-n-butylphthalide (NBP), a lipophilic small molecule originally derived from celery seed, has demonstrated antioxidant, anti-inflammatory, and anti-apoptotic activities across multiple IRI-related organ injury contexts (Wang et al., 2018). In renal disease models, NBP has been reported to attenuate kidney IRI and structural injury, in part through dampening inflammatory signaling and modulating cell-death programs (Zhang et al., 2023). However, whether NBP can protect against AKI after TCA resuscitation in a clinically proximate large-animal model—and whether such protection is associated with modulation of ERS/UPR-associated apoptotic signaling remains unresolved. Here, we integrate bulk and single-cell transcriptomic analyses to nominate and localize injury-associated pathways in IRI-AKI and then evaluate NBP-associated early renoprotection in a porcine TCA resuscitation model. Rather than establishing definitive pathway causality, this study provides cross-scale evidence that NBP treatment is associated with attenuation of ERS/UPR-associated apoptotic readouts and reduced early renal injury after TCA.

## 2 Methods

### 2.1 Study design

We implemented a two-stage strategy combining computational discovery with *in vivo* validation. First, we integrated public bulk microarray and single-cell RNA-seq datasets to identify injury-associated pathways at the tissue level and to resolve their dominant cellular sources and dynamic trajectories at single-cell resolution. Second, we evaluated the transcriptomically prioritized PERK–ATF4–CHOP-associated ERS/UPR–apoptosis-related readouts in a porcine trauma-related cardiac arrest resuscitation model and assessed whether NBP treatment was associated with attenuation of early post-resuscitation AKI, aligning molecular readouts with phenotypic endpoints. Because public single-cell datasets directly derived from post-resuscitation TCA-AKI are currently limited, murine renal IRI datasets were used as a discovery platform to identify conserved tubular injury-associated stress programs. The porcine TCA model was then used as a large-animal systemic ischemia–reperfusion context to determine whether the prioritized ERS/UPR–apoptosis-related readouts and NBP-associated renal effects were observable under post-resuscitation conditions.

### 2.2 Bioinformatic analyses

#### 2.2.1 Data sources

Bulk microarray data (GSE52004; mouse kidney, IRI 24 h vs. Sham; Affymetrix Mouse Gene 1.0 ST Array, GPL6246) and scRNA-seq data (GSE139107; Sham, IRI 4 h, IRI 12 h) were obtained from GEO. All computational analyses were performed in R 4.5.1; package versions and sessionInfo are provided in Supplementary Methods and Supplementary Table.

#### 2.2.2 Bulk preprocessing and differential expression

Raw microarray intensities were processed using oligorma for background correction, quantile normalization, and log2 transformation, followed by probe-to-gene symbol mapping and gene-level summarization. Differential expression was modeled using limma (lmFit + eBayes), with Benjamini–Hochberg false discovery rate (FDR) control. Differentially expressed genes were defined by FDR <0.05 and |log2FC| > 1 (Supplementary Table).

#### 2.2.3 Hallmark pathway enrichment and sample-level scoring

Gene set enrichment analysis (GSEA) was performed using MSigDB Hallmark gene sets (Liberzon et al., 2015) on ranked gene lists, with normalized enrichment score (NES) and FDR used to quantify enrichment (Subramanian et al., 2005). To quantify pathway activity at the sample level, GSVA-derived ssGSEA/GSVA scores were computed to generate UPR_total and Apoptosis scores for between-group comparisons and visualization (Hänzelmann et al., 2013). In addition, literature-curated signatures were used to compute branch-specific UPR scores corresponding to the PERK-ATF4-DDIT3/CHOP, IRE1–XBP1, and ATF6 branches. Candidate pathways for downstream validation were pre-specified as those that were positively enriched on the IRI side (FDR <0.05), ranked highly in both bulk Hallmark GSEA and PT-focused single-cell pathway analyses. UPR_total and Apoptosis were based on Hallmark gene sets; branch signatures were version-controlled with traceable gene lists (Supplementary Methods; Supplementary Tables).

#### 2.2.4 Single-cell processing, integration, and annotation

Single-cell data were processed using Seurat with standard quality control, doublet removal, normalization, dimensionality reduction, integration, and clustering. Cells were retained according to predefined quality-control thresholds: min_features = 200, max_features = 6,000, mitochondrial gene percentage <20%, nCount_RNA ≦ the 99th percentile, and no additional minimum nCount threshold. In this analysis, the 99th percentile of nCount_RNA was 3,471. Mitochondrial genes were identified using the “^mt-” pattern. Doublets were identified and removed using scDblFinder. After quality control and doublet removal, 60,969 cells were retained for downstream analysis, including 26,493 Sham cells, 14,341 IRI_4h cells, and 20,135 IRI_12h cells. In the primary integration workflow, data were normalized using SCTransform with percent.mt regressed out, followed by principal component analysis with 30 PCs. Batch effects were corrected using Harmony with sample_id as the grouping variable. The integrated object was then processed using FindNeighbors with dimensions 1:30, FindClusters at resolution 0.6, and RunUMAP with dimensions 1:30. Cell types were annotated using canonical marker genes and verified by DotPlot and FeaturePlot visualization.

#### 2.2.5 Proximal tubule (PT) deep profiling

PT cells were extracted from the integrated single-cell object and subjected to re-clustering. PT re-analysis was performed using Harmony integration with sample_id as the grouping variable, dimensions 1:30, graph-based clustering at resolution 0.6, and UMAP visualization. After PT subsetting, 22,706 PT cells were retained for deep profiling. Three PT states were operationally defined according to marker-gene logic: PT_normal_like cells were characterized by homeostatic transport markers Slc34a1, Lrp2, Slc5a2, and Aqp1; PT_injured cells were defined by injury markers Havcr1 and Lcn2; and PT_repair cells were defined by repair, dedifferentiation, and proliferation-associated markers including Sox9, Vcam1, Krt8, Krt18, Mki67, and Top2a. The final PT-state composition was 19,087 PT_normal_like cells, 2,530 PT_injured cells, and 1,089 PT_repair cells. By condition, the PT subset contained 10,522 cells in Sham, 6,214 cells in IRI_4h, and 5,970 cells in IRI_12h. Within the PT subset, per-cell UPR_total, Hallmark Apoptosis, and branch-specific UPR scores were computed using both Seurat module scoring and UCell for cross-validation (Andreatta and Carmona, 2021), and written to cell metadata (Supplementary Methods).

Trajectory inference was performed using Monocle3 to learn PT state transitions and compute pseudotime (Cao et al., 2019). We compared pseudotime distributions across conditions and visualized dynamic trends of UPR_total, branch-specific UPR, and Apoptosis scores along pseudotime, enabling linkage of PT state evolution to functional programs.

To strengthen reproducibility and align single-cell findings with bulk-style inference, PT cells were aggregated by sample to construct a PT pseudo-bulk expression matrix. Differential expression was conducted using edgeR under the quasi-likelihood framework to improve robustness to dispersion and limited sample size (Lun et al., 2016). GSEA was then performed on pseudo-bulk results using Hallmark gene sets (Liberzon et al., 2015; Subramanian et al., 2005) to confirm concordant pathway directionality, and key axis genes were summarized and visualized across core contrasts.

#### 2.2.6 Statistical testing and candidate selection rules

Pathway scores and state proportions were analyzed using the sample as the statistical unit. Multi-group comparisons used Kruskal–Wallis tests; pairwise comparisons used Wilcoxon rank-sum tests with BH adjustment. Effect sizes (e.g., Cliff’s delta) were reported to strengthen interpretability. Candidate pathway/axis selection followed predefined criteria: concordant IRI-side enrichment across bulk Hallmark GSEA and PT-centered single-cell pathway analyses, PT state- and pseudotime-supported localization, and experimental tractability of the PERK–ATF4–CHOP/caspase-related readouts.

### 2.3 Porcine TCA resuscitation model and NBP intervention

#### 2.3.1 Animals and ethics

Nineteen male Bama miniature pigs (33–41 kg, 4–6 months) were enrolled. Two animals initially assigned to the TCA group successfully underwent model induction and achieved ROSC, but did not survive to the predefined 24 h post-ROSC endpoint. Therefore, they were excluded from the final endpoint analysis, leaving 17 animals for analysis. All procedures were approved by the Institutional Animal Care and Use Committee of the Second Affiliated Hospital, Zhejiang University School of Medicine (approval no. 2023-040) and were performed in accordance with institutional guidelines for the care and use of laboratory animals. All efforts were made to minimize animal suffering.

#### 2.3.2 Grouping and dosing

Animals were randomized using a random-number table into Sham (n = 5), TCA (n = 7), and TCA + NBP (n = 7) groups. Two animals initially assigned to the TCA group successfully underwent model induction and achieved ROSC, but failed to survive to the predefined 24 h post-ROSC endpoint. These animals were excluded from the final endpoint analysis, yielding final analyzable group sizes of Sham n = 5, TCA n = 5, and TCA + NBP n = 7. In the intervention arm, NBP was administered after ROSC at 2.5 mg/kg via intravenous infusion over 120 min. This dosing strategy was selected based on a previous porcine hemorrhage-induced cardiac arrest study in which intravenous NBP was administered at 2.5 mg/kg during the early post-resuscitation period (Zhou et al., 2025). Because only one dose and one administration window were tested in the present study, the optimal dose–response relationship and therapeutic window remain to be defined. No formal *a priori* sample-size calculation was performed. The sample size was determined based on ethical considerations, the 3Rs principle, experimental feasibility, prior experience with the porcine TCA resuscitation model, and the resource-intensive nature of large-animal resuscitation studies. Therefore, the animal experiment should be interpreted as an exploratory preclinical study designed to identify biologically coherent treatment-associated signals rather than as a definitively powered efficacy trial.

#### 2.3.3 Model induction, resuscitation protocol, and observation window

Animals were fasted for 12 h with free access to water. Anesthesia was induced by intramuscular injection of tiletamine/zolazepam (5.0 mg/kg) combined with xylazine (1.0 mg/kg) to achieve initial sedation. After adequate sedation, a marginal ear vein catheter was established. Propofol (10 mg/mL) was then administered via the marginal ear vein at a loading dose of 5.0 mg/kg, followed by continuous infusion at 2.0–5.0 mg/kg/h to maintain a surgical plane of anesthesia during instrumentation and model induction. Adequate anesthetic depth was assessed by the loss of corneal reflex, together with continuous physiologic monitoring. Animals were intubated and mechanically ventilated (SV350, Mindray) with tidal volume 10 mL/kg, respiratory rate 12/min, peak flow 40 L/min, FiO_2_ 21%. Vascular access was established for continuous monitoring of aortic and right atrial pressures (Mindray BeneVision N15). A separate arterial sheath facilitated controlled hemorrhage and transfusion. Catheters were flushed with heparinized saline (5 U/mL). ECG and peripheral oxygen saturation were continuously monitored throughout the procedure.

After 15 min stabilization, baseline measurements were obtained. Cardiac arrest was induced by controlled hemorrhage *via* the femoral artery at 2 mL/kg/min until mean arterial pressure (MAP) ≤ 10 mmHg with pulseless electrical activity on ECG. Shed blood was collected aseptically. Ventilation was stopped for 7 min; if MAP increased, additional blood was withdrawn to maintain MAP ≤10 mmHg. Resuscitation began with reinfusion of 50% of the shed blood at 5 mL/kg/min and restoration of ventilation at FiO_2_ 100%. ROSC was defined as restoration of an organized rhythm with MAP ≥50 mmHg sustained for 5 min. If ROSC was not achieved, epinephrine (10 μg/kg) was administered every minute up to five doses; ventricular fibrillation was treated with 150 J biphasic defibrillation. After ROSC, FiO_2_ was reduced to 21% with other ventilator settings unchanged; animals were monitored for 4 h. Hypocalcemia (Ca^2+^ < 1.0 mmol/L) was corrected with 10 mL of 10% calcium gluconate. Animals were then recovered and observed until 24 h post-ROSC. At 24 h after ROSC, animals were deeply anesthetized with propofol (3 mg/kg, intravenous via the marginal ear vein) until loss of corneal reflex, and were subsequently euthanized by rapid intravenous administration of 10% potassium chloride (10 mL per animal) via the marginal ear vein. Death was confirmed by the absence of spontaneous respiration, pulse, and cardiac electrical activity. Kidneys were rapidly harvested, and left upper-pole tissue was collected for downstream assays.

### 2.4 Endpoints and assays

#### 2.4.1 Renal function and injury biomarkers

Serum creatinine (Cr) and blood urea nitrogen (BUN) were measured at baseline and 1 h, 2 h, 4 h, and 24 h after ROSC.

#### 2.4.2 Histology and immunohistochemistry

Formalin-fixed paraffin-embedded kidney sections were stained with hematoxylin and eosin (H&E) to assess injury, and TUNEL staining was used to quantify apoptosis. Immunohistochemistry was performed for KIM-1 and NGAL; integrated optical density (IOD) was quantified from multiple randomly selected, non-overlapping fields and averaged per section. Histological assessment, TUNEL counting, and immunohistochemical quantification were performed by investigators blinded to group allocation.

#### 2.4.3 Western blotting

Renal tissues were lysed for protein extraction and quantified using a BCA assay, followed by SDS–PAGE and transfer to membranes. PERK, CHOP, caspase-12, and caspase-3 were probed, with GAPDH used as the loading control. Signals were visualized by ECL and quantified in ImageJ with normalization to GAPDH. Western blot densitometry was performed by an investigator blinded to group allocation. PERK and CHOP correspond to the transcript-level genes Eif2ak3 and Ddit3, respectively, whereas caspase-12 and caspase-3 were used as downstream apoptosis-related protein readouts.

#### 2.4.4 Statistical analysis (animal experiments)

Analyses were conducted in SPSS 26.0. Continuous variables were assessed for normality using the Shapiro–Wilk test. Normally distributed data are presented as mean ± SD. For single-timepoint outcomes, between-group comparisons were performed using one-way ANOVA followed by Bonferroni-adjusted *post hoc* tests. For renal function indices measured repeatedly over time, repeated-measures ANOVA was used, with group, time, and group-by-time interaction included in the model. Sphericity was assessed using Mauchly’s test; when the sphericity assumption was violated, Greenhouse–Geisser correction was applied. Missing values were not imputed, and available observations were analyzed according to the final included animals. Where appropriate, effect sizes and 95% confidence intervals were reported to improve interpretability. A two-sided P < 0.05 was considered statistically significant. Raw measurements are provided in Supplementary_Raw data.

## 3 Results

### 3.1 Bulk microarray transcriptomics

**FIGURE 1:**
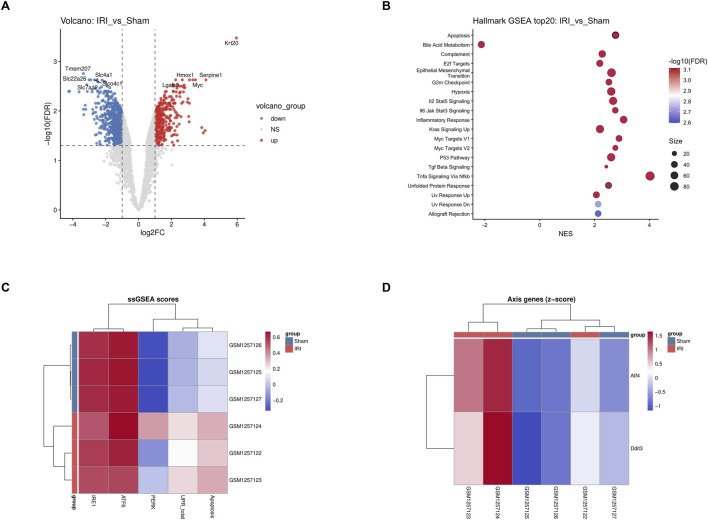
Differential expression, ERS/UPR-associated pathways, and PERK–ATF4–CHOP axis-gene signals from bulk microarray data. **(A)** Volcano plot of IRI *versus* Sham shows widespread transcriptional remodeling, with multiple stress- and injury-related genes among the prominently altered signals. **(B)** Hallmark GSEA dot plot indicates enrichment of the Hallmark Unfolded Protein Response and Hallmark Apoptosis pathways, accompanied by activation of injury-response pathways such as Hypoxia, TNFα/NF-κB, inflammatory response, and p53 signaling. **(C)** ssGSEA heatmap at the sample level shows condition-related increases in UPR_total and Apoptosis activity in the IRI group, with branch-specific variation across UPR-related scores. **(D)** Heatmap of PERK–ATF4–CHOP axis genes shows increased z-score expression of Atf4 and Ddit3/CHOP in IRI samples relative to Sham samples.

To identify IRI-associated mechanisms in an unbiased manner at the tissue level, we first performed differential expression analysis comparing IRI *versus* Sham in the microarray dataset. The volcano plot revealed widespread transcriptional remodeling after renal IRI, with multiple ERS/UPR- and apoptosis-related genes represented among the prominently altered signals (Figure 1A). Hallmark GSEA demonstrated significant positive enrichment of Unfolded Protein Response and Apoptosis in IRI, alongside activation of Hypoxia, TNFα/NF-κB, inflammatory response, p53 pathway, and other stress-response programs (Figure 1B). To translate enrichment into sample-resolved metrics, we computed GSVA/ssGSEA scores. Heatmap visualization showed globally increased UPR_total and Apoptosis activity in IRI samples, with branch-specific UPR scores displaying condition-related variation (Figure 1C). At the node level, the PERK-ATF4-CHOP transcriptional output was coherently induced, as reflected by increased z-score expression of Atf4 and Ddit3/CHOP in IRI samples relative to Sham samples (Figure 1D). Collectively, these data define a tissue-level signature of heightened UPR- and apoptosis-related transcriptional activity after renal IRI, with prominent involvement of the PERK-ATF4-CHOP stress-response axis.

### 3.2 Cross-platform concordance and single-cell annotation

**FIGURE 2:**
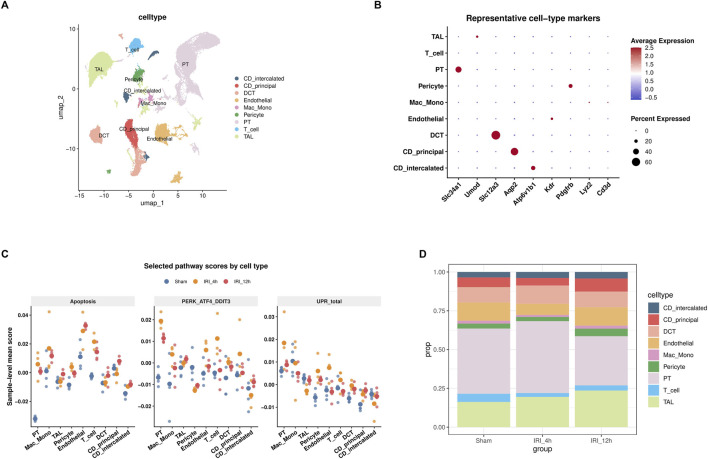
Global cell-type annotation and cell-type–resolved pathway scoring **(A)** UMAP visualization shows separation of major renal cell populations, including proximal tubule cells, thick ascending limb cells, distal convoluted tubule cells, collecting-duct principal/intercalated cells, endothelial cells, pericytes, macrophage/monocyte populations, and T cells. **(B)** Representative marker-gene dot plot shows cell-type–specific expression patterns, including Slc34a1 for PT, Umod for TAL, Slc12a3 for DCT, Aqp2 for collecting-duct principal cells, Atp6v1b1 for collecting-duct intercalated cells, Kdr for endothelial cells, Pdgfrb for pericytes, Lyz2 for macrophage/monocyte populations, and Cd8d for T cells. **(C)** Cell-type–wise sample-mean pathway scores show condition-dependent changes in Apoptosis, PERK_ATF4_DDIT3, and UPR_total scores, with prominent IRI-associated stress and apoptosis-related signals in the PT compartment. **(D)** Cell-type composition analysis shows the relative proportions of major renal cell populations across Sham, IRI_4h, and IRI_12 h samples.

To determine the cellular context of the injury-associated ERS/UPR and apoptosis-related transcriptional programs, we next analyzed the single-cell RNA-seq dataset. To improve reproducibility and allow readers to evaluate the robustness of the single-cell workflow, predefined quality-control criteria were applied, including min_features = 200, max_features = 6,000, mitochondrial gene percentage <20%, nCount_RNA ≤ the 99th percentile, and doublet removal using scDblFinder. After quality control and doublet removal, 60,969 cells were retained for downstream analysis, including 26,493 cells from Sham, 14,341 cells from IRI_4h, and 20,135 cells from IRI_12h. Harmony integration and UMAP visualization resolved major renal cell populations, including PT, TAL, DCT, collecting-duct principal/intercalated cells, endothelium, pericytes, macrophage/monocyte populations, and T cells (Figure 2A).

DotPlot marker validation supported annotation fidelity: PT cells expressed Slc34a1, TAL cells expressed Umod, DCT cells expressed Slc12a3, collecting-duct principal cells expressed Aqp2, collecting-duct intercalated cells expressed Atp6v1b1, endothelial cells expressed Kdr, pericytes expressed Pdgfrb, macrophage/monocyte populations expressed Lyz2, and T cells expressed Cd8d (Figure 2B). After annotation, we aggregated selected pathway scores by cell type and condition. Under IRI, PT cells showed prominent increases in Apoptosis, UPR_total, and PERK_ATF4_DDIT3 branch scores, indicating that the PERK–ATF4–CHOP-related stress axis and apoptosis-related programs were preferentially evident in the PT compartment (Figure 2C). Cell-type proportion analysis further showed that major renal cell populations were represented across Sham, IRI_4h, and IRI_12 h samples, while IRI was accompanied by compositional shifts across compartments over time (Figure 2D). Given the PT enrichment of PERK–ATF4–CHOP-related UPR activity and apoptosis-related signals, downstream single-cell analyses centered on PT re-clustering to resolve intra-PT heterogeneity and identify the PT states carrying this axis-related response.

### 3.3 Marker-driven PT state definition

Within the PT subset, we defined cell states using marker programs reflecting homeostatic transport, injury, and repair/dedifferentiation with proliferation. After PT subsetting and re-clustering, 22,706 PT cells were retained for deep profiling. The final PT-state composition included 19,087 PT_normal_like cells, 2,530 PT_injured cells, and 1,089 PT_repair cells. Stratified by condition, Sham contained 10,153 PT_normal_like cells, 75 PT_injured cells, and 294 PT_repair cells; IRI_4h contained 5,264 PT_normal_like cells, 838 PT_injured cells, and 112 PT_repair cells; and IRI_12h contained 3,670 PT_normal_like cells, 1,617 PT_injured cells, and 683 PT_repair cells.

**FIGURE 3:**
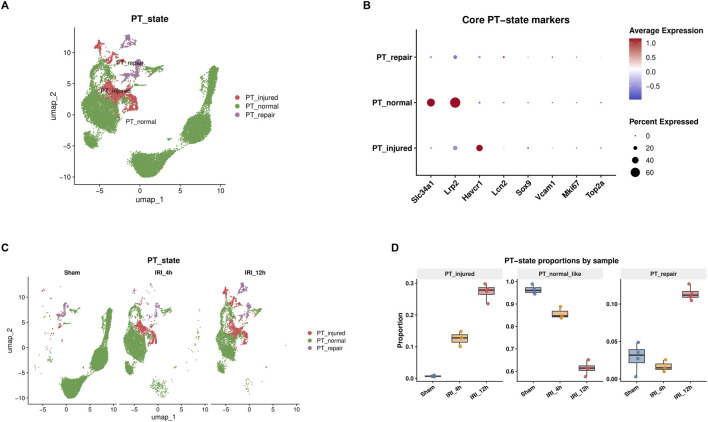
PT state definition and distribution after IRI **(A)** UMAP visualization of the PT subset shows three operational PT states: PT_normal_like, PT_injured, and PT_repair. **(B)** PT-state marker dot plot shows Slc34a1 and Lrp2 enriched in PT_normal_like cells, Havcr1 and Lcn2 enriched in PT_injured cells, and Sox9, Vcam1, Mki67, and Top2a associated with PT_repair cells. **(C)** Group-wise UMAP plots show progressive expansion of PT_injured and PT_repair regions after IRI, particularly at IRI_12 h. **(D)** Sample-level PT-state composition analysis shows a decrease in PT_normal_like cells and increases in PT_injured and PT_repair states after IRI.

UMAP visualization showed that PT_normal_like, PT_injured, and PT_repair occupied distinct but partially connected regions within the PT manifold (Figure 3A). DotPlot quantification supported these assignments: Slc34a1 and Lrp2 were enriched in PT_normal_like cells; Havcr1 and Lcn2 were enriched in PT_injured cells; and Sox9, Vcam1, Mki67, and Top2a were associated with PT_repair cells (Figure 3B). Condition-stratified UMAPs revealed expansion of PT_injured and PT_repair states after IRI, with stronger expansion at 12 h than at 4 h (Figure 3C). Compositional analyses corroborated depletion of PT_normal_like cells and enrichment of PT_injured/PT_repair states under IRI, particularly at 12 h (Figure 3D). Thus, the dominant PT phenotype at single-cell resolution can be summarized as an IRI-driven transition from homeostatic PT toward injured and repair-associated states that intensifies over time, providing the cellular-state framework for subsequent localization of PERK–ATF4–CHOP axis activity.

### 3.4 State-coupled UPR/apoptosis within PT

**FIGURE 4:**
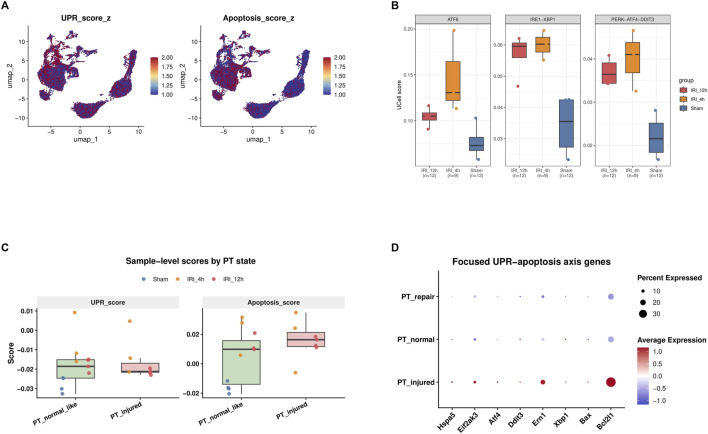
Pathway scoring within PT and spatial localization of key nodes **(A)** UMAP projection of UPR_score_z and Apoptosis_score_z shows focal enrichment of UPR- and apoptosis-related activity within specific PT subregions. **(B)** UCell-based branch scoring shows activation of UPR branch programs under IRI, including ATF6, IRE1–XBP1, and PERK–ATF4–DDIT3, with the PERK–ATF4–DDIT3 branch directly corresponding to the CHOP-centered axis prioritized in this study. **(C)** Sample-level scores by PT state show state-dependent remodeling of UPR and Apoptosis activity, with apoptosis-related activity more prominently elevated in PT_injured cells. **(D)** Focused dot plot of UPR–apoptosis axis genes shows state-dependent expression of ERS/UPR-related genes, including Hspa5, Eif2ak3/PERK, Atf4, Ddit3/CHOP, Ern1, and Xbp1, together with apoptosis-related genes including Bax and Bcl2l1.

Within the PT state framework, we next interrogated ERS/UPR and apoptosis-related pathway activity and their spatial organization, with particular attention to the PERK–ATF4–CHOP axis. Importantly, the main pathway-scoring conclusions were robust to the choice of scoring method. UPR_total and Apoptosis scores computed by UCell and Seurat AddModuleScore showed high concordance across PT cells, with correlation coefficients of 0.923 and 0.856, respectively, supporting the stability of the main pathway conclusions across alternative scoring approaches. When projected onto the PT UMAP, high UPR_score_z and Apoptosis_score_z signals formed focal regions rather than diffuse gradients, consistent with enrichment in injury-associated PT regions/states (Figure 4A).

At the branch level, UCell-based sample-resolved comparisons indicated activation of UPR branch programs under IRI, including ATF6, IRE1–XBP1, and PERK–ATF4–DDIT3, with the PERK–ATF4–DDIT3 branch representing the axis most directly aligned with the downstream CHOP-centered apoptotic program evaluated in this study (Figure 4B). Stratification by PT_state further revealed state-dependent remodeling: UPR scores differed between PT_normal_like and PT_injured cells, and apoptotic activity was more prominently elevated in PT_injured cells (Figure 4C). At the node level, canonical PERK–ATF4–CHOP axis genes, including Eif2ak3/PERK, Atf4, and Ddit3/CHOP, together with ER stress mediator Hspa5 and apoptosis-related genes such as Bax and Bcl2l1, showed state-dependent expression patterns across PT_normal_like, PT_injured, and PT_repair cells (Figure 4D). Collectively, these data indicate that PERK–ATF4–CHOP branch activity and apoptosis-related readouts are coupled to PT injury-state remodeling, with prominent convergence within injury-associated PT states.

### 3.5 Pseudotime trajectory analysis

**FIGURE 5:**
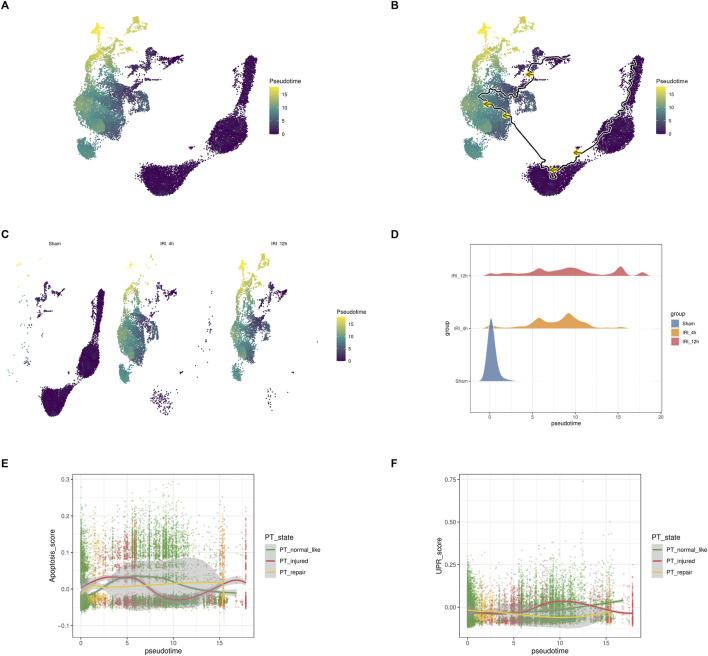
PT pseudotime trajectory and dynamic changes in UPR/apoptosis scores **(A)** Pseudotime UMAP shows a continuous gradient across the PT manifold. **(B)** Inferred trajectory overlay illustrates the principal path of PT state transition across the manifold. **(C)** Group-wise pseudotime UMAP plots show that Sham cells are mainly concentrated in early pseudotime regions, whereas IRI_4 h and IRI_12 h cells occupy broader and later pseudotime segments. **(D)** Ridge plots by group show an overall shift of IRI cells toward later pseudotime, with IRI_12 h showing a stronger displacement than IRI_4 h. **(E)** Apoptosis_score varies dynamically along pseudotime, with injury-associated PT states contributing prominently to higher apoptosis-related activity. **(F)** UPR_score shows a non-linear and state-dependent pattern along pseudotime, consistent with a phase-structured ER stress response during PT injury progression.

To capture PT state evolution as a dynamic process following IRI, we performed pseudotime analysis in the PT subset. Pseudotime values mapped onto the UMAP manifold as a continuous gradient spanning early and later regions of the PT trajectory (Figure 5A). The inferred trajectory further illustrated a principal path across the PT manifold, linking normal-like, injured, and repair-associated regions (Figure 5B). Condition-specific projections revealed systematic differences in trajectory occupancy between Sham and IRI, with Sham cells concentrated mainly in early pseudotime regions, whereas IRI_4h and IRI_12h cells occupied broader and later pseudotime segments (Figure 5C).

Ridge plots further demonstrated a global shift toward later pseudotime in IRI, with IRI_12h displaced more strongly than IRI_4h (Figure 5D). Linking functional programs to this trajectory revealed dynamic changes in apoptosis along pseudotime, with injury-associated PT states contributing prominently to higher apoptosis scores (Figure 5E). In parallel, UPR_score exhibited a non-linear and state-dependent relationship with pseudotime, consistent with a phase-structured ER stress response during PT injury progression rather than a simple cumulative escalation (Figure 5F). Thus, pseudotime analysis supports a process model in which PT cells transition from homeostatic states toward injured and repair-associated states after IRI, accompanied by dynamic remodeling of UPR- and apoptosis-related programs that are compatible with progressive engagement of the PERK–ATF4–CHOP-associated stress-apoptosis axis.

### 3.6 PT pseudo-bulk analysis

**FIGURE 6:**
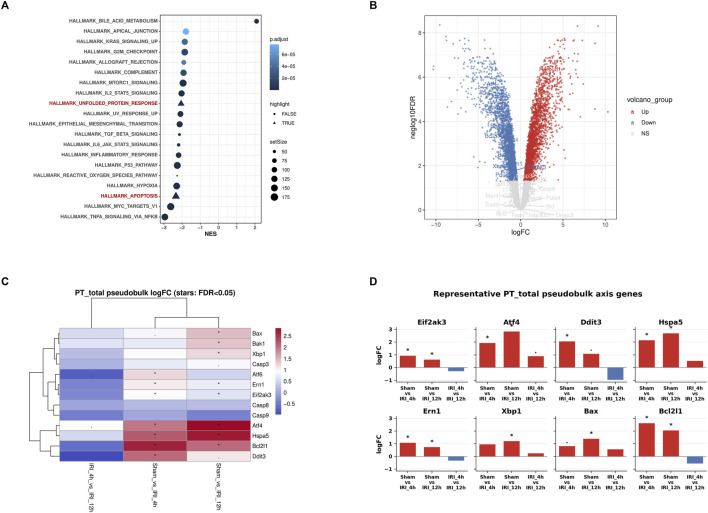
PT pseudo-bulk pathway enrichment and PERK–ATF4–CHOP axis-gene signals **(A)** Hallmark GSEA dot plot from PT pseudo-bulk analysis highlights Unfolded Protein Response and Apoptosis among the significantly altered pathways in the Sham *versus* IRI_12 h contrast. Because the contrast direction is Sham *versus* IRI_12h, negative NES values indicate enrichment toward the IRI_12 h condition. **(B)** PT pseudo-bulk volcano plot shows extensive transcriptional remodeling, with ERS/UPR- and apoptosis-related genes represented among the significantly altered signals. **(C)** Heatmap of axis-gene logFC across key PT pseudo-bulk contrasts shows coordinated changes in core UPR mediators, including Hspa5, Eif2ak3/PERK, Atf4, Ddit3/CHOP, Ern1/Xbp1, and Atf6, together with apoptosis-related genes including Bax, Bak1, Casp3/8/9, and Bcl2l1. Stars indicate FDR <0.05. **(D)** Bar plots of representative PT pseudo-bulk axis genes show contrast-level changes in PERK–ATF4–CHOP branch components, UPR-related mediators, and apoptosis-related effectors, including Eif2ak3/PERK, Atf4, Ddit3/CHOP, Hspa5, Ern1, Xbp1, Bax, and Bcl2l1. Stars indicate FDR <0.05.

To convert single-cell localization and scoring into reproducible, differential-evidence outputs, we generated PT pseudo-bulk profiles and focused on Sham *versus* IRI_12h as the core contrast to close the evidence loop. At the pathway level, Hallmark GSEA ranked Unfolded Protein Response and Apoptosis among the most significantly altered programs in the PT pseudo-bulk analysis. Because the contrast was plotted as Sham *versus* IRI_12h, the negative NES values indicate enrichment toward the IRI_12h condition (Figure 6A). Differential expression analysis revealed extensive transcriptional remodeling, with ERS/UPR- and apoptosis-related genes prominently represented among strongly differential signals (Figure 6B).

At the node level, log2FC heatmaps demonstrated coherent directional changes of core UPR mediators, including Hspa5, Eif2ak3/PERK, Atf4, Ddit3/CHOP, Ern1/Xbp1, and Atf6, together with apoptosis-related genes including Bax, Bak1, Casp3/8/9, and Bcl2l1 across the key PT pseudo-bulk contrasts (Figure 6C). Bar-plot summaries further highlighted contrast-level shifts of representative PERK–ATF4–CHOP branch components, UPR-related mediators, and apoptosis-related effectors, including Eif2ak3/PERK, Atf4, Ddit3/CHOP, Hspa5, Ern1, Xbp1, Bax, and Bcl2l1 (Figure 6D). Integrated with single-cell spatial mapping, PT state stratification, pathway scoring, and pseudotime analysis, these pseudo-bulk analyses support coordinated remodeling of PT UPR circuitry and apoptosis-related programs in the IRI condition, with convergent evidence pointing to the PERK–ATF4–CHOP axis as a prioritized ERS/UPR-associated stress-apoptosis module for downstream validation.

### 3.7 Overview of the porcine TCA resuscitation model

**FIGURE 7:**
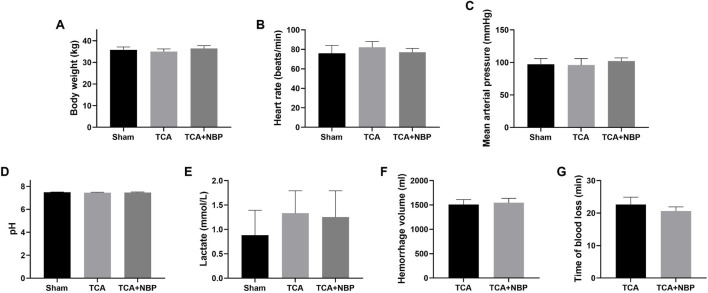
Baseline characteristics and modeling parameters across groups. **(A)** Body weight; **(B)** heart rate; **(C)** mean arterial pressure; **(D)** pH; **(E)** blood lactate; **(F)** blood loss; **(G)** bleeding duration. Data are presented as mean ± SD. For two-group comparisons, an independent-samples t-test was used; for three-group comparisons, one-way ANOVA was used.

Nineteen pigs were enrolled. Two animals initially assigned to the TCA group successfully underwent model induction and achieved ROSC, but failed to survive to the predefined 24 h post-ROSC endpoint and were therefore excluded from the final endpoint analysis. The final analyzable cohort comprised Sham (n = 5), TCA (n = 5), and TCA + NBP (n = 7). Baseline body weight, heart rate, and mean arterial pressure (MAP) were comparable across groups (Figures 7A–C). Arterial pH and lactate did not differ significantly at baseline (Figures 7D,E). During model induction, hemorrhage volume and hemorrhage duration were similar between the TCA and TCA + NBP groups (Figures 7F,G), supporting baseline balance and procedural comparability.

### 3.8 NBP is associated with improved early renal function after TCA resuscitation

**FIGURE 8:**
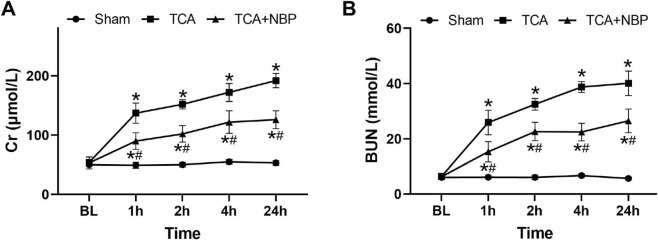
Renal function across groups. **(A)** Creatinine (Cr); **(B)** blood urea nitrogen (BUN). Data are presented as mean ± SD. Comparisons among three groups were performed using one-way ANOVA with Bonferroni correction. *P < 0.05 vs. Sham; #*P* < 0.05 vs. TCA.

Baseline serum creatinine (Cr) and blood urea nitrogen (BUN) were comparable across groups. Following resuscitation, both the TCA and TCA + NBP groups developed renal dysfunction, with Cr and BUN significantly elevated at all assessed time points (1–24 h) relative to Sham (P < 0.05). Notably, compared with the TCA group, NBP treatment significantly reduced Cr and BUN at each corresponding post-ROSC time point (all P < 0.05) (Figures 8A,B). At 24 h after ROSC, serum Cr was lower in the TCA + NBP group than in the TCA group (mean difference: −66.23 μmol/L, 95% CI: −84.19 to −48.28; P = 9.27 × 10^−6^), and BUN was also reduced (mean difference: −13.60 mmol/L, 95% CI: −19.28 to −7.92; P = 3.32 × 10^−4^). These findings support an association between NBP treatment and reduced early post-resuscitation AKI severity within the 24 h observation window.

### 3.9 Renal KIM-1 and NGAL expression

**FIGURE 9:**
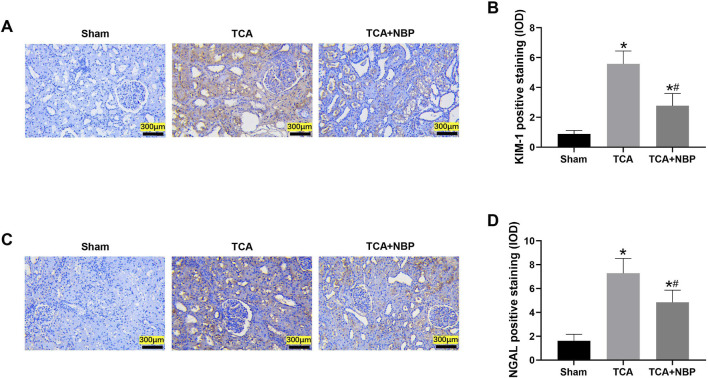
Expression levels of KIM-1 and NGAL in renal tissue across groups. **(A,B)** Representative immunohistochemistry images (×200) for KIM-1 and quantification by integrated optical density (IOD). **(C,D)** Representative immunohistochemistry images (×200) for NGAL and quantification by IOD. Data are presented as mean ± SD. Comparisons among three groups were performed using one-way ANOVA with Bonferroni correction. *P < 0.05 vs. Sham; #*P* < 0.05 vs. TCA.

Immunohistochemistry showed minimal KIM-1 and NGAL expression in Sham kidneys. Following TCA resuscitation, both markers were induced in the TCA and TCA + NBP groups, with IOD values significantly higher than Sham. Compared with TCA controls, NBP reduced renal KIM-1 expression (mean difference: −2.81 IOD units, 95% CI: −3.90 to −1.72; P = 1.88 × 10^−4^) and NGAL expression (mean difference: −2.43 IOD units, 95% CI: −3.89 to −0.98; P = 0.0039) (Figures 9A–D), further supporting an association between NBP treatment and reduced renal tubular injury after TCA resuscitation.

### 3.10 Histopathologic changes in renal tissue

**FIGURE 10:**
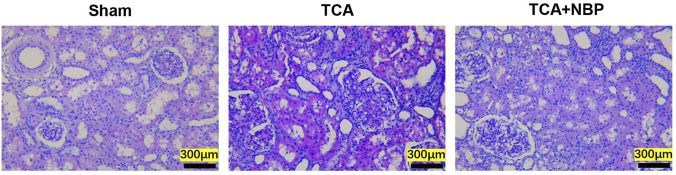
Gross histopathological injury assessment of renal tissue across groups (representative HE-stained images, ×200).

H&E staining demonstrated largely preserved renal architecture in Sham animals. In contrast, the TCA group exhibited canonical features of acute kidney injury, including glomerular edema/degeneration, necrotic cell appearance, tubular epithelial sloughing, and inflammatory cell infiltration. These histopathologic lesions were substantially attenuated in the TCA + NBP group relative to TCA controls (Figure 10), consistent with an association between NBP treatment and less severe morphological kidney injury after TCA resuscitation.

### 3.11 Assessment of renal apoptosis

**FIGURE 11:**
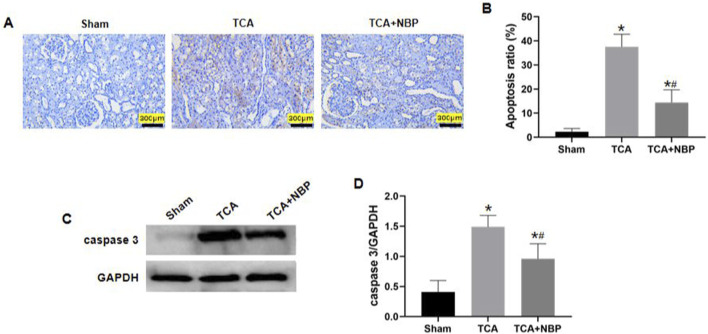
Renal-cell apoptosis across groups. **(A,B)** Representative TUNEL-stained images (×200) and quantification of apoptotic cell percentage. **(C,D)** Representative Western blot bands for Caspase-3 and densitometric quantification. Data are presented as mean ± SD. Group comparisons were performed using one-way ANOVA with Bonferroni correction. *P < 0.05 vs. Sham; #*P* < 0.05 vs. TCA.

TUNEL staining revealed negligible apoptosis in Sham kidneys, whereas apoptotic cells were increased in both TCA and TCA + NBP groups, yielding higher apoptotic indices than Sham. Compared with TCA controls, NBP significantly reduced the apoptotic index (mean difference: −23.10%, 95% CI: −29.96 to −16.25; P = 2.03 × 10^−5^) (Figures 11A,B). Consistently, Western blotting showed that caspase-3/GAPDH was lower in the TCA + NBP group than in the TCA group (mean difference: −0.53, 95% CI: −0.85 to −0.20; P = 0.0058) (Figures 11C,D). Together, these findings are consistent with reduced apoptosis-related readouts in renal tissue after resuscitation in the NBP-treated group.

### 3.12 Expression of ERS/UPR-associated and apoptosis-related proteins

**FIGURE 12:**
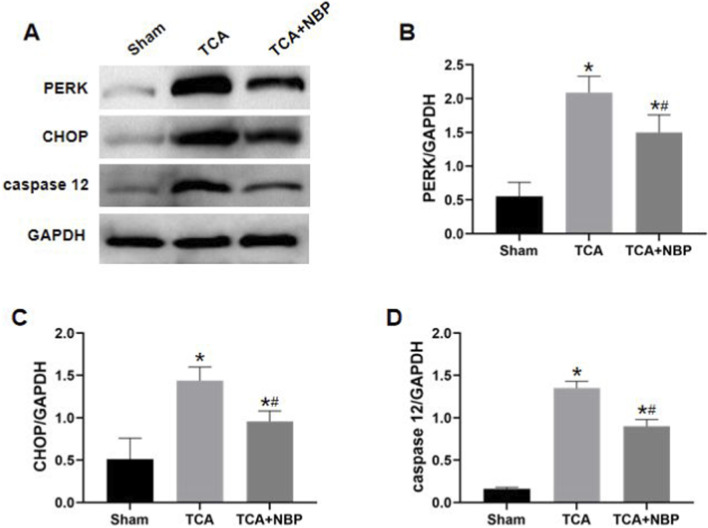
Expression of ERS/UPR-associated proteins and apoptosis-related proteins in renal tissue across groups. **(A-D)** Representative Western blot bands and densitometric quantification of PERK (Eif2ak3), CHOP(Ddit3), and Caspase-12. Data are presented as mean ± SD. Group comparisons were performed using one-way ANOVA with Bonferroni correction. *P < 0.05 vs. Sham; #*P* < 0.05 vs. TCA.

Western blotting demonstrated significant induction of PERK, CHOP, and caspase-12 in renal tissue from both the TCA and TCA + NBP groups relative to Sham (P < 0.05), consistent with increased ERS/UPR-associated stress signaling and elevated apoptosis-related protein readouts after resuscitation. Compared with TCA controls, NBP treatment was associated with lower normalized protein abundance of PERK (mean difference: −0.59, 95% CI: −0.96 to −0.23; P = 0.0058), CHOP (mean difference: −0.48, 95% CI: −0.69 to −0.27; P = 8.34 × 10^−4^), and caspase-12 (mean difference: −0.45, 95% CI: −0.56 to −0.34; P = 1.52 × 10^−5^) (Figures 12A–D). Together with the concordant reduction in TUNEL positivity and caspase-3 abundance, these findings indicate that NBP treatment was associated with lower ERS/UPR-associated protein expression and reduced apoptosis-related readouts. However, because pathway-specific perturbation was not performed, these data should be interpreted as supportive of a mechanistic association rather than proof of a causal PERK–CHOP-dependent mechanism.

## 4 Discussion

In a porcine TCA resuscitation model, we observed that NBP administration was associated with attenuated post-resuscitation AKI. Relative to TCA controls, NBP lowered serum Cr and BUN, reduced renal KIM-1 and NGAL expression, improved histopathologic injury, and decreased TUNEL-positive cells. In parallel, ERS/UPR-associated and apoptosis-related proteins, including PERK, CHOP, caspase-12, and caspase-3, were reduced. These findings suggest that NBP-associated early renal protection may be accompanied by attenuated ERS/UPR-associated apoptotic readouts; however, they do not establish that suppression of this pathway is necessary or sufficient for the observed protection. A central design feature of this study is the deliberate alignment of tissue-level transcriptomic signals, single-cell–resolved cell-type/cell-state attribution, and *in vivo* node-level readouts within a single interpretive chain. This triangulation is intended to reduce cross-platform inferential ambiguity and to ensure that key conclusions are corroborated across biological scales, thereby improving reproducibility and mechanistic plausibility.

In bulk transcriptomic analyses, Hallmark enrichment revealed coordinated induction of proteostasis disruption–associated ERS/UPR programs together with an Apoptosis program, alongside enrichment of hypoxia- and inflammation-linked pathways characteristic of ischemia–reperfusion injury. Importantly, these enrichment results capture coordinated transcript-level shifts and do not, by themselves, constitute direct evidence of protein-level pathway activationBy decomposing UPR activity into its canonical branches, we further prioritized the PERK–ATF4–CHOP branch as the UPR-related axis most directly aligned with the CHOP-centered apoptotic readouts evaluated in the porcine validation model. This bias is biologically coherent: under homeostatic or adaptive stress, the UPR reduces translational burden and expands folding/degradation capacity to restore ER proteostasis; under intense or sustained stress, UPR signaling can pivot toward cell-fate regulation, with the PERK–eIF2α–ATF4–CHOP branch particularly prone to driving pro-apoptotic transcriptional outputs that couple to downstream execution machinery (Walter and Ron, 2011; Hetz, 2012). Consistent with this framework, reviews of ERS/UPR in AKI emphasize that PERK–ATF4–CHOP is frequently linked to cell death, inflammatory amplification, and maladaptive repair across injury models (Porter et al., 2022). Accordingly, we interpret our results as evidence of coordinated ERS/UPR-associated and apoptosis-related remodeling, with the PERK-ATF4-CHOP axis prioritized as a biologically plausible stress-apoptosis module rather than as a proven causal pathway.

In single-cell analyses, UPR/ERS and apoptosis signals were not uniformly distributed across nephron cell types; instead, they concentrated within proximal tubule (PT) injury/repair lineages and were most pronounced in PT states reflecting more severe injury or impaired repair. This localization aligns with the stability of major renal cell-type identification in kidney single-cell atlases (Lake et al., 2019) and with reports that PT cells traverse multiple injury–repair trajectories with dynamic state transitions in AKI/IRI (Rudman-Melnick et al., 2020). Notably, failed-repair–associated PT subpopulations described in mouse AKI studies link PT-specific injury states to maladaptive recovery programs (Kirita et al., 2020). Human kidney single-nucleus multi-omic profiling (RNA + ATAC) further supports the concept that failed injury response/failed repair corresponds to relatively stable regulatory networks and transcription factor–driven states, providing a testable framework for persistence and chronicity after AKI (Ledru et al., 2024). KPMP-related syntheses similarly emphasize that injured PT cell states are reproducible and are coupled to regeneration programs, inflammatory crosstalk, and fibrosis risk in human kidney tissue (Janosevic and De Luca, 2025). In this context, our selection of PERK, CHOP, and downstream caspases as validation nodes was intended to align PT state–resolved cellular phenotypes with biologically plausible ERS/UPR-associated apoptotic readouts, rather than to posit an exclusive, single-pathway attribution.

Building on this localization, pseudotime analysis along the PT injury trajectory suggested dynamic remodeling of stress and apoptosis-related programs: UPR activity varied in a non-linear, phase-structured manner, whereas apoptosis-related activity was more prominently associated with injury-enriched PT states along the trajectory. This pattern supports the inference that an early reperfusion window may be particularly drug-responsive; attenuating excessive PERK–ATF4–CHOP transcriptional output early in the injury course could plausibly blunt feed-forward propagation toward caspase-dependent execution, thereby slowing AKI progression. Time-resolved studies in mouse renal IRI provide supportive context: GRP78/BiP peaks earlier after reperfusion, while CHOP and caspase-12 become more prominent in subsequent windows, with necrosis/apoptosis readouts increasing later (Gao et al., 2012). Pharmacologically, ERS inhibitors such as 4-PBA or TUDCA suppress ERS responses and reduce apoptosis and functional impairment in mouse renal IRI (Gu et al., 2018). Moreover, interventions initiated after injury onset (e.g., beginning 1 h post-IRI) can still downregulate UPR-related transcription factors and improve injury metrics, directly supporting the concept that early post-insult treatment can remain beneficial (Diaz-Bulnes et al., 2024). Consistent with this framework, we initiated intravenous NBP infusion immediately after ROSC and observed reduced PERK, CHOP, and caspase-12/3, fewer TUNEL-positive cells, and concomitant improvement in Cr and BUN. These findings are compatible with attenuation of ERS/UPR-associated apoptotic signaling during an early therapeutic window, but they do not demonstrate direct interruption of the ERS-to-apoptosis cascade.

Prior work has consistently implicated NBP as renoprotective across diverse kidney injury paradigms, often accompanied by attenuation of inflammatory tone, oxidative stress, and cell-death readouts. In chronic and metabolic kidney injury contexts, continuous NBP administration improved renal function indices and histopathology in a spontaneously hypertensive rat model of hypertensive nephropathy, concomitant with reduced oxidative stress and inflammatory markers (Zhu et al., 2015). In db/db diabetic kidney disease, DL-NBP improved renal function and fibrotic phenotypes in association with reduced ERS and decreased podocyte apoptosis (Xu et al., 2021). In acute ischemia–reperfusion settings, NBP pre-treatment in mouse renal IRI improved creatinine and mitigated tissue injury while suppressing inflammation-, oxidative stress-, and cell-death–associated outputs (Dong et al., 2021).

Complementing these NBP-focused observations, a broader intervention literature suggests that attenuation of ERS/UPR—particularly CHOP-related pro-apoptotic signaling—is biologically relevant to renal injury mitigation in experimental settings. In a rat resuscitation model, edaravone improved renal function concomitant with reductions in GRP78, CHOP, and apoptosis-related proteins (Fu et al., 2020). In rat renal IRI, upregulation of Intermedin reduced GRP78, CHOP, and caspase-12 and was associated with decreased apoptosis (Wang et al., 2015). Genetic attenuation of upstream sensing via GPR4 knockout in mouse renal IRI reduced CHOP and cleaved caspase-3, decreased TUNEL positivity, and improved renal injury (Dong et al., 2017). Taken together, these findings support the biological plausibility that the renal effects observed with NBP treatment may be accompanied by suppression of ERS/UPR-associated pro-apoptotic signaling, particularly PERK–ATF4–CHOP-related readouts. In this light, the concordant phenotypic improvement and downregulation of this axis in our porcine model is best interpreted as support for a pre-specified mechanistic framework rather than an incidental observation.

With respect to modeling and generalizability, it is essential to articulate both the boundaries and the value of extrapolation. On the one hand, our mechanistic nomination and cell-of-origin localization relied primarily on public mouse AKI/IRI datasets, which do not fully recapitulate the systemic low-flow–reperfusion physiology that characterizes post–cardiac arrest syndrome. Given the substantial heterogeneity of AKI, strategies that appear effective in animal models can attenuate during clinical translation; therefore, the stability and scope of applicability of the prioritized pathways require validation in longitudinal cohorts and multi-omic frameworks more directly aligned with post-resuscitation syndromes (Ortiz et al., 2015). On the other hand, we validated key nodes and phenotypes in a porcine TCA resuscitation model. As a large mammal, the pig more closely approximates human organ scale, hemodynamics, and several physiologic parameters; moreover, porcine urinary system anatomy and physiology share substantial similarities with humans, supporting evaluation of kidney injury and pharmacologic effects in a clinically proximate systemic IRI context (Lunney et al., 2021; Huang et al., 2021). In addition, porcine kidney single-cell atlases indicate transcriptomic concordance between pig and human renal cell types and suggest the feasibility of cross-species cell-type mapping in key compartments such as PT. Nevertheless, such comparability supports translational relevance rather than complete equivalence between species or injury contexts (Yao et al., 2025).

Several limitations should be acknowledged. First, cross-species evidence stitching imposes residual uncertainty on generalizability. Mechanistic discovery and cellular attribution were primarily derived from public mouse AKI/IRI datasets, whereas node-level validation and phenotypic assessment were performed in a porcine TCA resuscitation model. Although the porcine TCA model provides a clinically relevant large-animal systemic ischemia–reperfusion context, mouse renal IRI does not fully recapitulate the systemic low-flow, hemorrhagic, inflammatory, and resuscitation-related physiology of porcine TCA. Species-specific differences in immune responses, metabolic context, nephron cellular composition, drug metabolism, and post-injury state landscapes may influence pathway effect-size estimates, inferred state proportions, treatment responsiveness, and the precision of therapeutic-window inference. Therefore, the present cross-species framework supports biological plausibility but does not establish complete mechanistic equivalence between murine renal IRI and porcine post-resuscitation AKI. Additionally, although pathway scores were cross-validated using both Seurat AddModuleScore and UCell, we did not systematically evaluate alternative integration parameters, such as different Harmony settings, principal component ranges, or clustering resolutions. Therefore, the single-cell conclusions should be interpreted as robust to pathway-scoring strategy, while potential sensitivity to integration-parameter choices cannot be fully excluded. Second, the porcine experiment was limited by a modest final sample size. Two animals initially assigned to the TCA group did not survive to the predefined 24 h post-ROSC endpoint, resulting in final analyzable group sizes of Sham n = 5, TCA n = 5, and TCA + NBP n = 7. No formal *a priori* power analysis was conducted. Expansion of the cohort was constrained by ethical considerations, the 3Rs principle, experimental feasibility, and the resource-intensive nature of technically demanding large-animal resuscitation studies. Although renal functional, histological injury, apoptosis, and ERS/UPR-associated protein readouts showed directionally consistent changes, treatment-effect estimates remain imprecise and should be validated in larger, prospectively powered large-animal studies. Third, our *in vivo* observation window was largely confined to the acute post-ROSC period and does not address longer-term outcomes, including sustained renal recovery, AKI-to-CKD transition, or survival benefit. Prior work on IRI-related acute-to-chronic trajectories indicates that partial normalization of acute-phase indices can still evolve into persistent injury and structural remodeling, motivating extended follow-up incorporating fibrosis, remodeling, renal functional recovery, and survival endpoints (Liu et al., 2017). Therefore, the present study supports early biological efficacy within a 24 h observation window, but not durable renoprotection or clinical outcome benefit. Fourth, causal resolution remains limited. Although phenotypic improvement with NBP paralleled downregulation of the PERK–ATF4–CHOP branch and downstream caspase-related readouts, we did not establish the necessity or sufficiency of this pathway in post-resuscitation AKI via pathway-specific genetic or pharmacologic perturbation. Nor did we disentangle direct suppression of ERS/UPR-associated pro-apoptotic readouts from indirect mitigation of ERS/UPR through improved systemic perfusion, reduced oxidative stress, or attenuated inflammation. Therefore, the current evidence is better interpreted as supporting a mechanistically plausible association rather than proving a causal PERK–ATF4–CHOP-dependent mechanism. Fifth, dose and timing were not systematically optimized. Only a single NBP dose and a fixed post-ROSC initiation window were tested, precluding definition of an optimal dose–response relationship and the boundaries of the effective therapeutic window. Future work should incorporate dose escalation, delayed-treatment designs, serial post-ROSC sampling, and initiation-time stratification within the same model to derive clinically actionable dosing parameters and to determine whether early modulation of ERS/UPR-associated apoptotic signaling translates into sustained renal and survival benefits.

In light of these limitations, future work could be strengthened along three axes—causal validation, mechanistic closure, and translational parameterization. First, within the same model, targeted PERK pathway inhibition or blockade downstream of CHOP could be tested alone and in combination with NBP to assess additivity or epistasis (Lehár et al., 2008). Ideally, genetic knockdown/rescue experiments should be established in mouse IRI or *in vitro* PT cell/organoid systems first, followed by pharmacologic confirmation of key readouts in the porcine TCA model (Hetz and Papa, 2018). Second, proximal mechanistic measurements should be expanded to include phospho-eIF2α, ATF4 abundance and nuclear localization (Cheng et al., 2024), together with more direct apoptotic execution markers such as cleaved caspase-3 and/or PARP cleavage. Sampling multiple time points after ROSC would enable reconstruction of the temporal sequence “ERS initiation → CHOP output → executioner activation” (Vitale et al., 2023). Third, upstream drivers—including ROS and mitochondrial dysfunction—should be quantified in parallel and explicitly coupled to ERS readouts. Systematic comparison of dosing and initiation time would further support rational optimization of combination strategies and clinically translatable treatment parameters (Inoue et al., 2019).

## 5 Conclusion

In summary, integrative analyses of bulk and single-cell transcriptomic data highlighted coordinated activation of ERS/UPR- and apoptosis-related programs after renal ischemia–reperfusion, with enrichment of a PERK–ATF4–CHOP branch and downstream caspase-related signature in injured proximal tubule states. In a porcine traumatic cardiac arrest resuscitation model, early post-ROSC NBP treatment was associated with attenuation of early post-resuscitation AKI within a 24 h observation window, as reflected by improved Cr/BUN, reduced KIM-1/NGAL, and ameliorated histopathologic injury. These phenotypic changes occurred in parallel with decreased renal apoptosis and lower expression of ERS/UPR-associated and apoptosis-related protein readouts, supporting a potential association between these molecular changes and early kidney injury severity. Longer-term studies are required to determine whether these early biological effects translate into sustained renal recovery or survival benefit.

## Data Availability

The datasets presented in this study can be found in online repositories. The names of the repository/repositories and accession number(s) can be found in the article/Supplementary Material.
