# Supplementary figures and images for: Integrated transcriptomics identifies ER stress–associated apoptosis in post-resuscitation AKI and supports early Dl-3-n-butylphthalide–associated renoprotection in a porcine TCA model
Source: Front Pharmacol. 2026 Jun 4;17:1841271. doi: 10.3389/fphar.2026.1841271 (PMC13275486; doi:10.3389/fphar.2026.1841271)

Supplementary File: Western blot raw band images

#
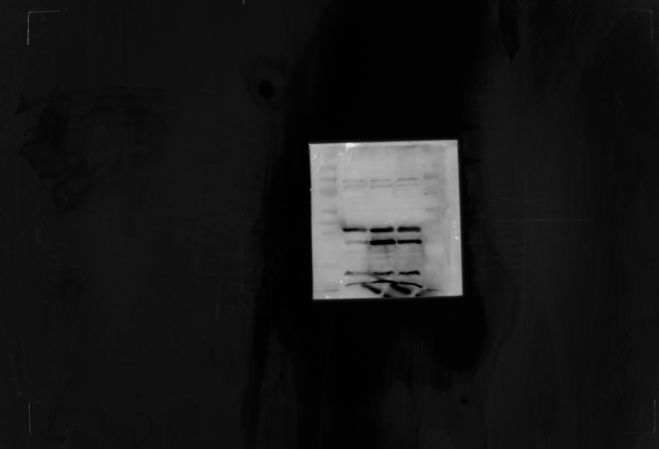

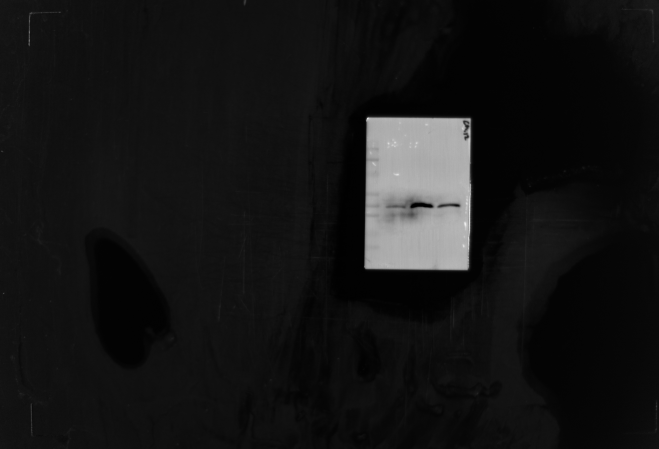

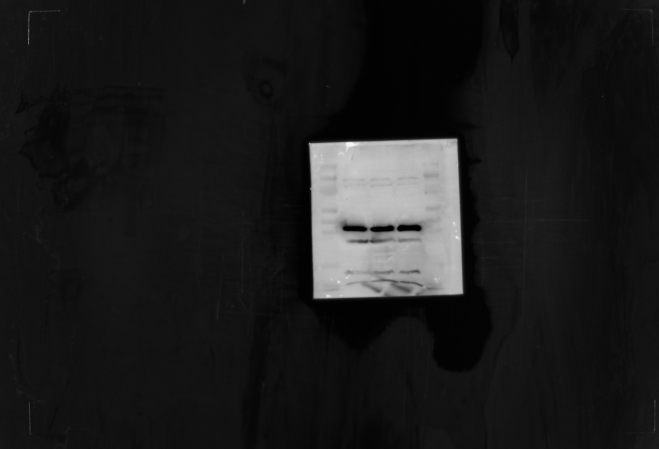

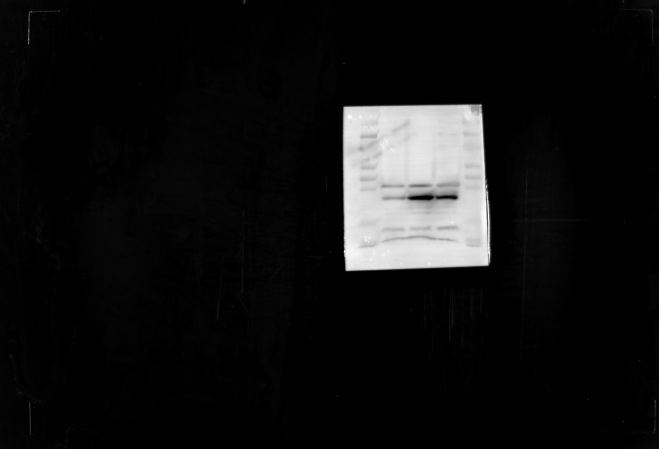

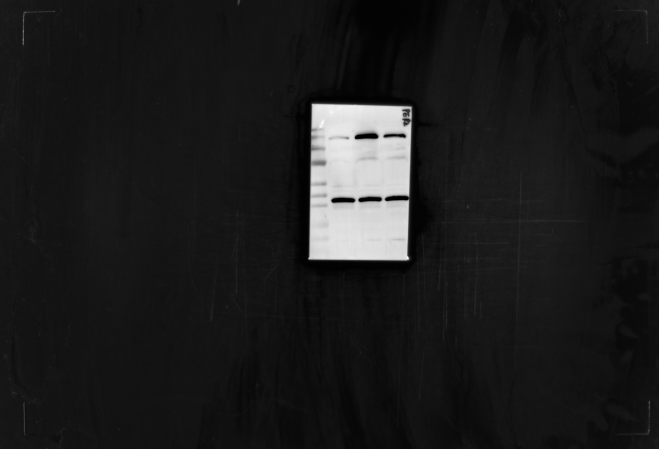

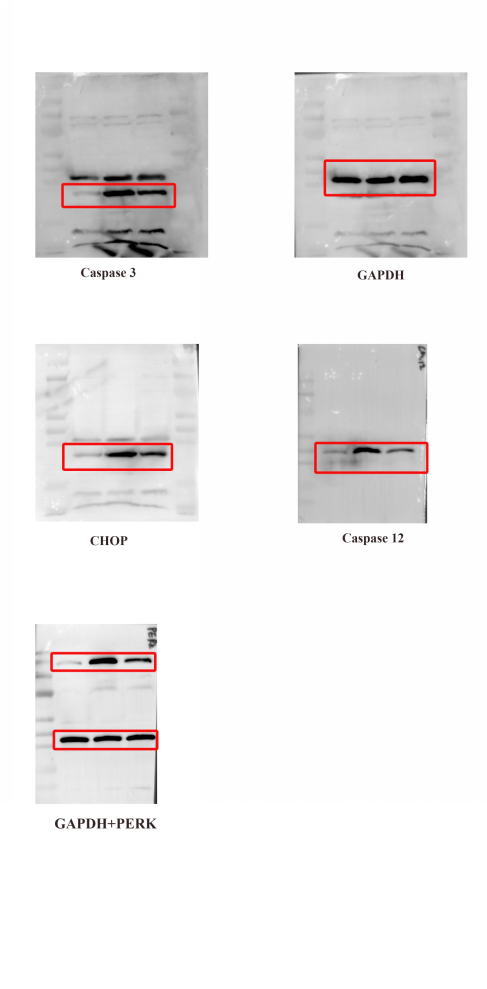

Supplement: Supplementary file 1 [file DataSheet4.docx]
