# Supplementary material for: Integrated transcriptomics identifies ER stress–associated apoptosis in post-resuscitation AKI and supports early Dl-3-n-butylphthalide–associated renoprotection in a porcine TCA model
Source: Front Pharmacol. 2026 Jun 4;17:1841271. doi: 10.3389/fphar.2026.1841271 (PMC13275486; doi:10.3389/fphar.2026.1841271)
Supplement: Supplementary file 2 [file Supplementaryfile1.docx]

Supplementary File 1: Animal Experiment Figures

# Section: Physiological and biochemical indicator plots


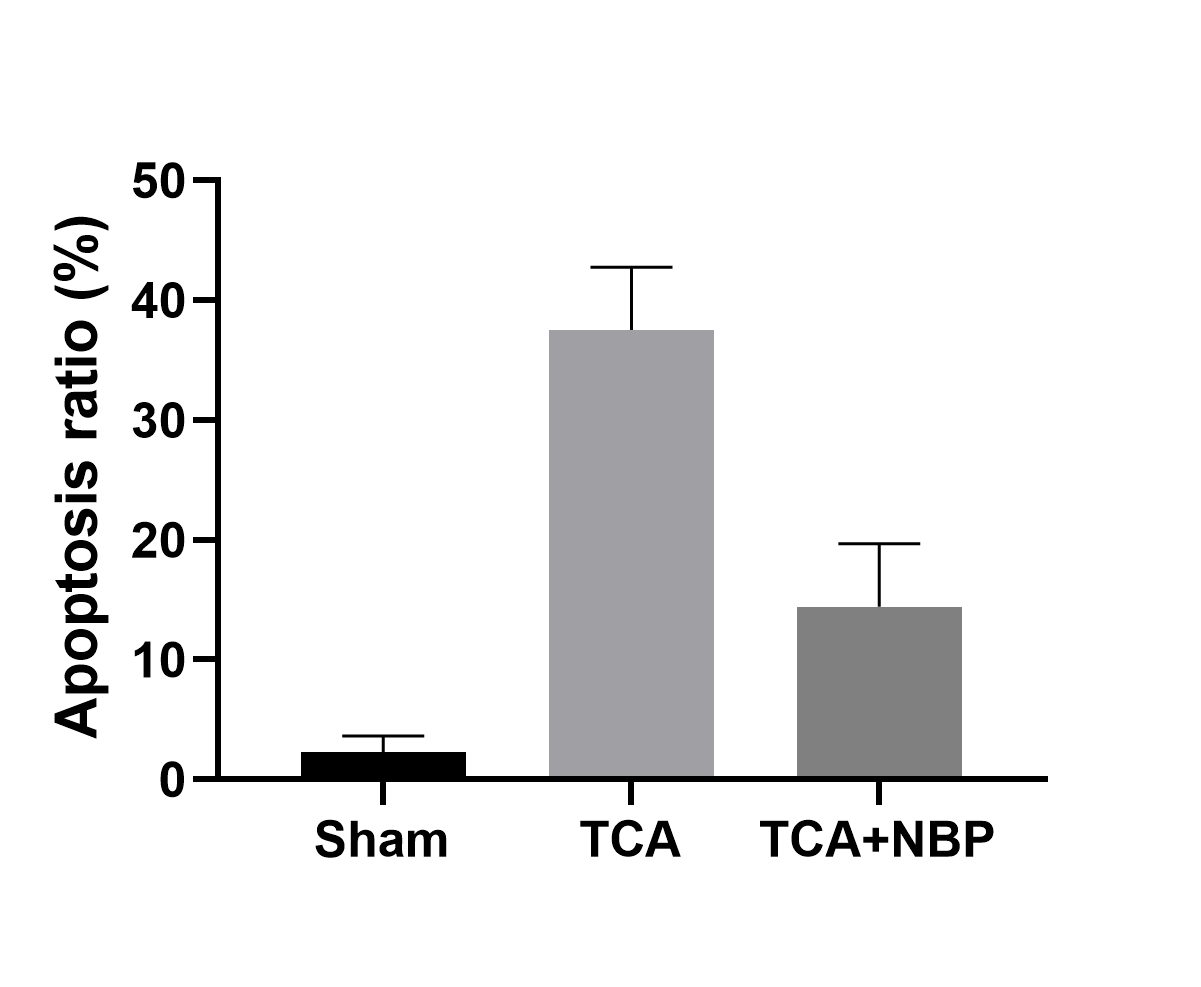


**Figure S1-023. Physiological/biochemical indicator chart; sample or target: AR**


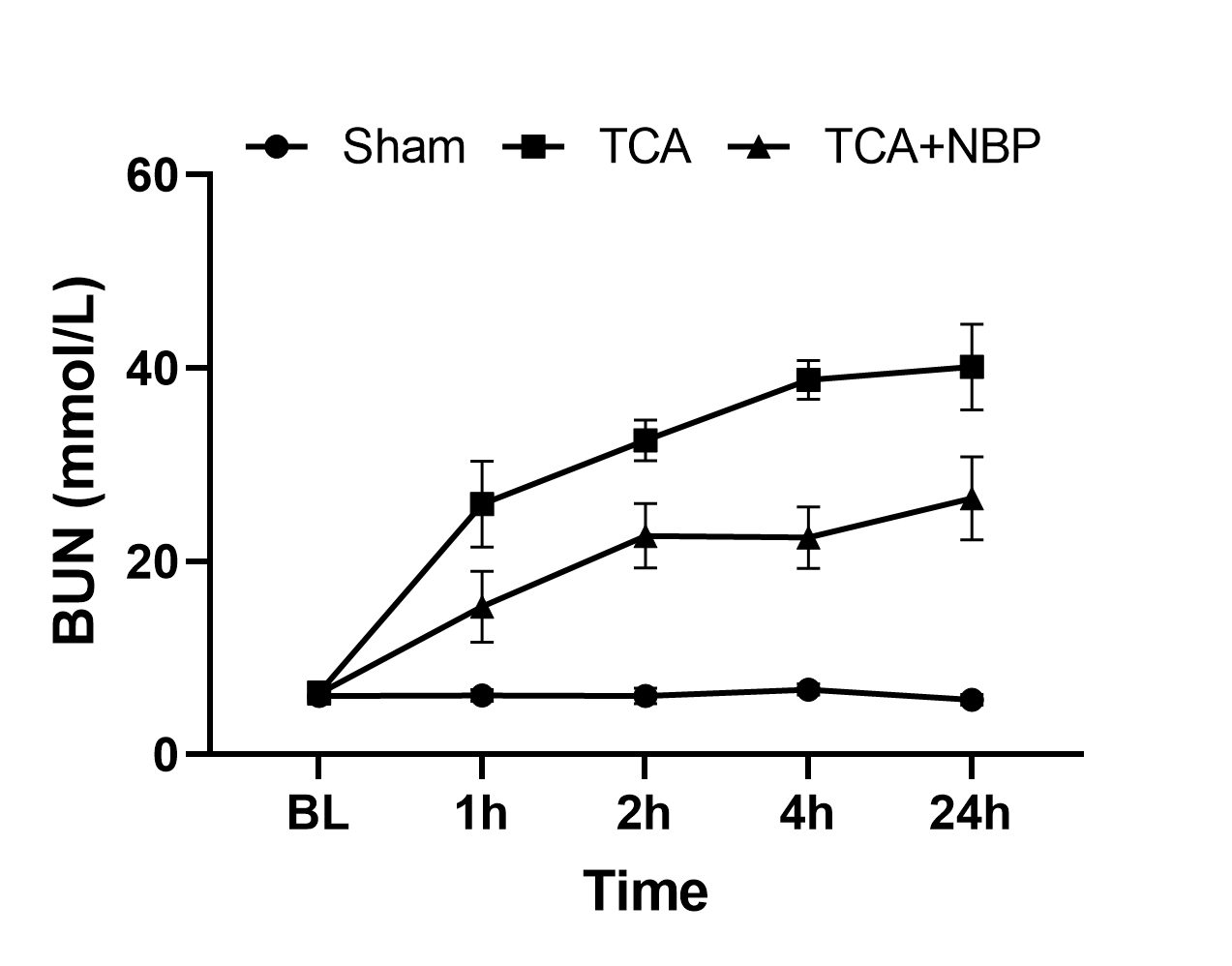


**Figure S1-024. Physiological/biochemical indicator chart; sample or target: BUN**


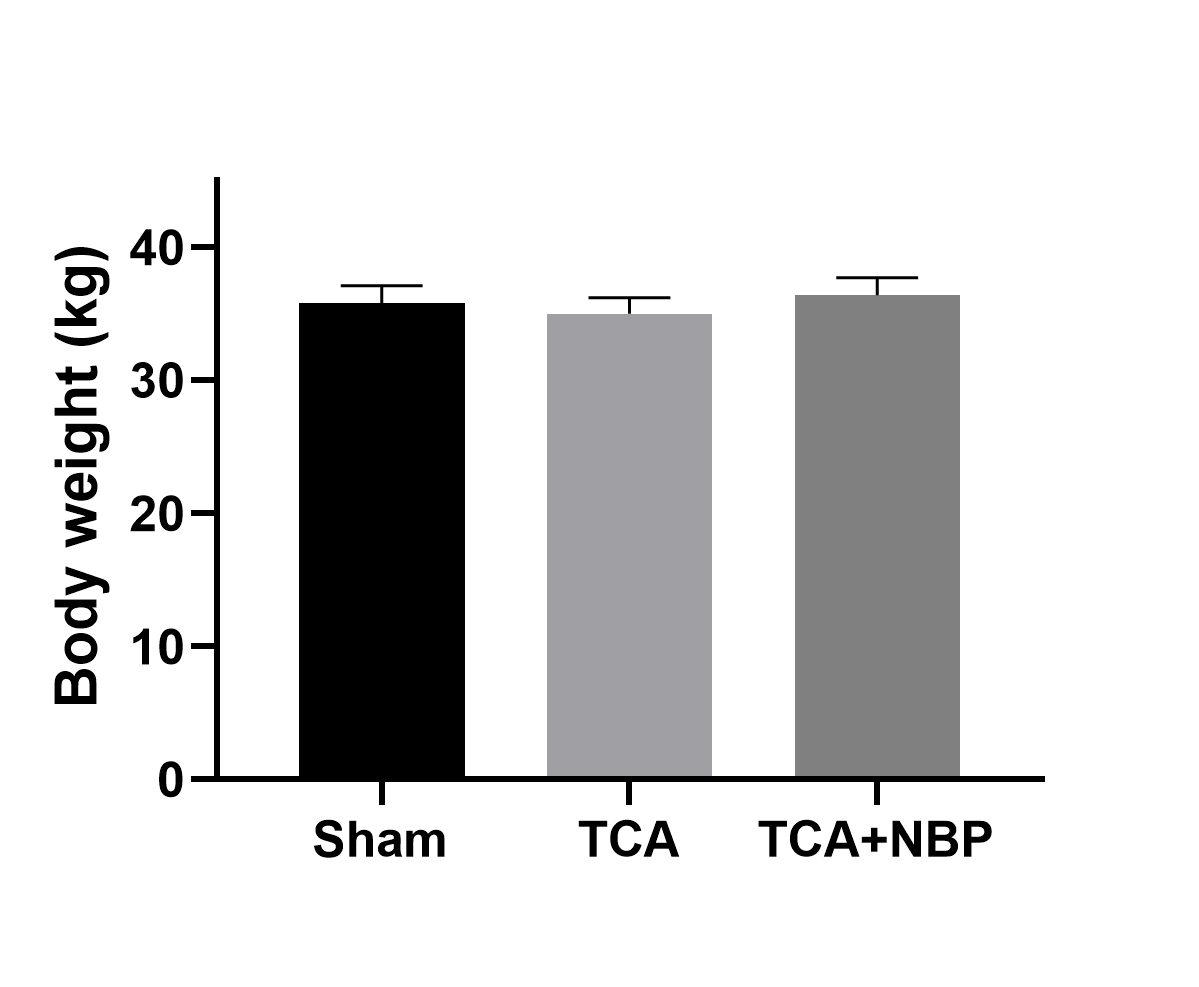


**Figure S1-025. Physiological/biochemical indicator chart; sample or target: BW**


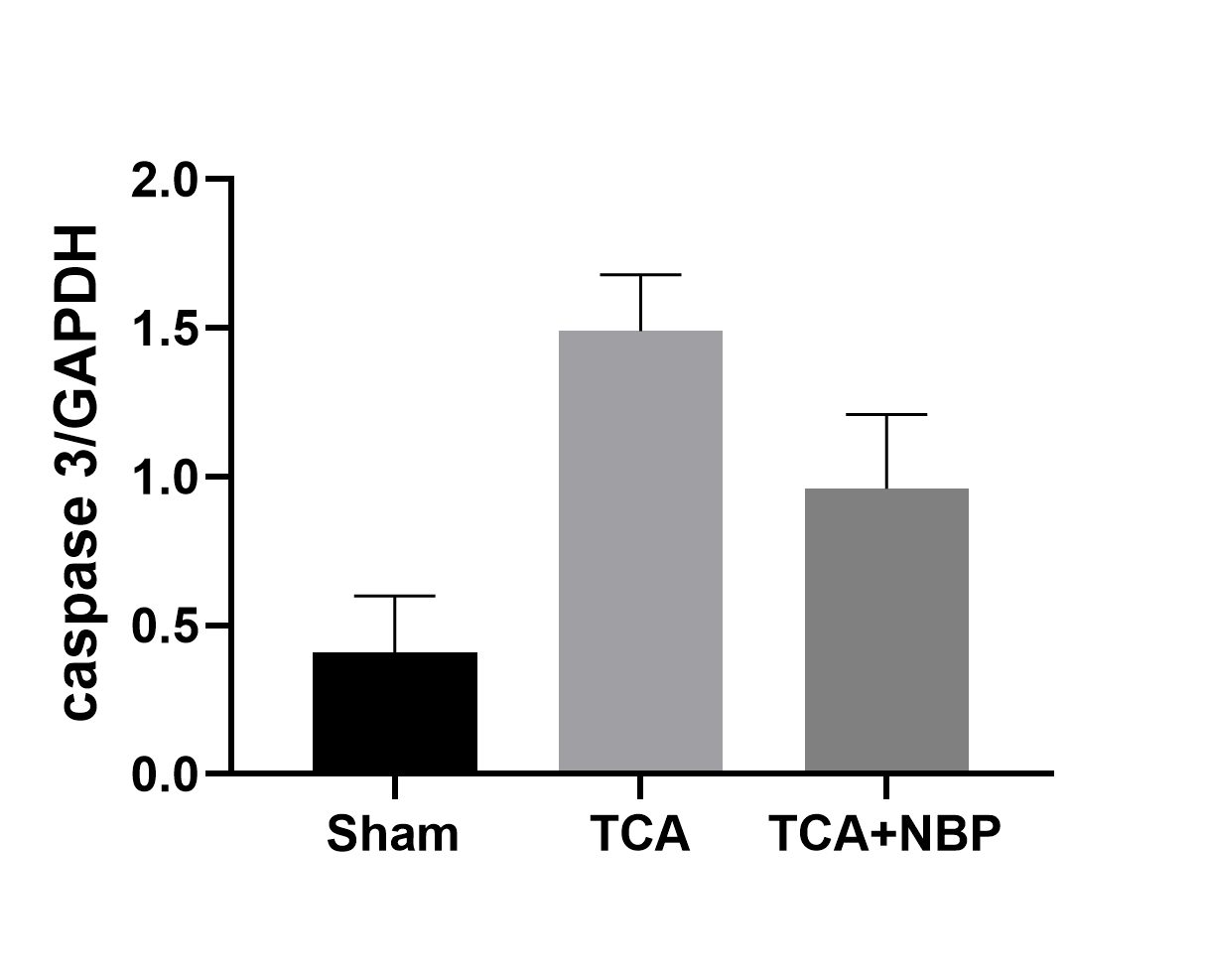


**Figure S1-026. Physiological/biochemical indicator chart; sample or target: CAS3**


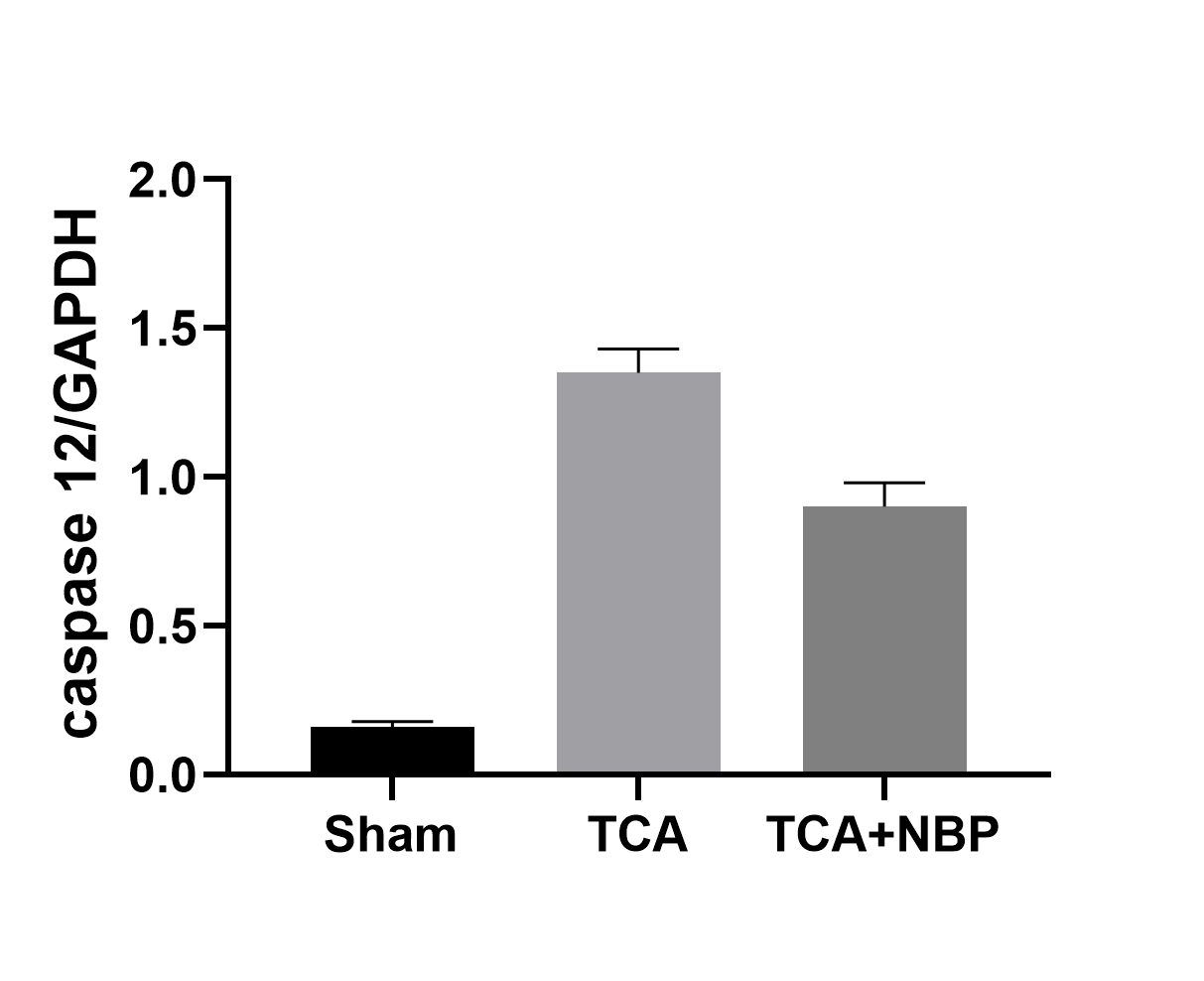


**Figure S1-027. Physiological/biochemical indicator chart; sample or target: CAS12**


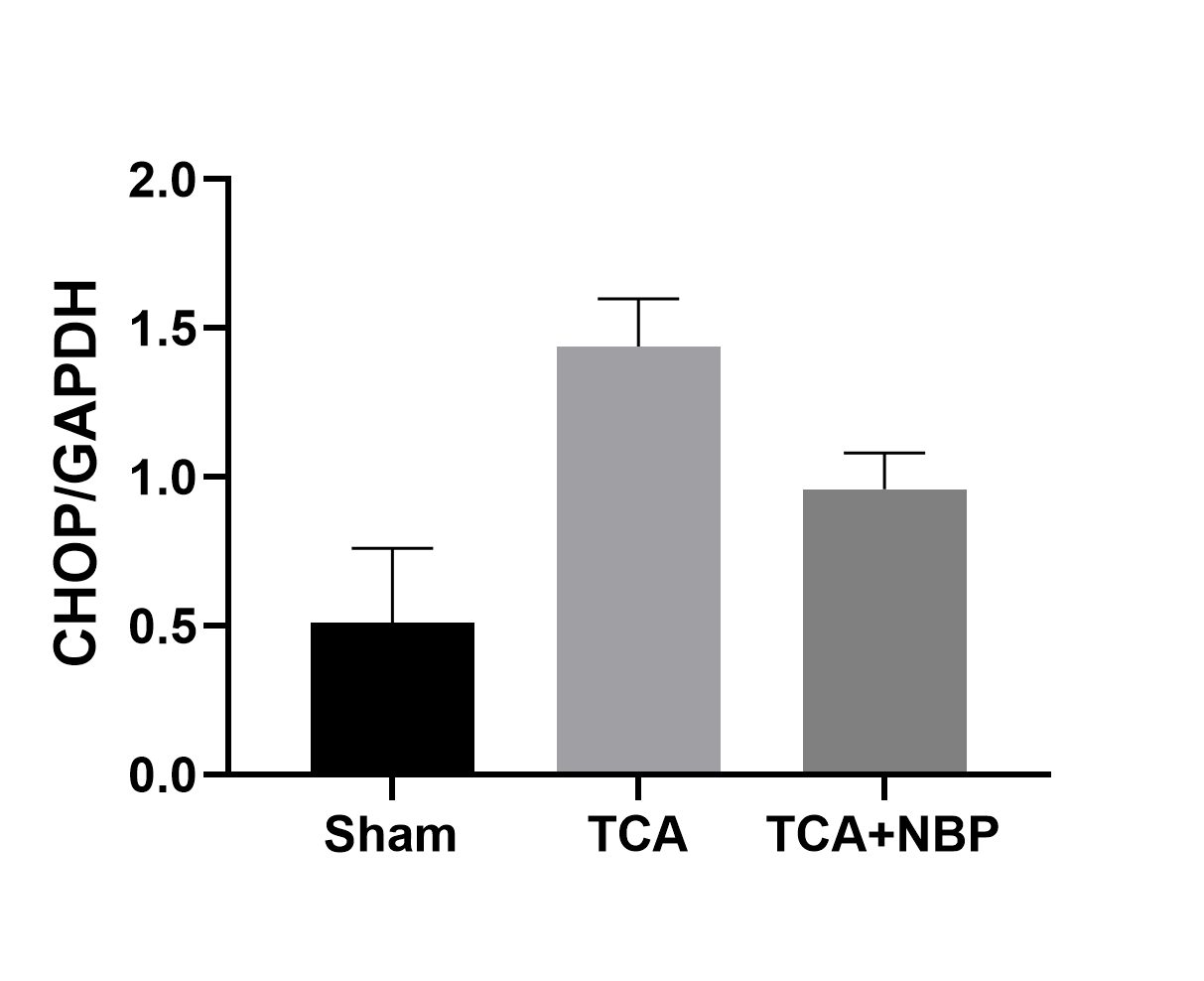


**Figure S1-028. Physiological/biochemical indicator chart; sample or target: CHOP**


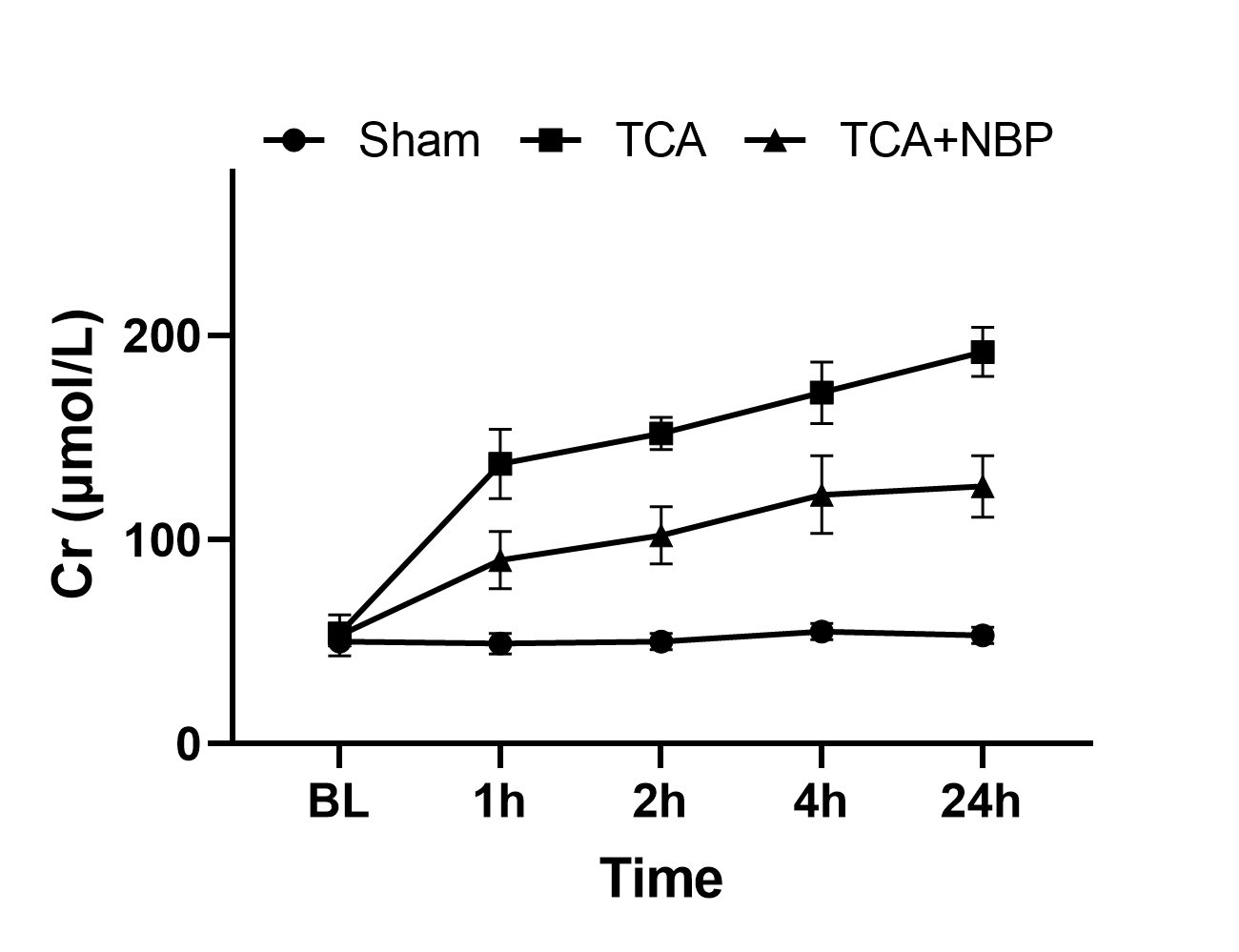


**Figure S1-029. Physiological/biochemical indicator chart; sample or target: Cr**


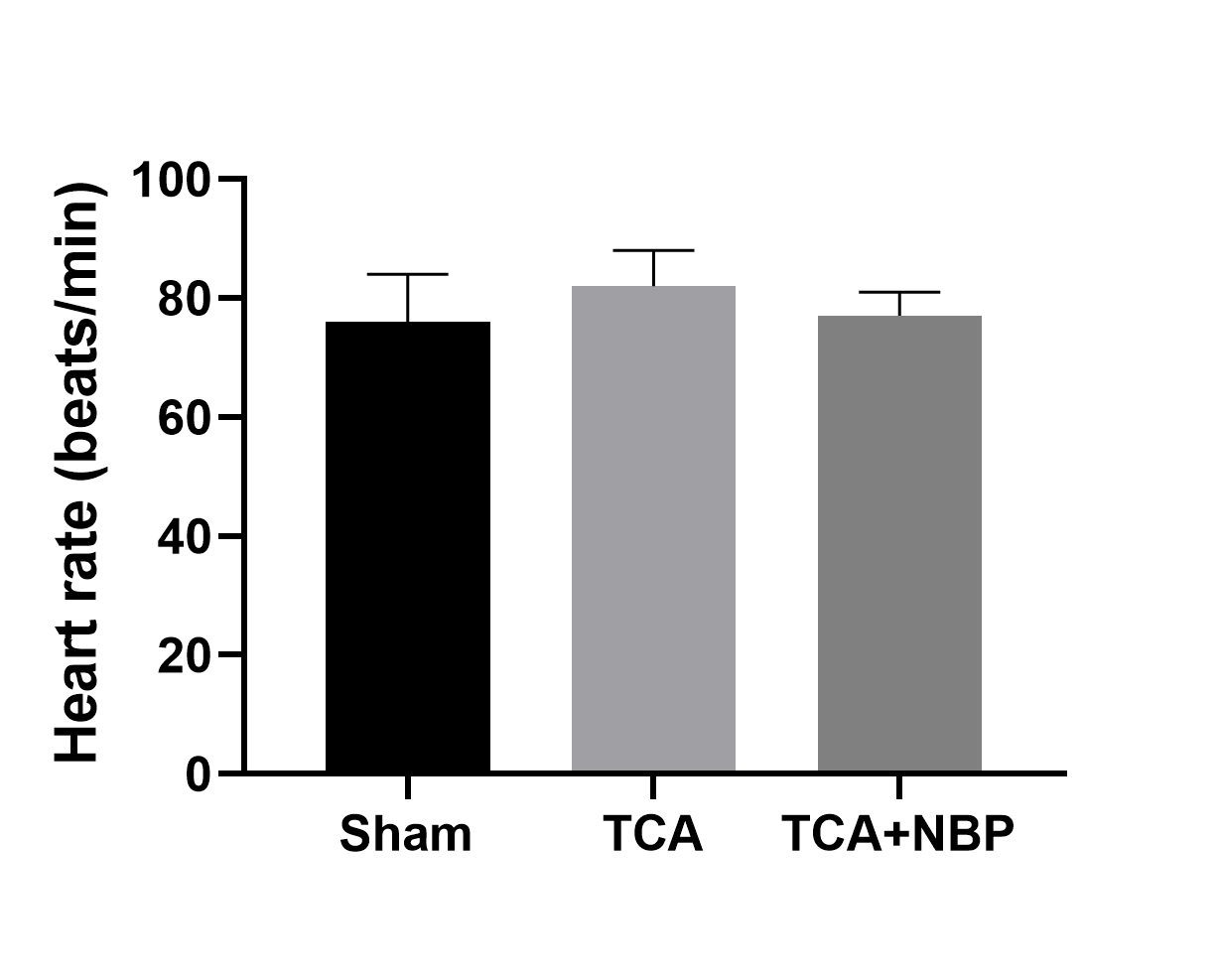


**Figure S1-030. Physiological/biochemical indicator chart; sample or target: HR**


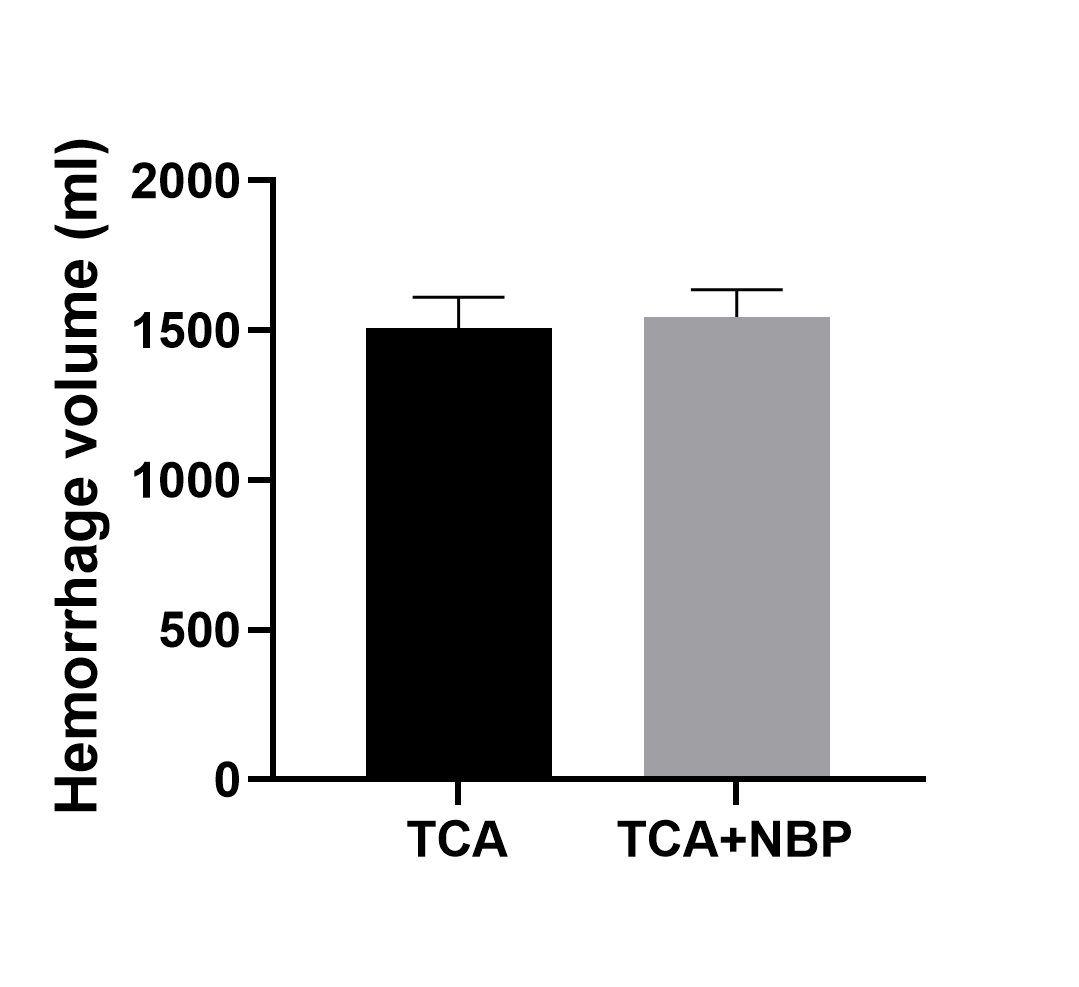


**Figure S1-031. Physiological/biochemical indicator chart; sample or target: HV**


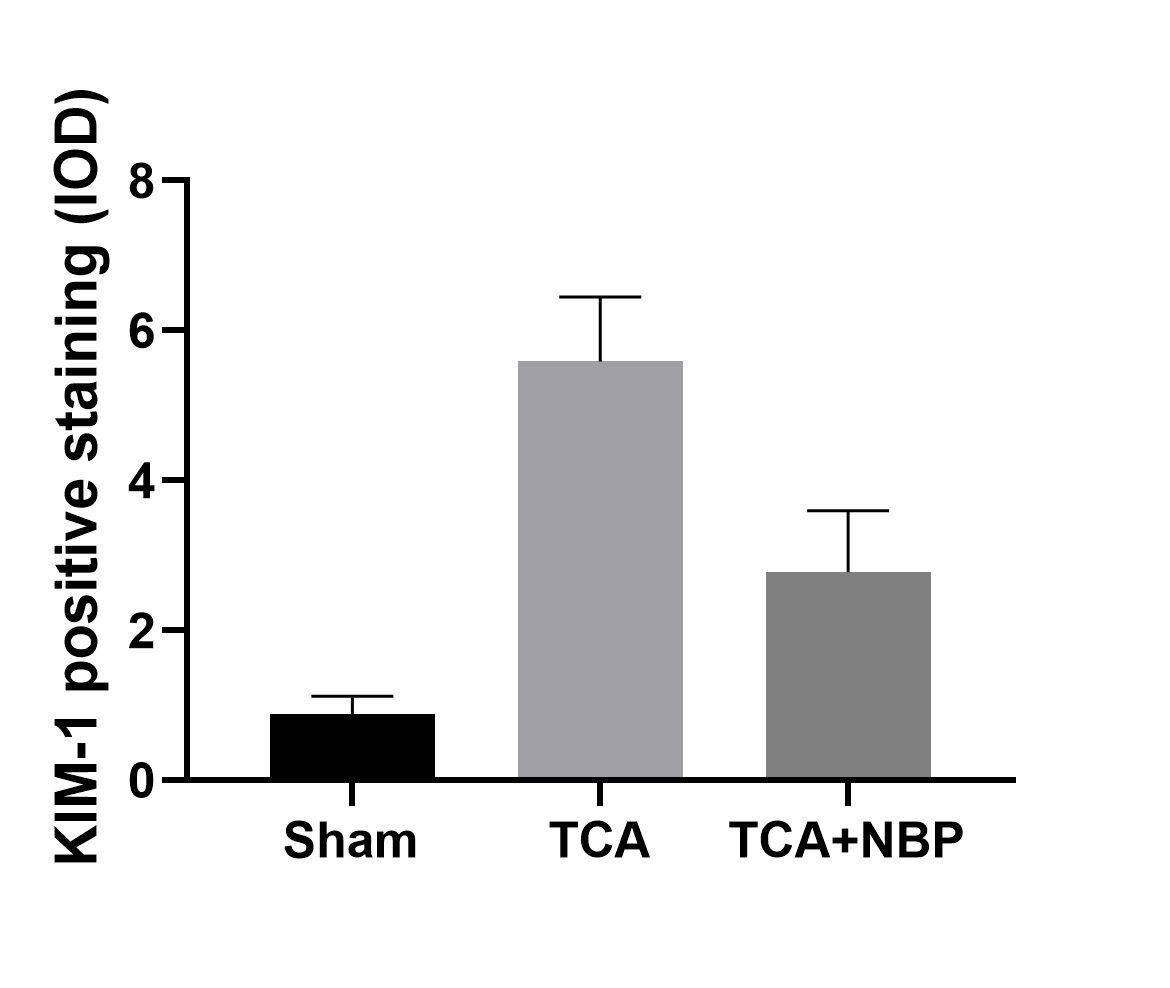


**Figure S1-032. Physiological/biochemical indicator chart; sample or target: KIM-1**


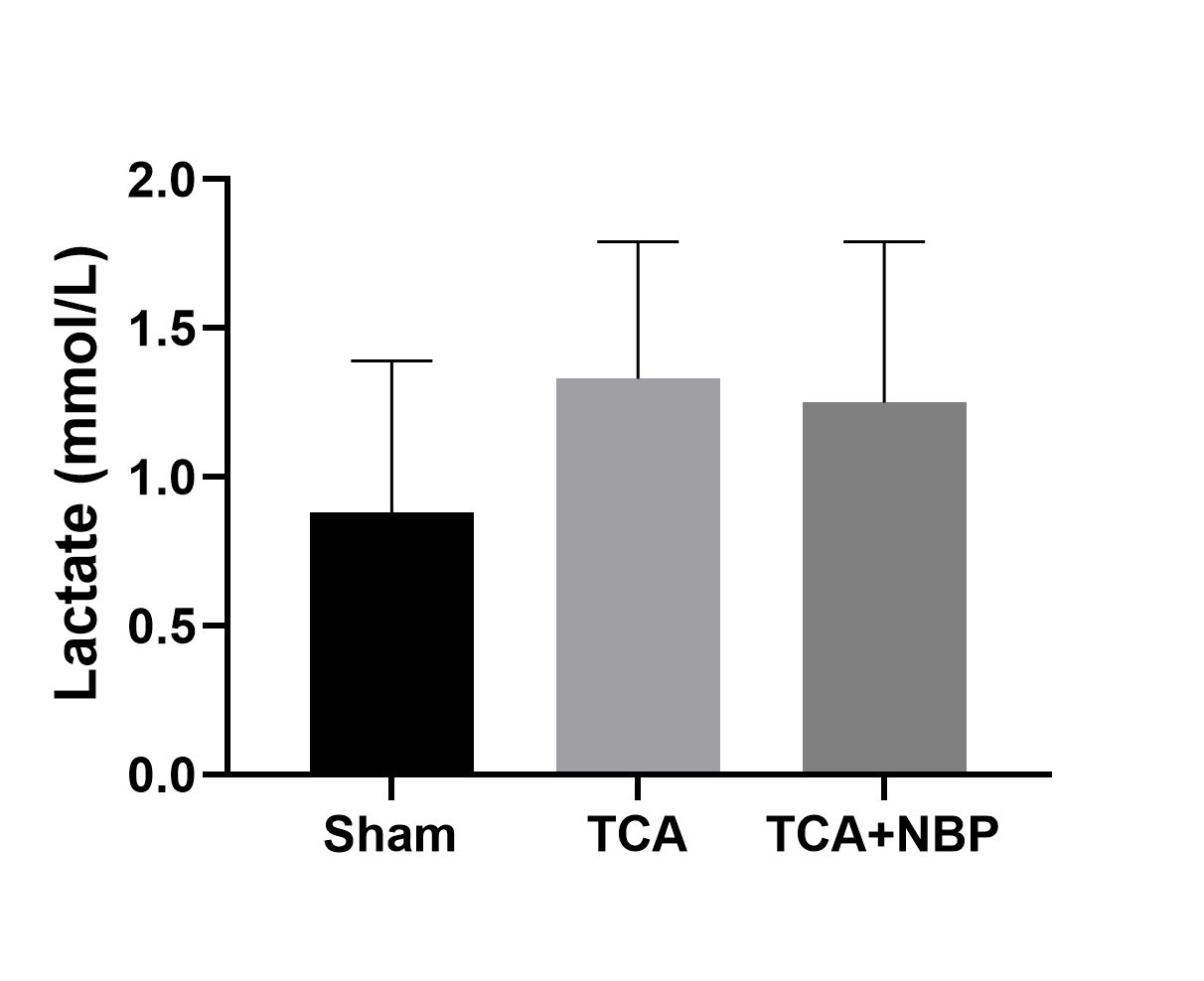


**Figure S1-033. Physiological/biochemical indicator chart; sample or target: LAC**


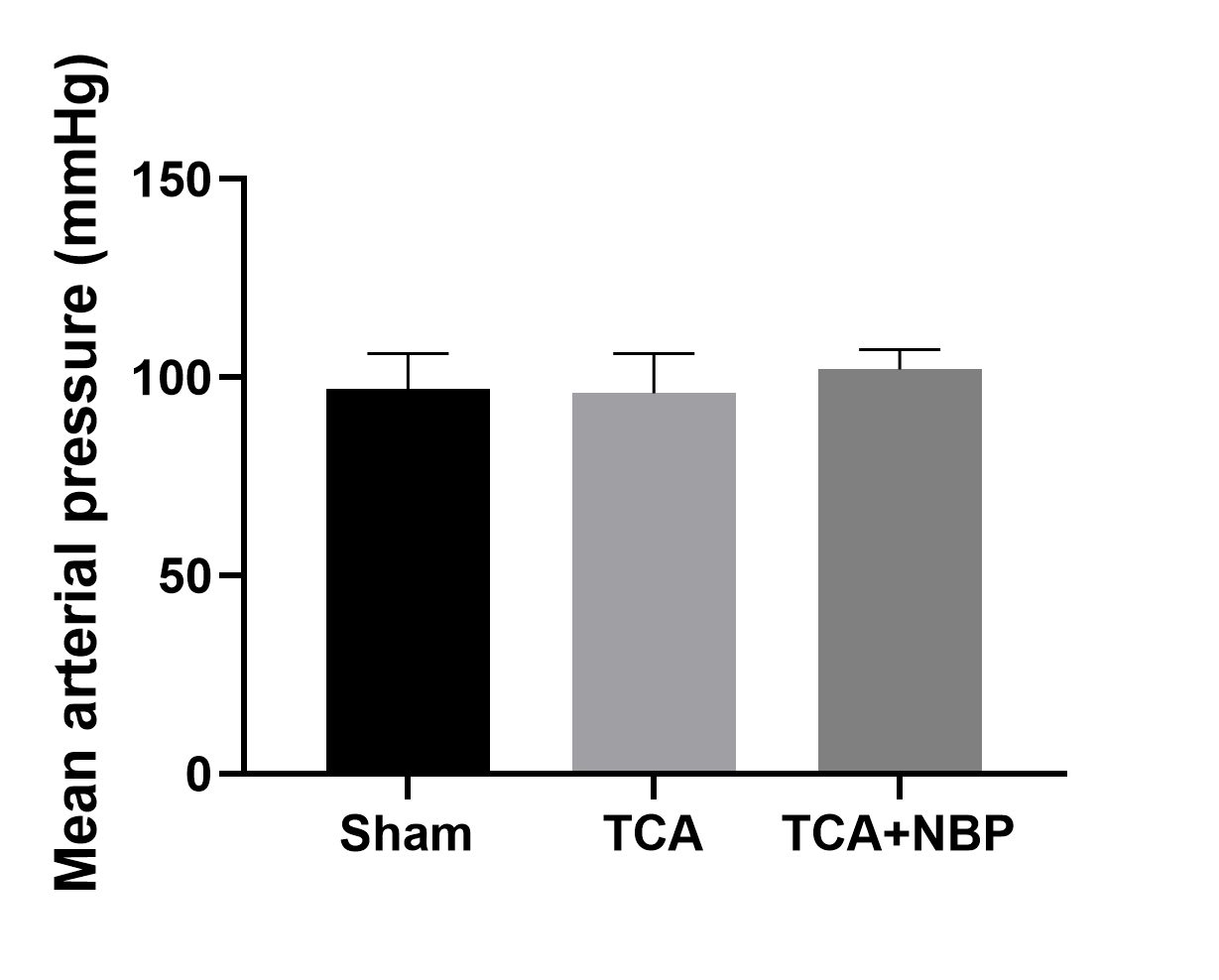


**Figure S1-034. Physiological/biochemical indicator chart; sample or target: MAP**


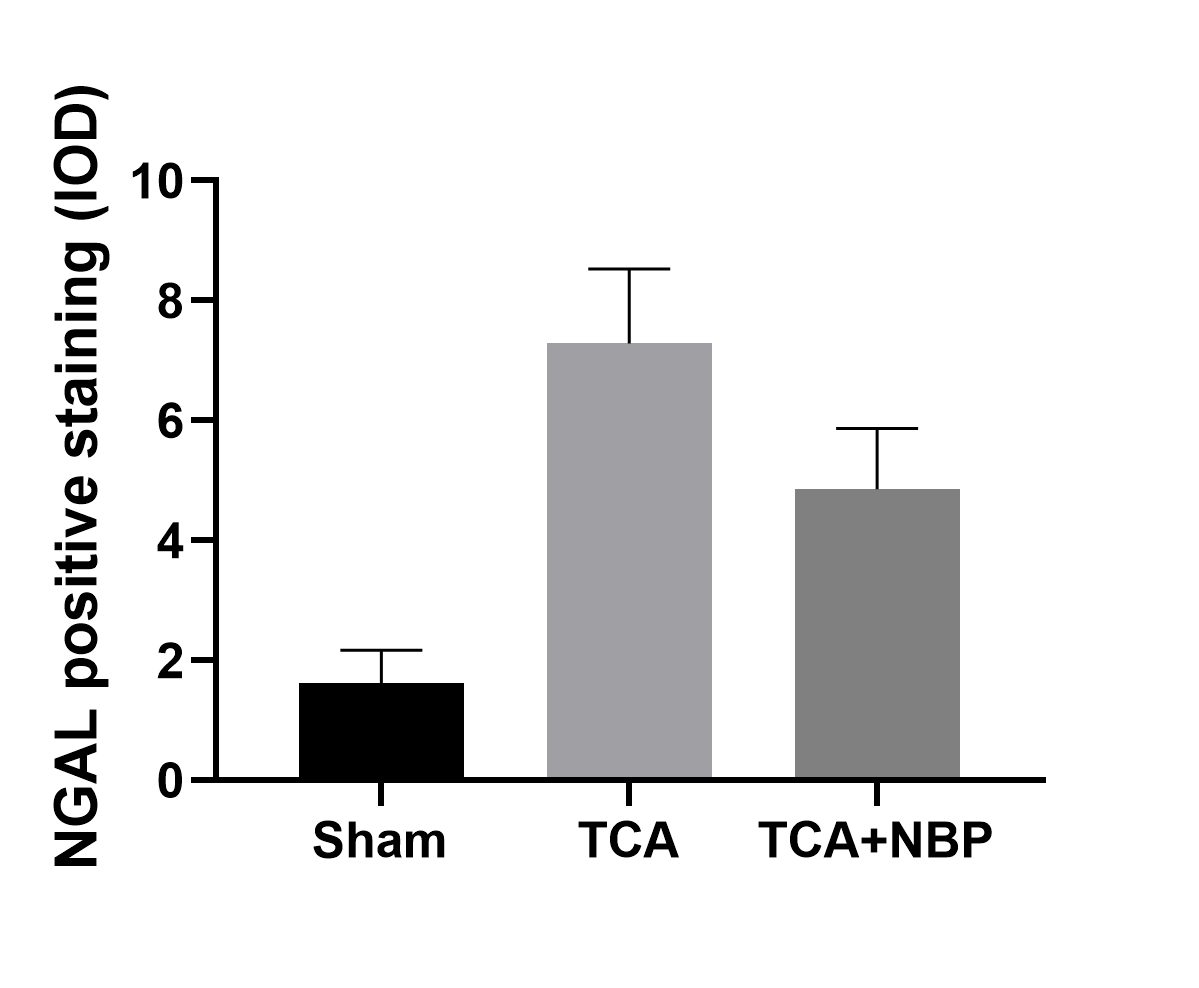


**Figure S1-035. Physiological/biochemical indicator chart; sample or target: NGAL**


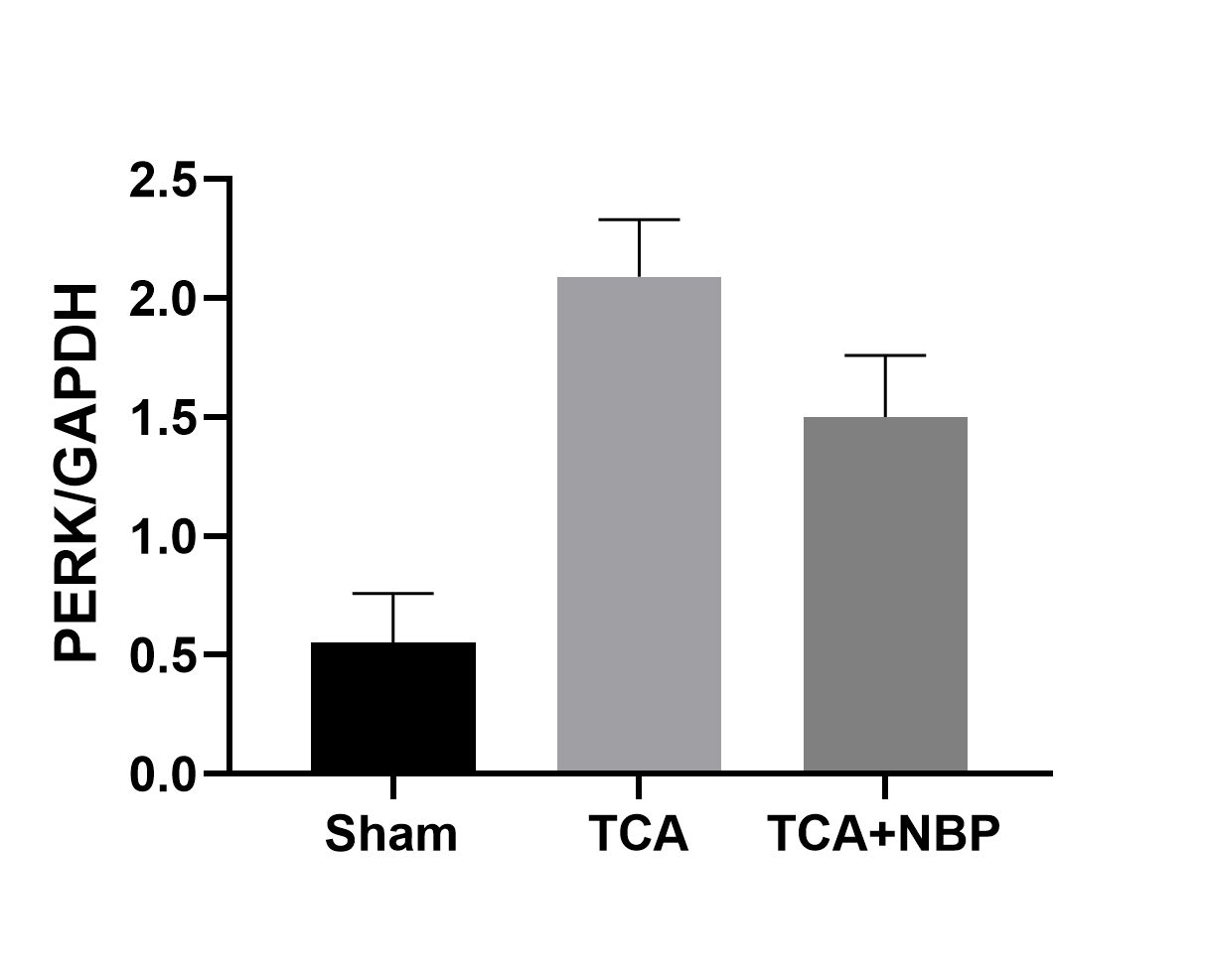


**Figure S1-036. Physiological/biochemical indicator chart; sample or target: PERK**


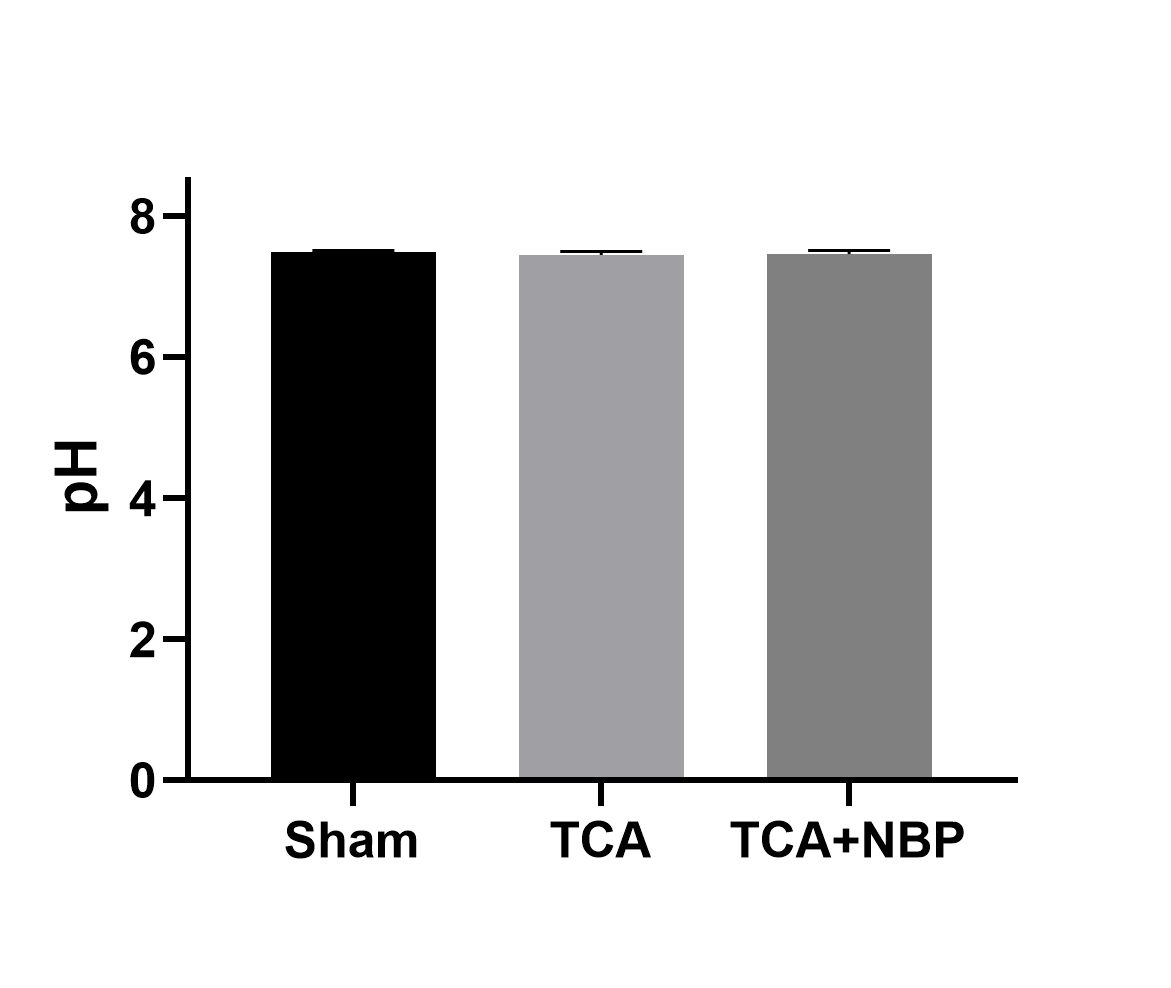


**Figure S1-037. Physiological/biochemical indicator chart; sample or target: pH**


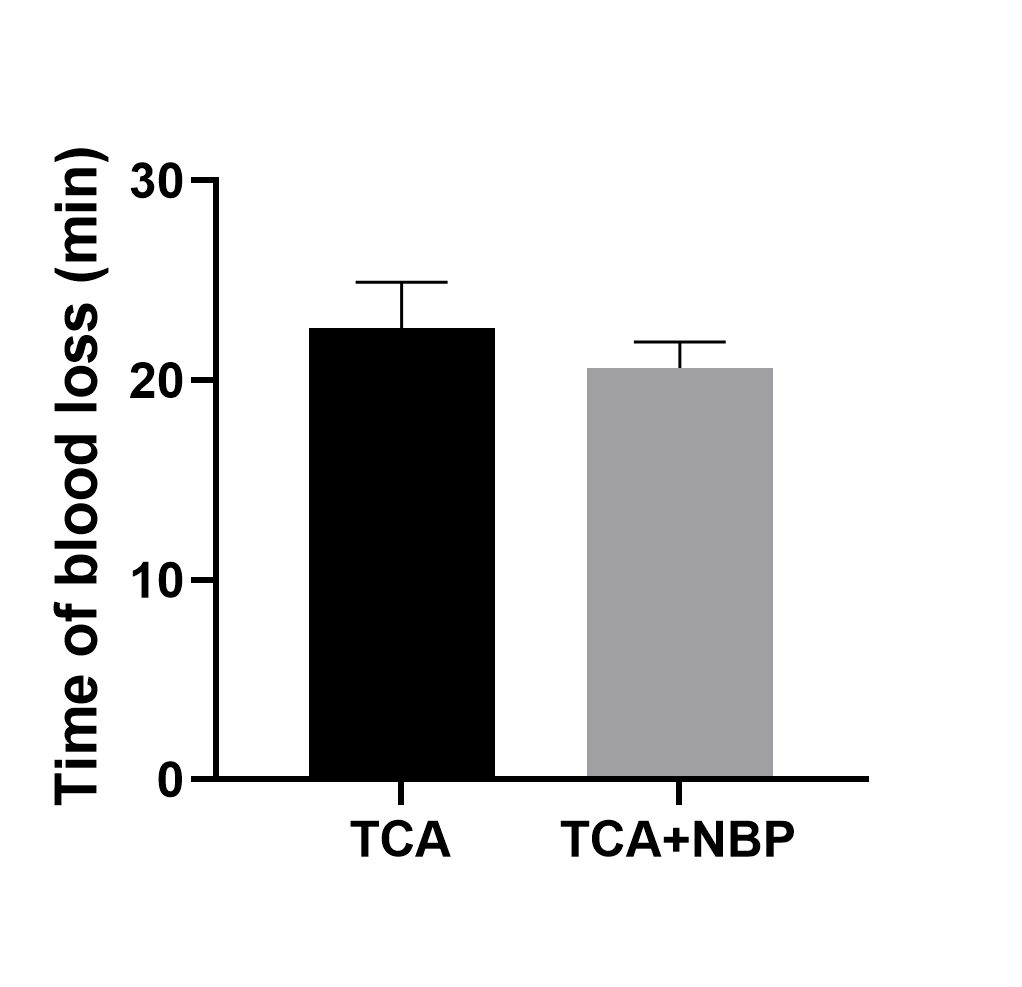


**Figure S1-038. Physiological/biochemical indicator chart; sample or target: TBL**

# Section: H&E staining


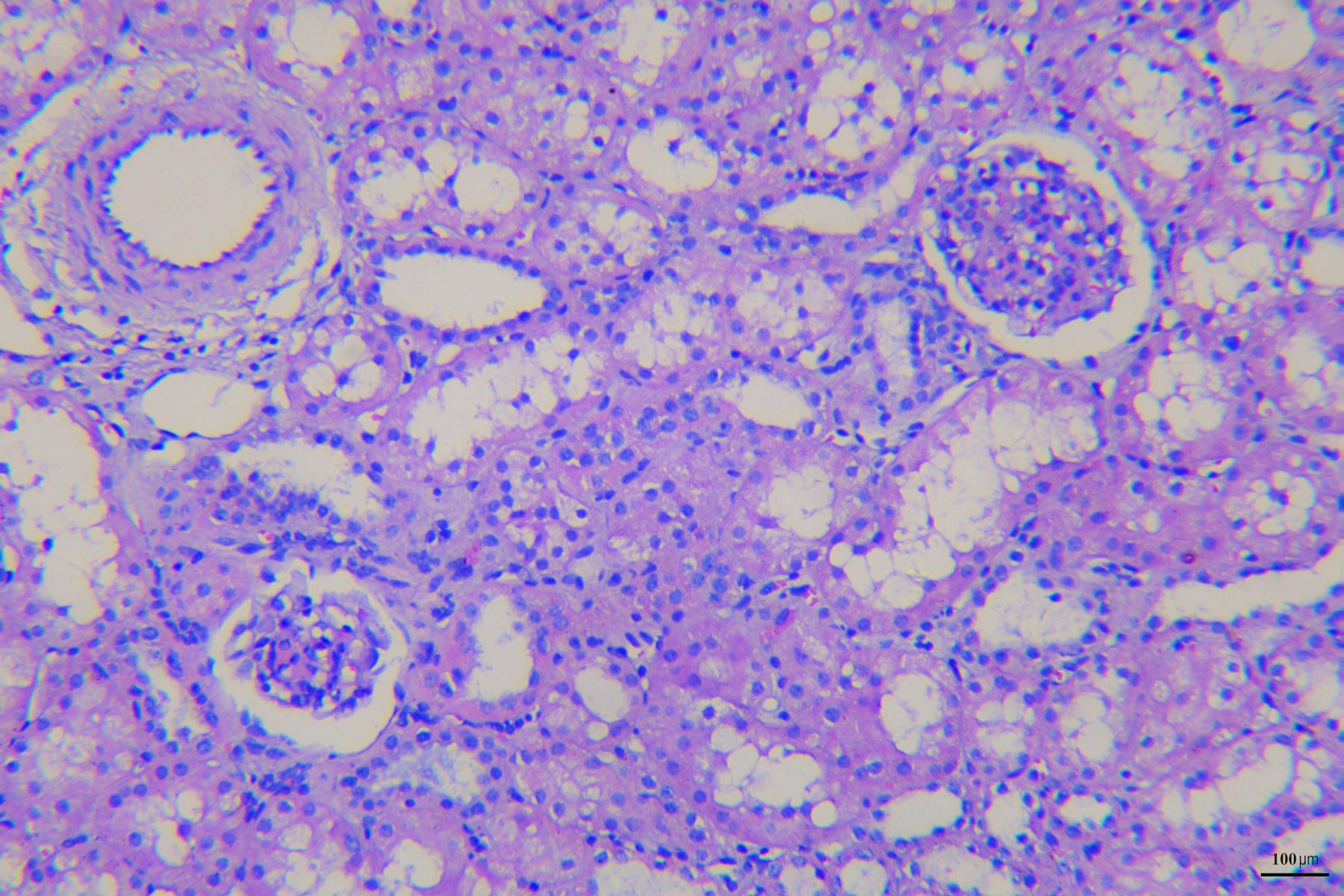


**Figure S1-039. H&E staining (renal histopathology); sample or target: sham-03**


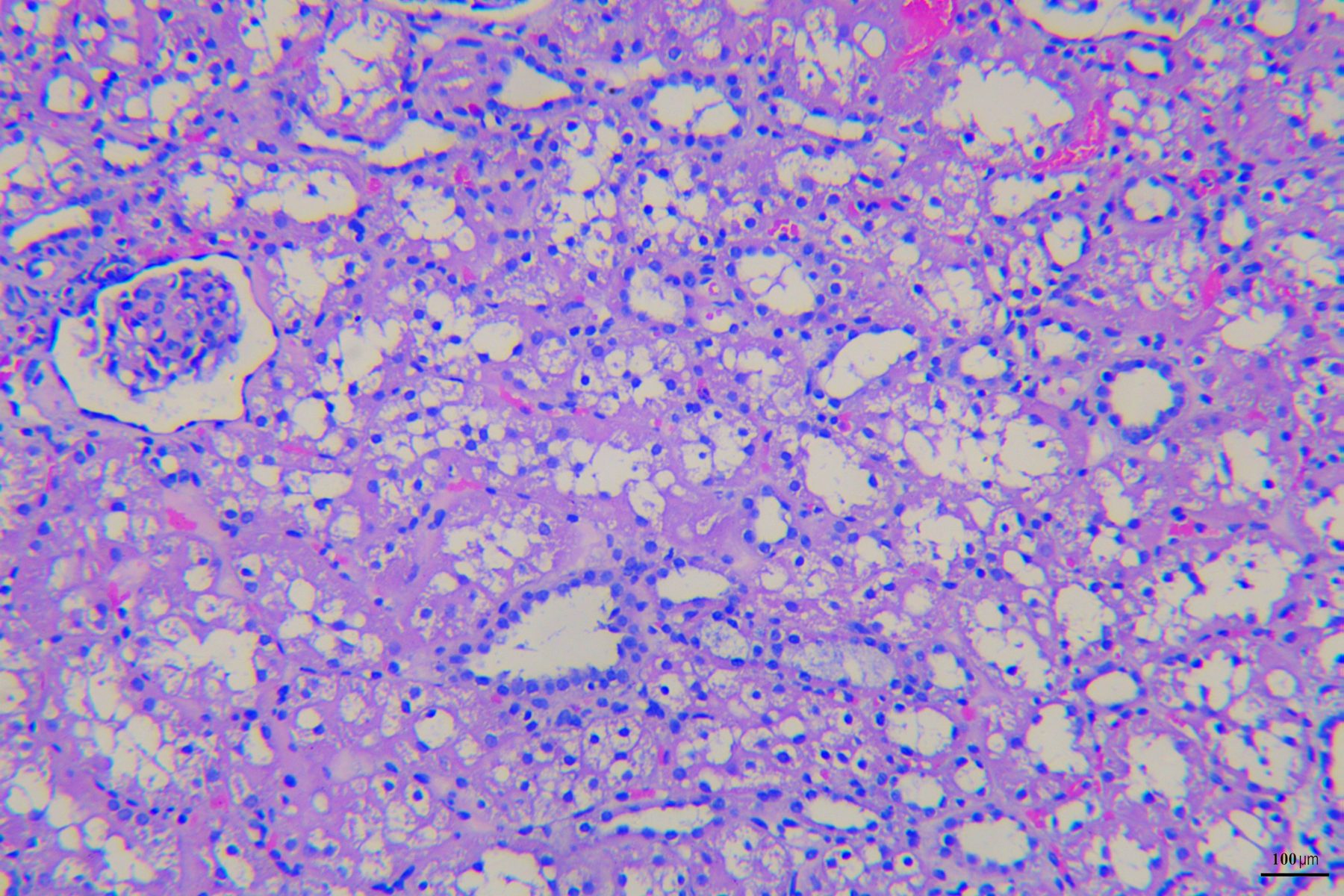


**Figure S1-040. H&E staining (renal histopathology); sample or target: sham-09**


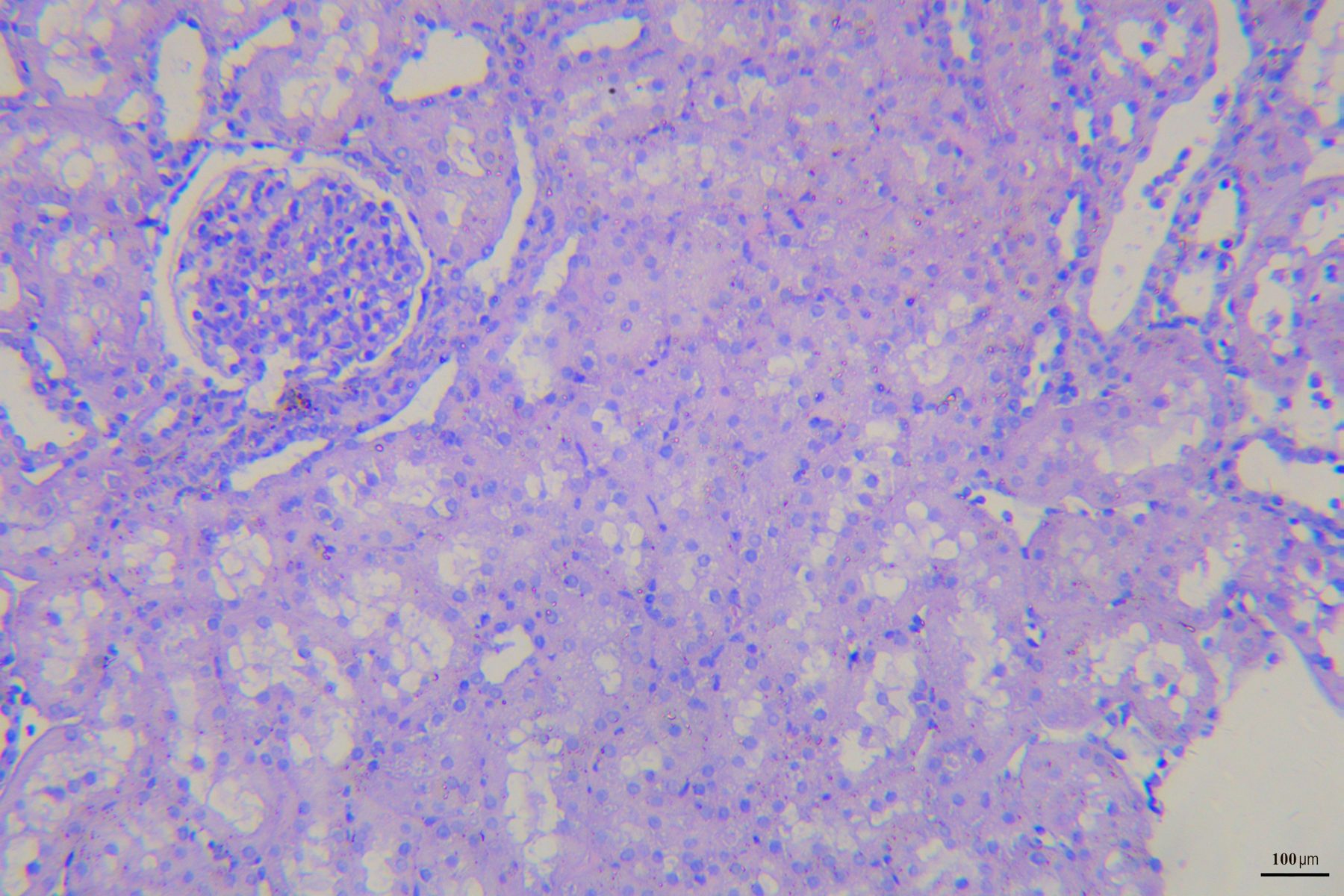


**Figure S1-041. H&E staining (renal histopathology); sample or target: sham-13**


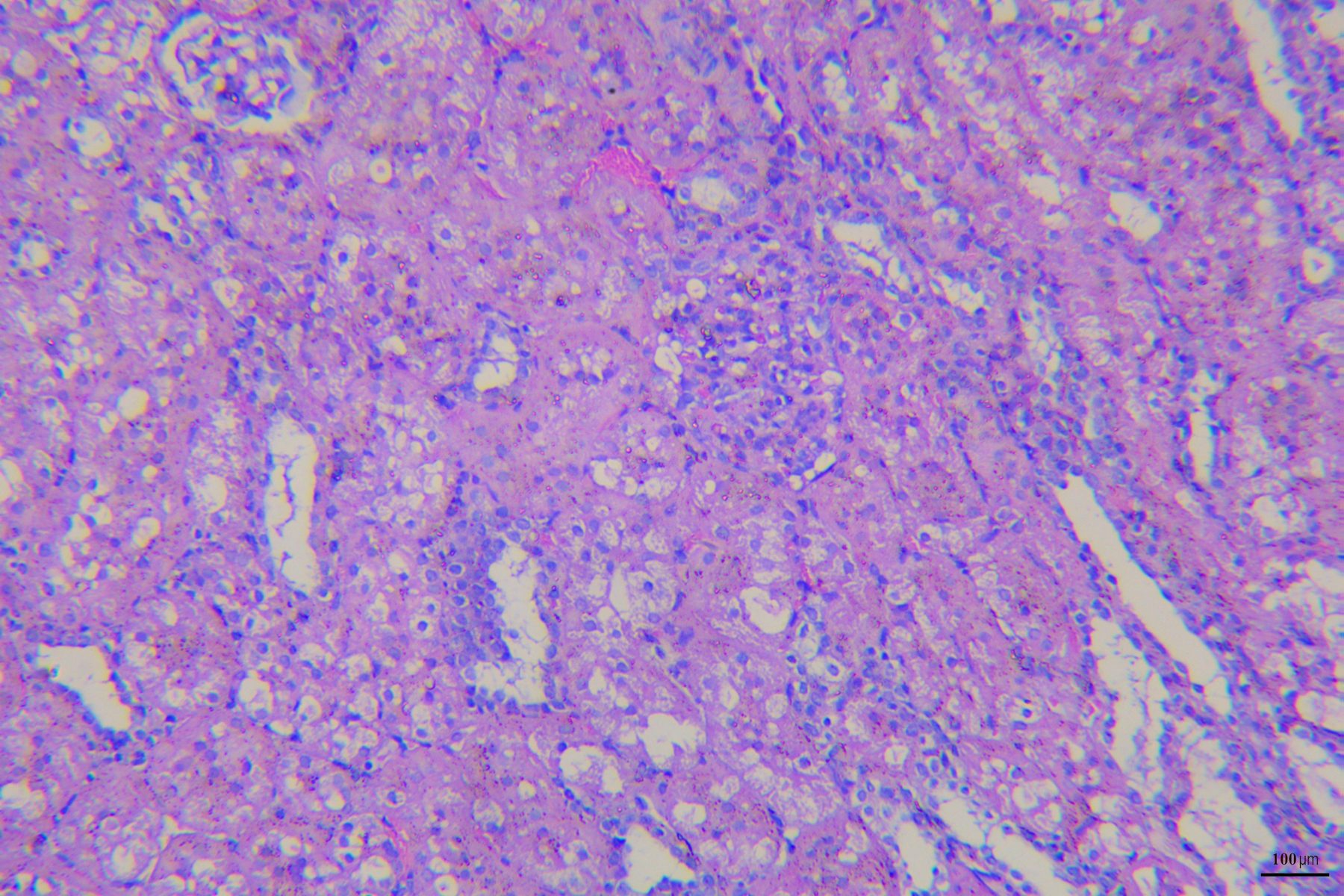


**Figure S1-042. H&E staining (renal histopathology); sample or target: sham-16**


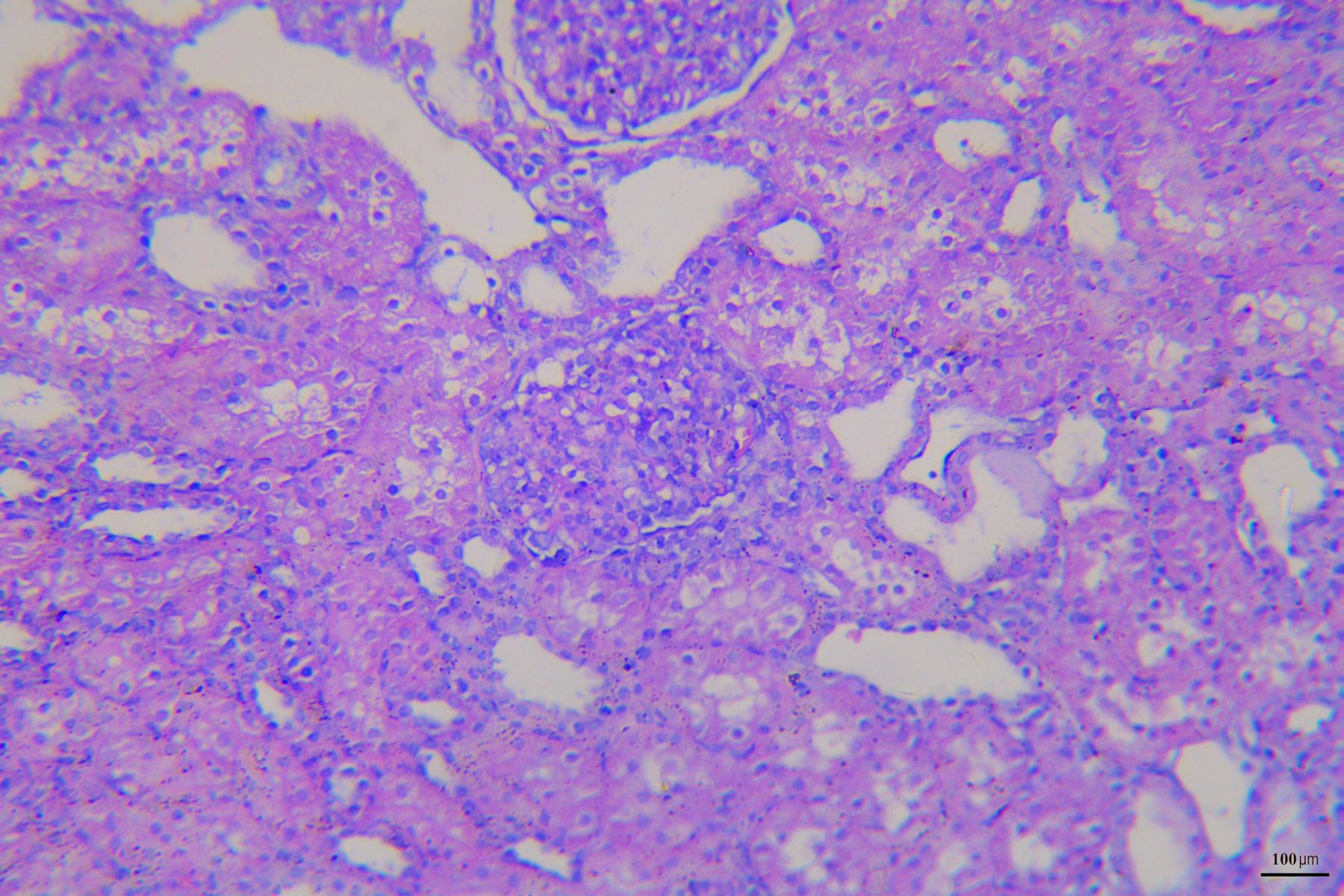


**Figure S1-043. H&E staining (renal histopathology); sample or target: sham-23**


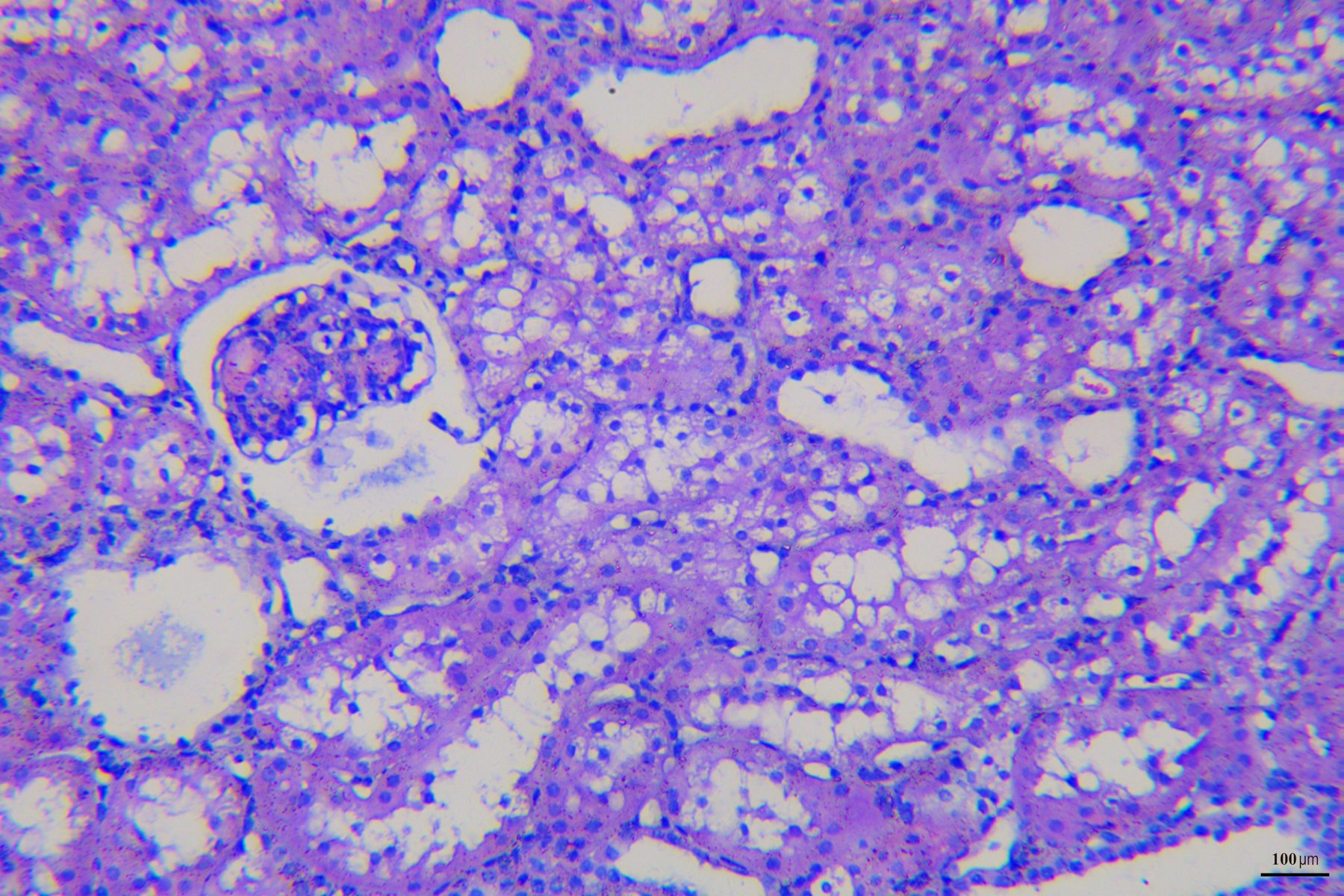


**Figure S1-044. H&E staining (renal histopathology); sample or target: TCA+NBP-05**


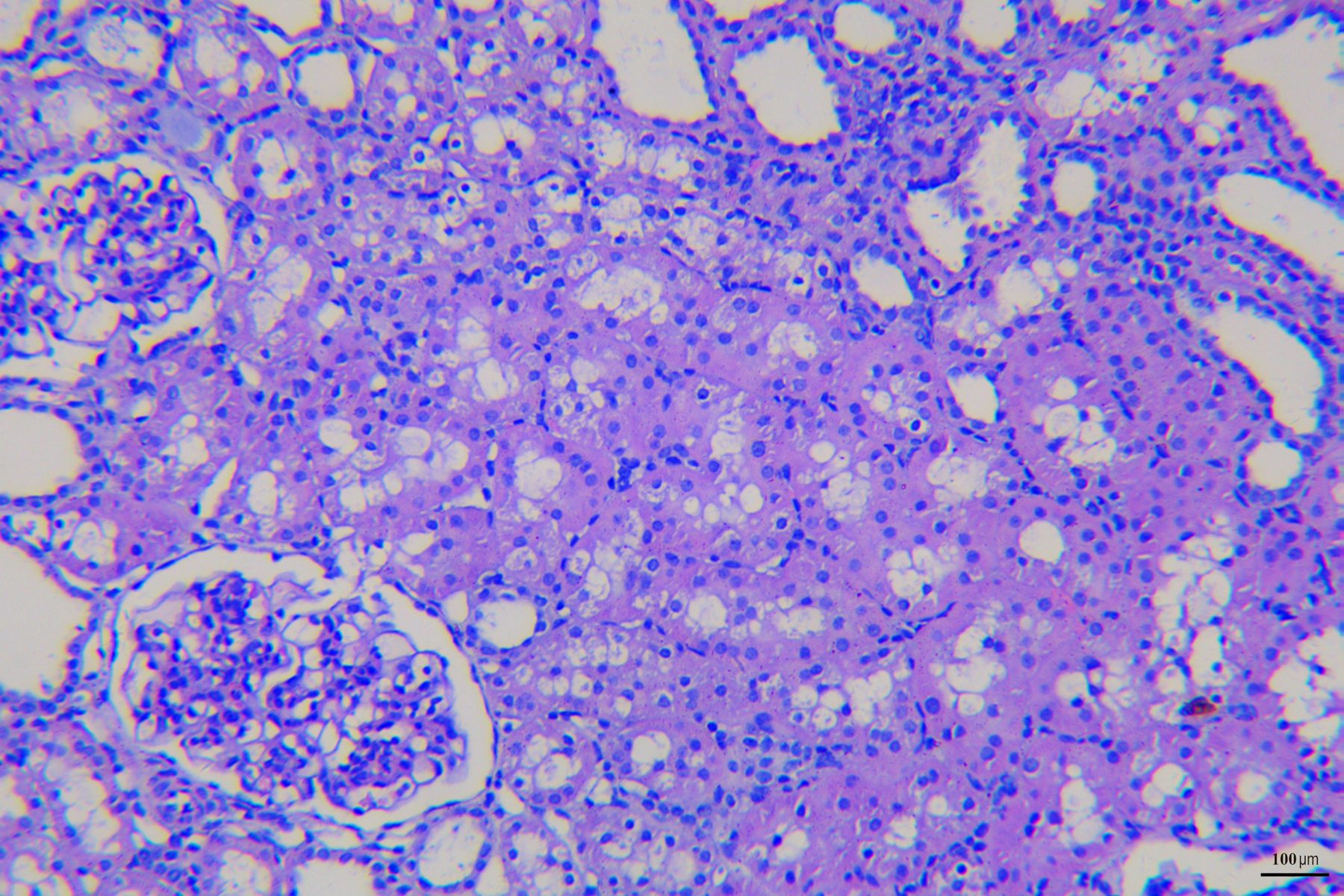


**Figure S1-045. H&E staining (renal histopathology); sample or target: TCA+NBP-07**


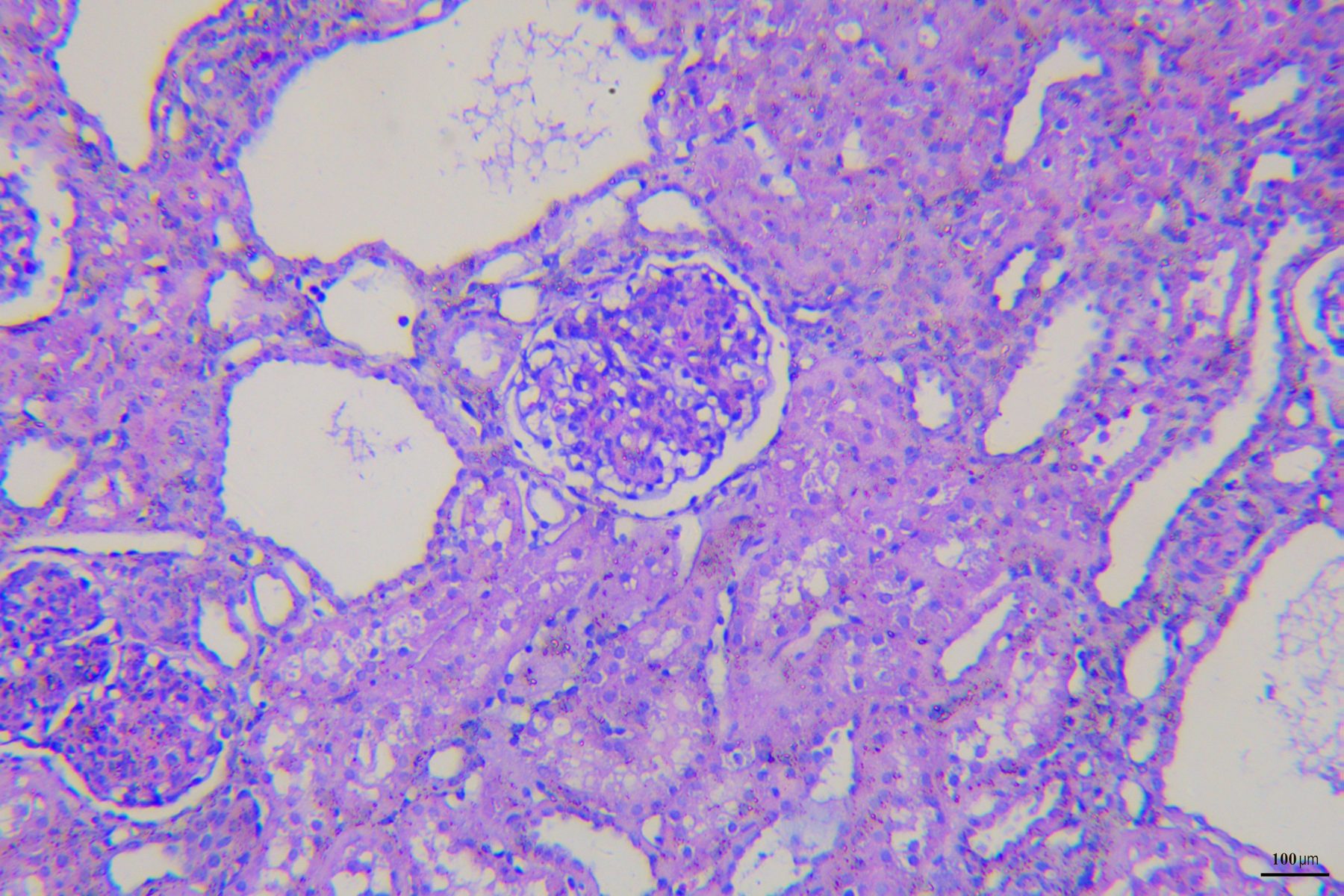


**Figure S1-046. H&E staining (renal histopathology); sample or target: TCA+NBP-17**


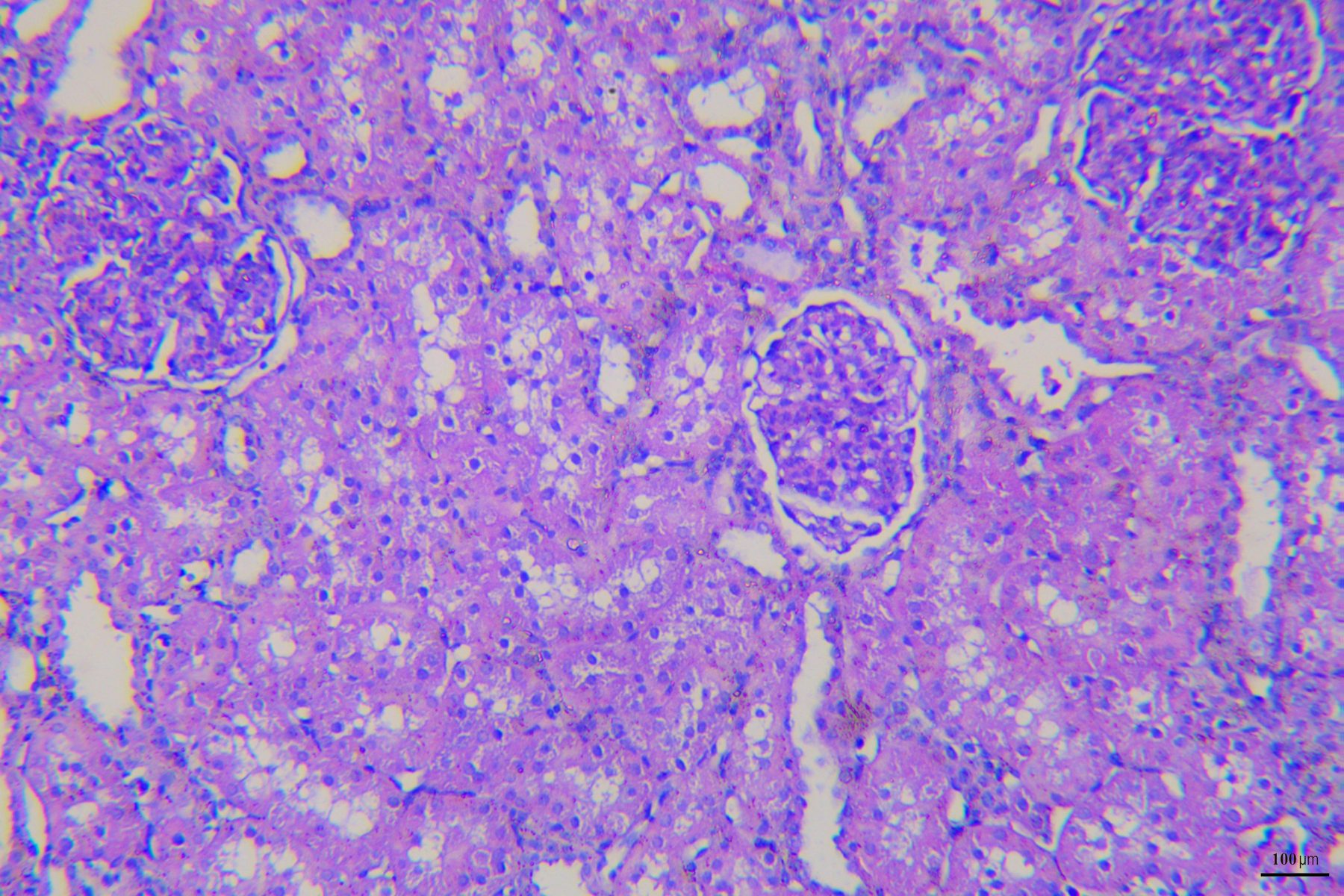


**Figure S1-047. H&E staining (renal histopathology); sample or target: TCA+NBP-20**


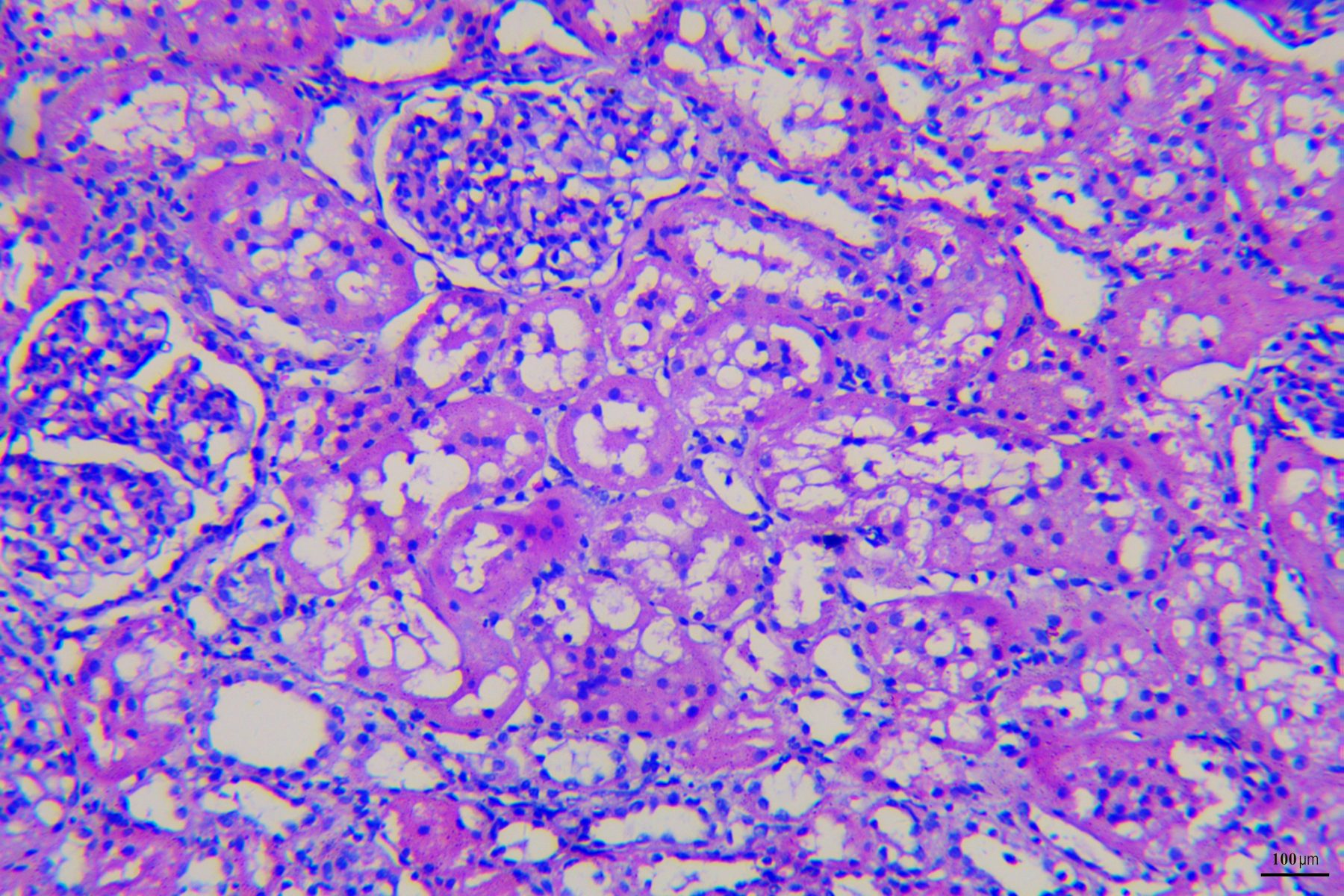


**Figure S1-048. H&E staining (renal histopathology); sample or target: TCA+NBP-21**


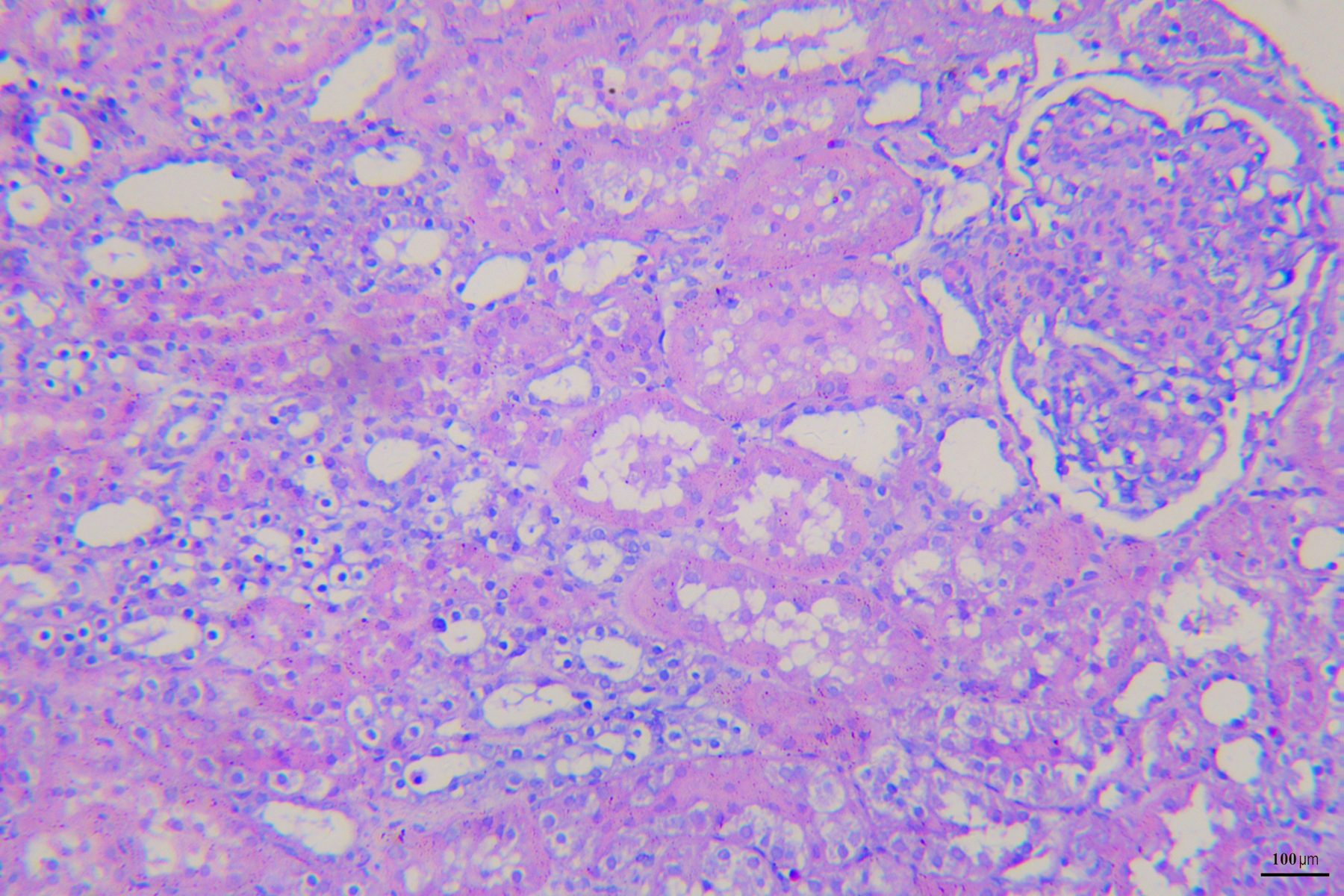


**Figure S1-049. H&E staining (renal histopathology); sample or target: TCA+NBP-22**


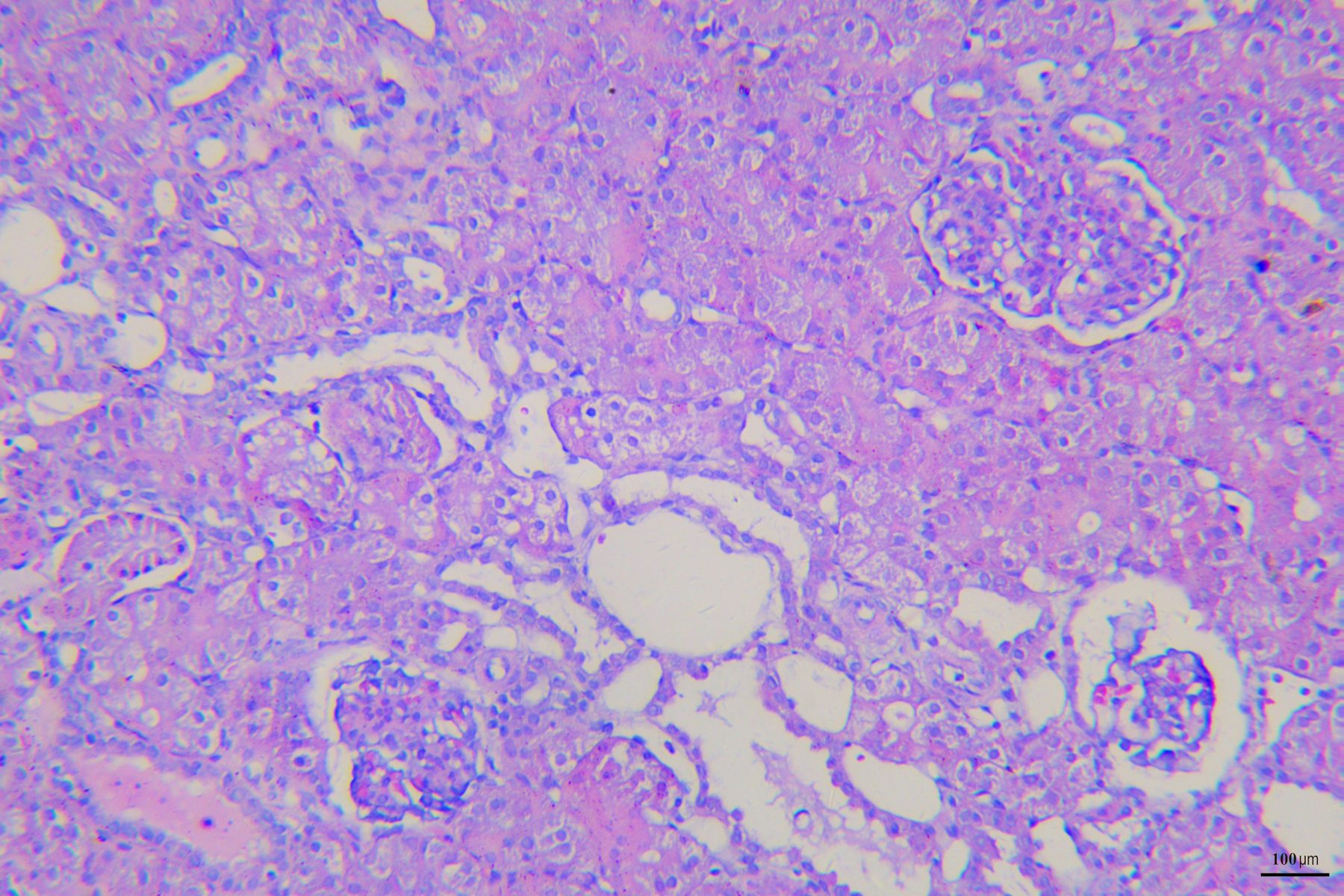


**Figure S1-050. H&E staining (renal histopathology); sample or target: TCA+NBP-26**


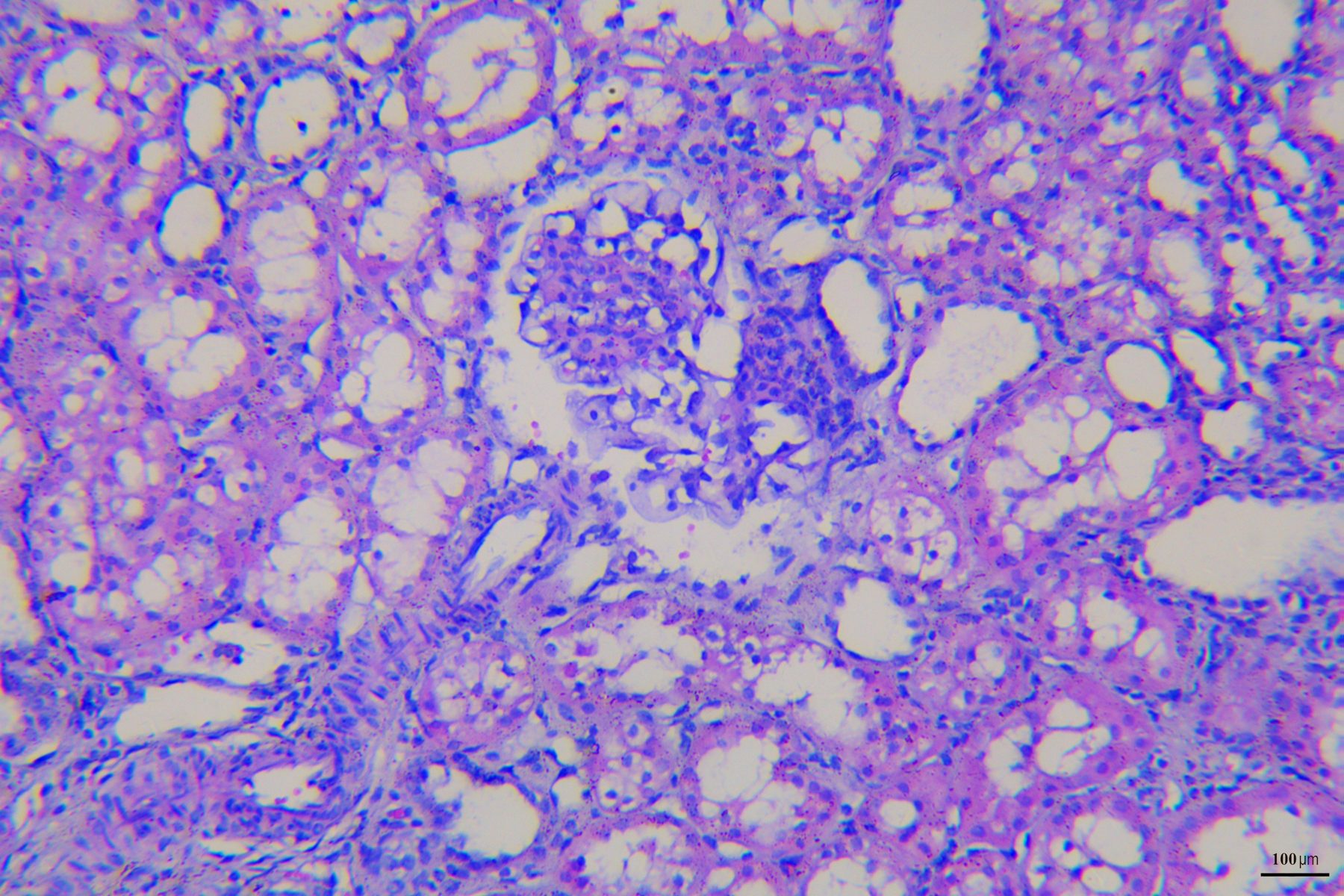


**Figure S1-051. H&E staining (renal histopathology); sample or target: TCA-01**


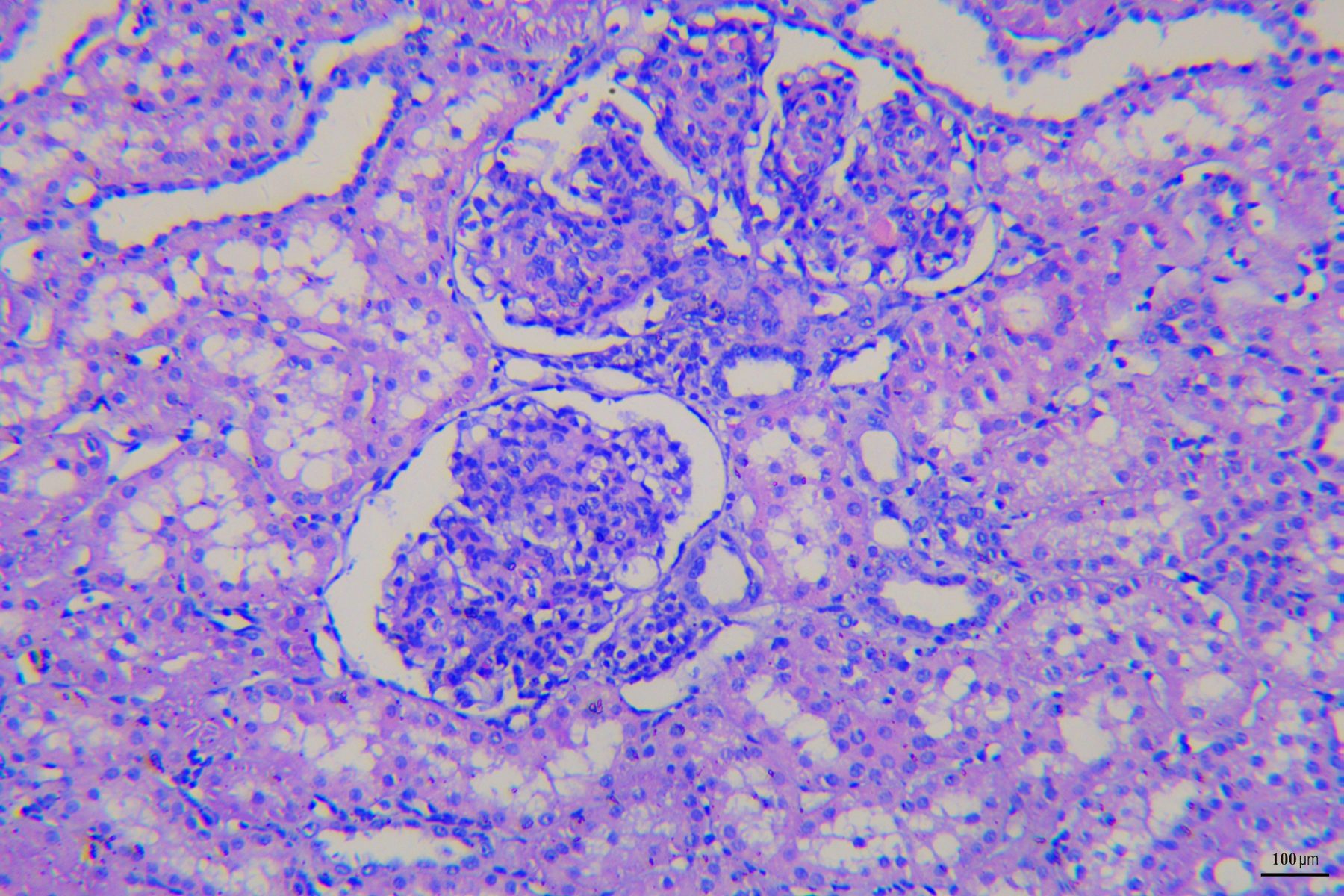


**Figure S1-052. H&E staining (renal histopathology); sample or target: TCA-02**


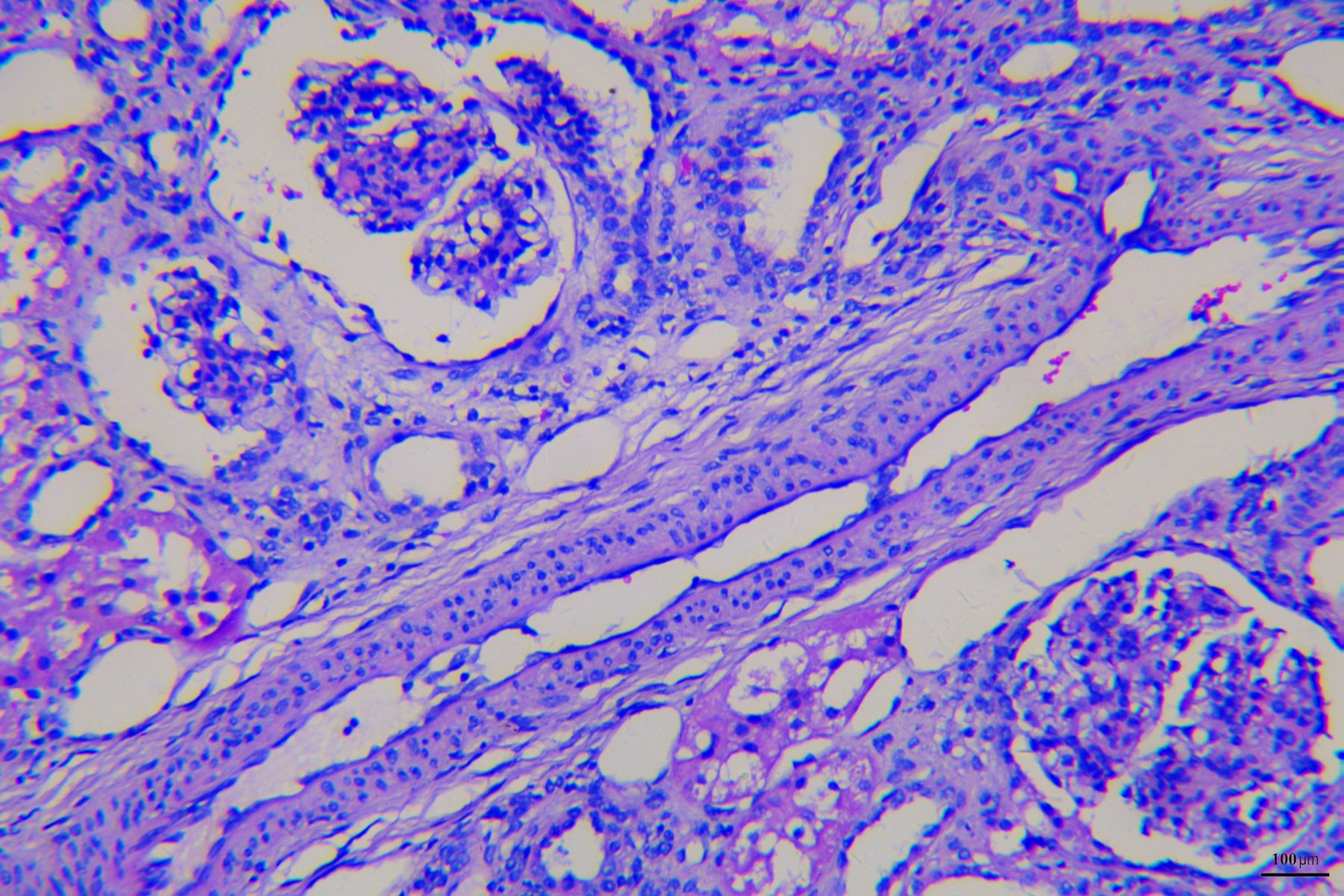


**Figure S1-053. H&E staining (renal histopathology); sample or target: TCA-04**


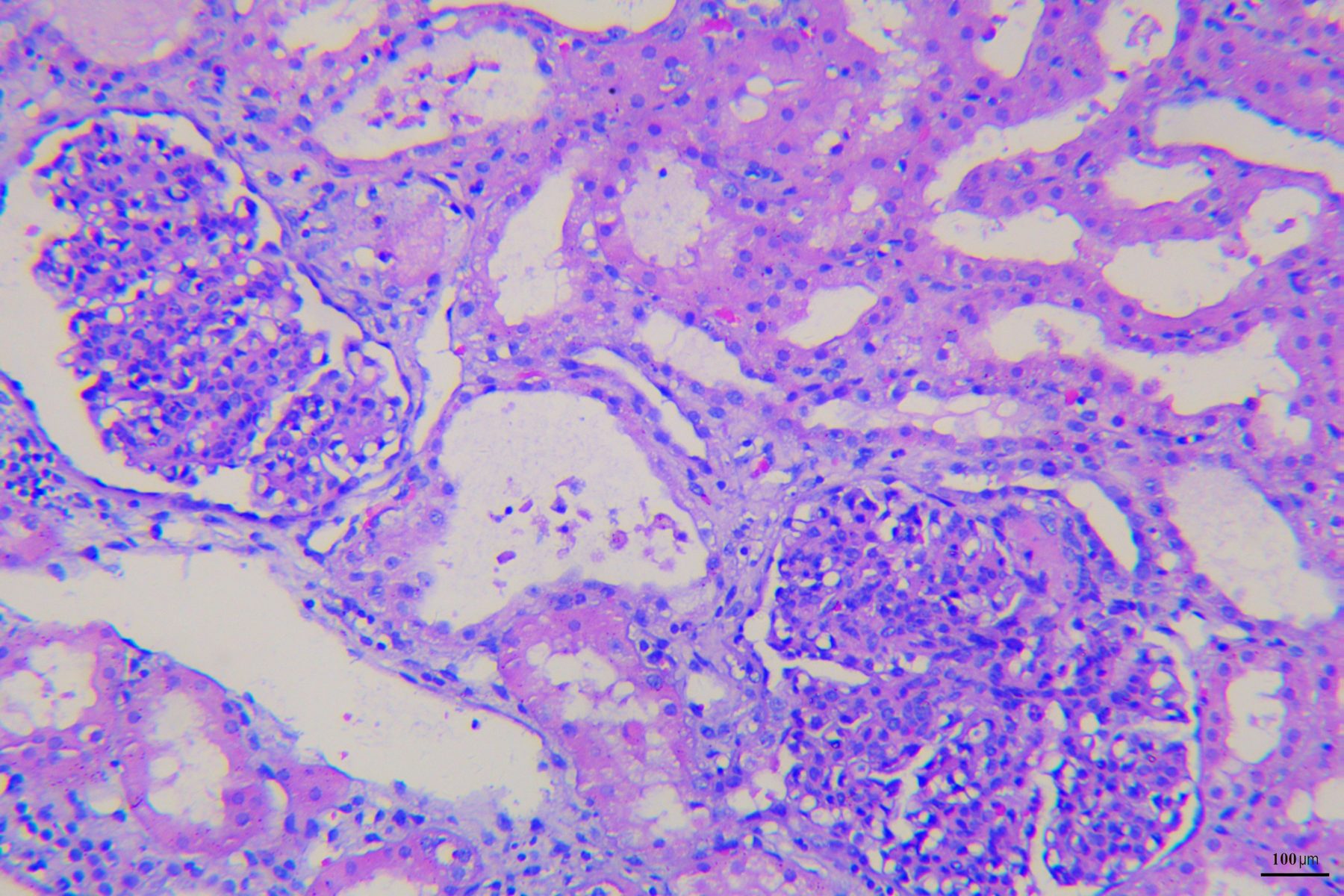


**Figure S1-054. H&E staining (renal histopathology); sample or target: TCA-10**


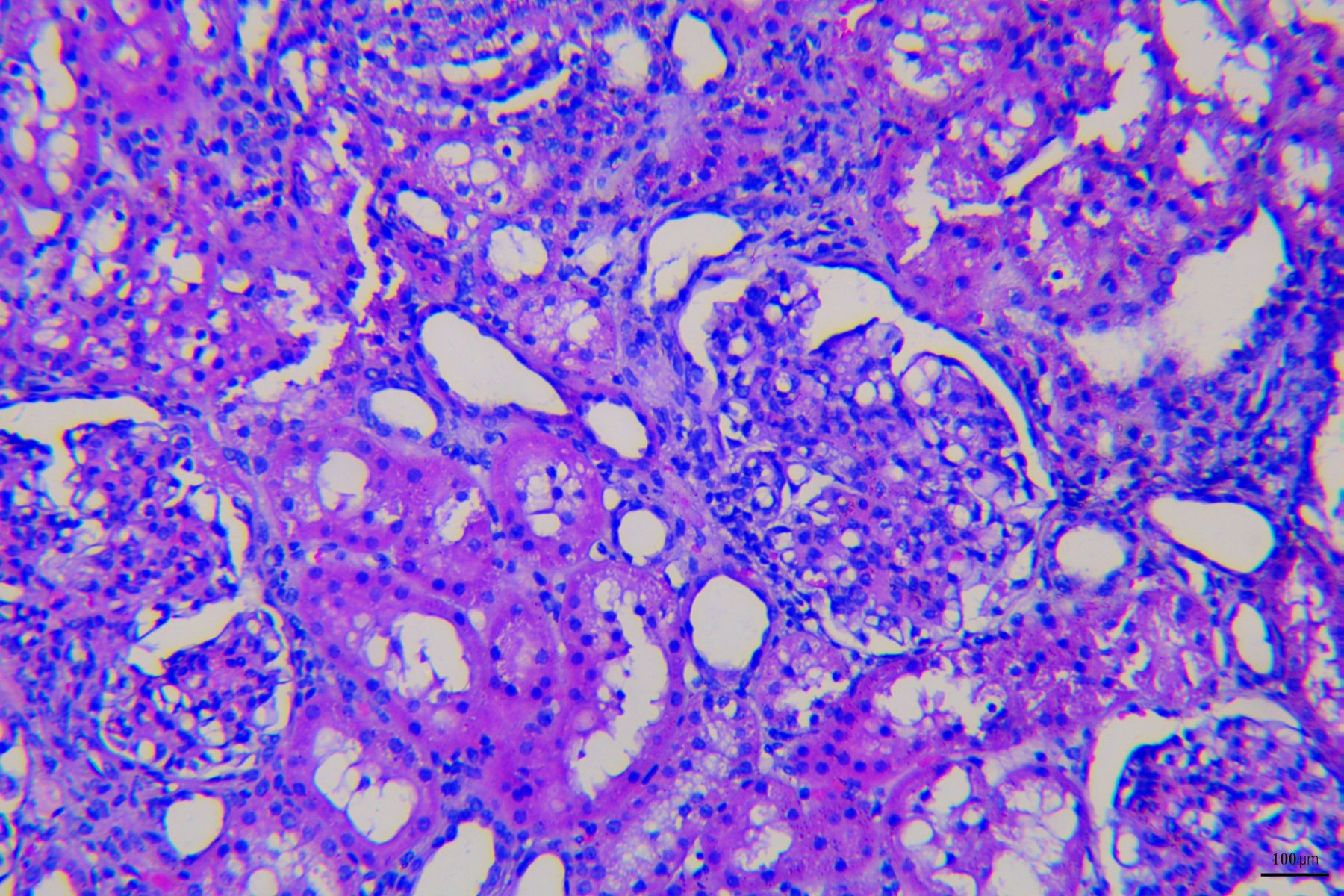


**Figure S1-055. H&E staining (renal histopathology); sample or target: TCA-11**

# Section: KIM-1 immunohistochemistry


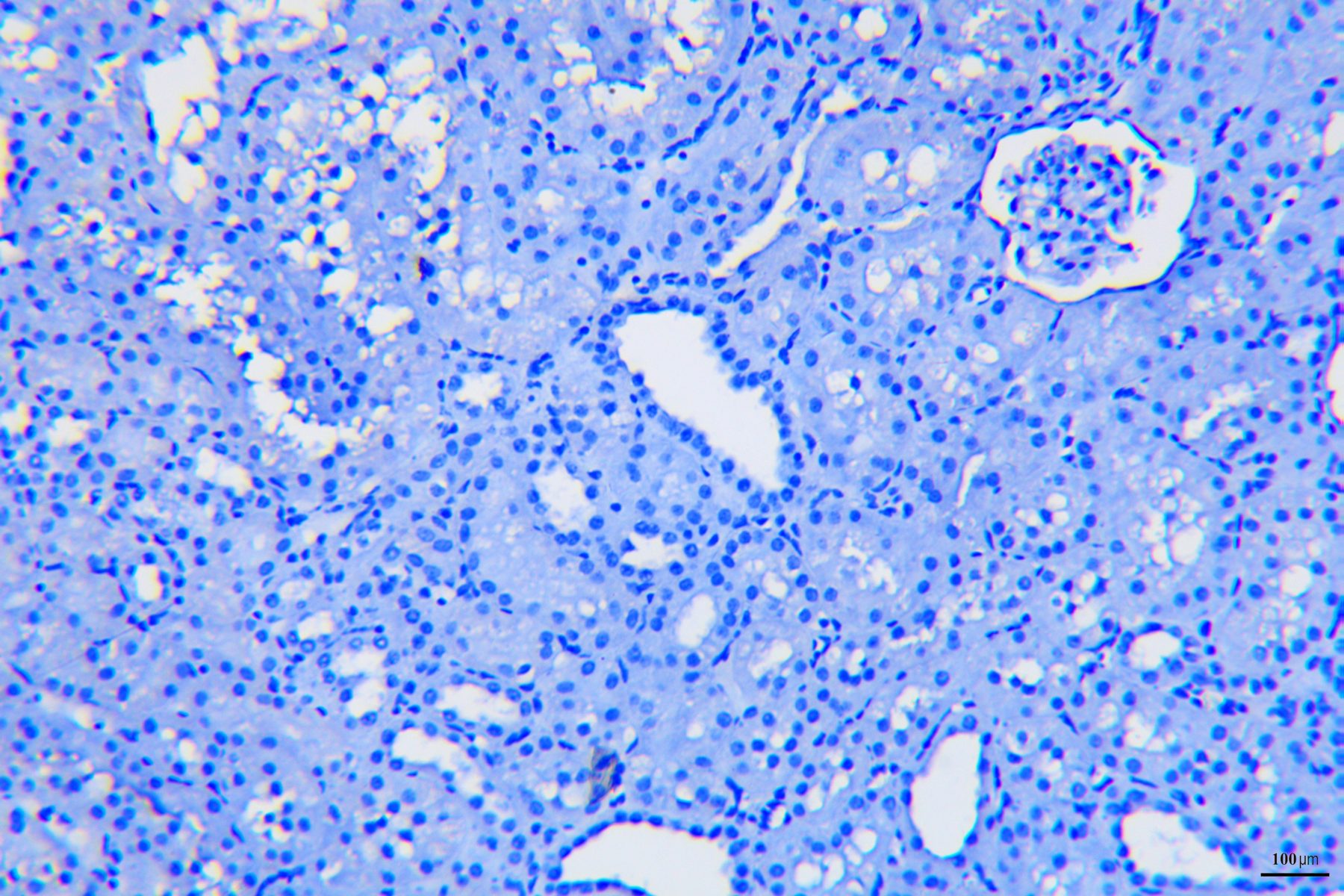


**Figure S1-056. KIM-1 immunohistochemistry; sample or target: sham-03**


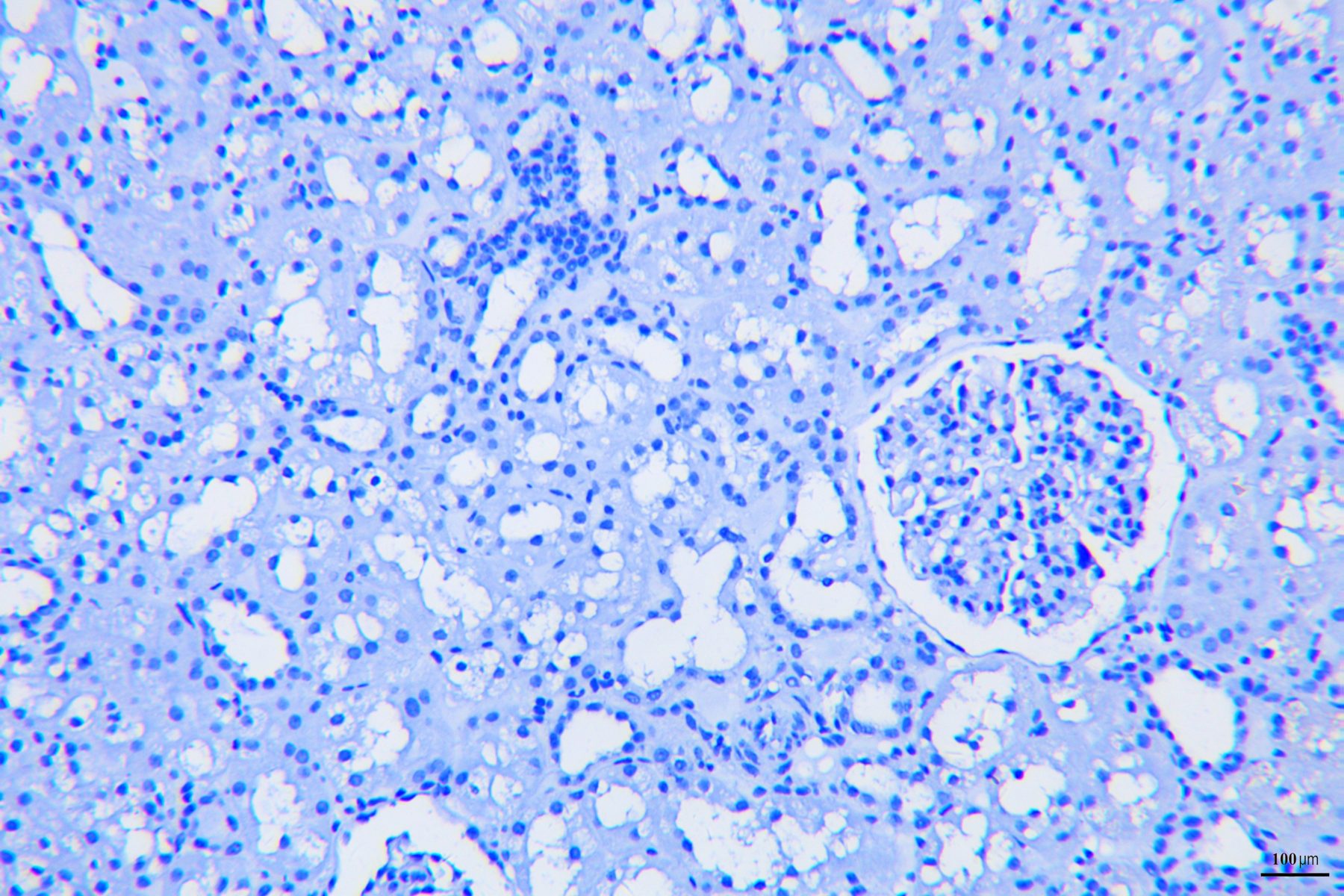


**Figure S1-057. KIM-1 immunohistochemistry; sample or target: sham-09**


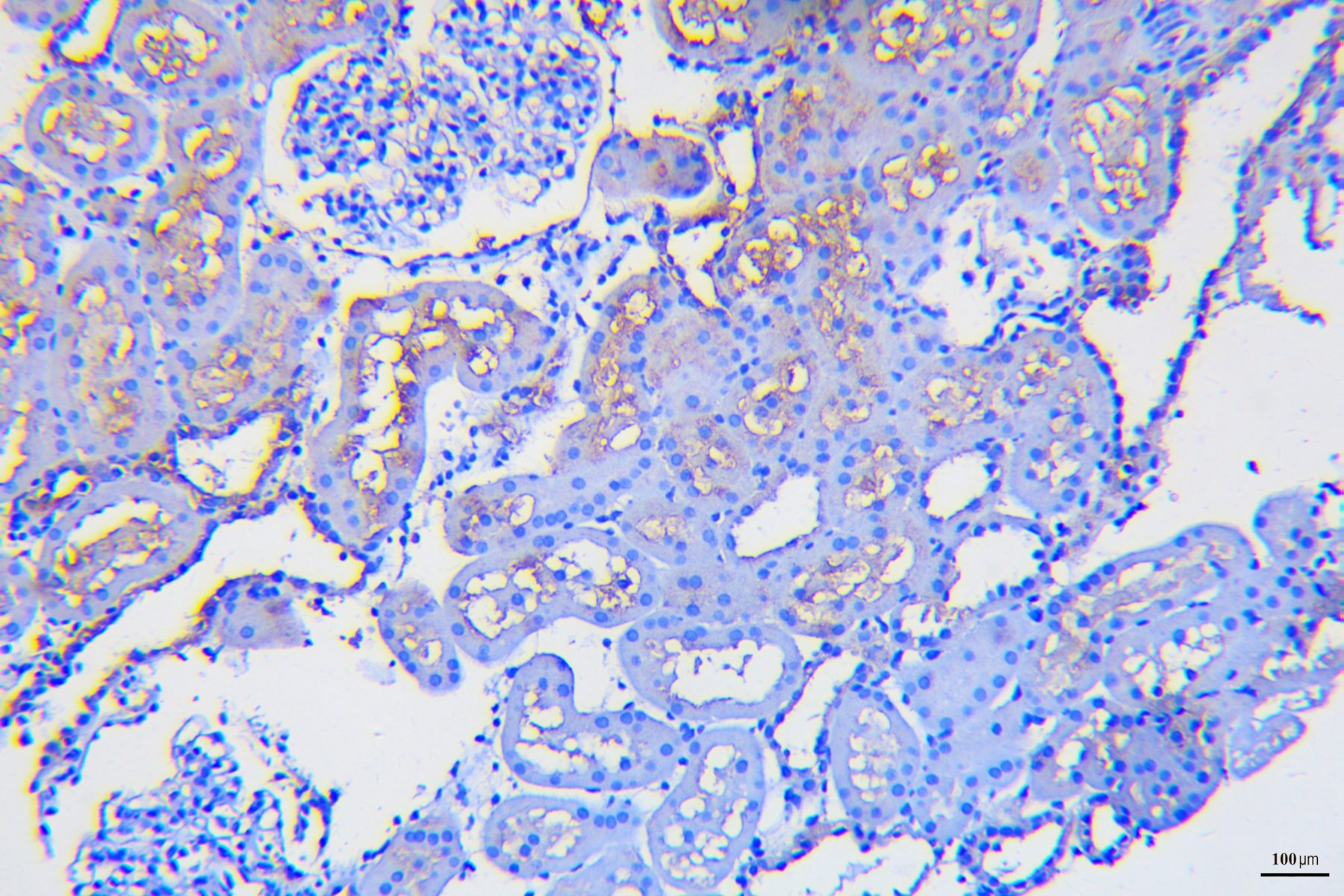


**Figure S1-058. KIM-1 immunohistochemistry; sample or target: sham-13**


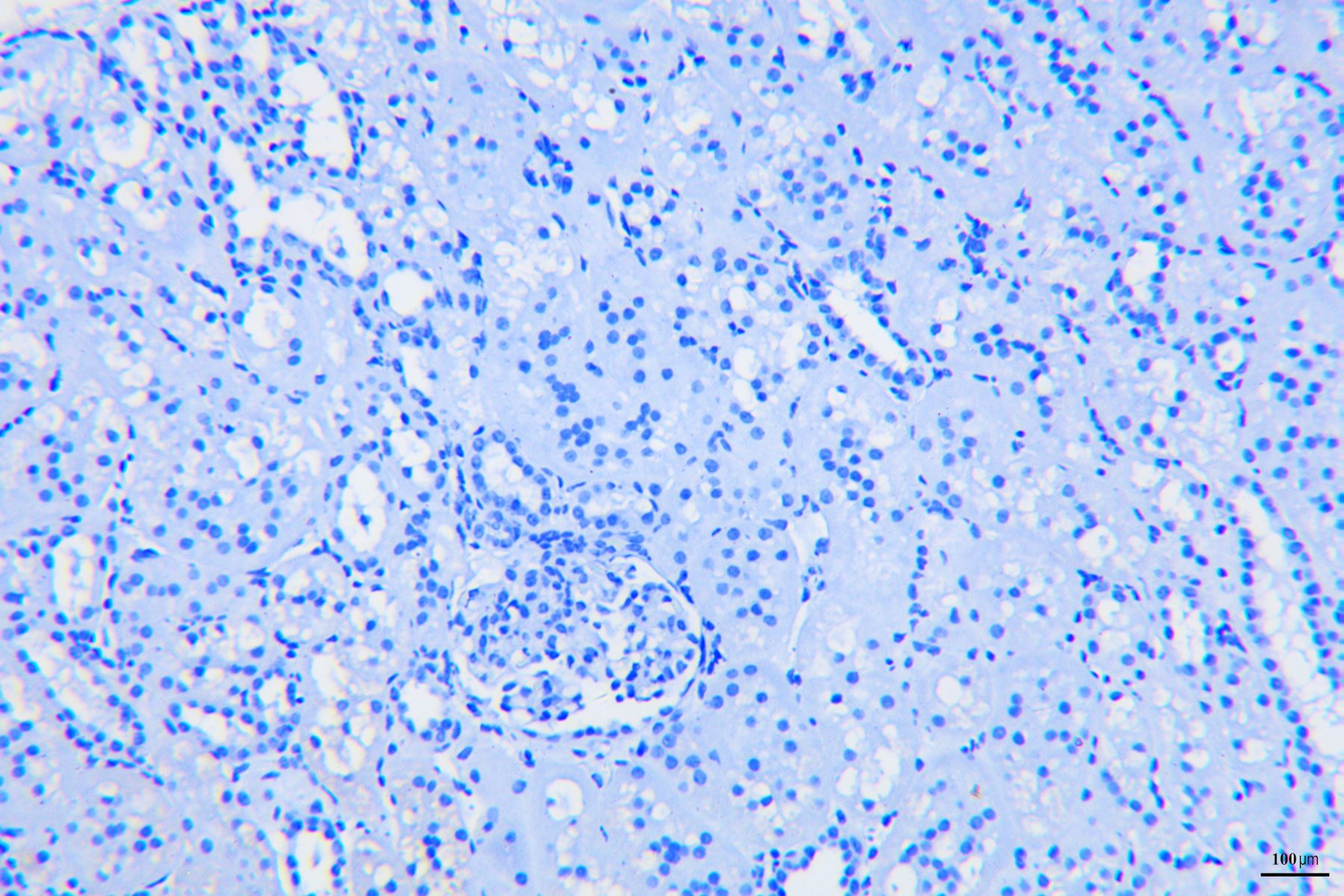


**Figure S1-059. KIM-1 immunohistochemistry; sample or target: sham-16**


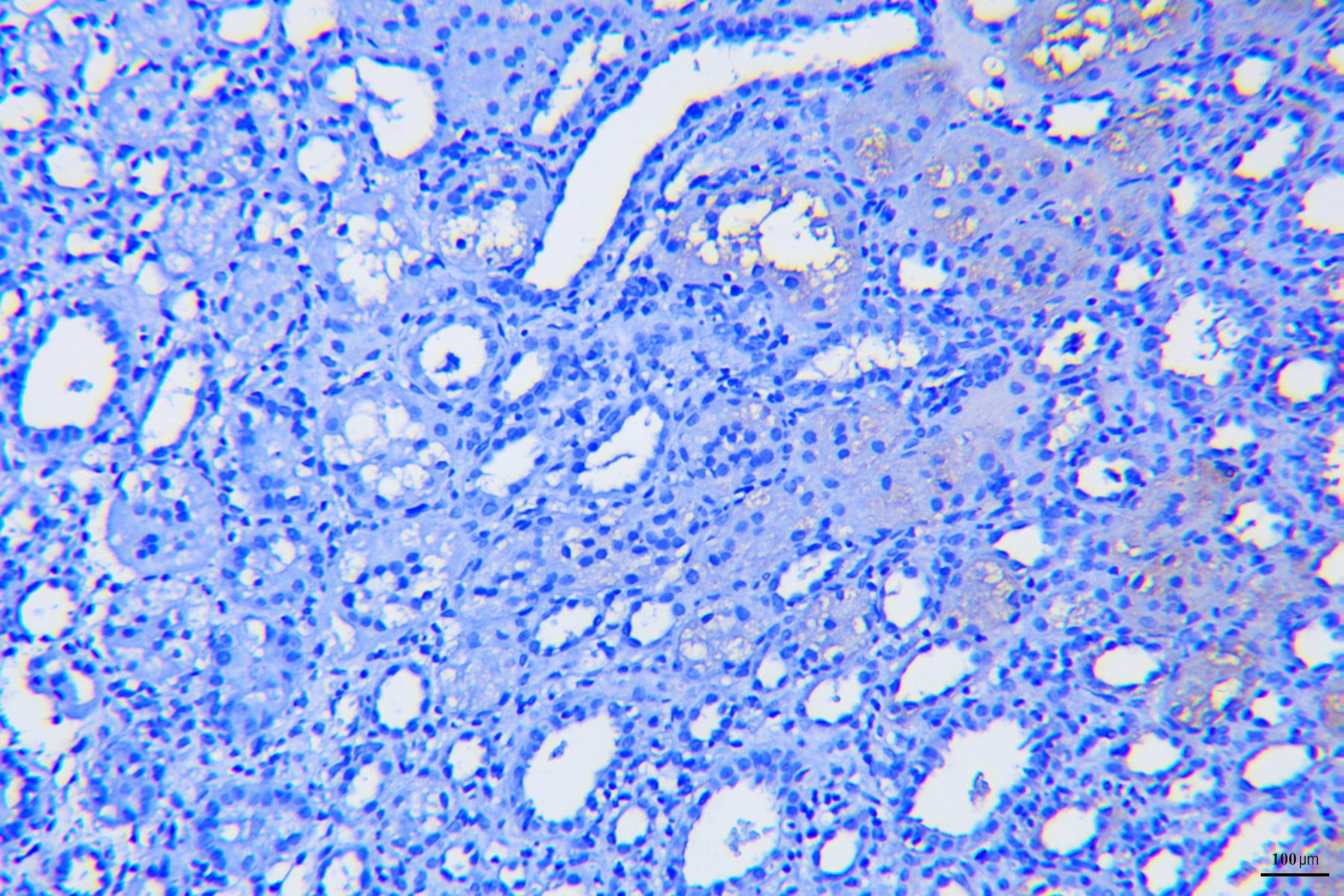


**Figure S1-060. KIM-1 immunohistochemistry; sample or target: sham-23**


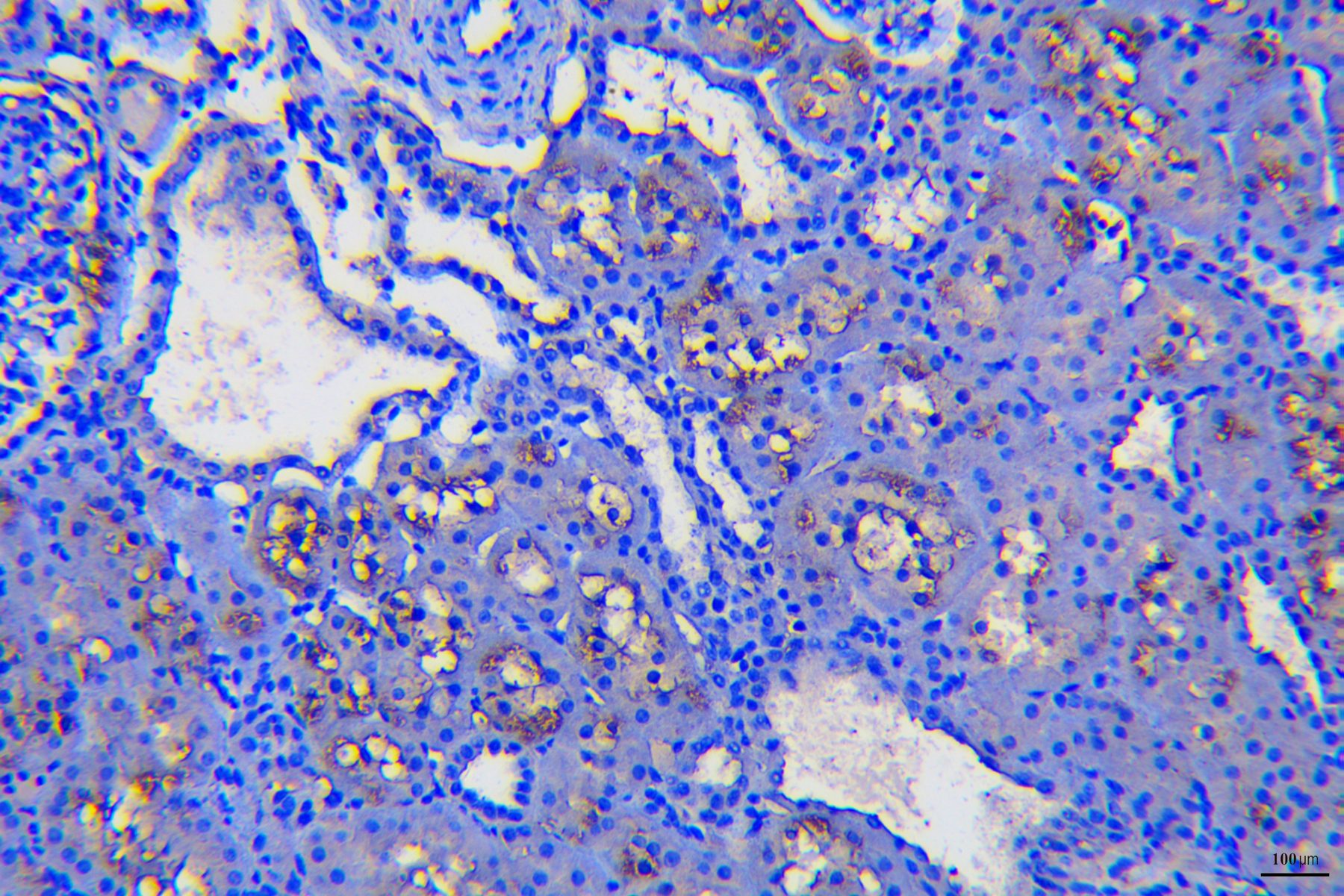


**Figure S1-061. KIM-1 immunohistochemistry; sample or target: TCA+NBP-05**


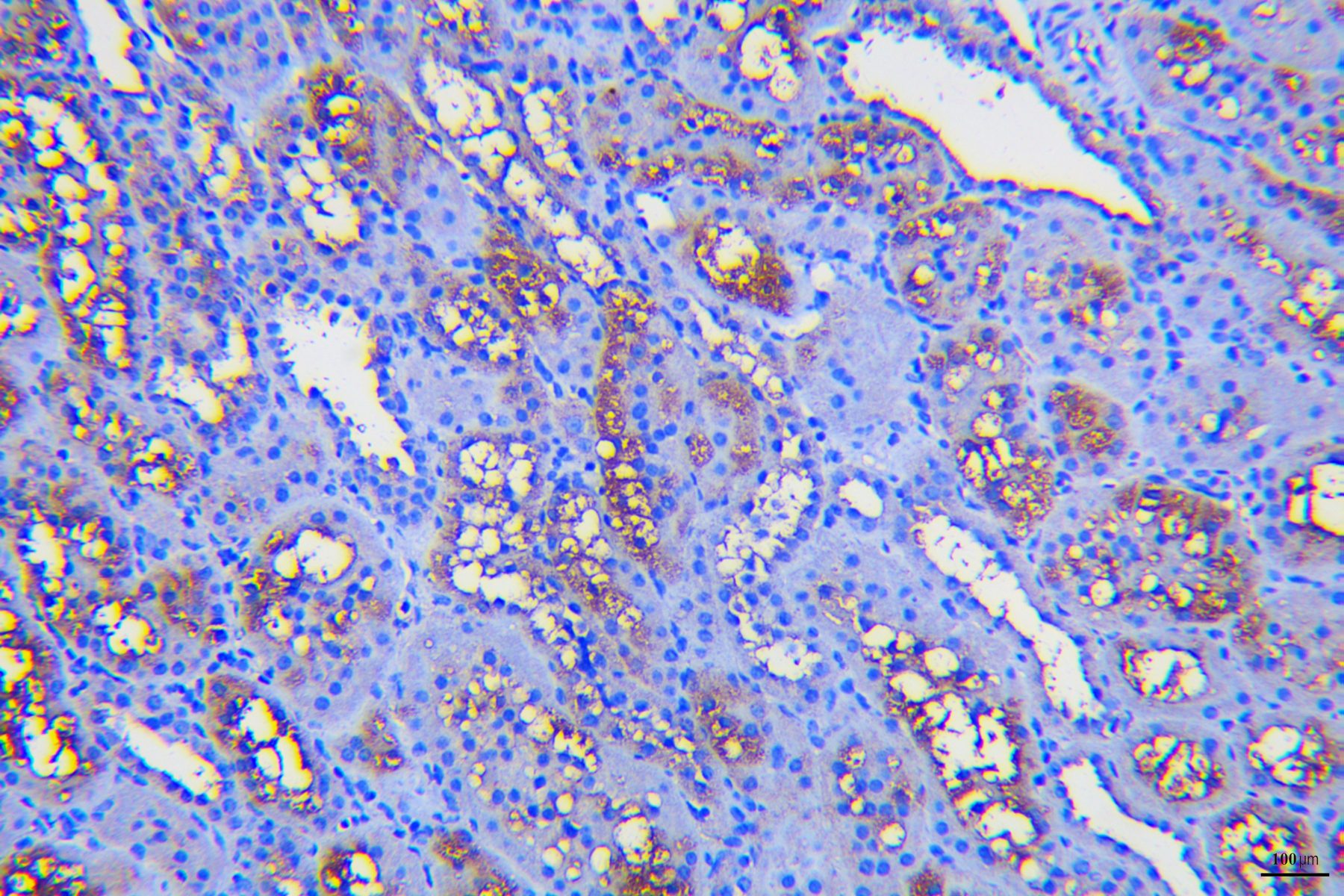


**Figure S1-062. KIM-1 immunohistochemistry; sample or target: TCA+NBP-07**


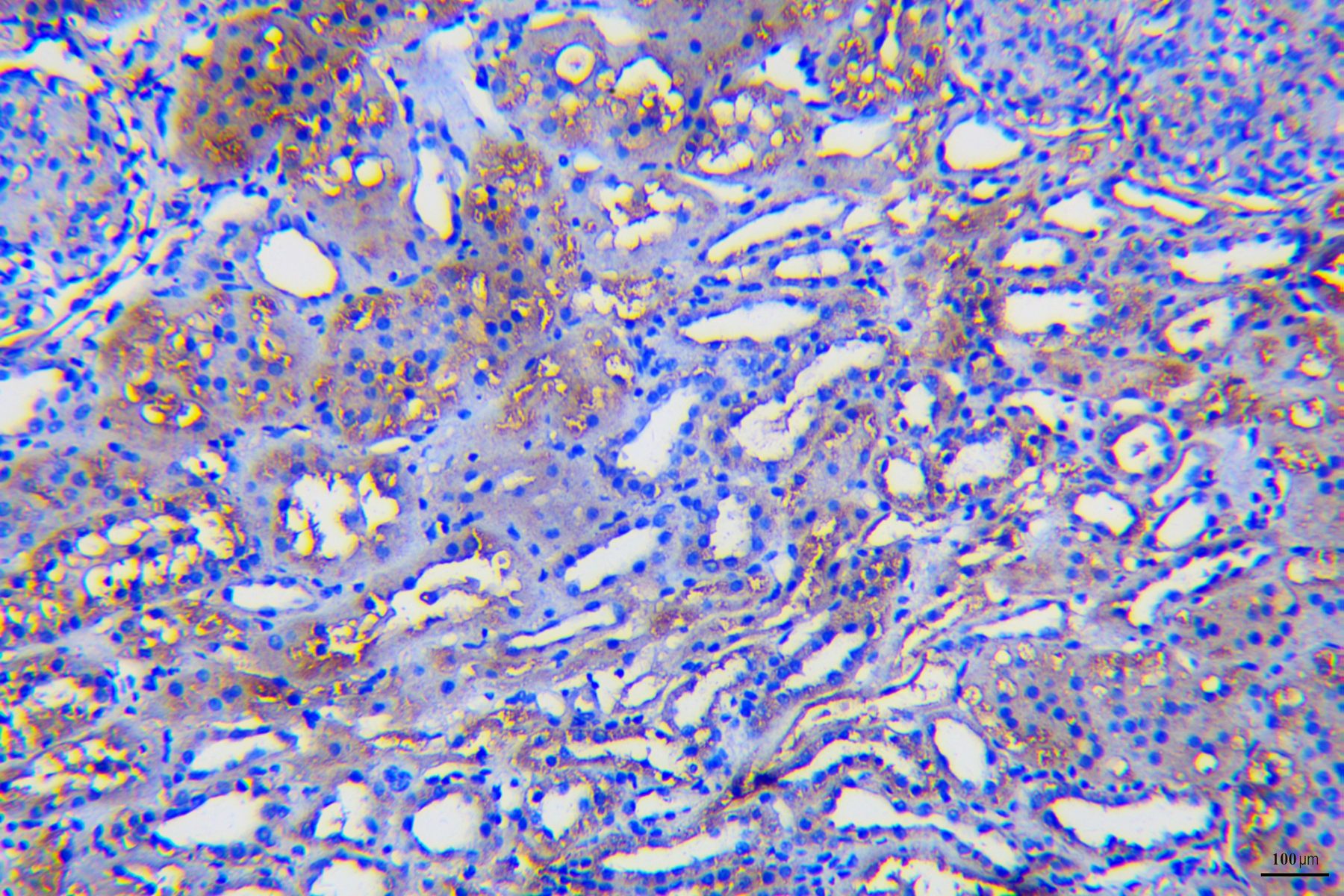


**Figure S1-063. KIM-1 immunohistochemistry; sample or target: TCA+NBP-17**


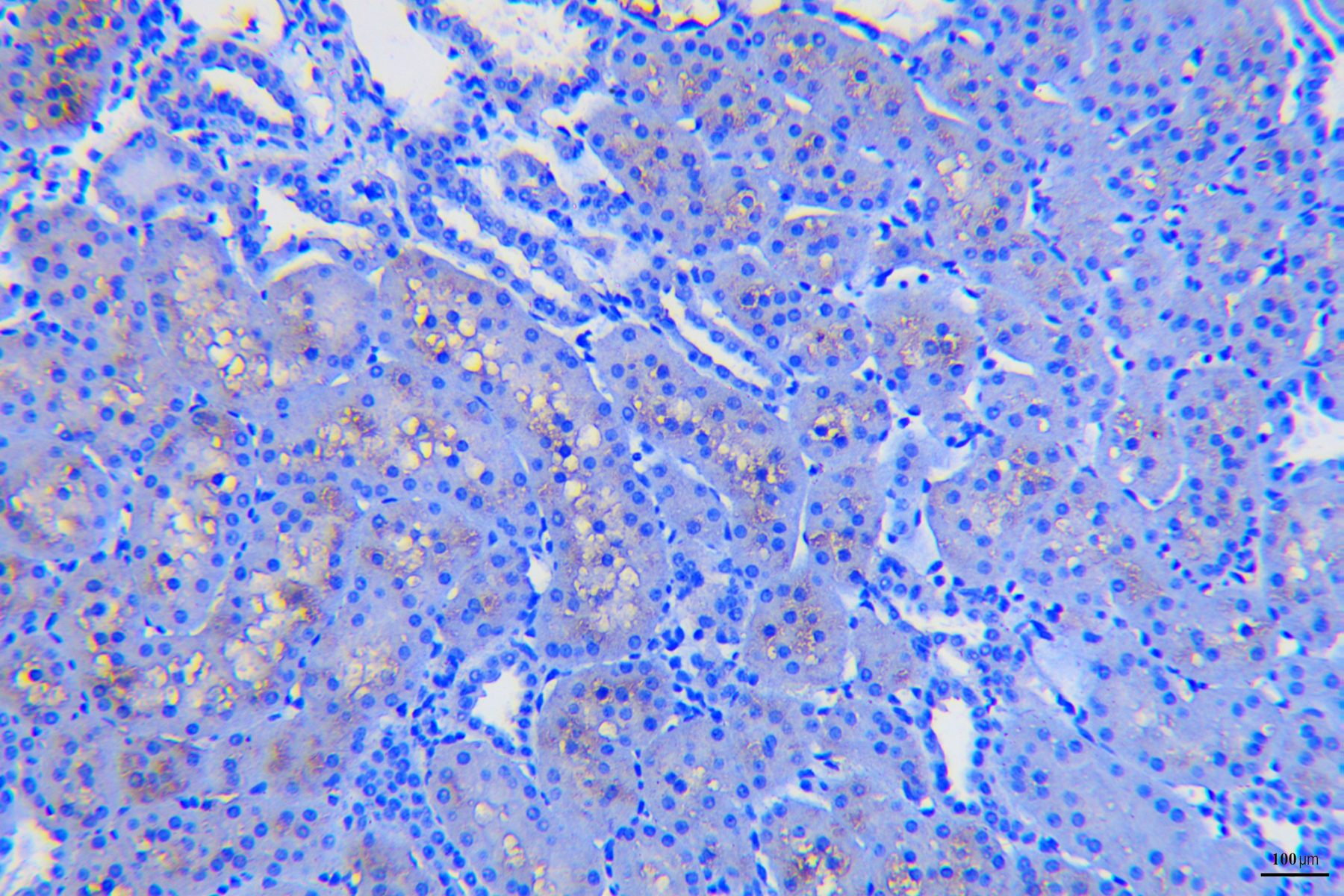


**Figure S1-064. KIM-1 immunohistochemistry; sample or target: TCA+NBP-20**


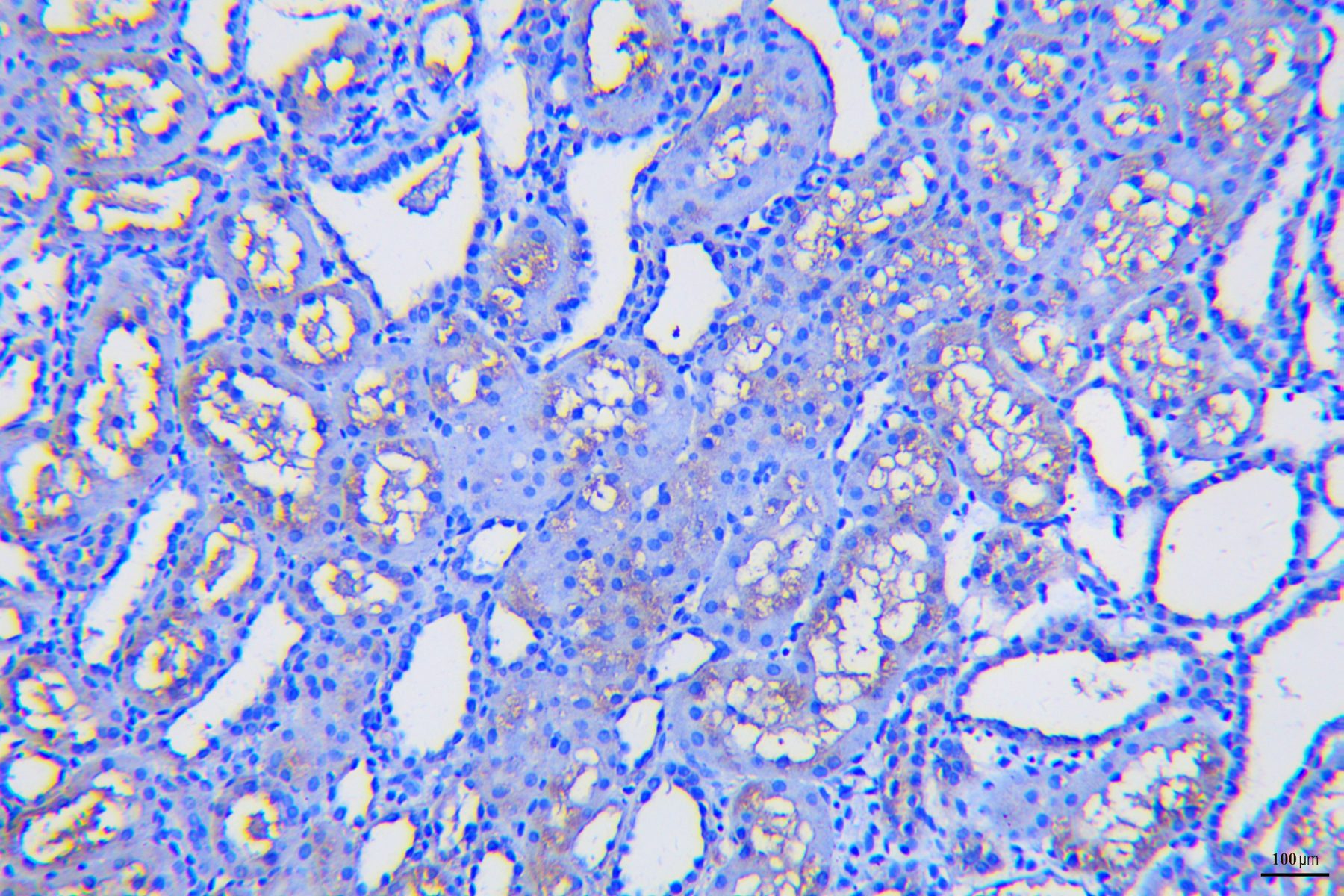


**Figure S1-065. KIM-1 immunohistochemistry; sample or target: TCA+NBP-21**


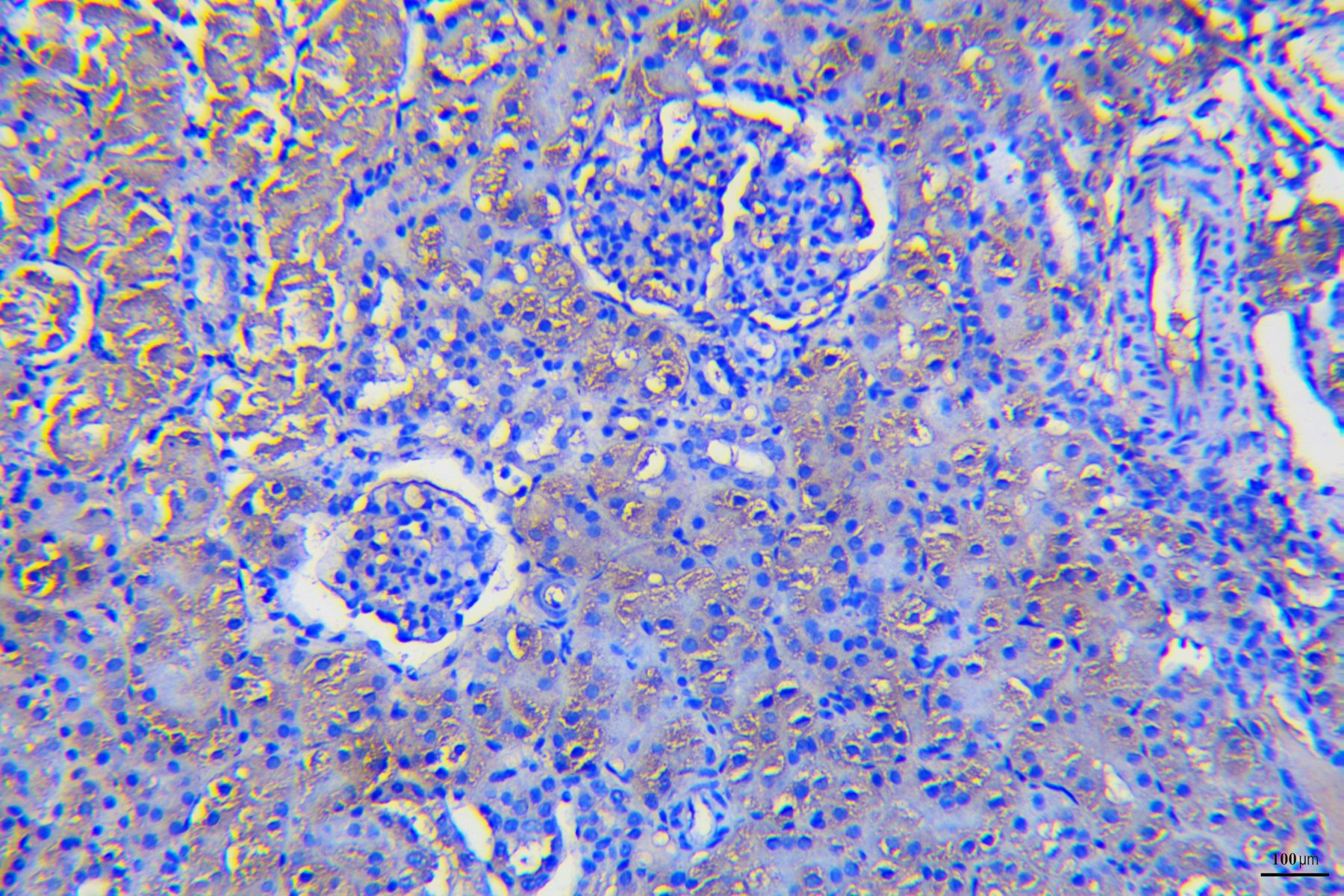


**Figure S1-066. KIM-1 immunohistochemistry; sample or target: TCA+NBP-22**


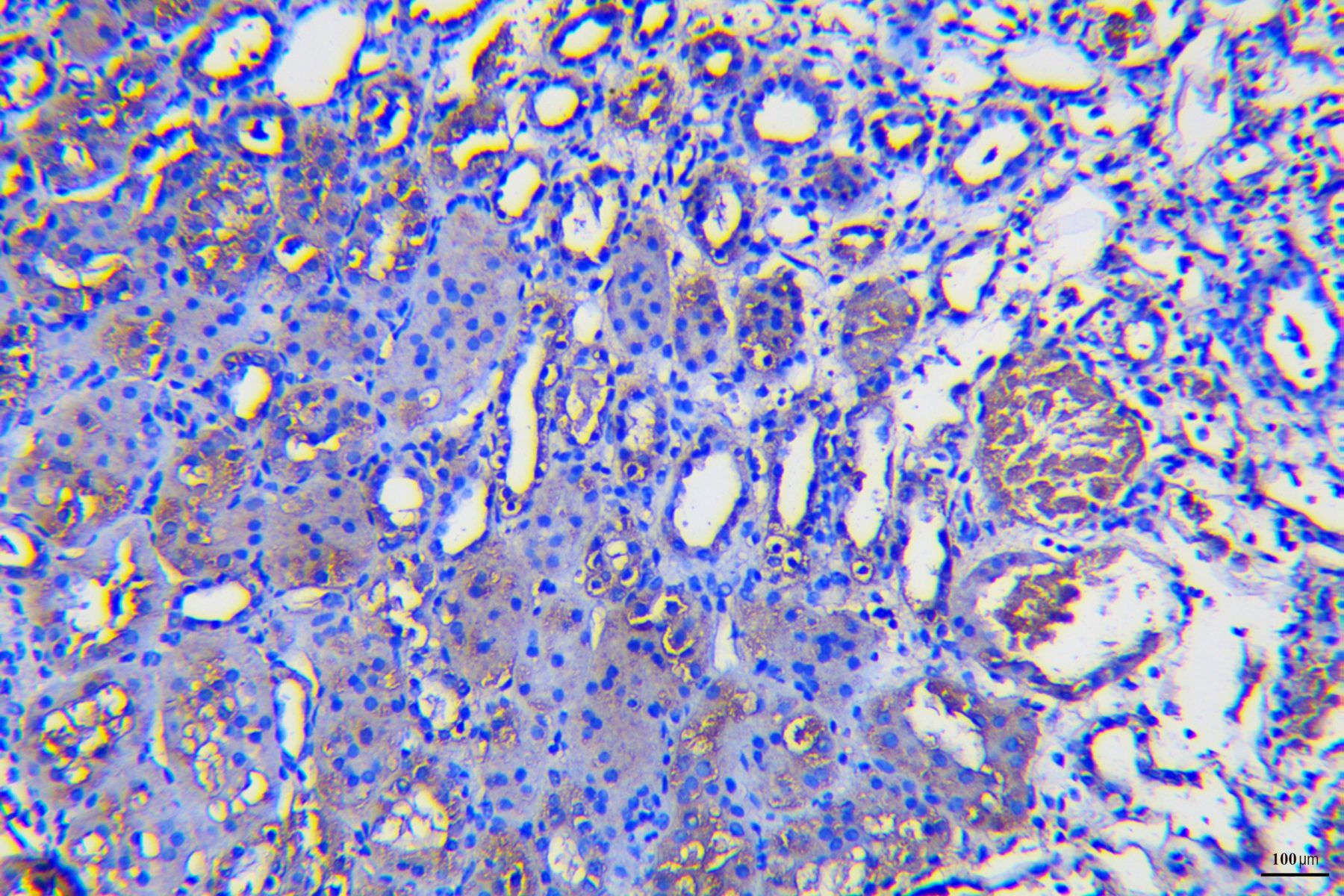


**Figure S1-067. KIM-1 immunohistochemistry; sample or target: TCA+NBP-26**


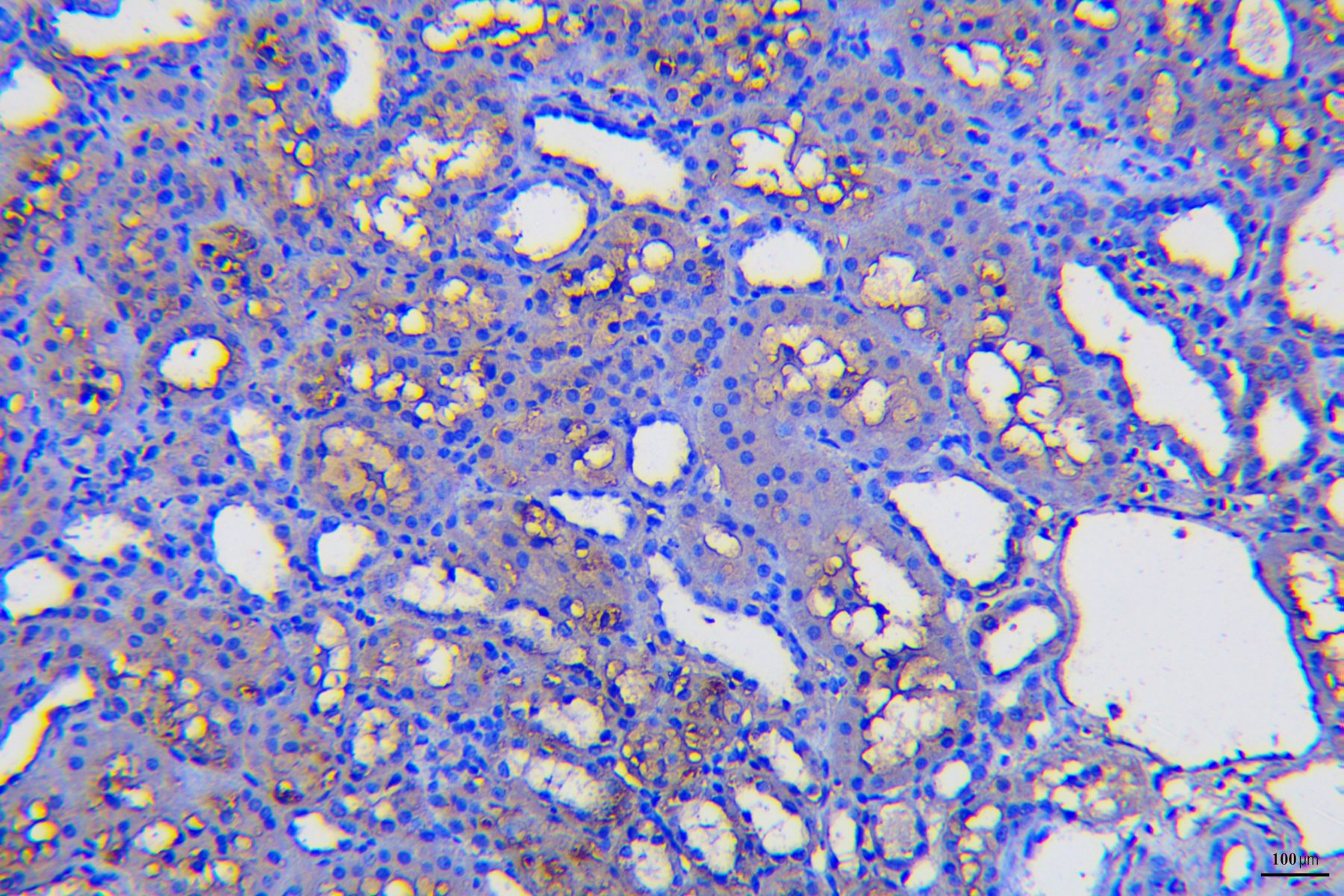


**Figure S1-068. KIM-1 immunohistochemistry; sample or target: TCA-01**


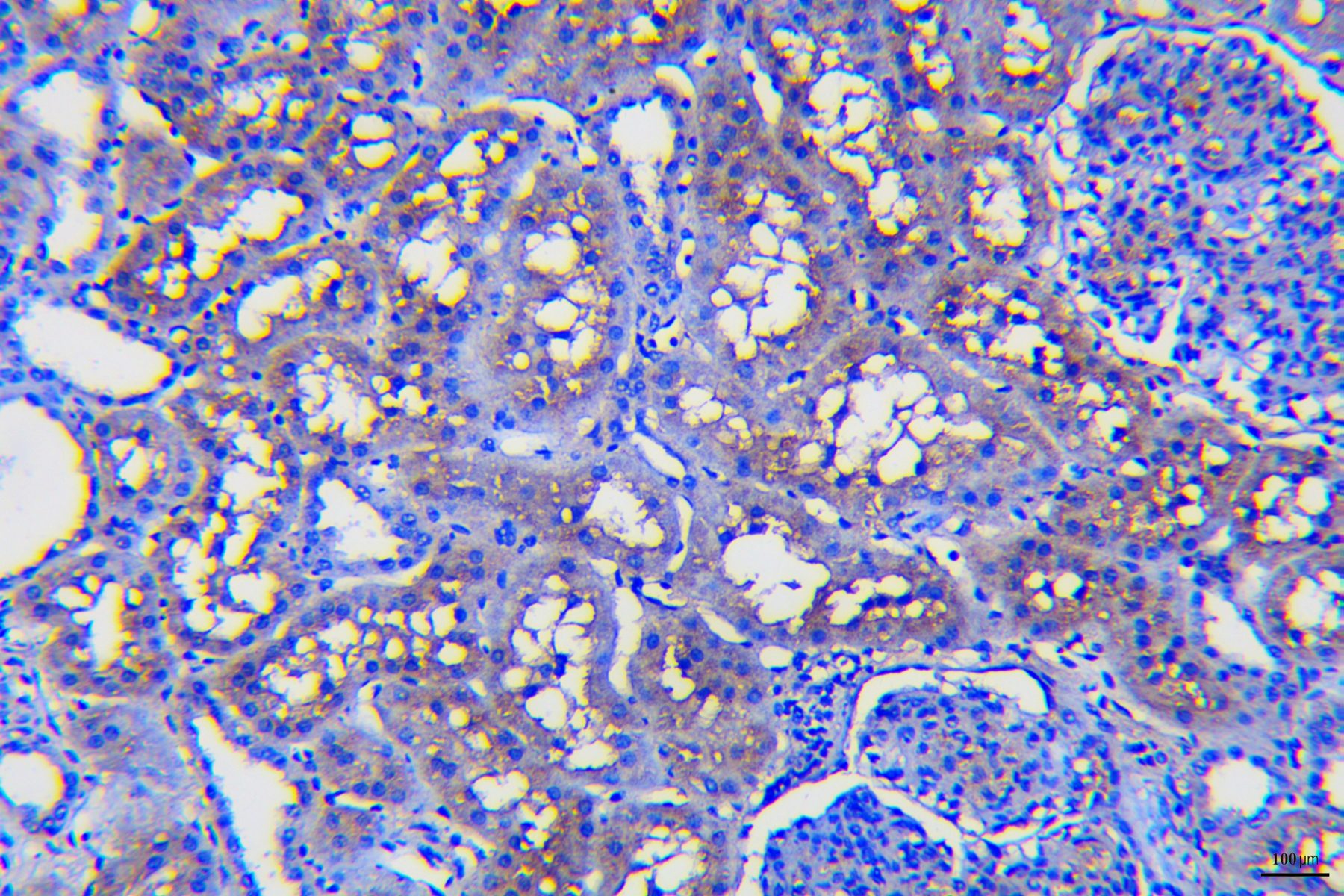


**Figure S1-069. KIM-1 immunohistochemistry; sample or target: TCA-02**


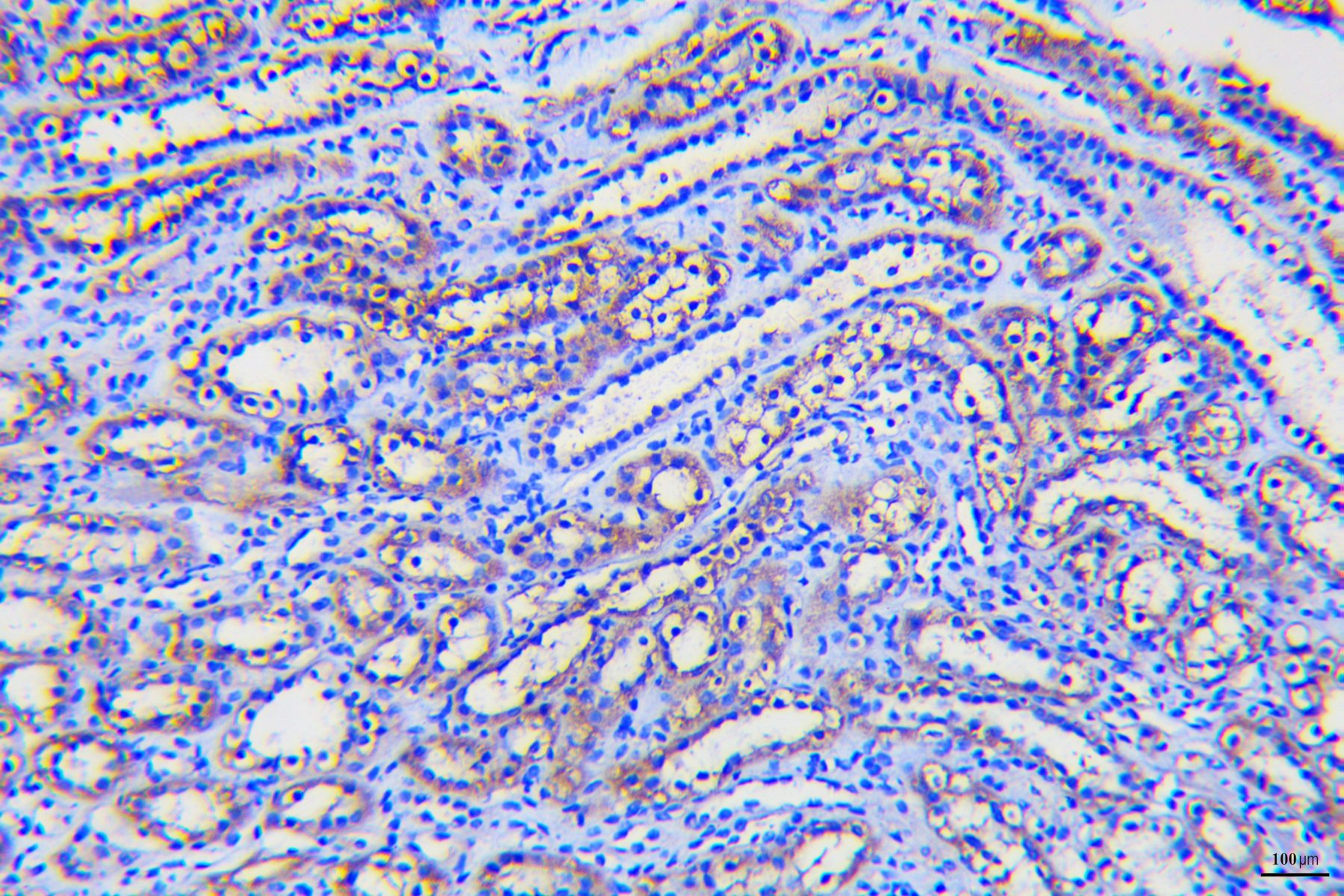


**Figure S1-070. KIM-1 immunohistochemistry; sample or target: TCA-04**


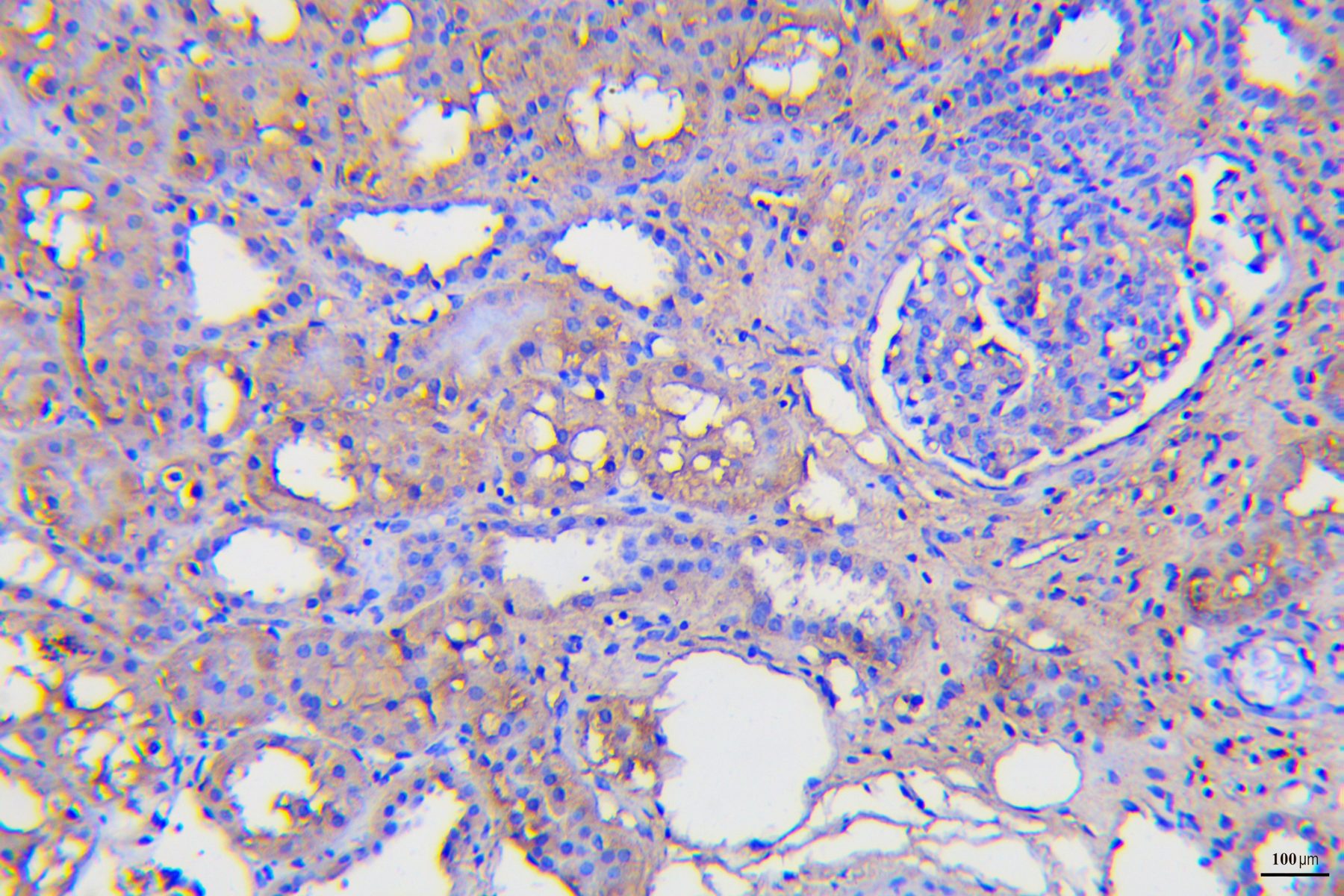


**Figure S1-071. KIM-1 immunohistochemistry; sample or target: TCA-10**


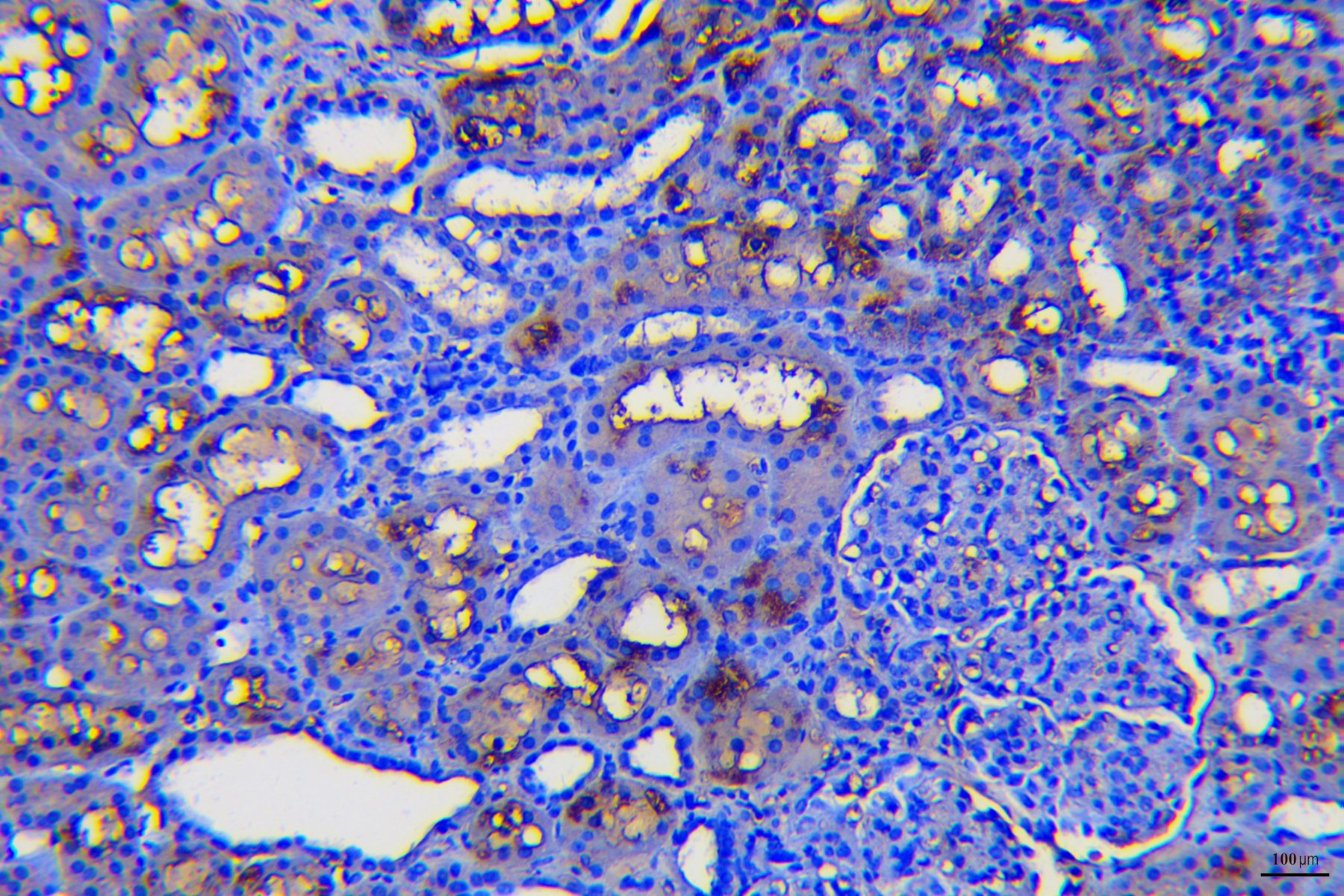


**Figure S1-072. KIM-1 immunohistochemistry; sample or target: TCA-11**

# Section: NGAL immunohistochemistry


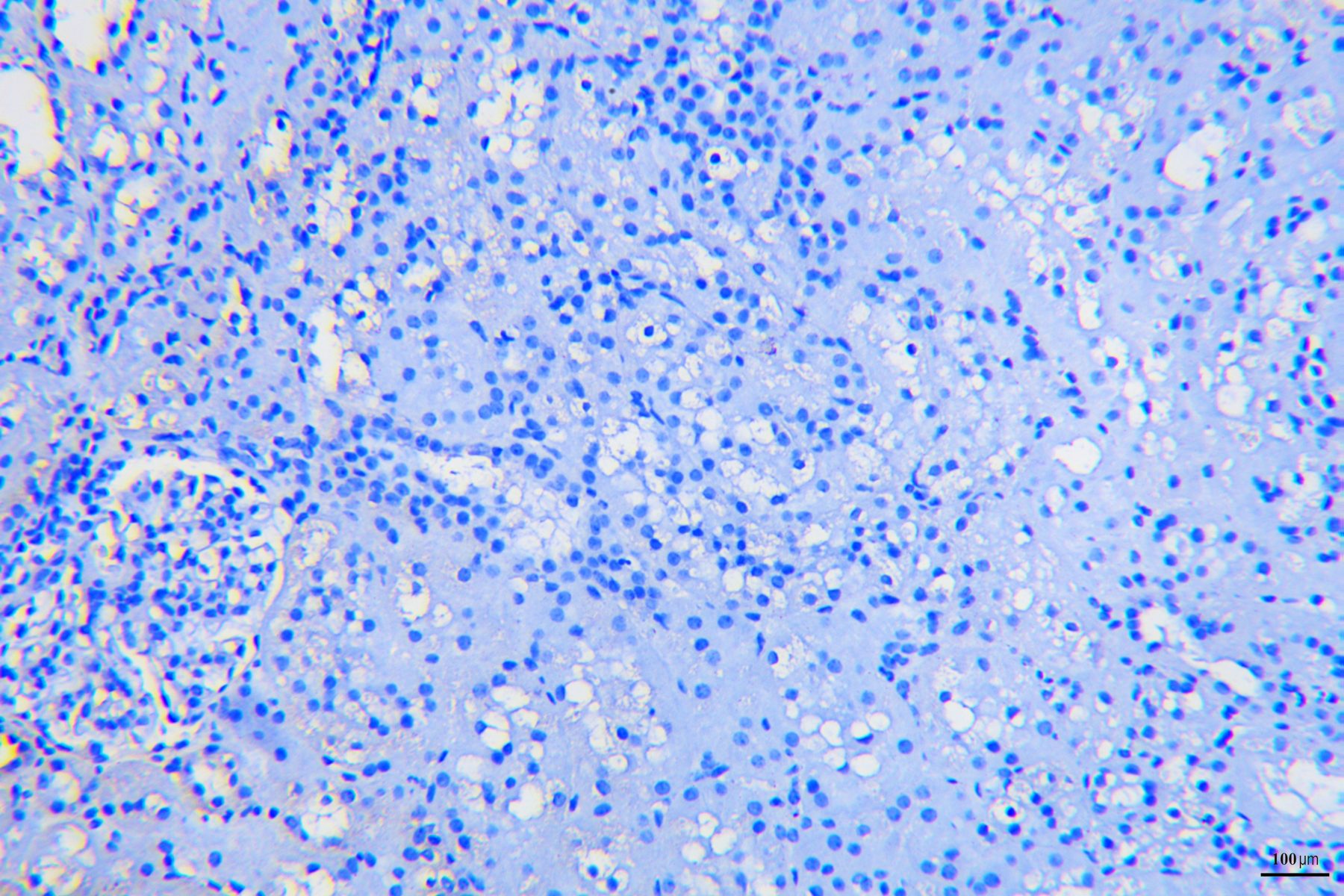


**Figure S1-073. NGAL immunohistochemistry; sample or target: sham-03**


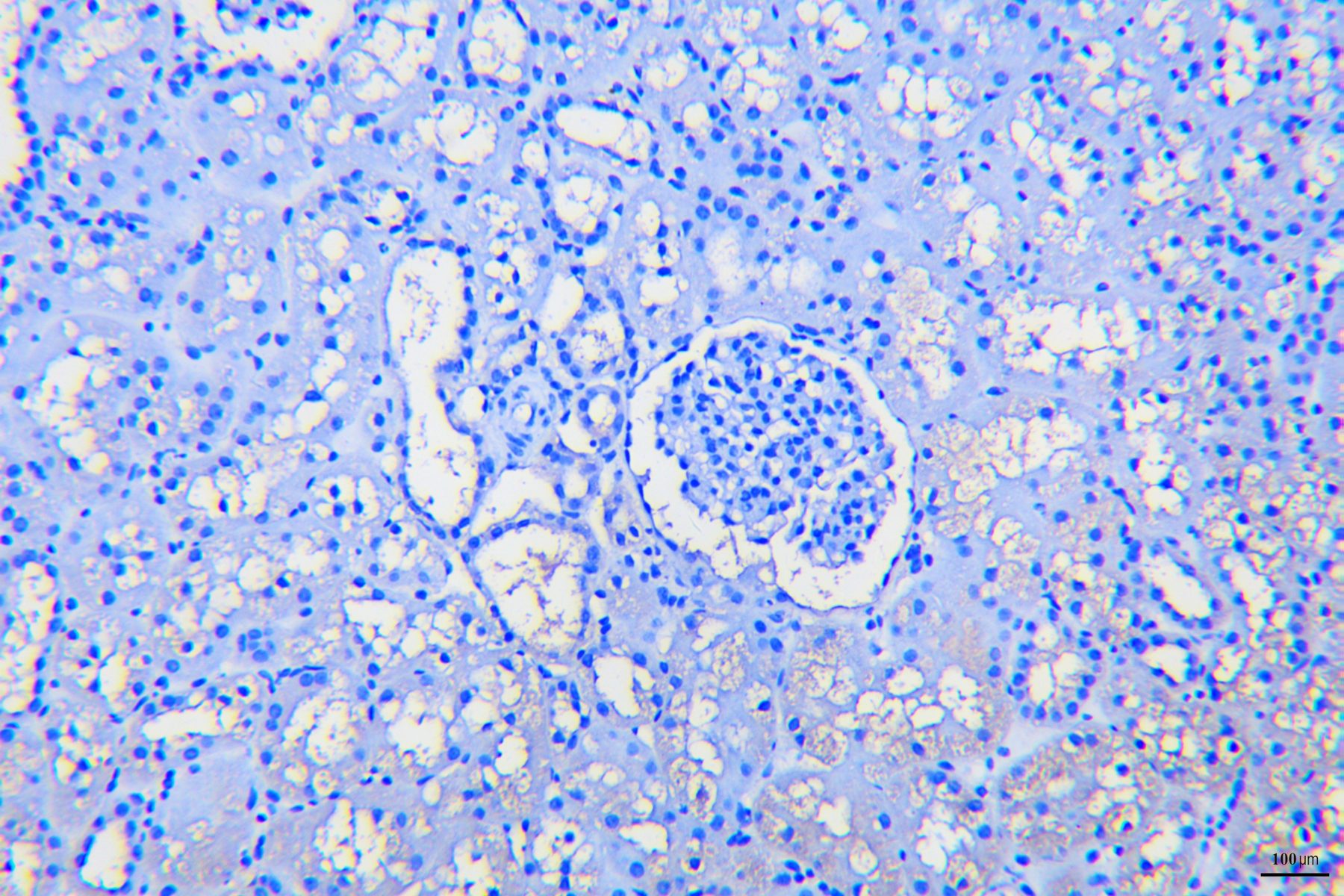


**Figure S1-074. NGAL immunohistochemistry; sample or target: sham-09**


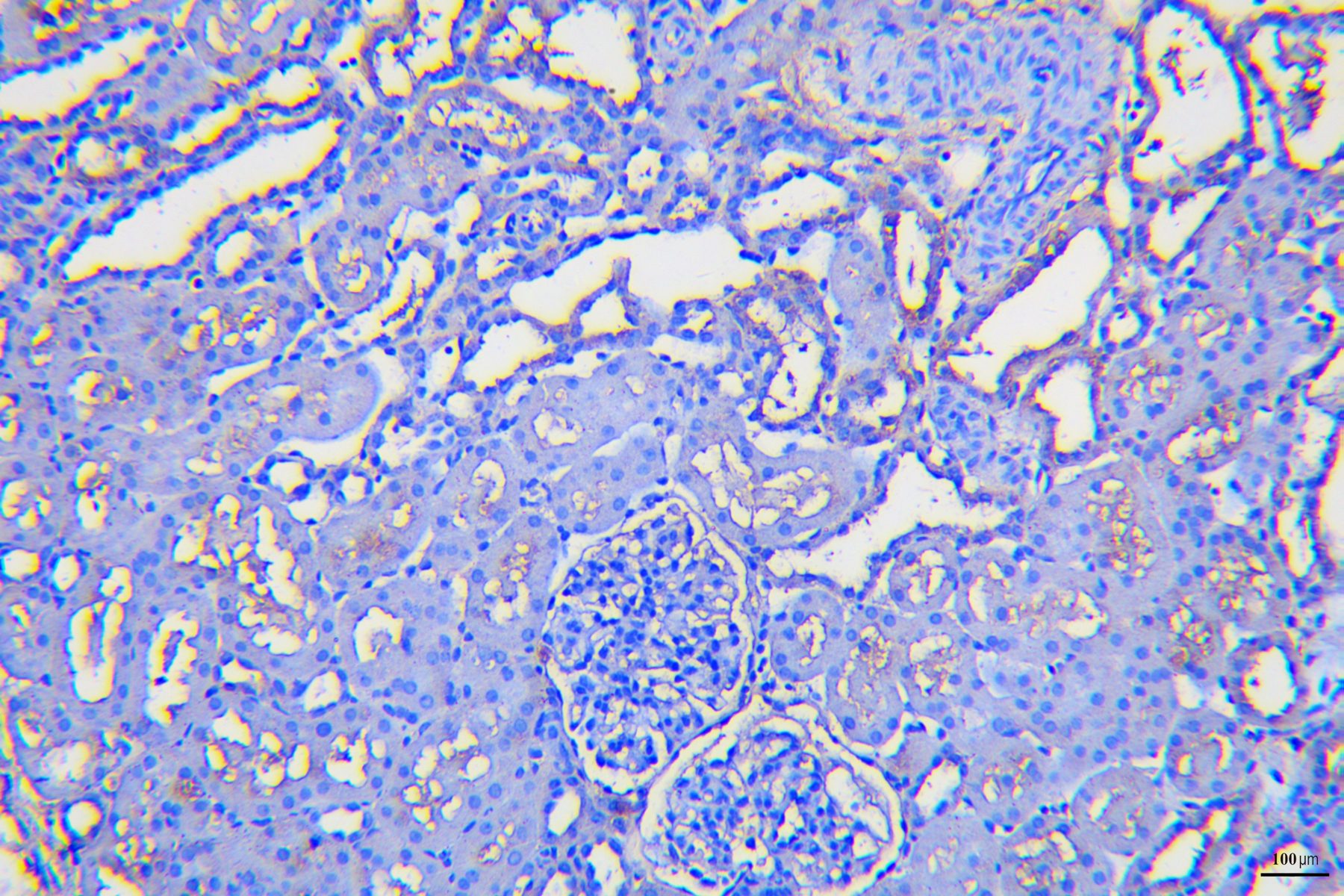


**Figure S1-075. NGAL immunohistochemistry; sample or target: sham-13**


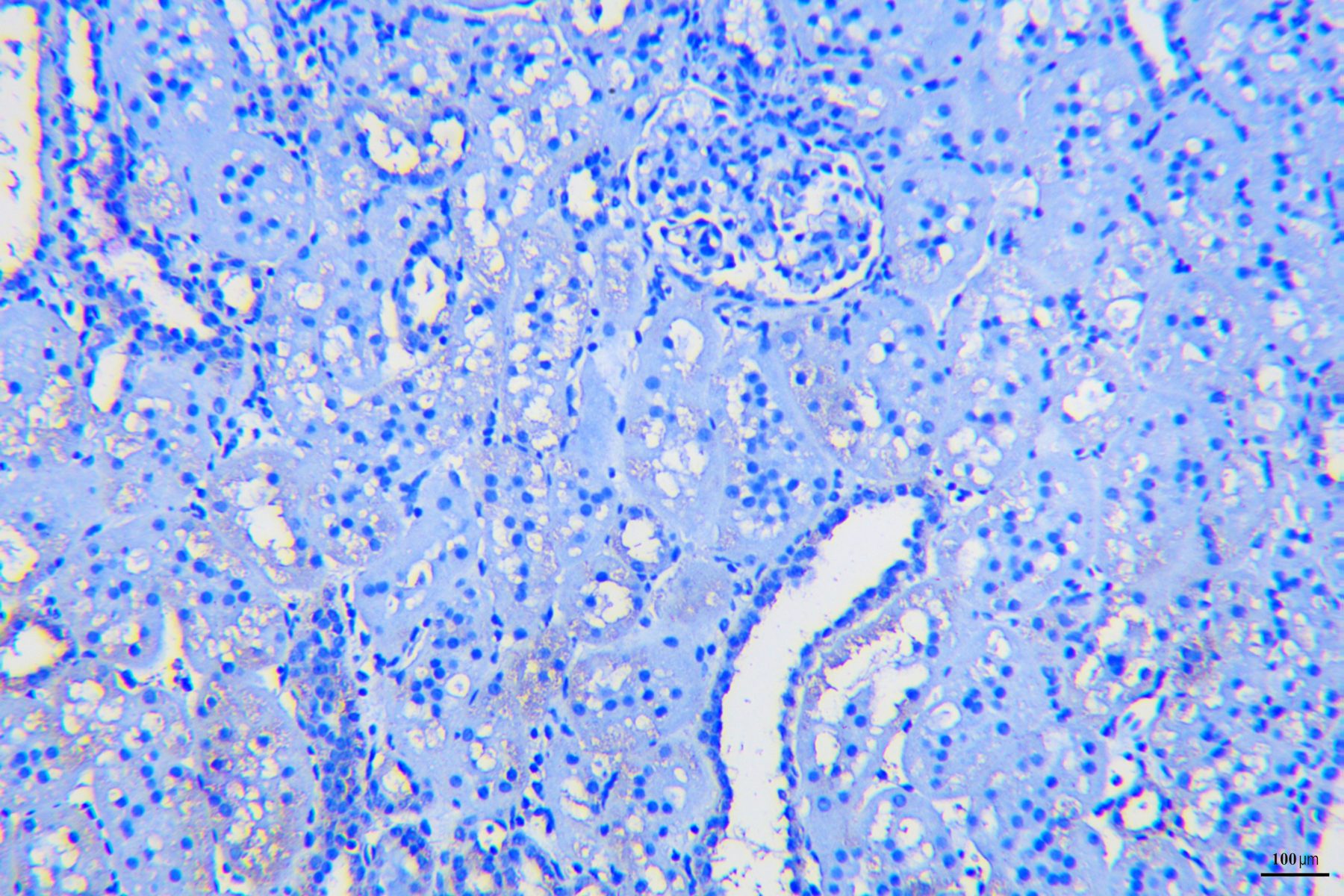


**Figure S1-076. NGAL immunohistochemistry; sample or target: sham-16**


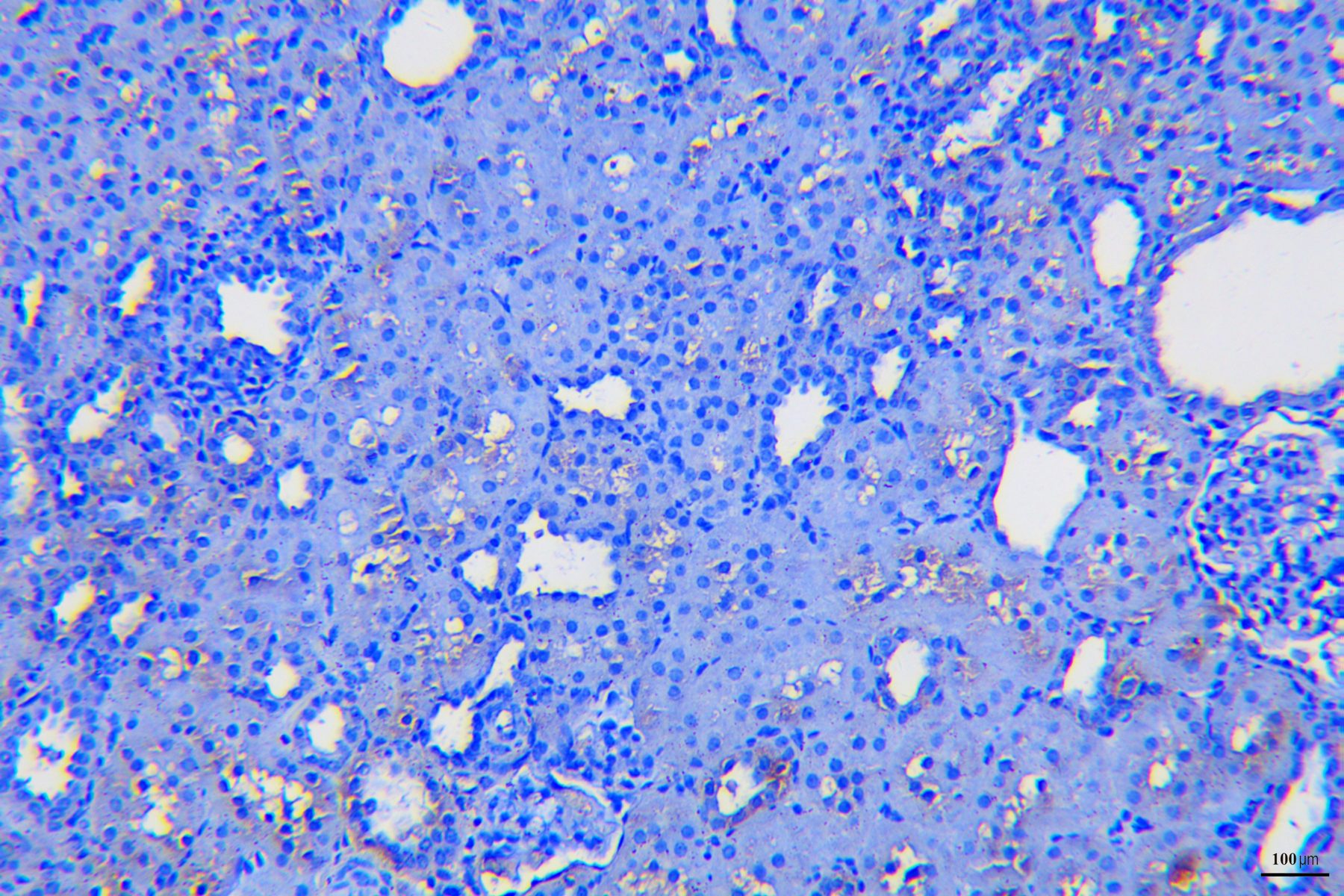


**Figure S1-077. NGAL immunohistochemistry; sample or target: sham-23**


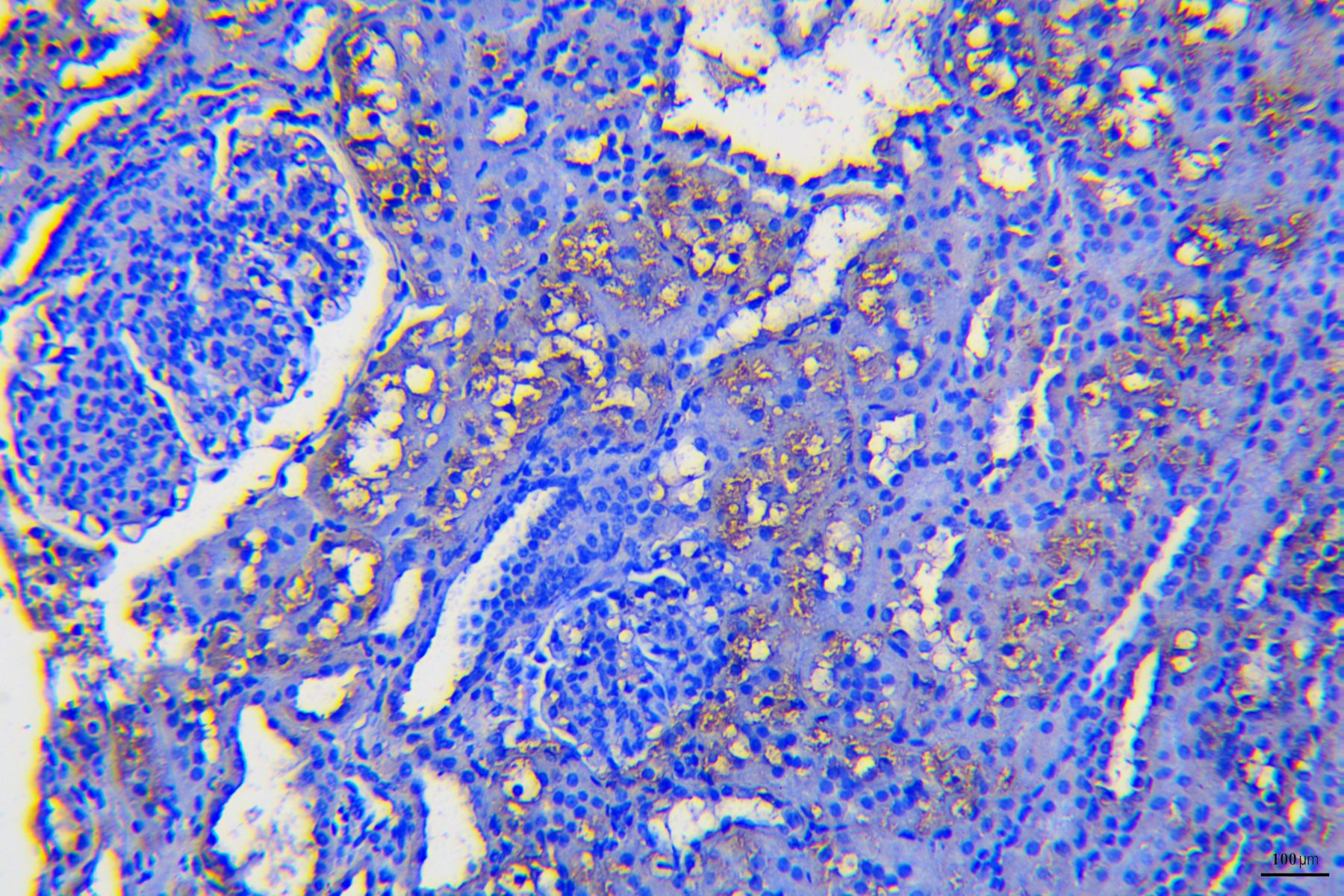


**Figure S1-078. NGAL immunohistochemistry; sample or target: TCA+NBP-05**


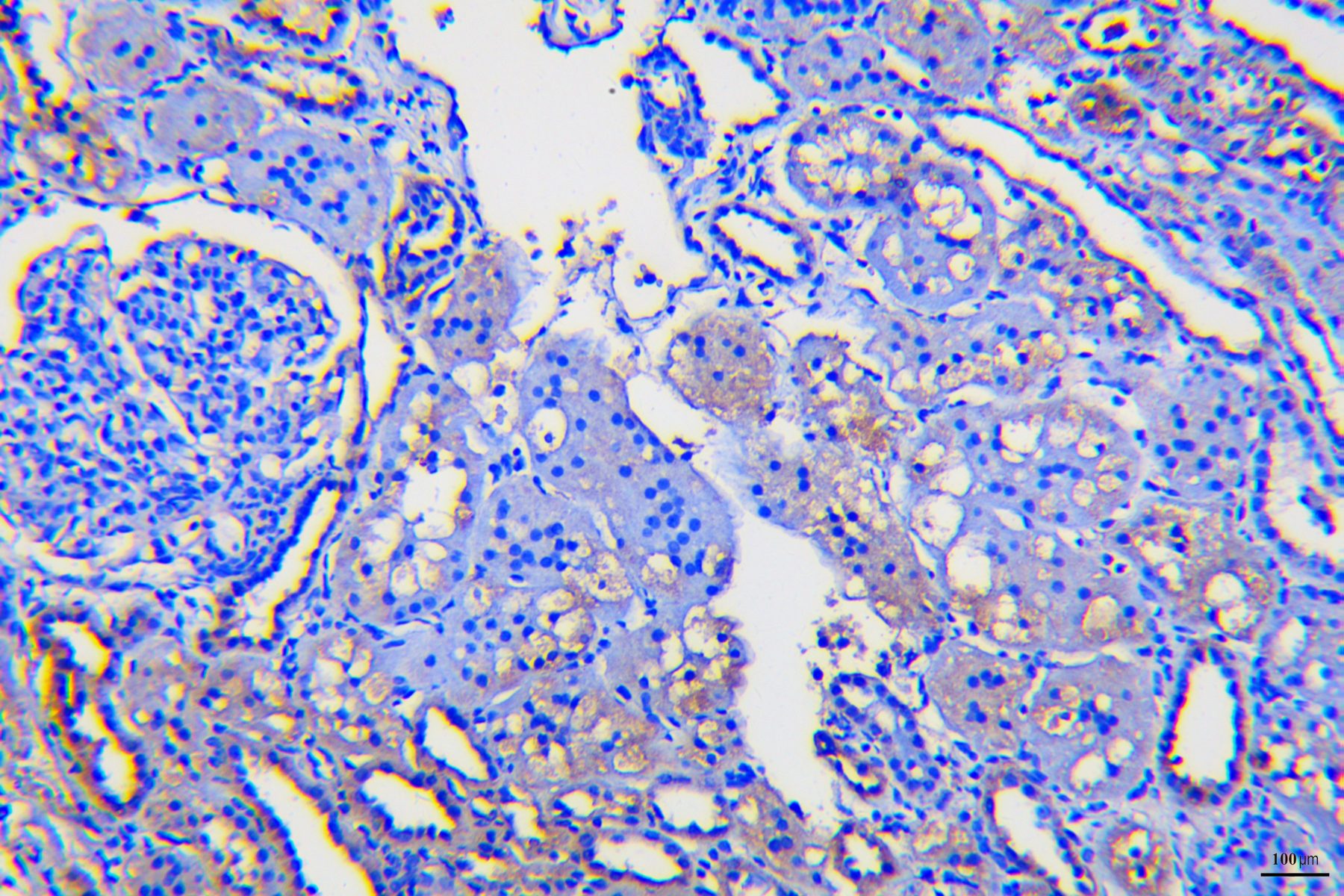


**Figure S1-079. NGAL immunohistochemistry; sample or target: TCA+NBP-07**


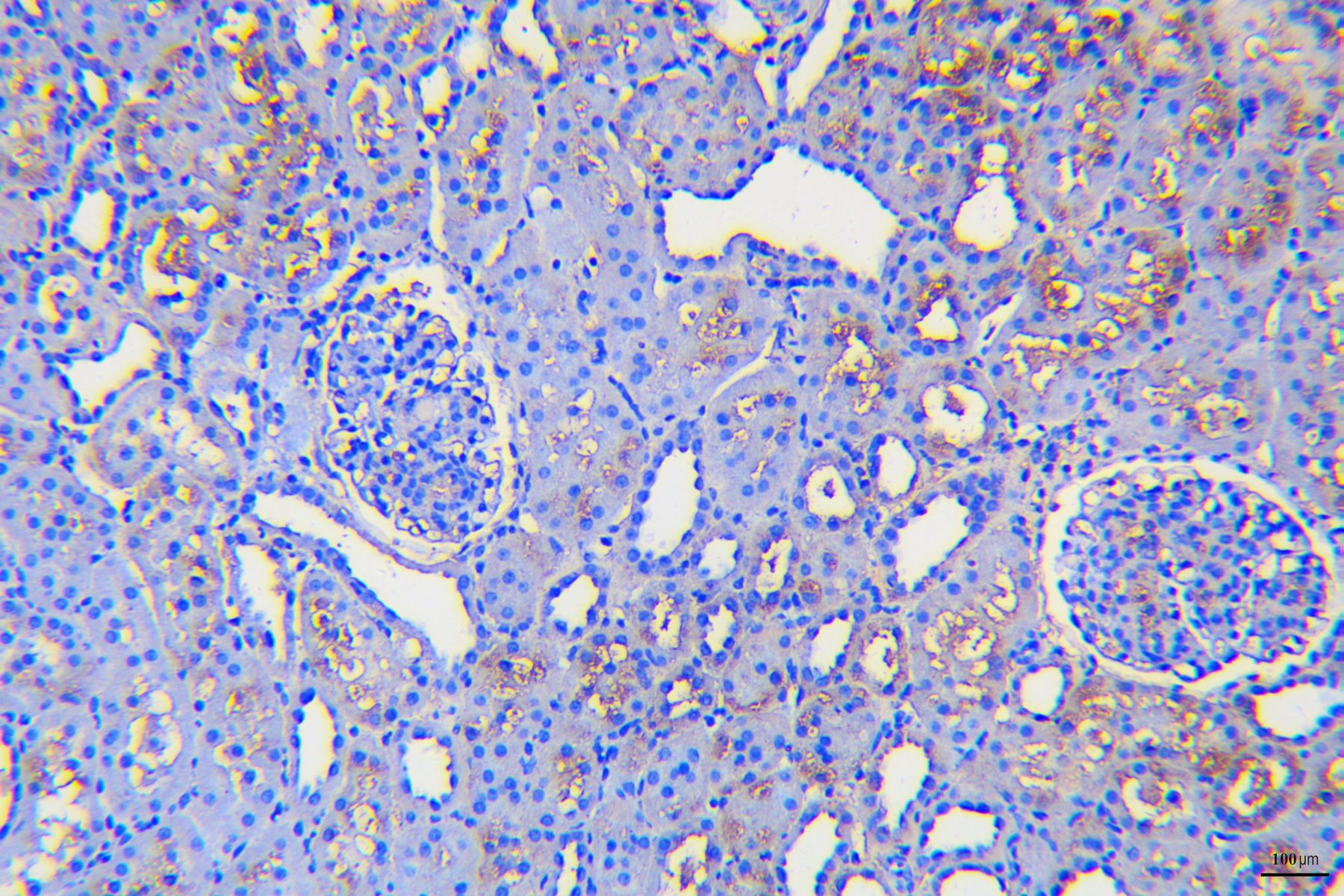


**Figure S1-080. NGAL immunohistochemistry; sample or target: TCA+NBP-17**


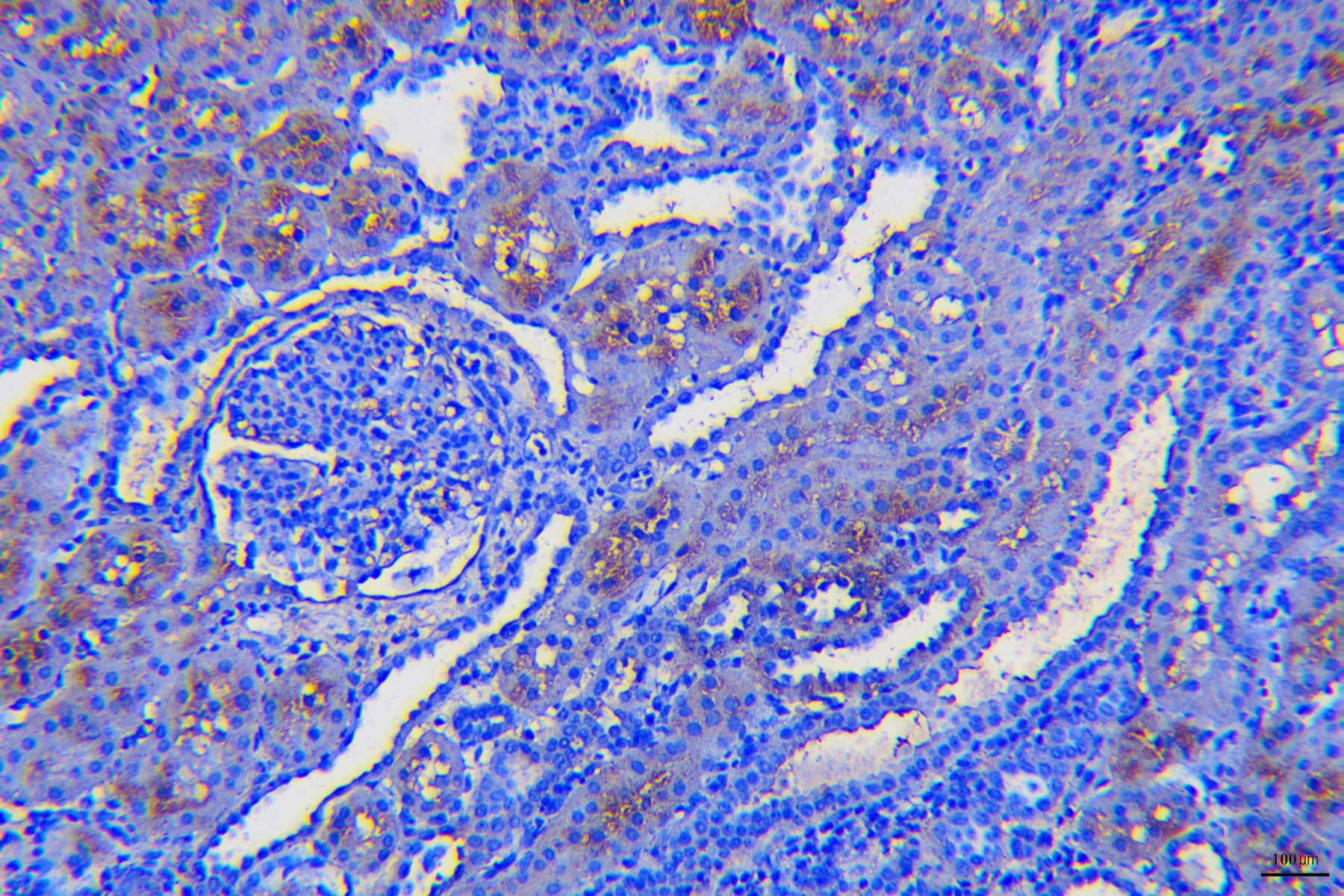


**Figure S1-081. NGAL immunohistochemistry; sample or target: TCA+NBP-20**


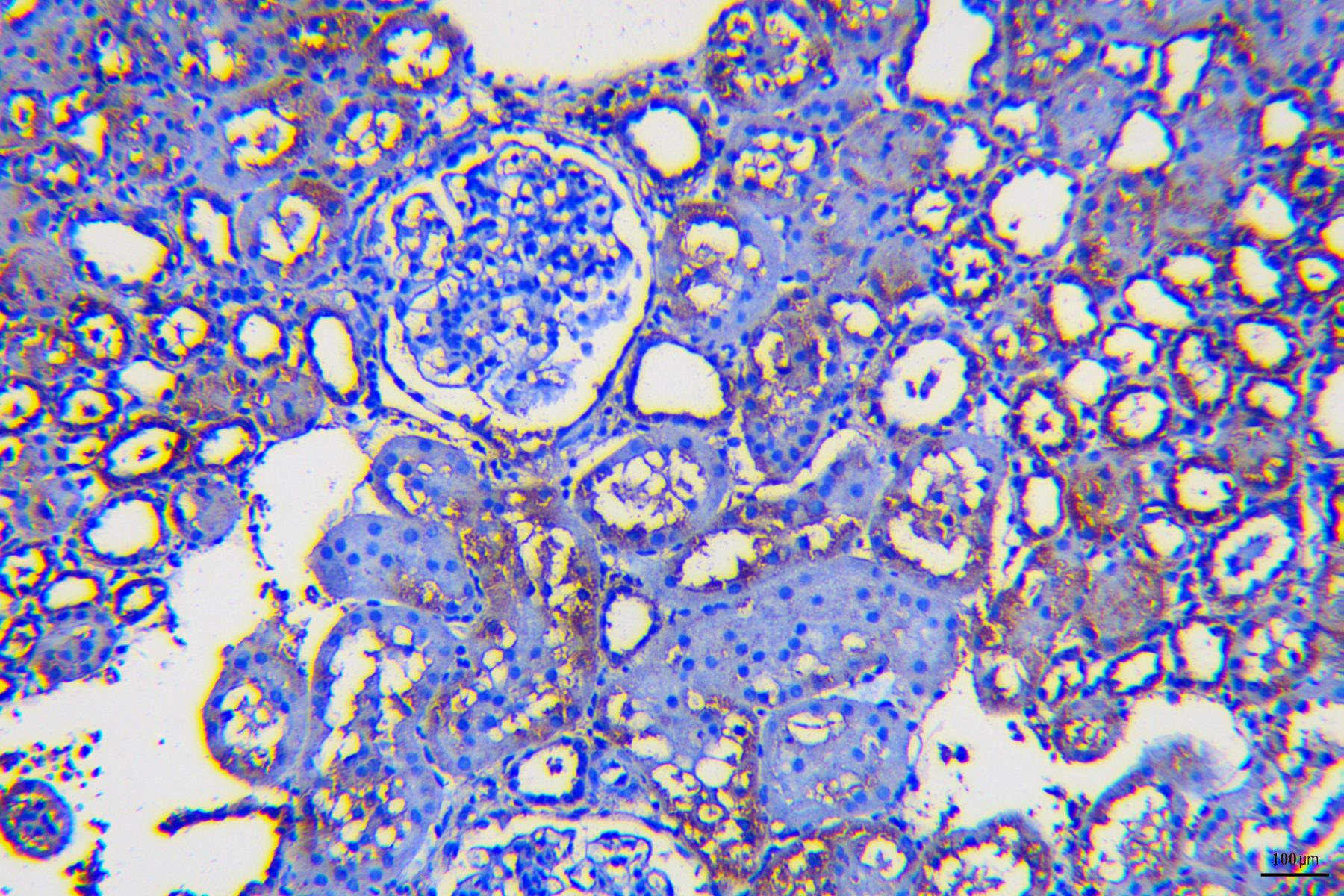


**Figure S1-082. NGAL immunohistochemistry; sample or target: TCA+NBP-21**


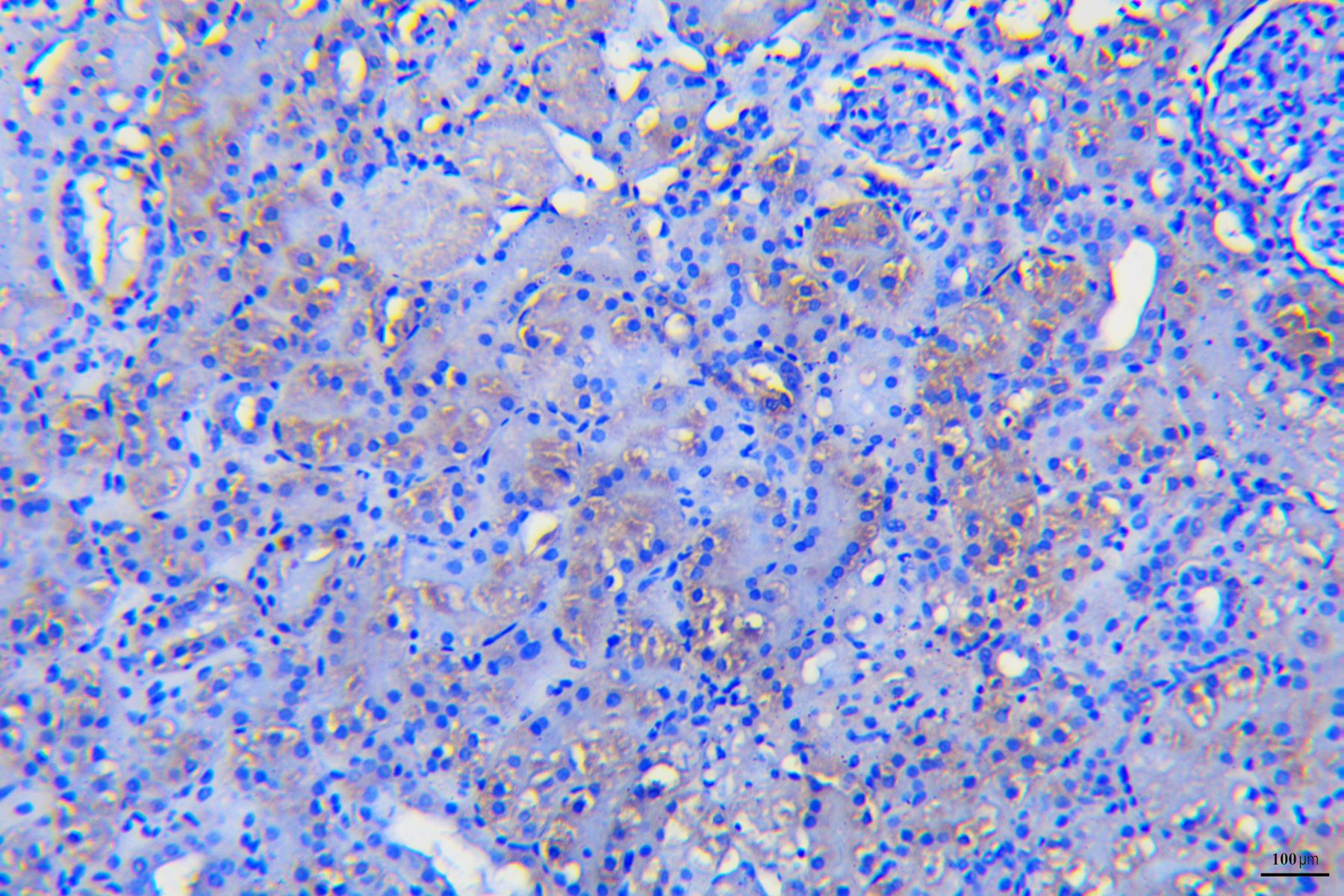


**Figure S1-083. NGAL immunohistochemistry; sample or target: TCA+NBP-22**


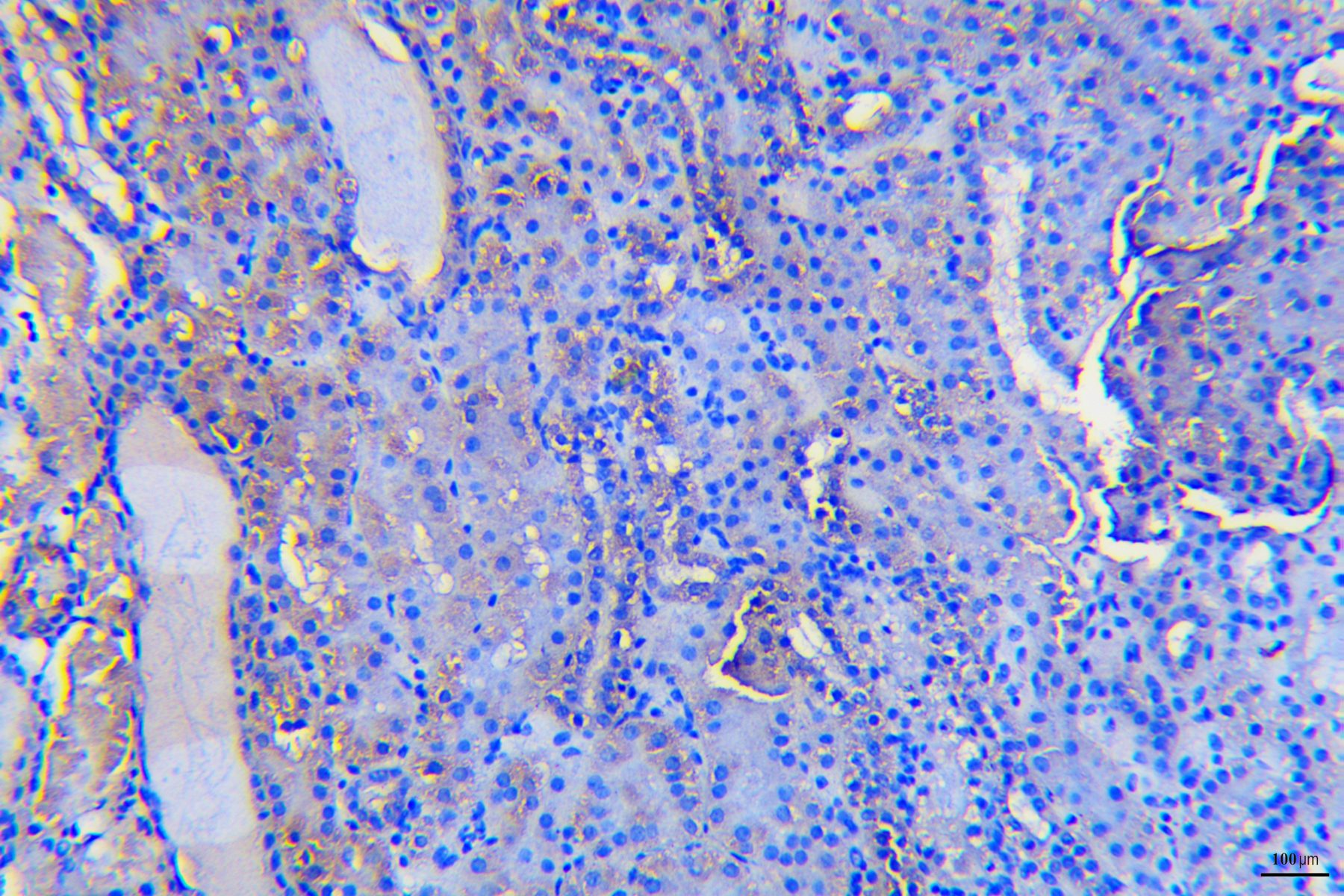


**Figure S1-084. NGAL immunohistochemistry; sample or target: TCA+NBP-26**


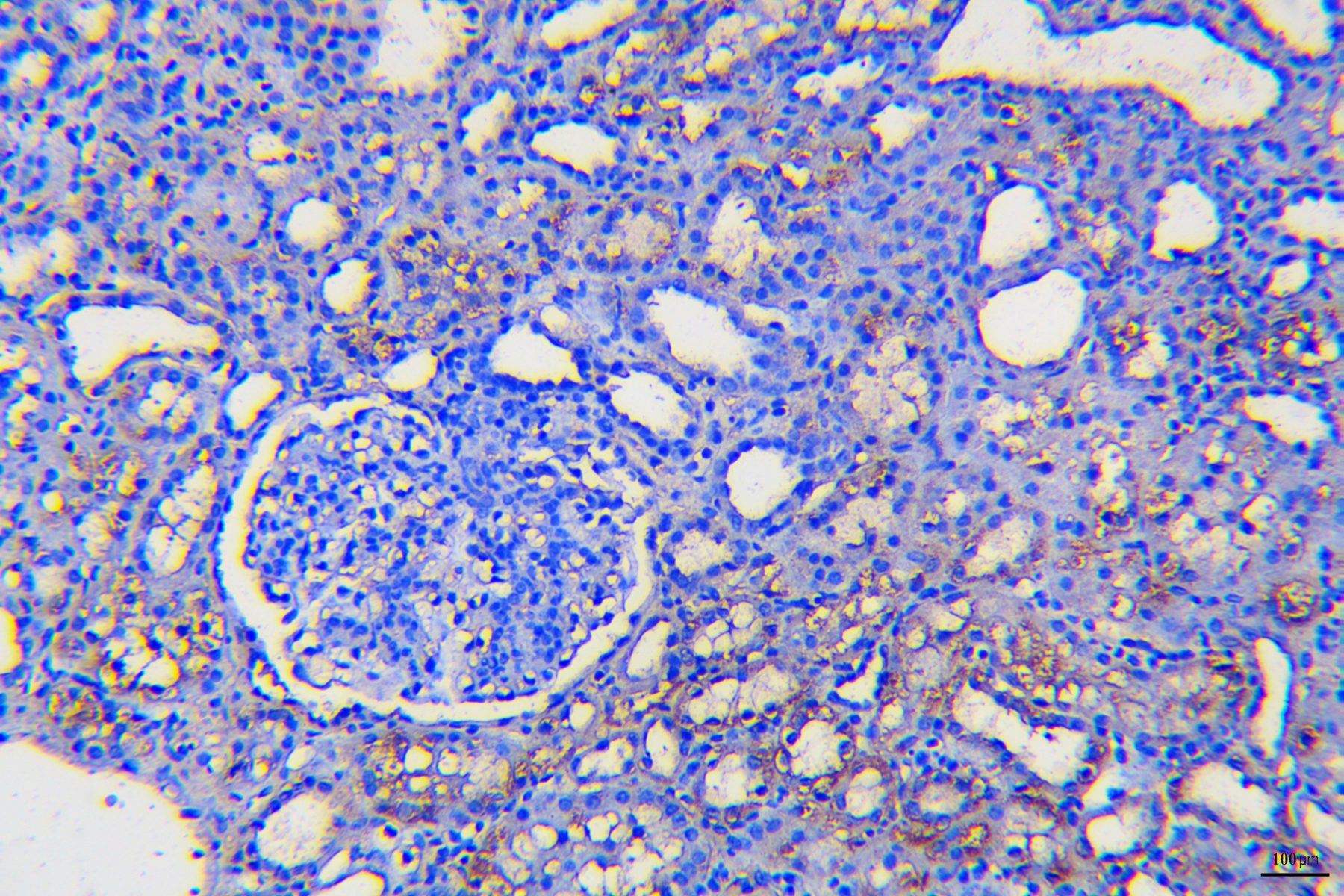


**Figure S1-085. NGAL immunohistochemistry; sample or target: TCA-01**


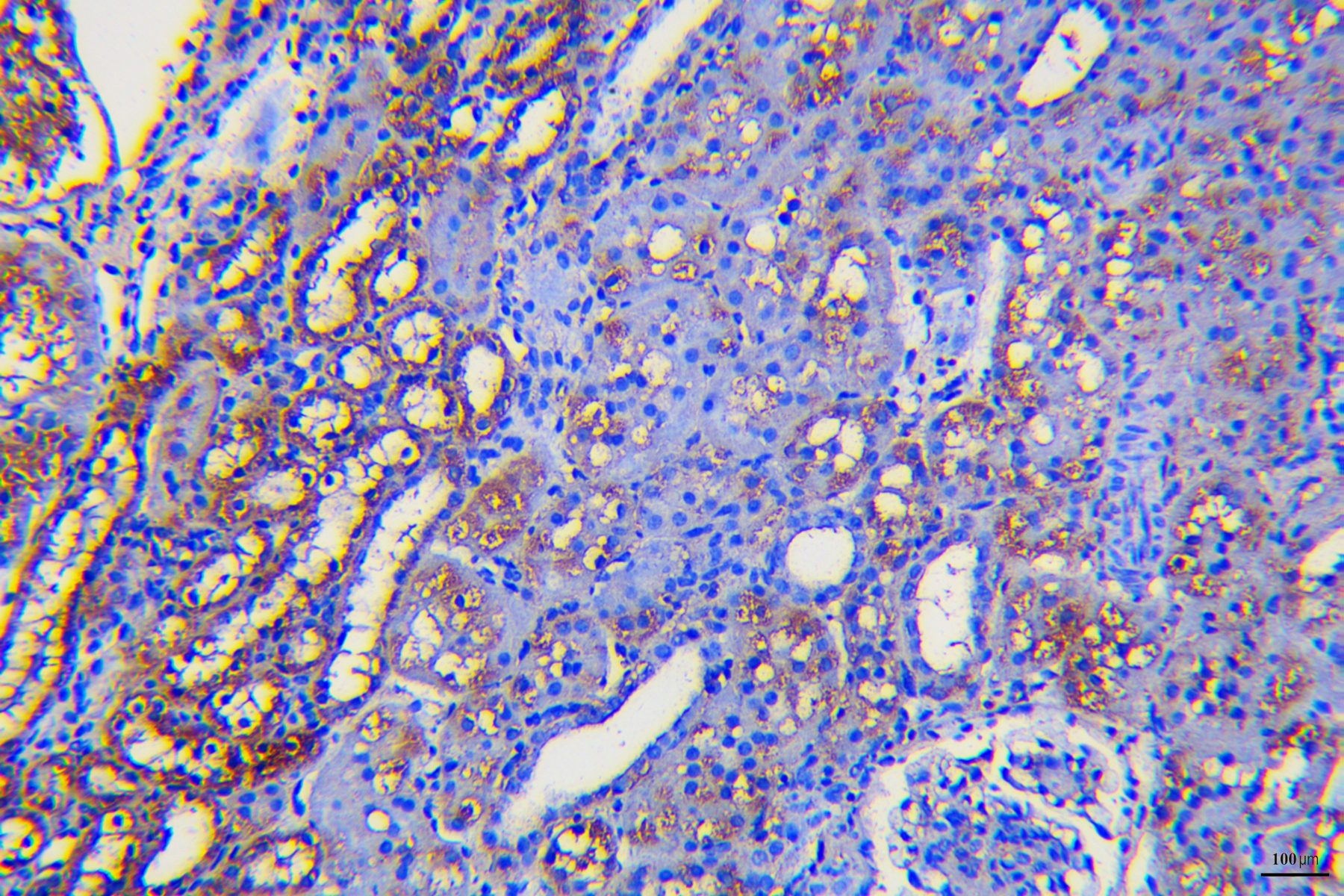


**Figure S1-086. NGAL immunohistochemistry; sample or target: TCA-02**


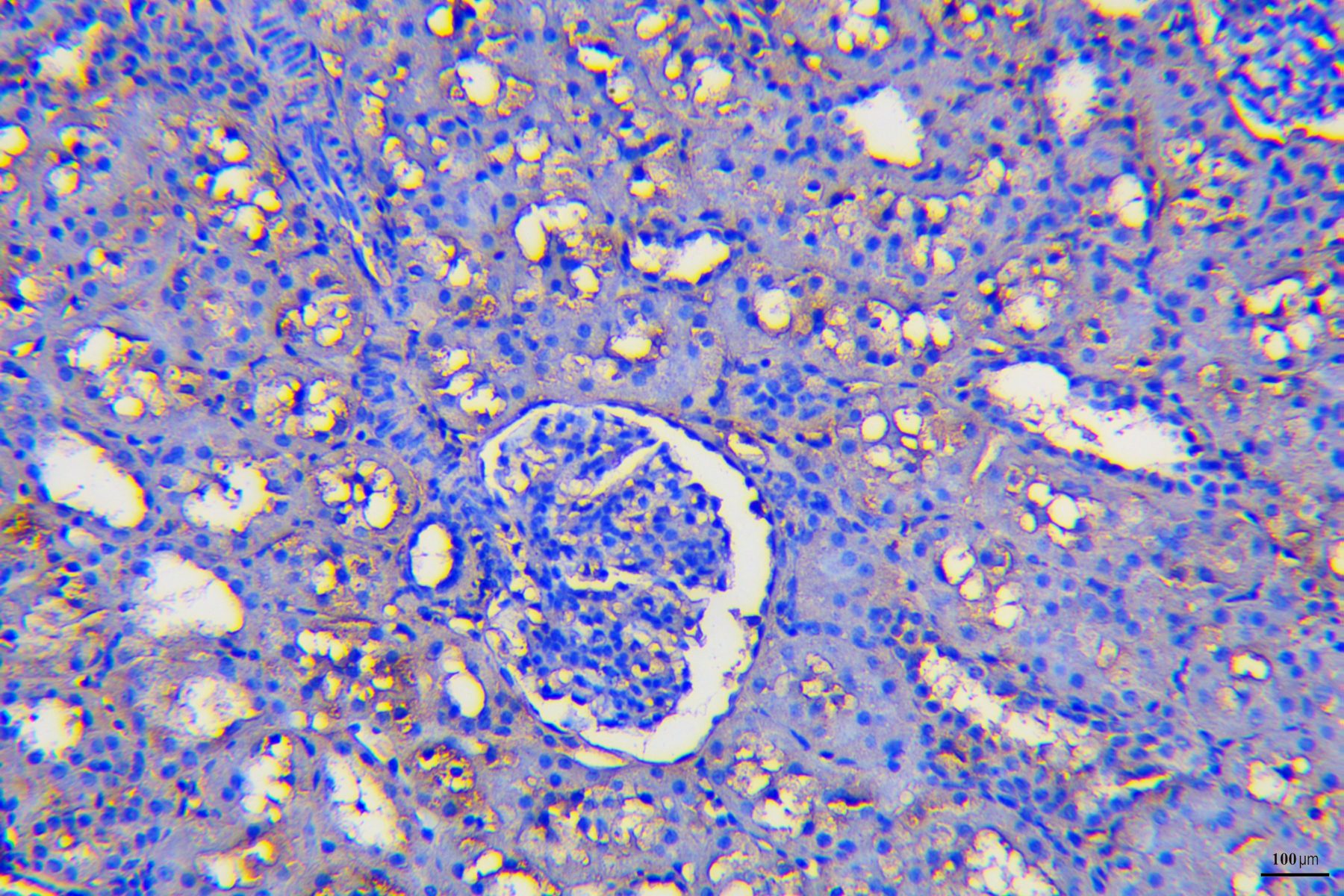


**Figure S1-087. NGAL immunohistochemistry; sample or target: TCA-04**


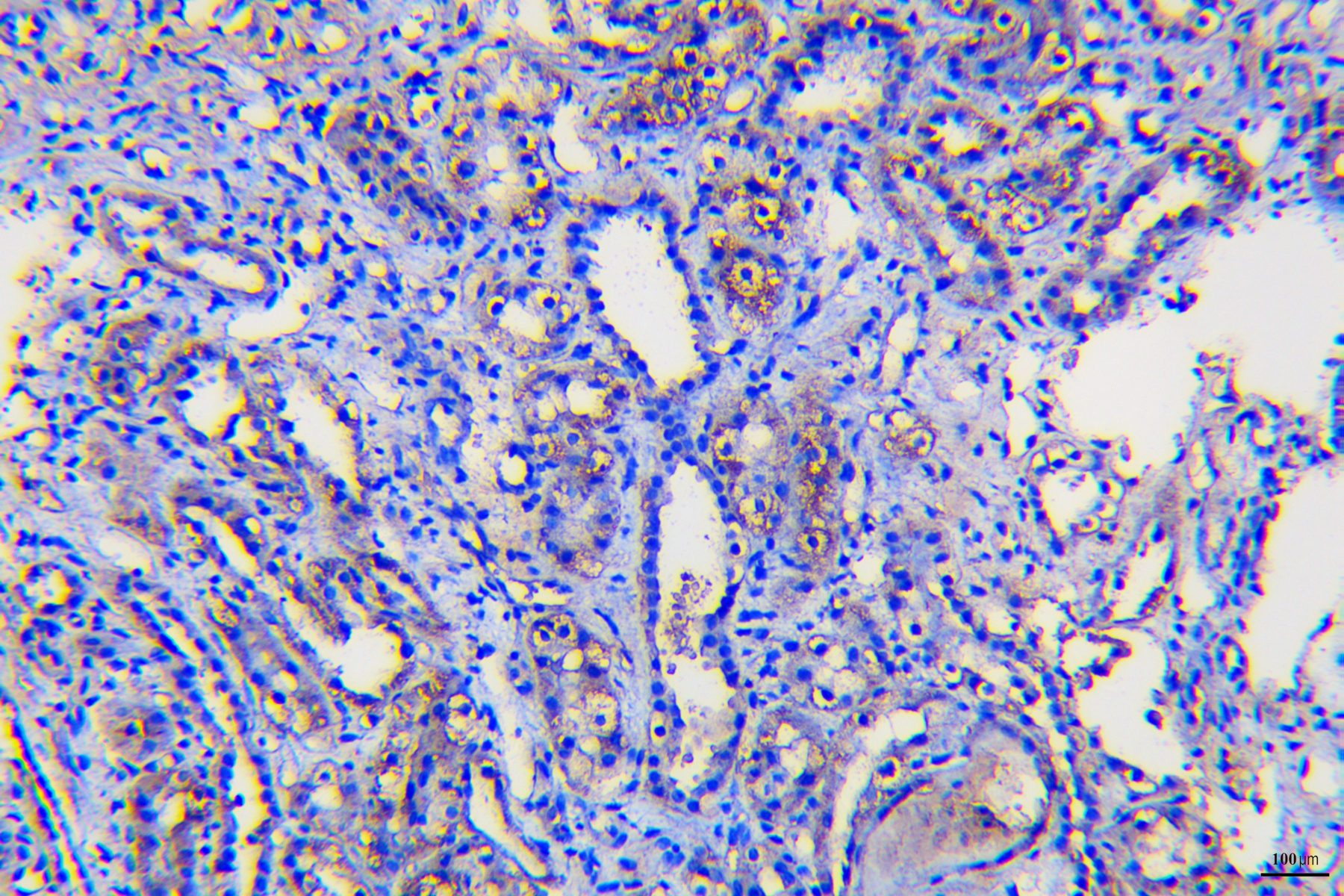


**Figure S1-088. NGAL immunohistochemistry; sample or target: TCA-10**


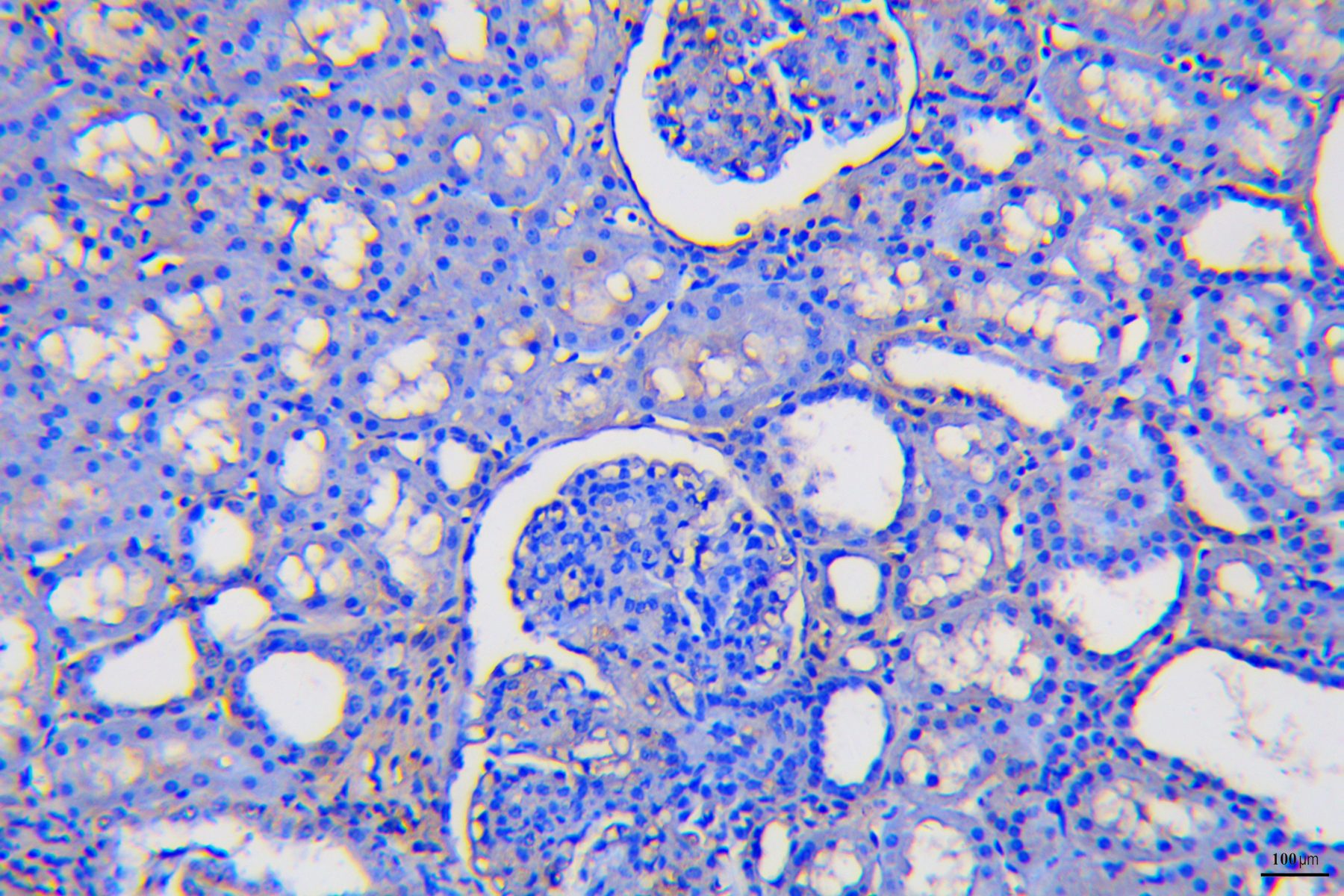


**Figure S1-089. NGAL immunohistochemistry; sample or target: TCA-11**

# Section: TUNEL staining


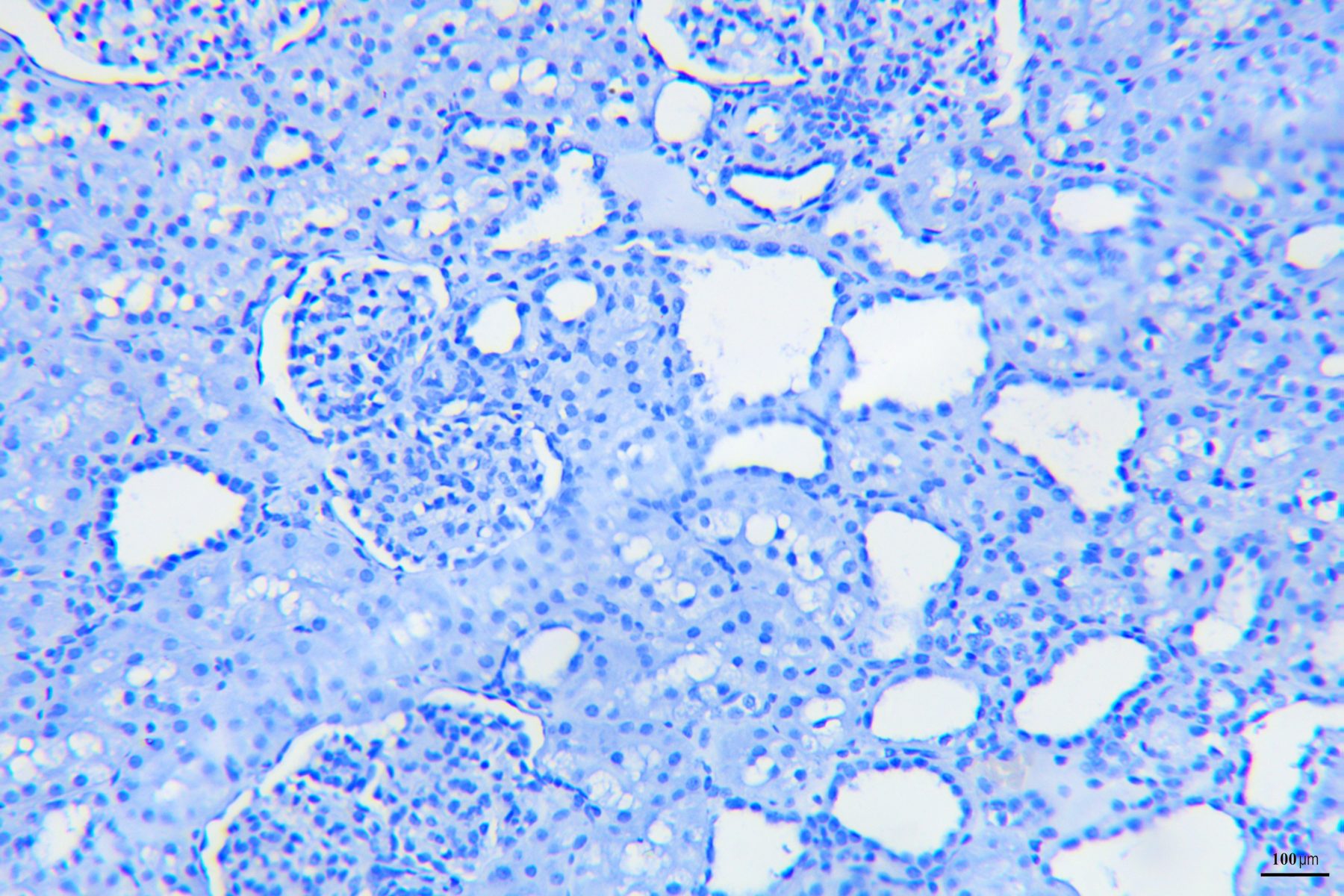


**Figure S1-090. TUNEL apoptosis staining; sample or target: sham-03**


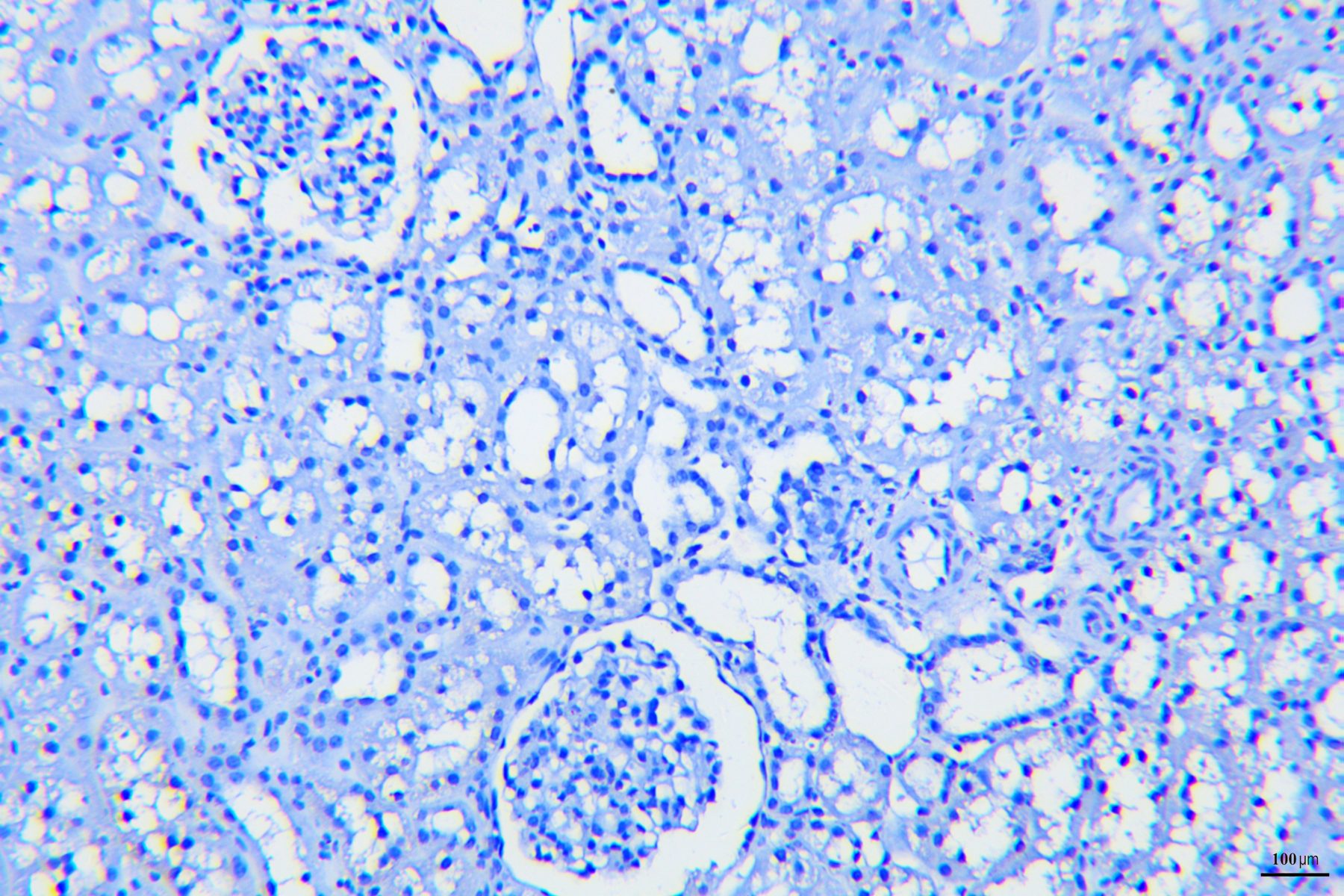


**Figure S1-091. TUNEL apoptosis staining; sample or target: sham-09**


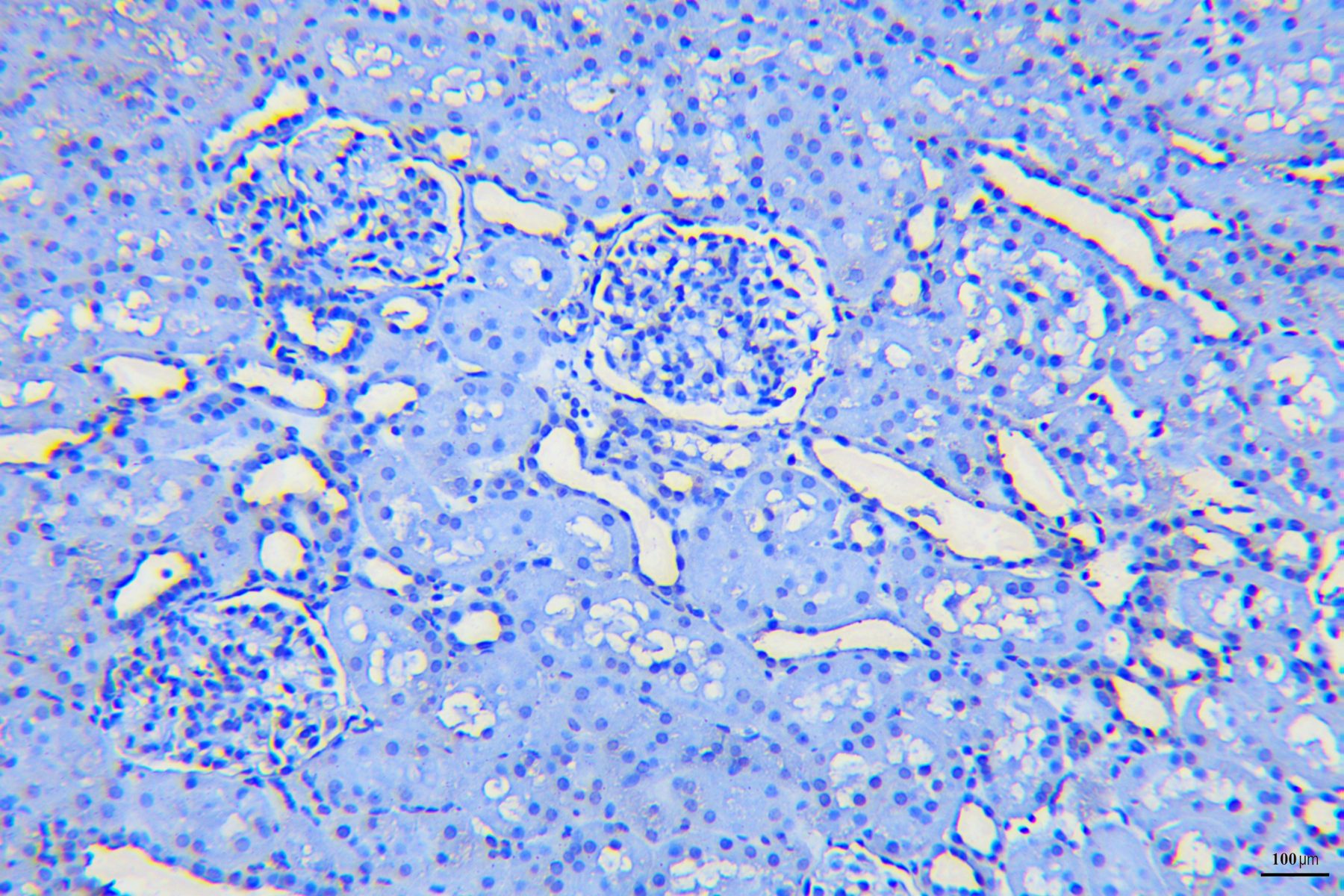


**Figure S1-092. TUNEL apoptosis staining; sample or target: sham-13**


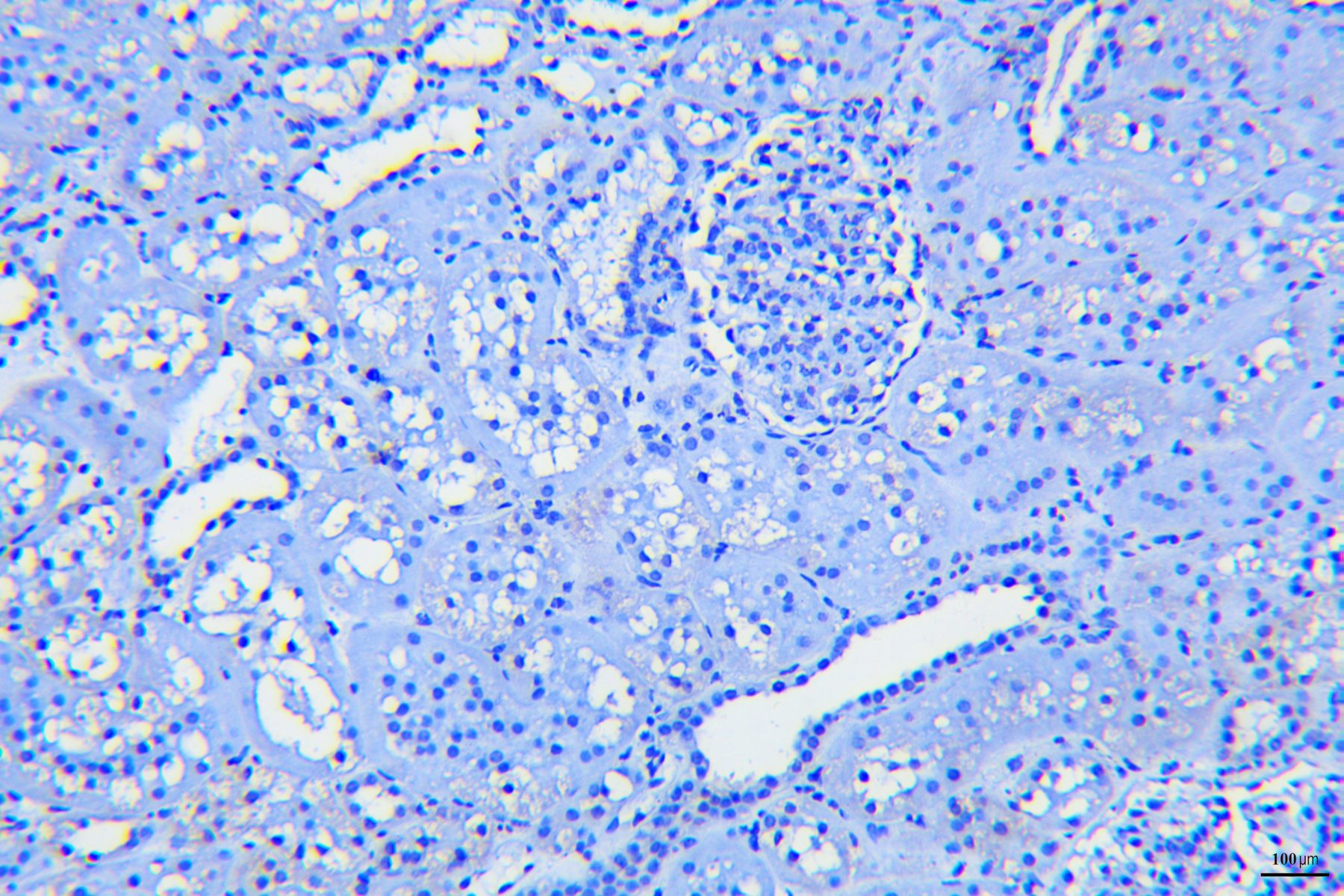


**Figure S1-093. TUNEL apoptosis staining; sample or target: sham-16**


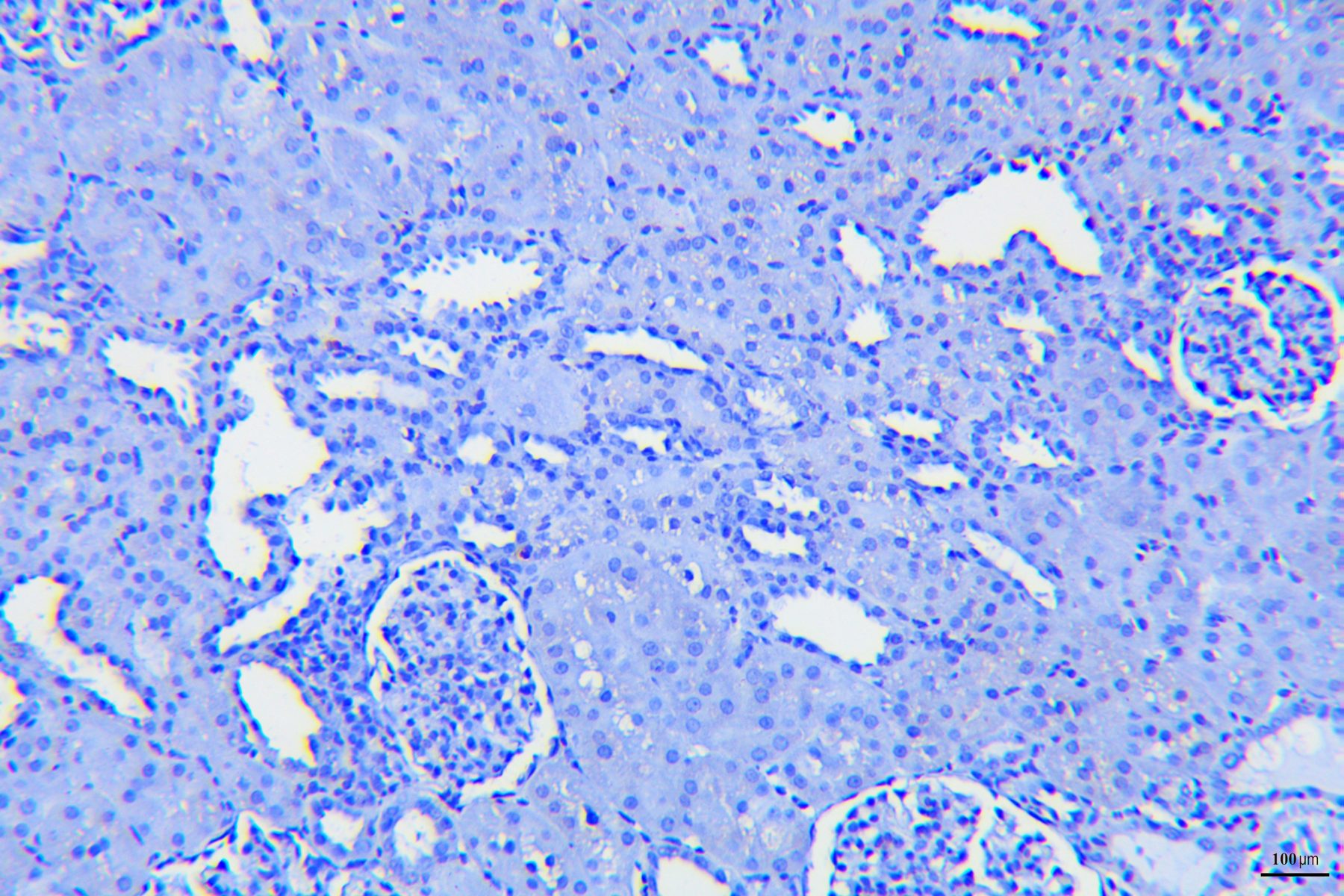


**Figure S1-094. TUNEL apoptosis staining; sample or target: sham-23**


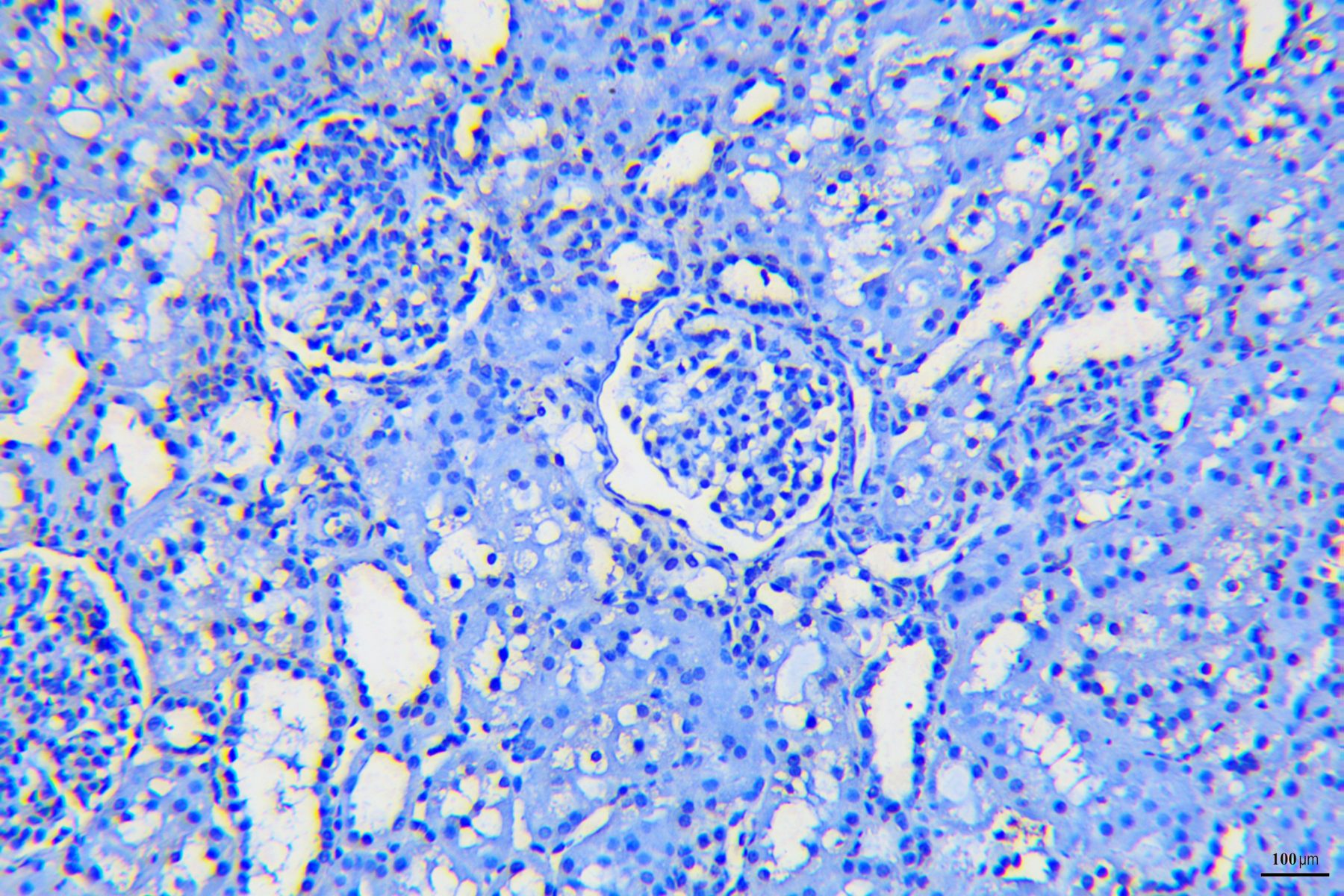


**Figure S1-095. TUNEL apoptosis staining; sample or target: TCA+NBP-05**


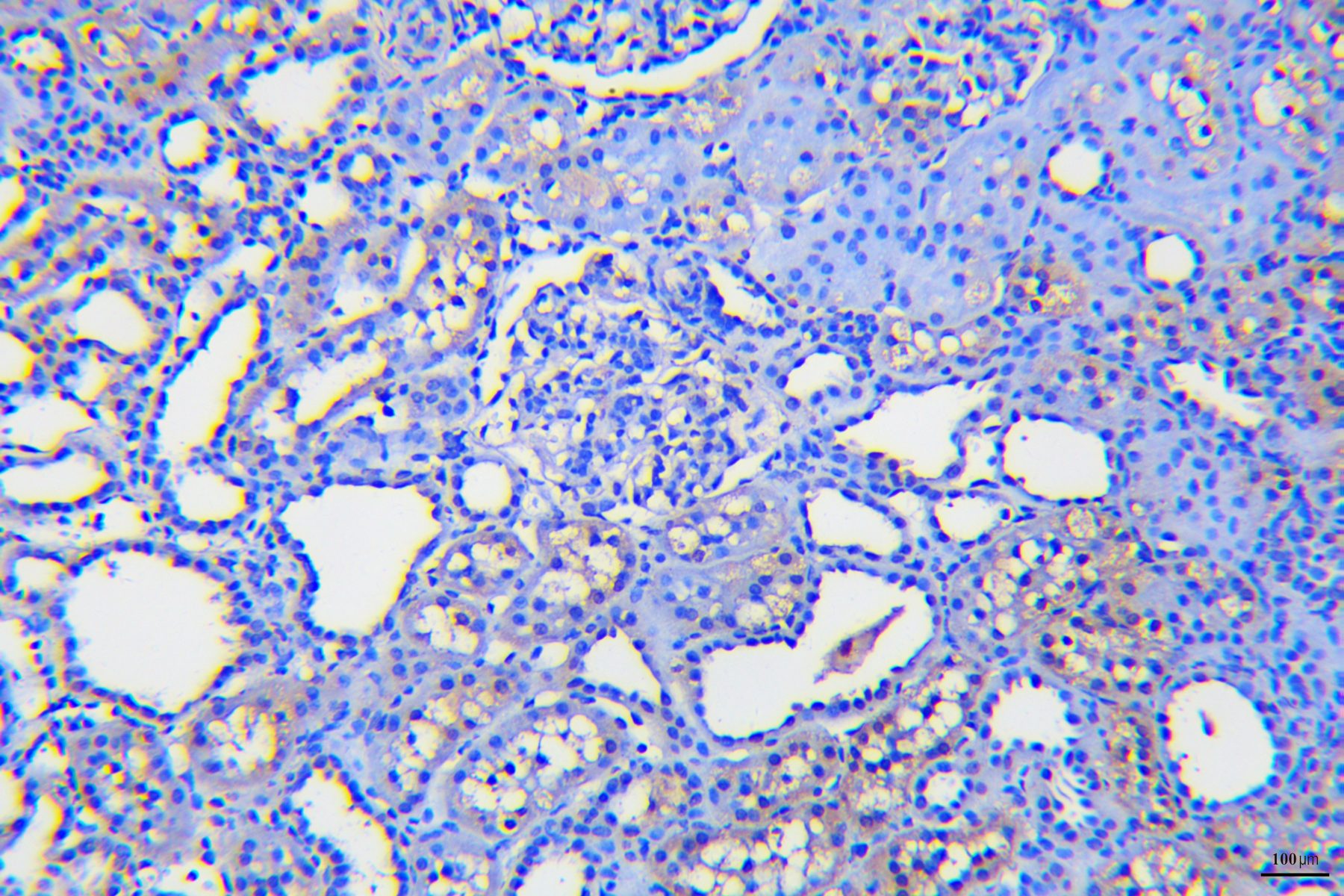


**Figure S1-096. TUNEL apoptosis staining; sample or target: TCA+NBP-07**


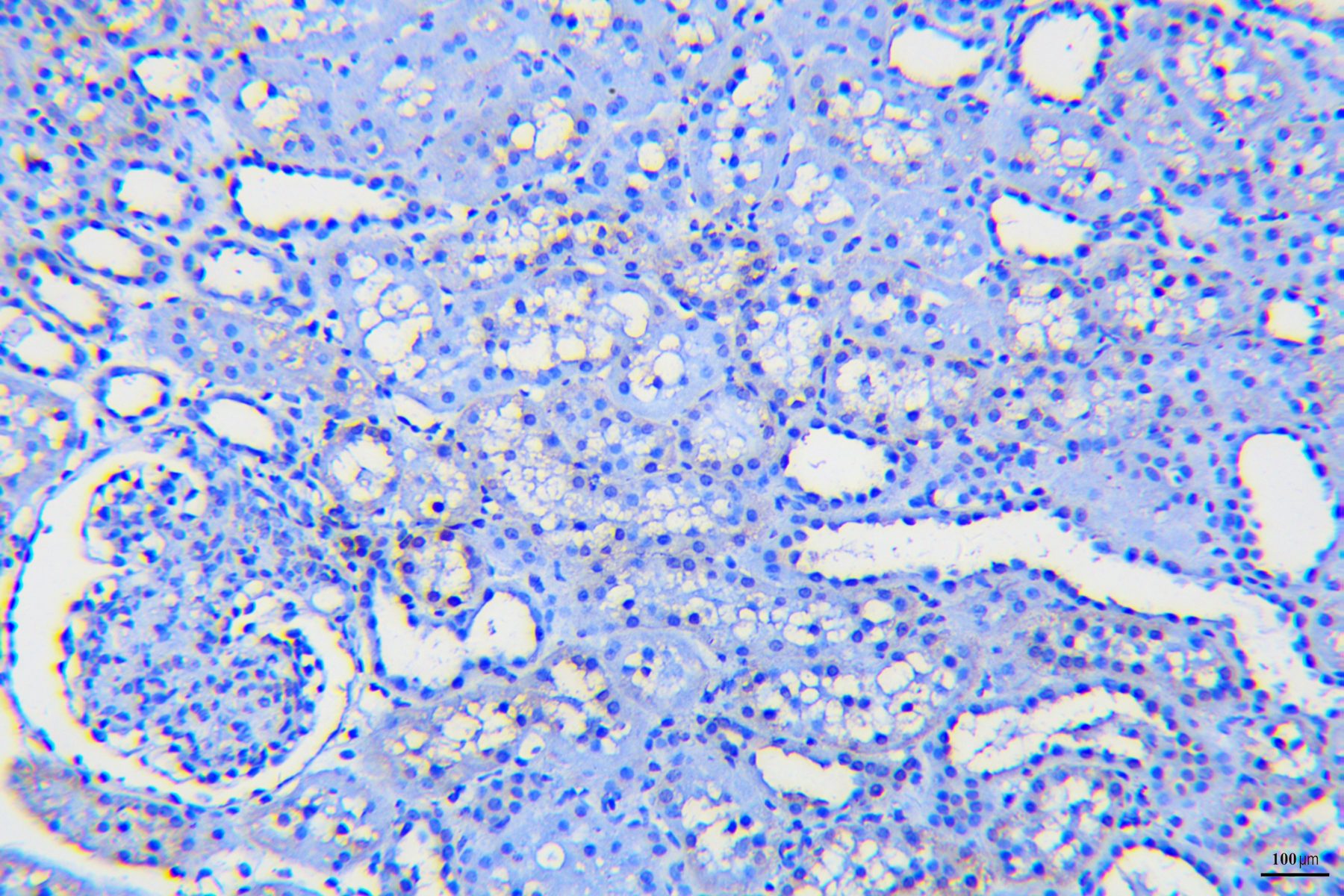


**Figure S1-097. TUNEL apoptosis staining; sample or target: TCA+NBP-17**


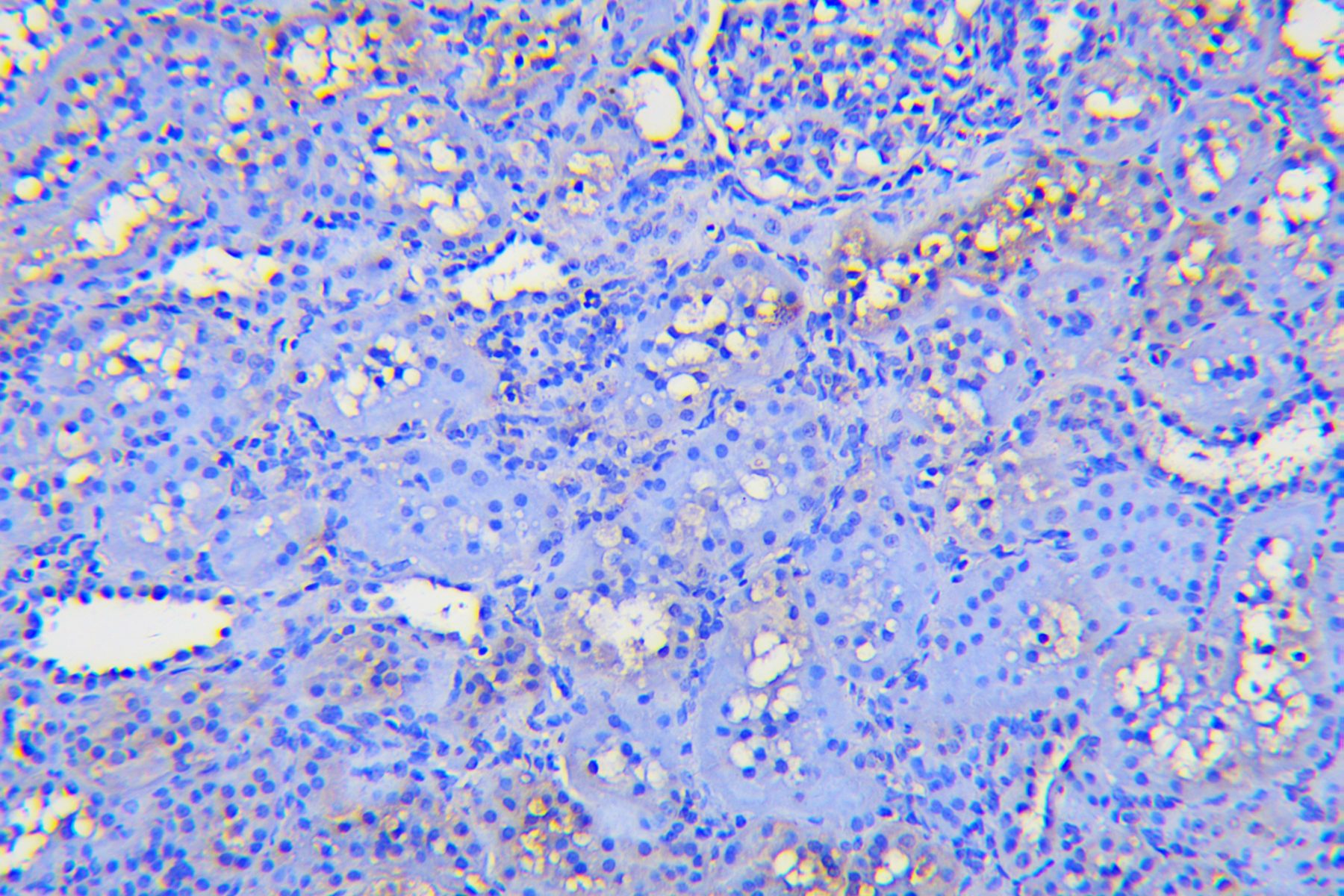


**Figure S1-098. TUNEL apoptosis staining; sample or target: TCA+NBP-20**


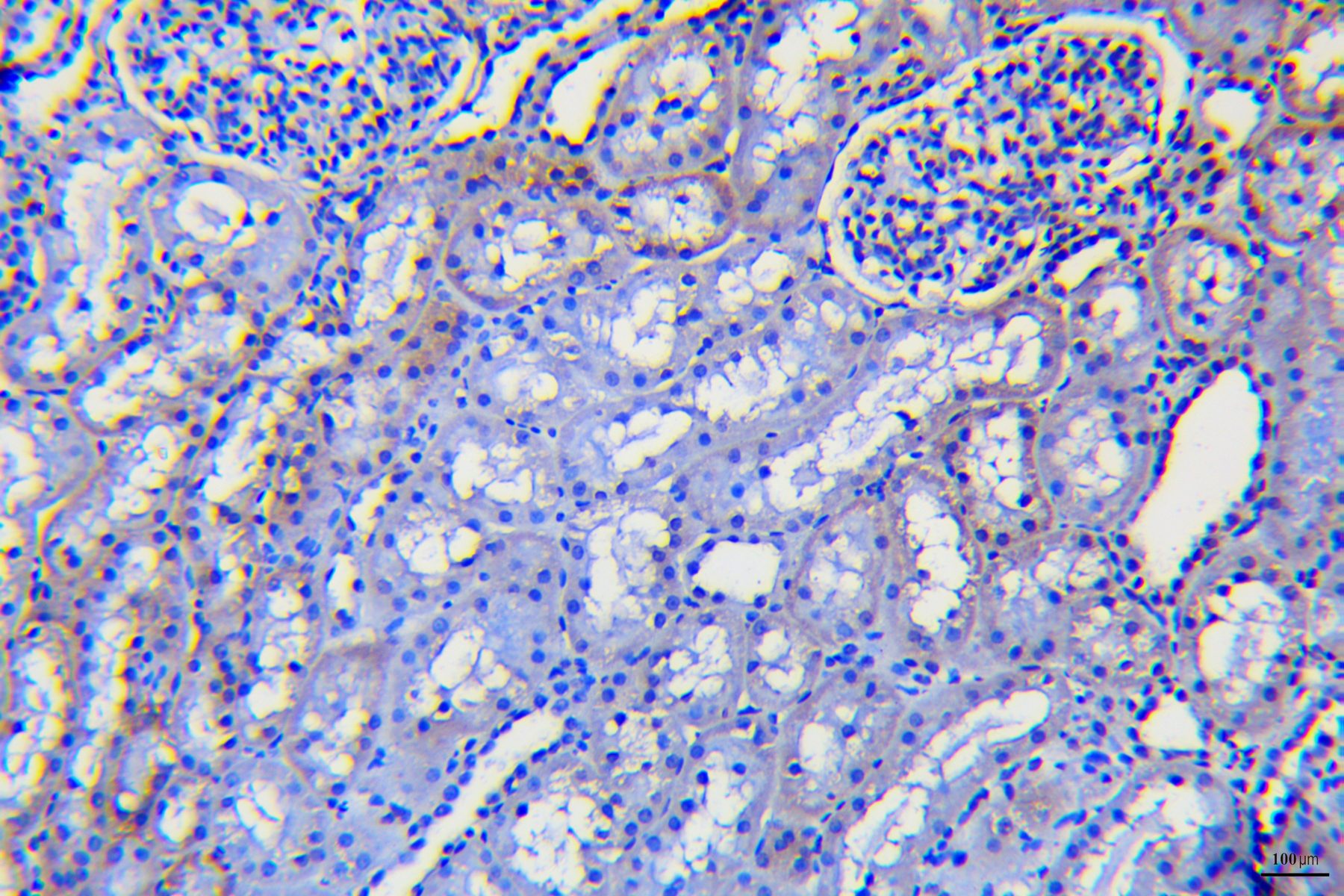


**Figure S1-099. TUNEL apoptosis staining; sample or target: TCA+NBP-21**


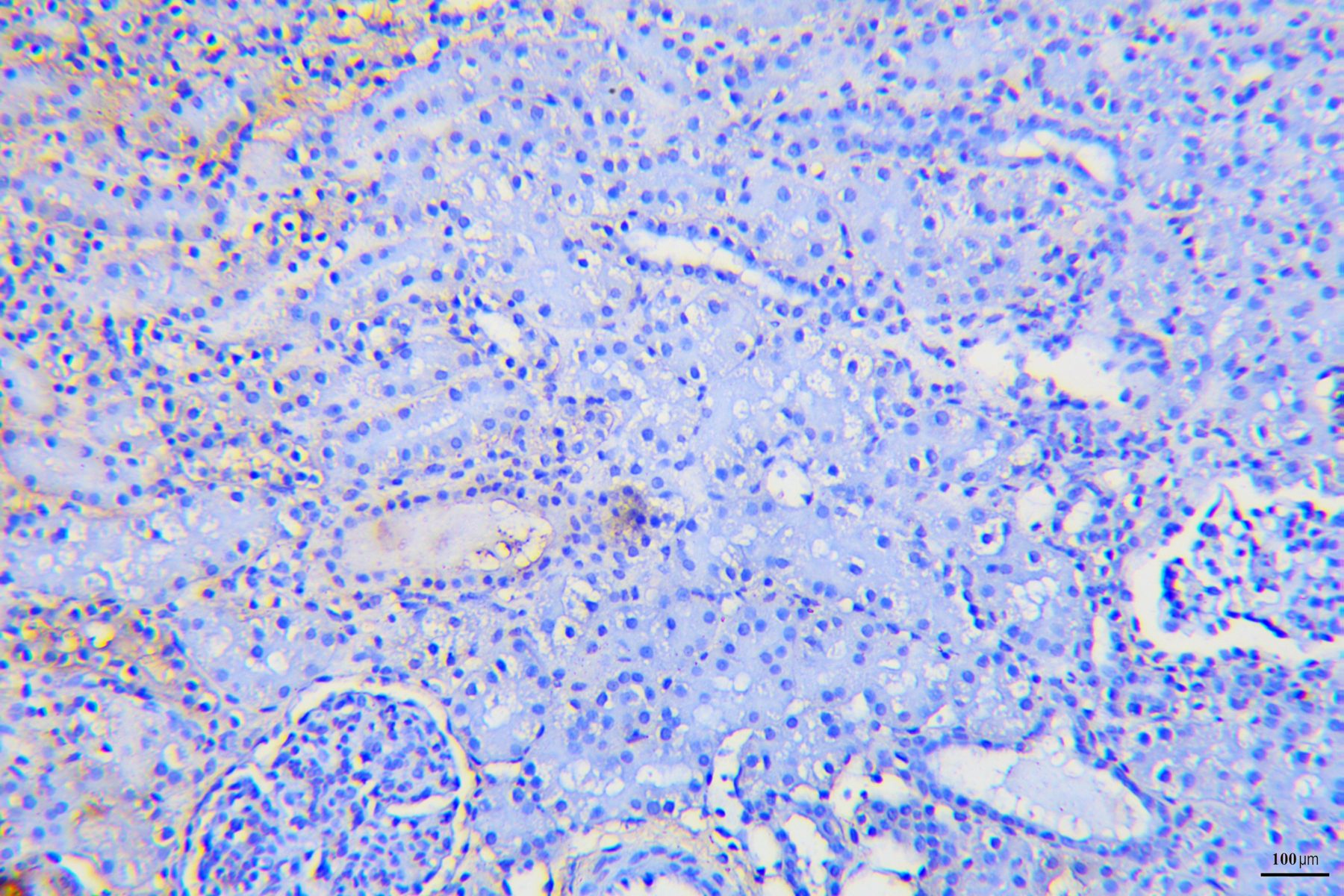


**Figure S1-100. TUNEL apoptosis staining; sample or target: TCA+NBP-22**


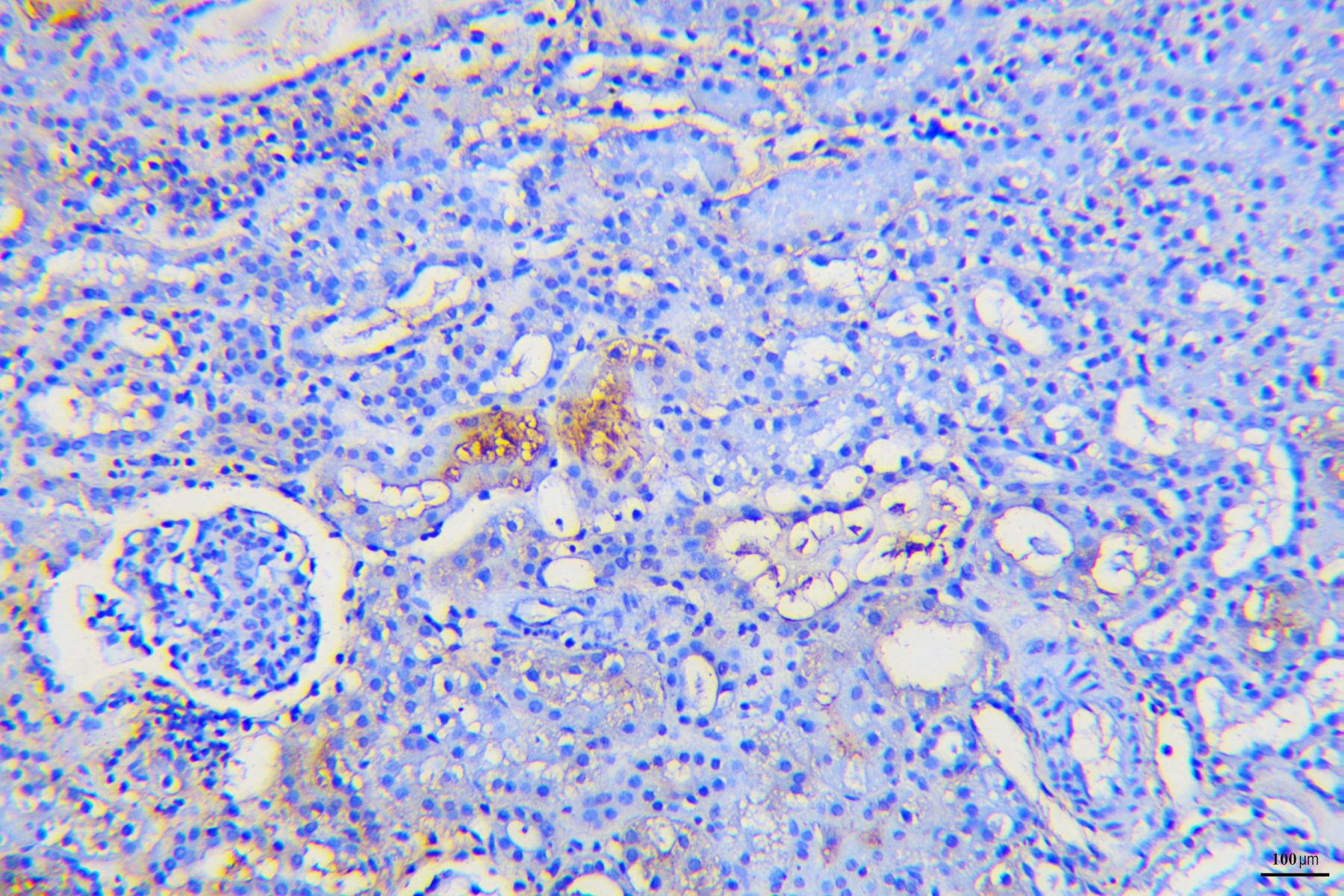


**Figure S1-101. TUNEL apoptosis staining; sample or target: TCA+NBP-26**


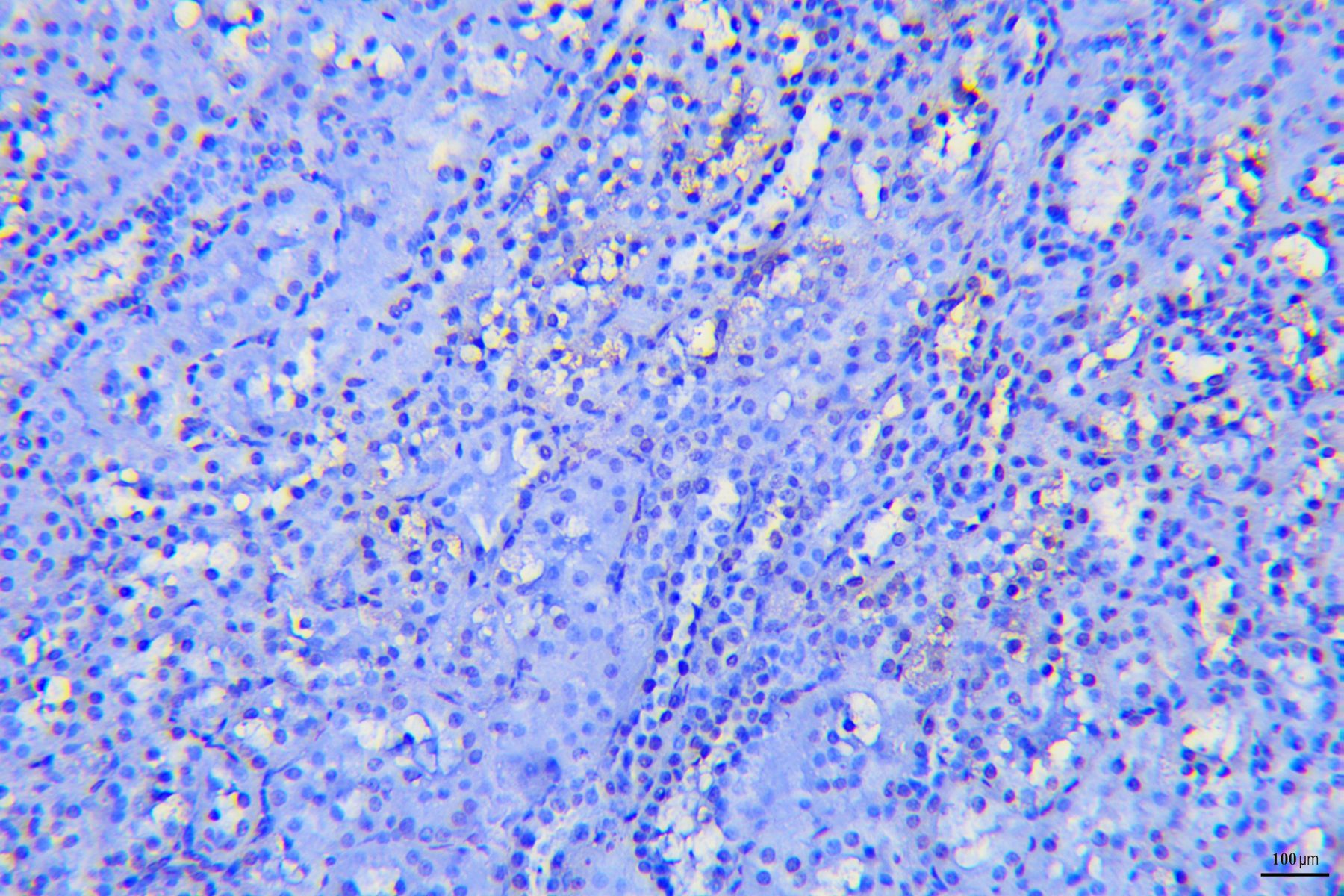


**Figure S1-102. TUNEL apoptosis staining; sample or target: TCA-01**


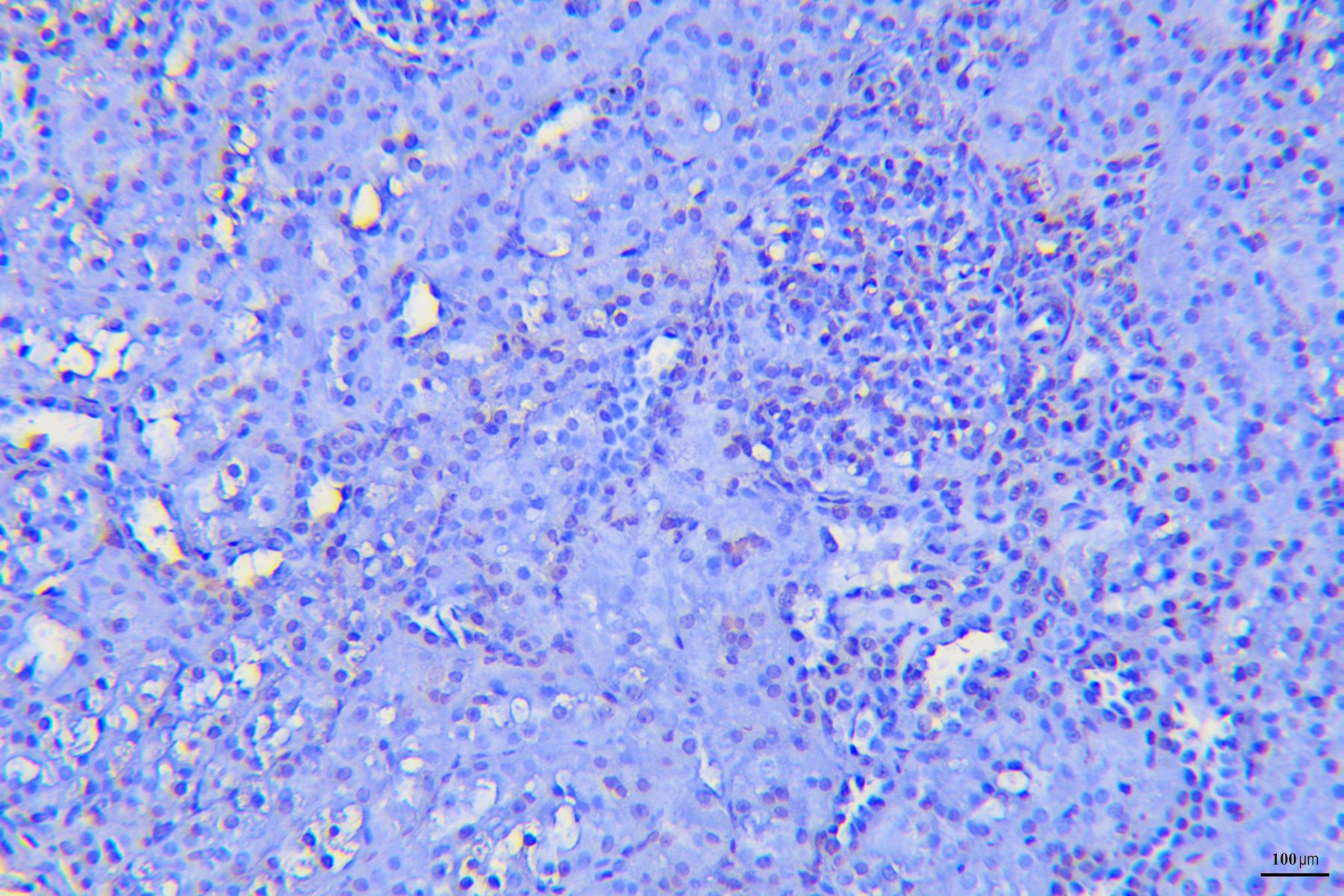


**Figure S1-103. TUNEL apoptosis staining; sample or target: TCA-02**


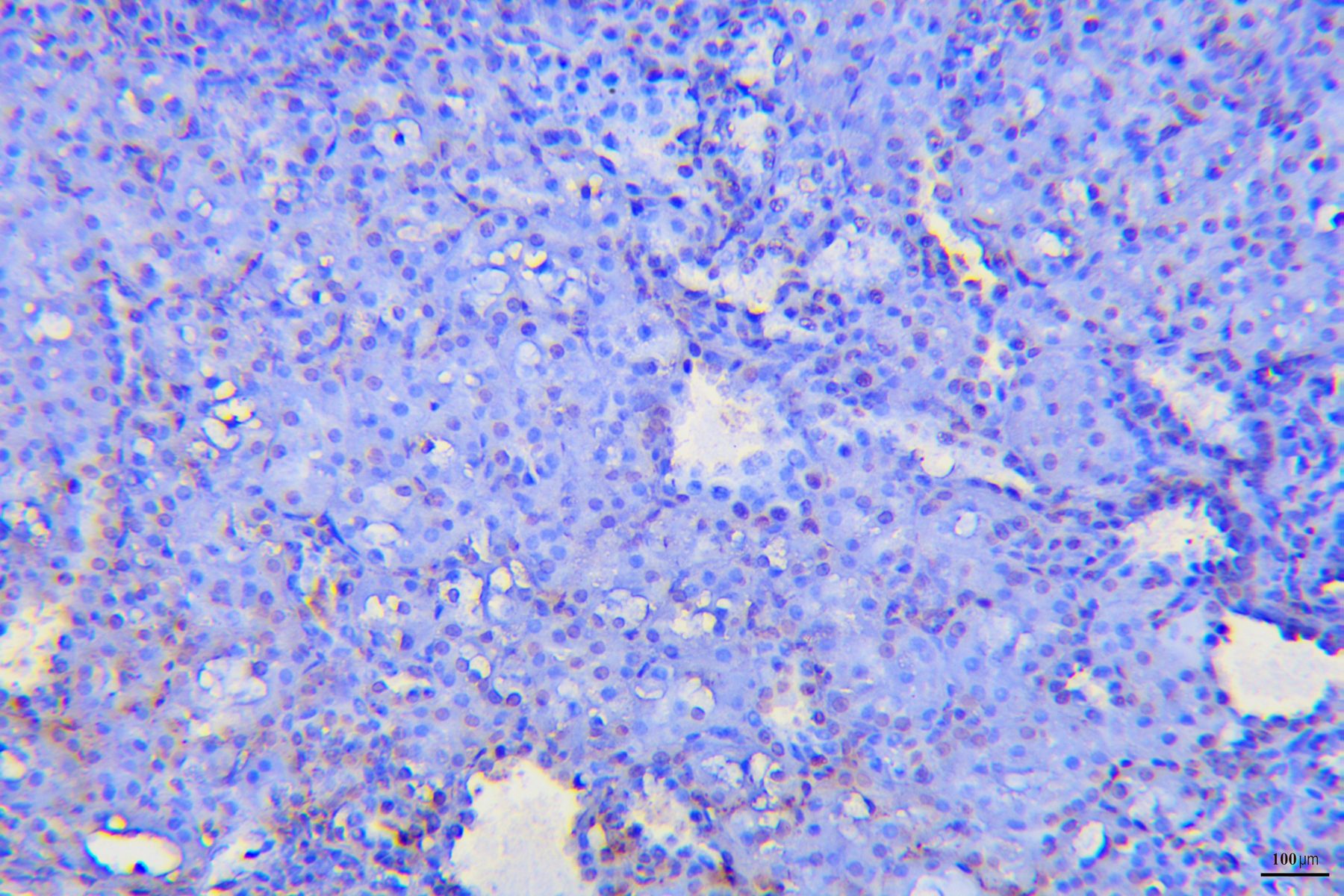


**Figure S1-104. TUNEL apoptosis staining; sample or target: TCA-04**


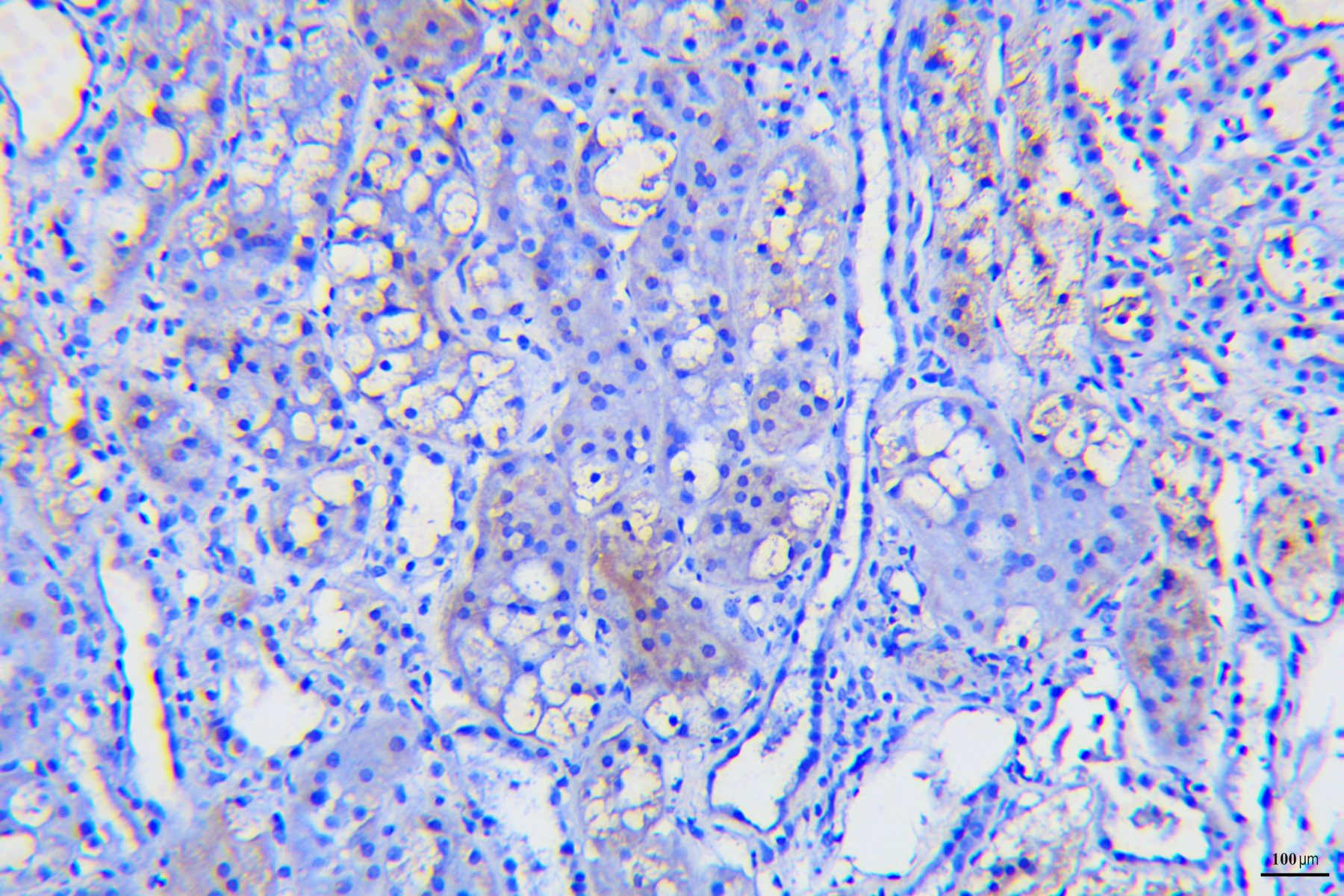


**Figure S1-105. TUNEL apoptosis staining; sample or target: TCA-10**


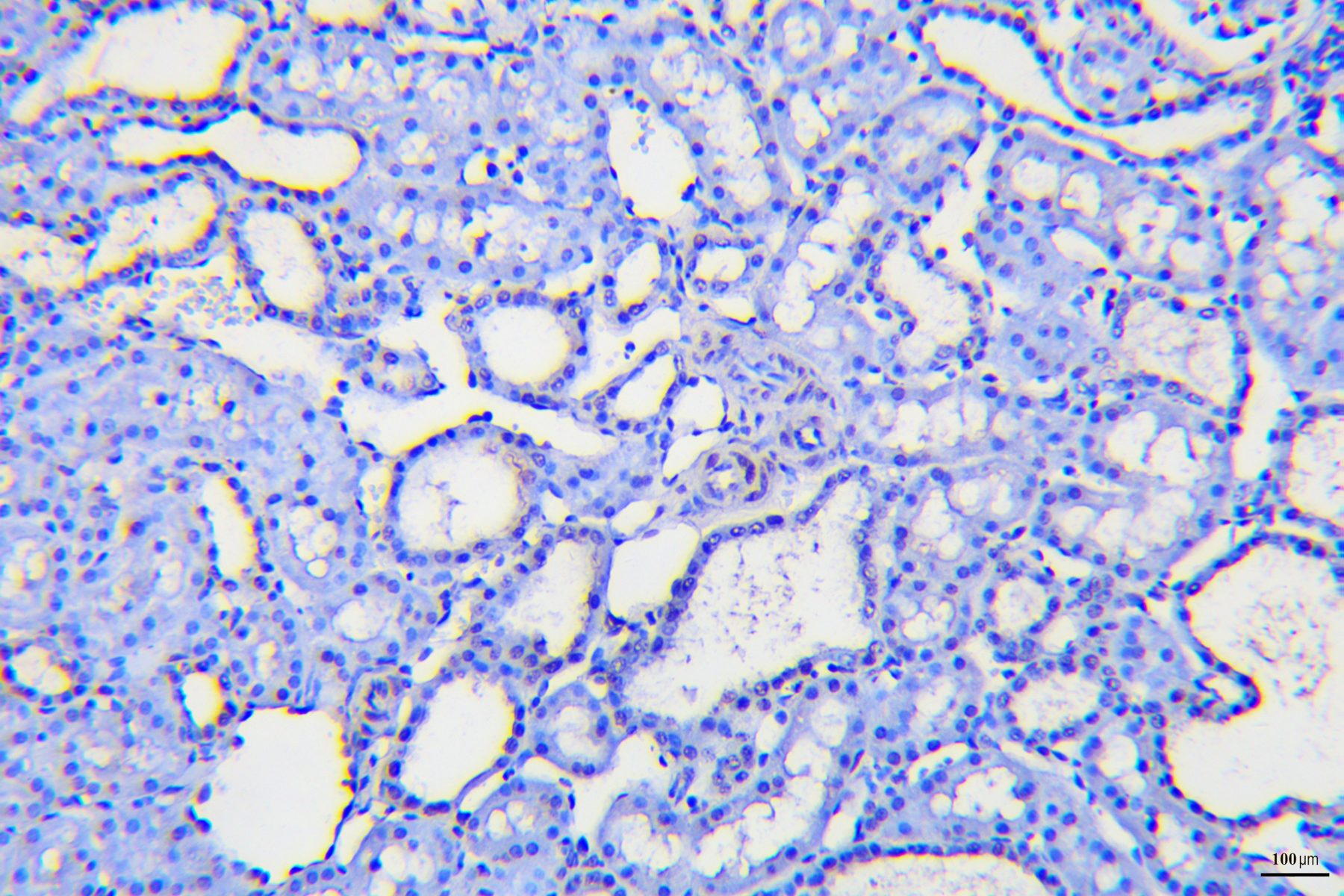


**Figure S1-106. TUNEL apoptosis staining; sample or target: TCA-11**
