# Supplementary material for: Integrated transcriptomics identifies ER stress–associated apoptosis in post-resuscitation AKI and supports early Dl-3-n-butylphthalide–associated renoprotection in a porcine TCA model
Source: Front Pharmacol. 2026 Jun 4;17:1841271. doi: 10.3389/fphar.2026.1841271 (PMC13275486; doi:10.3389/fphar.2026.1841271)
Supplement: Supplementary file 3 [file Supplementaryfile3.docx]

**Supplementary File 3: Single-cell Transcriptome Figures**

# Section: step01_QC


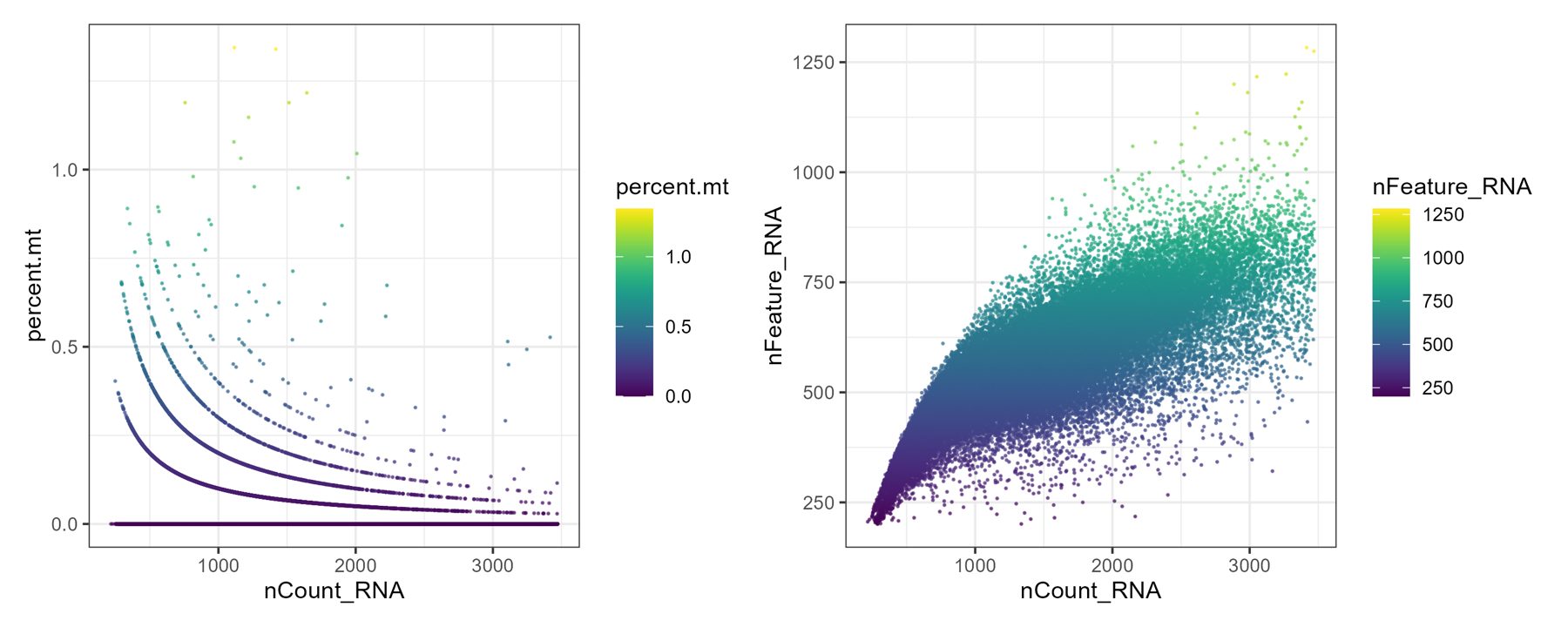


**Figure S3-001. Single-cell transcriptome analysis, step01_QC: 01 QC scatter after**


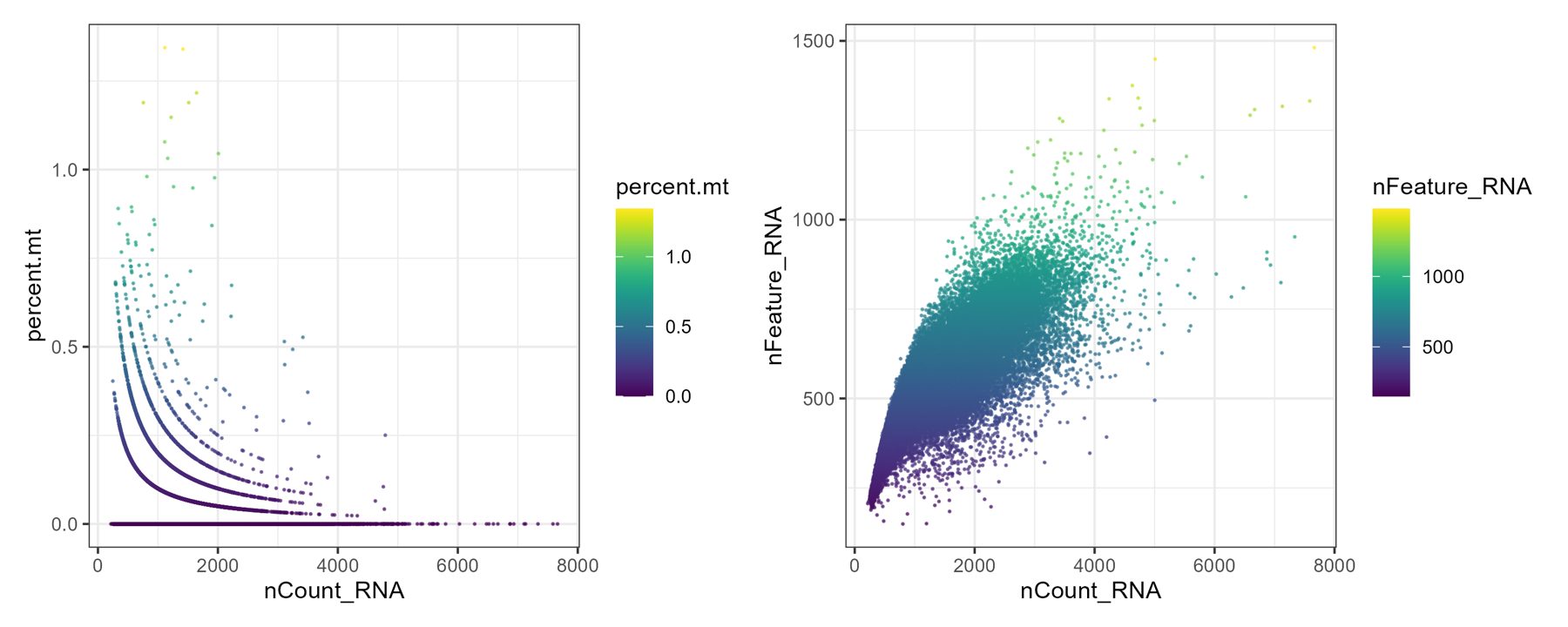


**Figure S3-002. Single-cell transcriptome analysis, step01_QC: 01 QC scatter before**


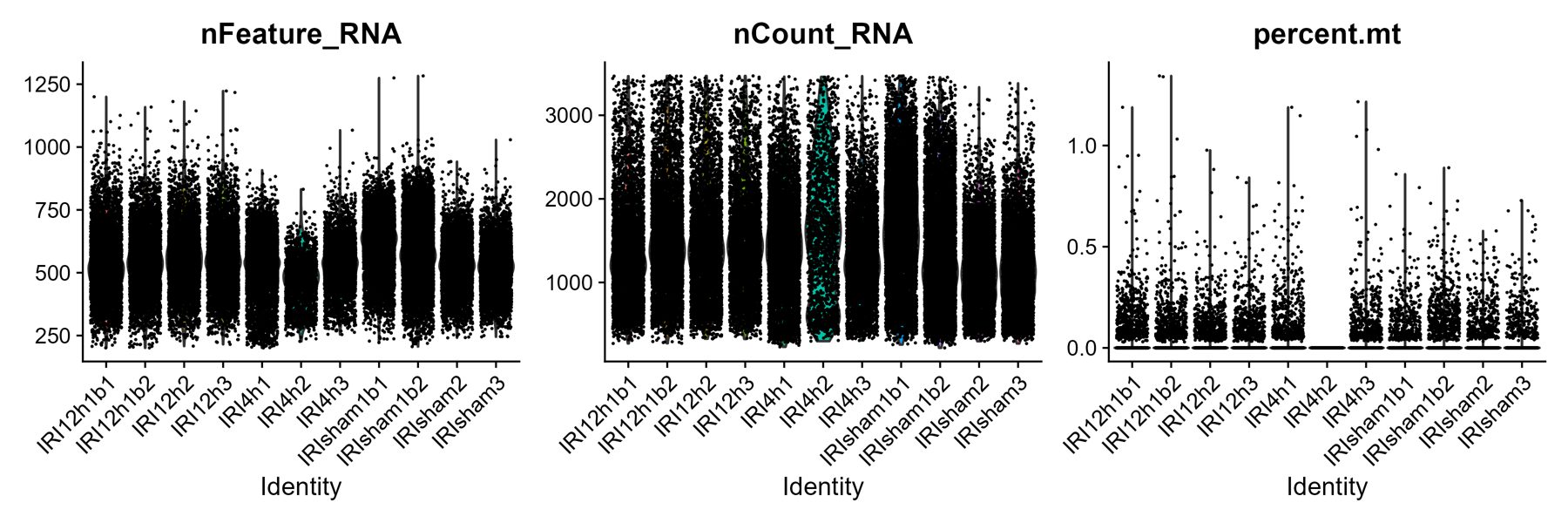


**Figure S3-003. Single-cell transcriptome analysis, step01_QC: 01 QC violin after by sample**


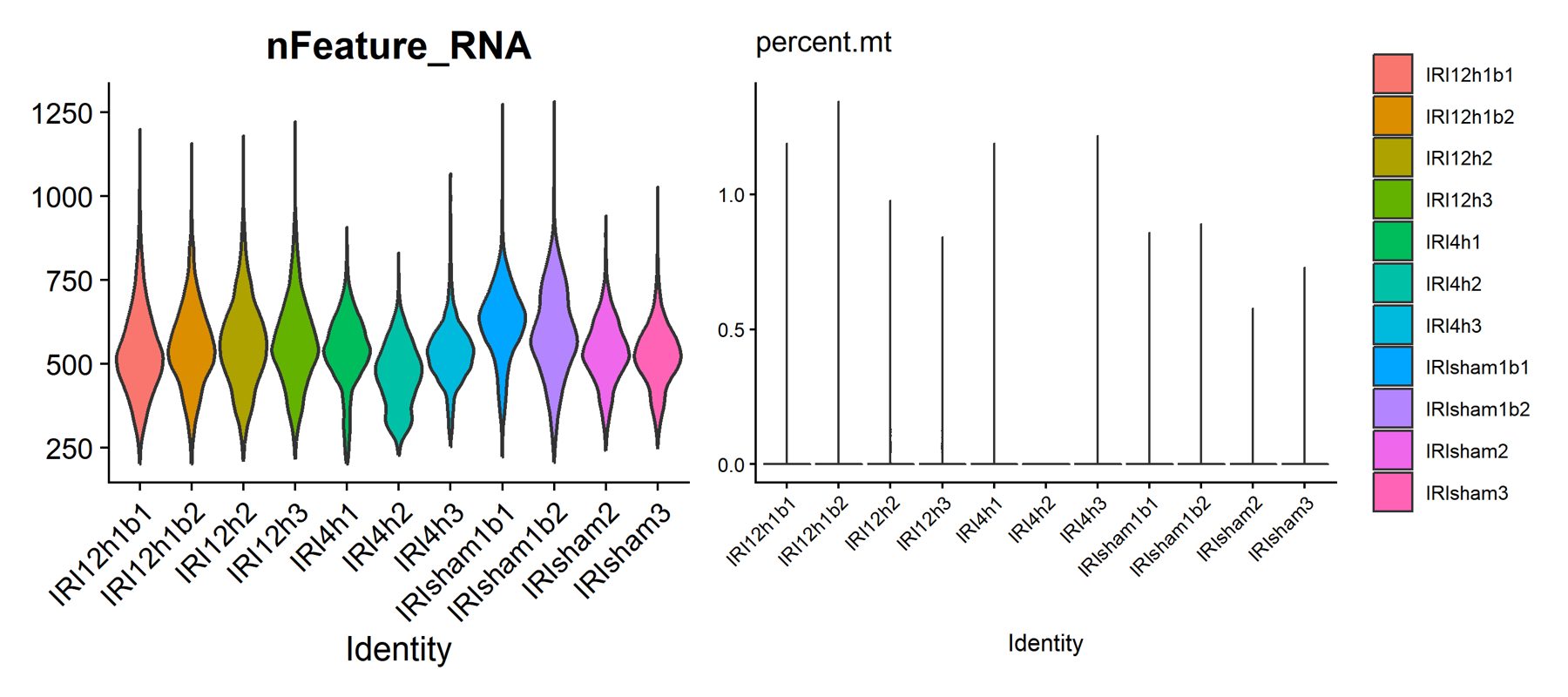


**Figure S3-004. Single-cell transcriptome analysis, step01_QC: 01 QC violin after by sample subset**


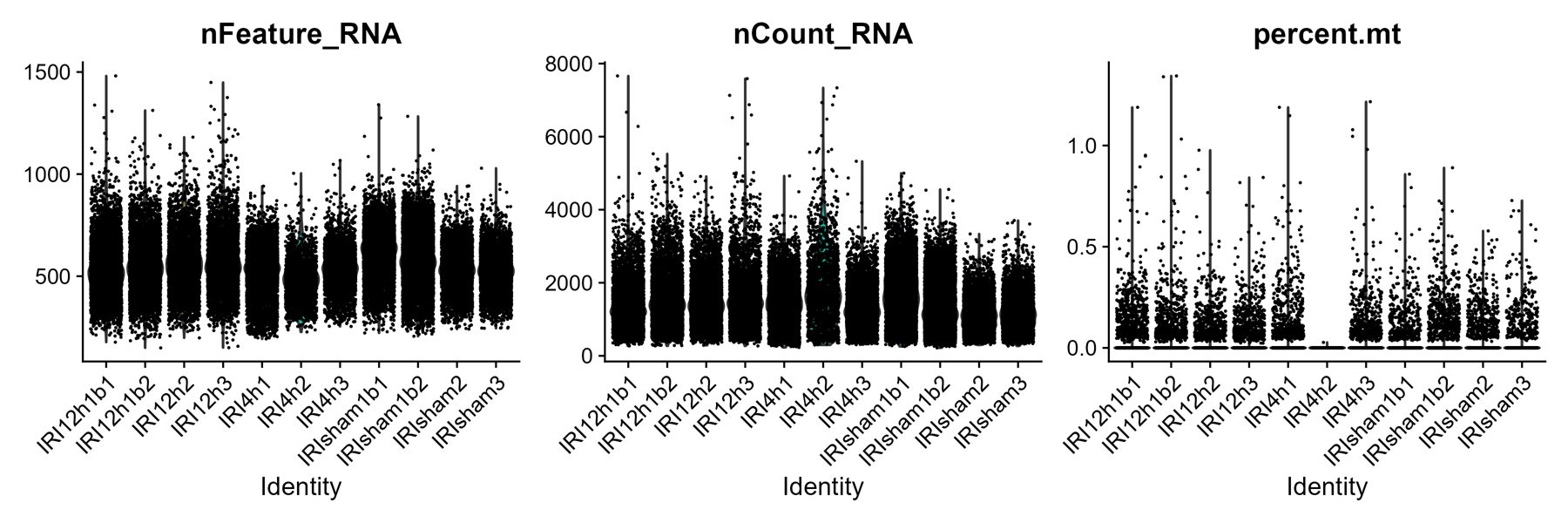


**Figure S3-005. Single-cell transcriptome analysis, step01_QC: 01 QC violin before by sample**


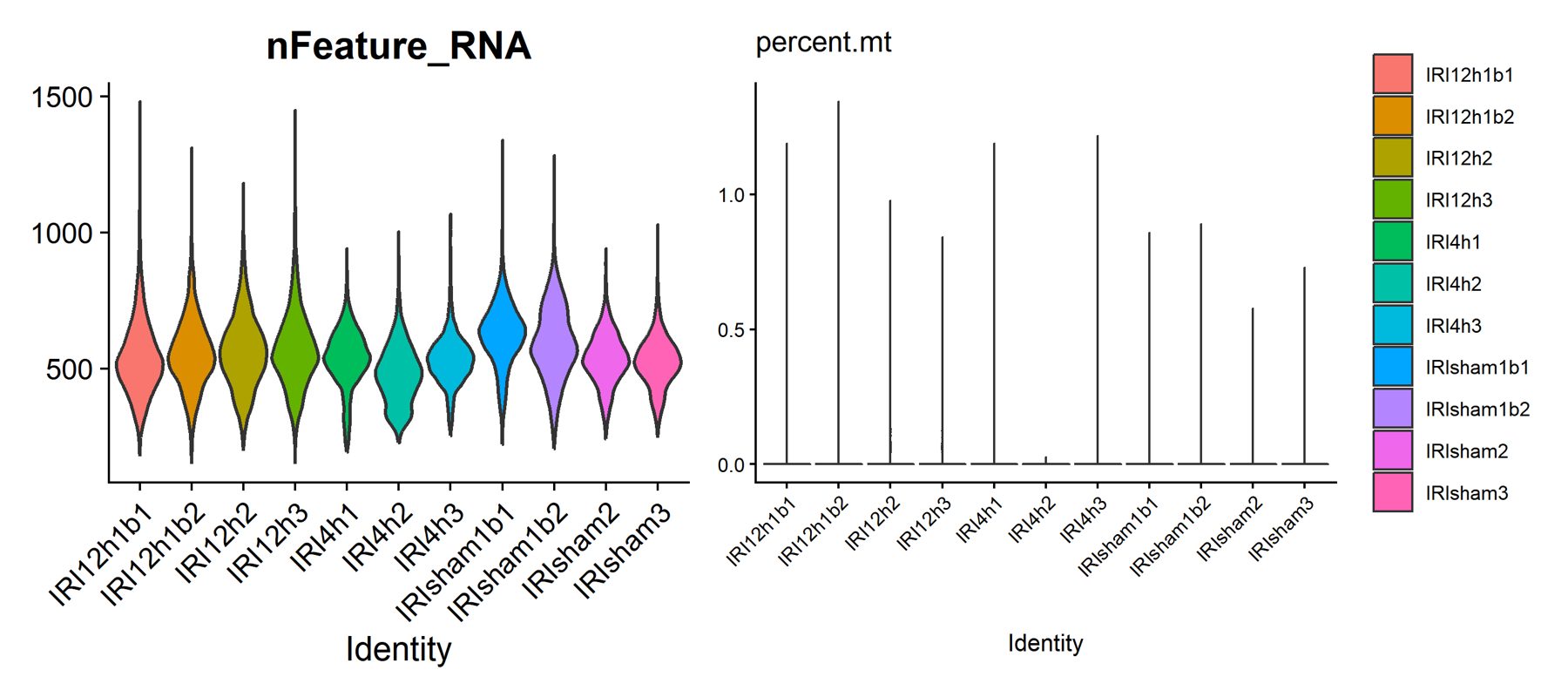


**Figure S3-006. Single-cell transcriptome analysis, step01_QC: 01 QC violin before by sample subset**

# Section: step02_Doublet


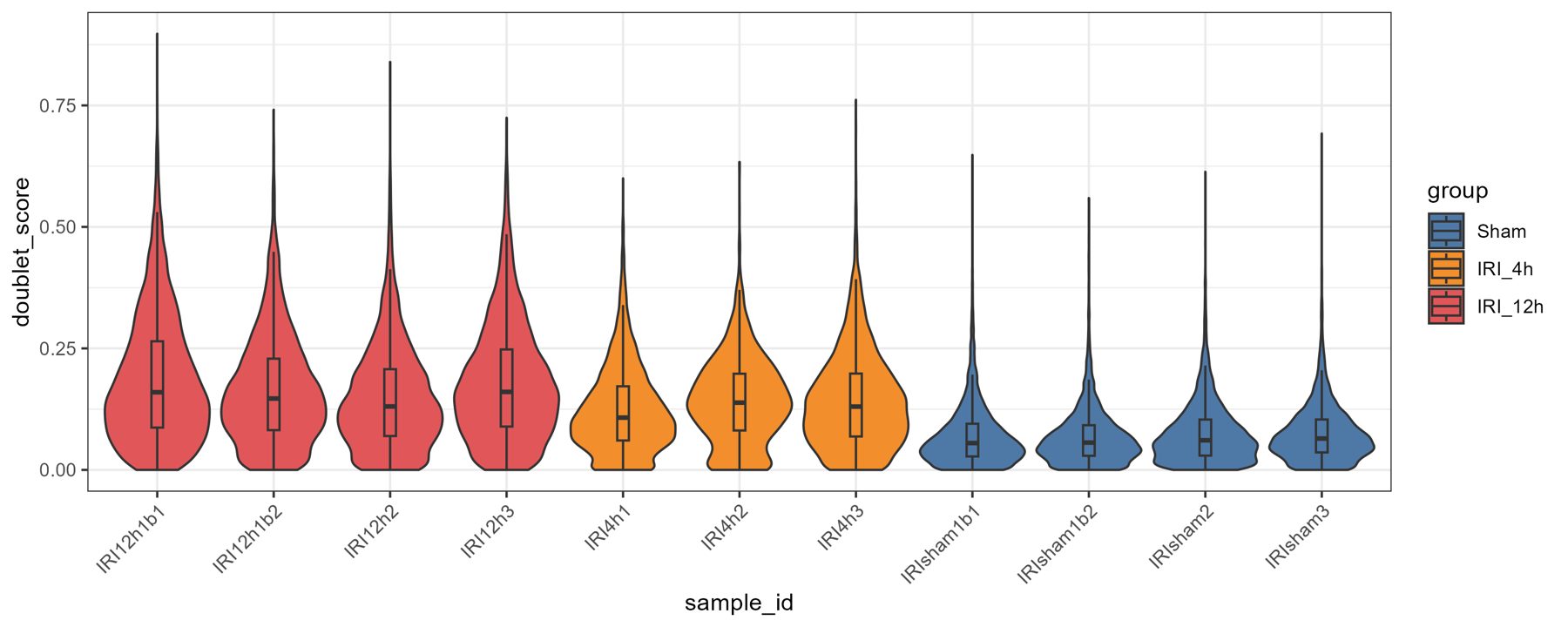


**Figure S3-007. Single-cell transcriptome analysis, step02_Doublet: 02 doublet score by sample**


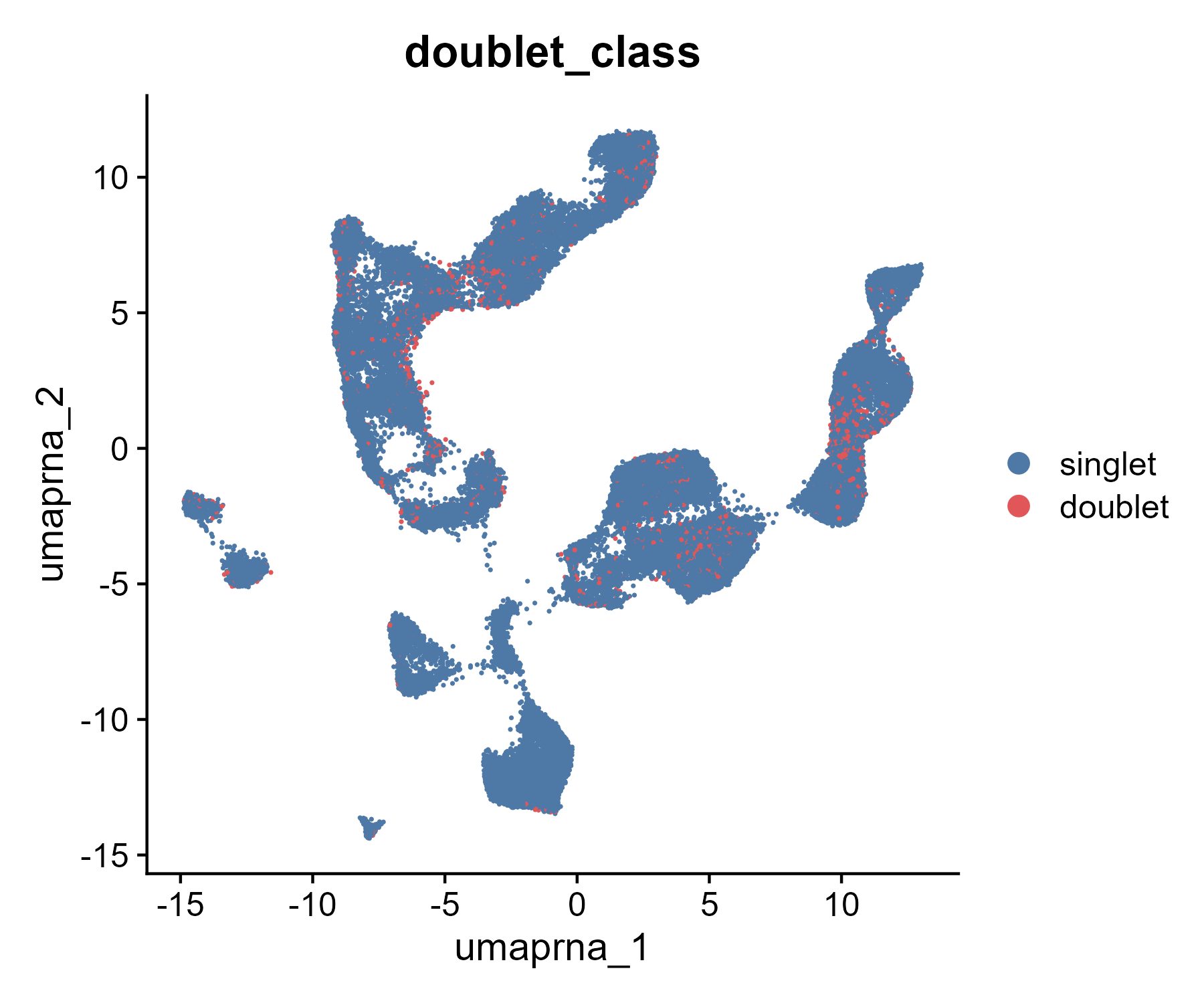


**Figure S3-008. Single-cell transcriptome analysis, step02_Doublet: 02 umap doublet overlay**

# Section: step03_Integration


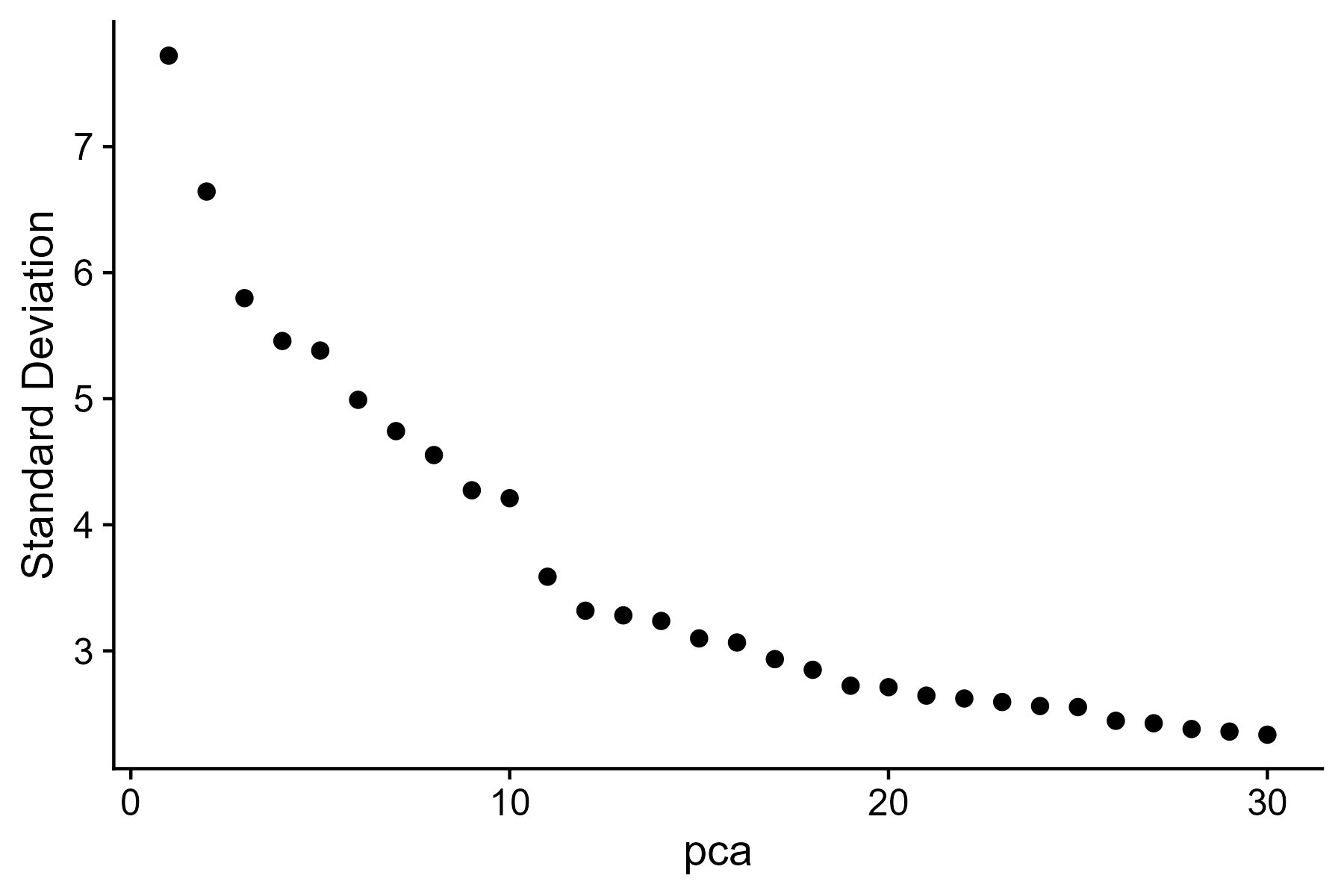


**Figure S3-009. Single-cell transcriptome analysis, step03_Integration: 03 elbowplot**


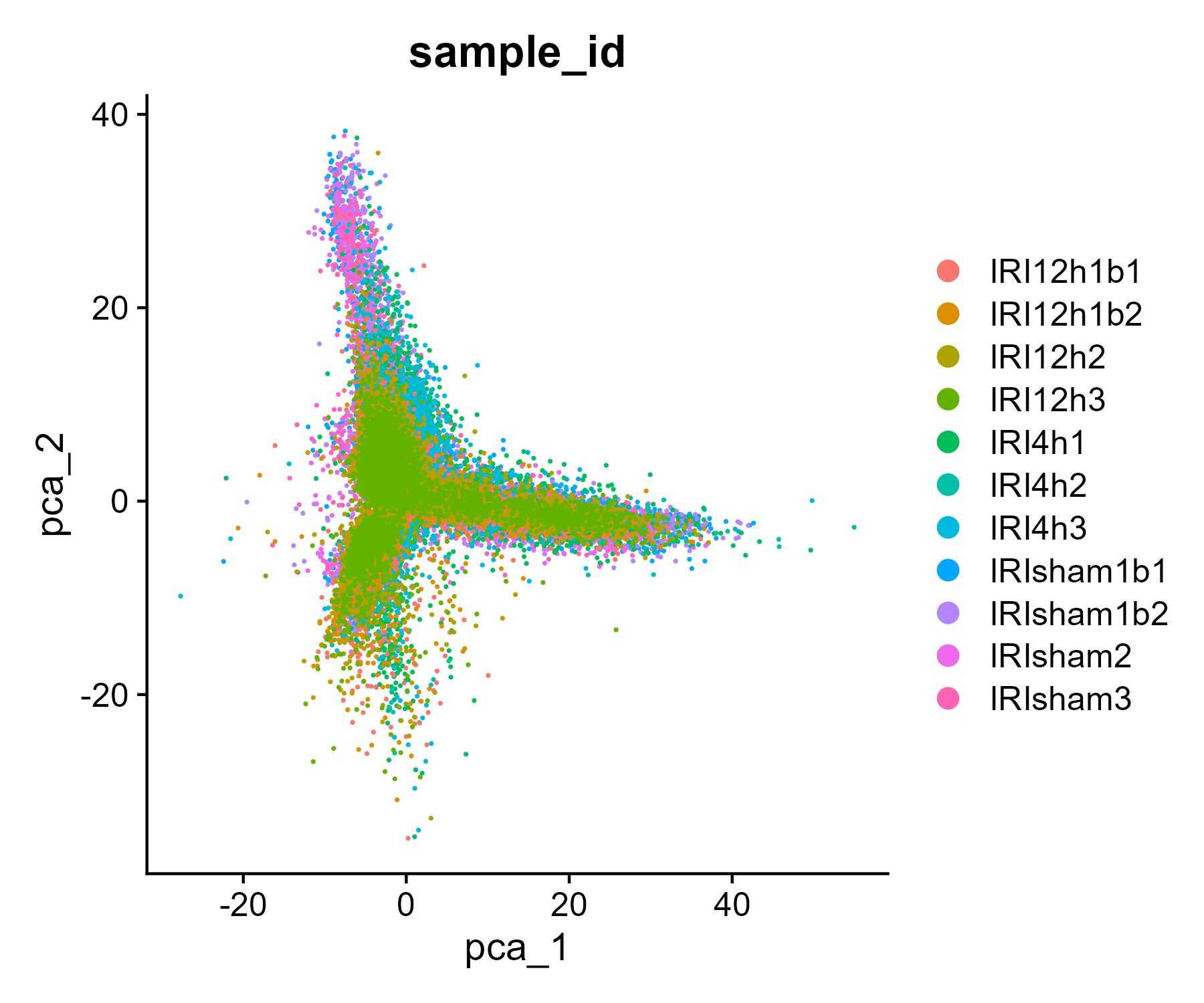


**Figure S3-010. Single-cell transcriptome analysis, step03_Integration: 03 pca by sample**


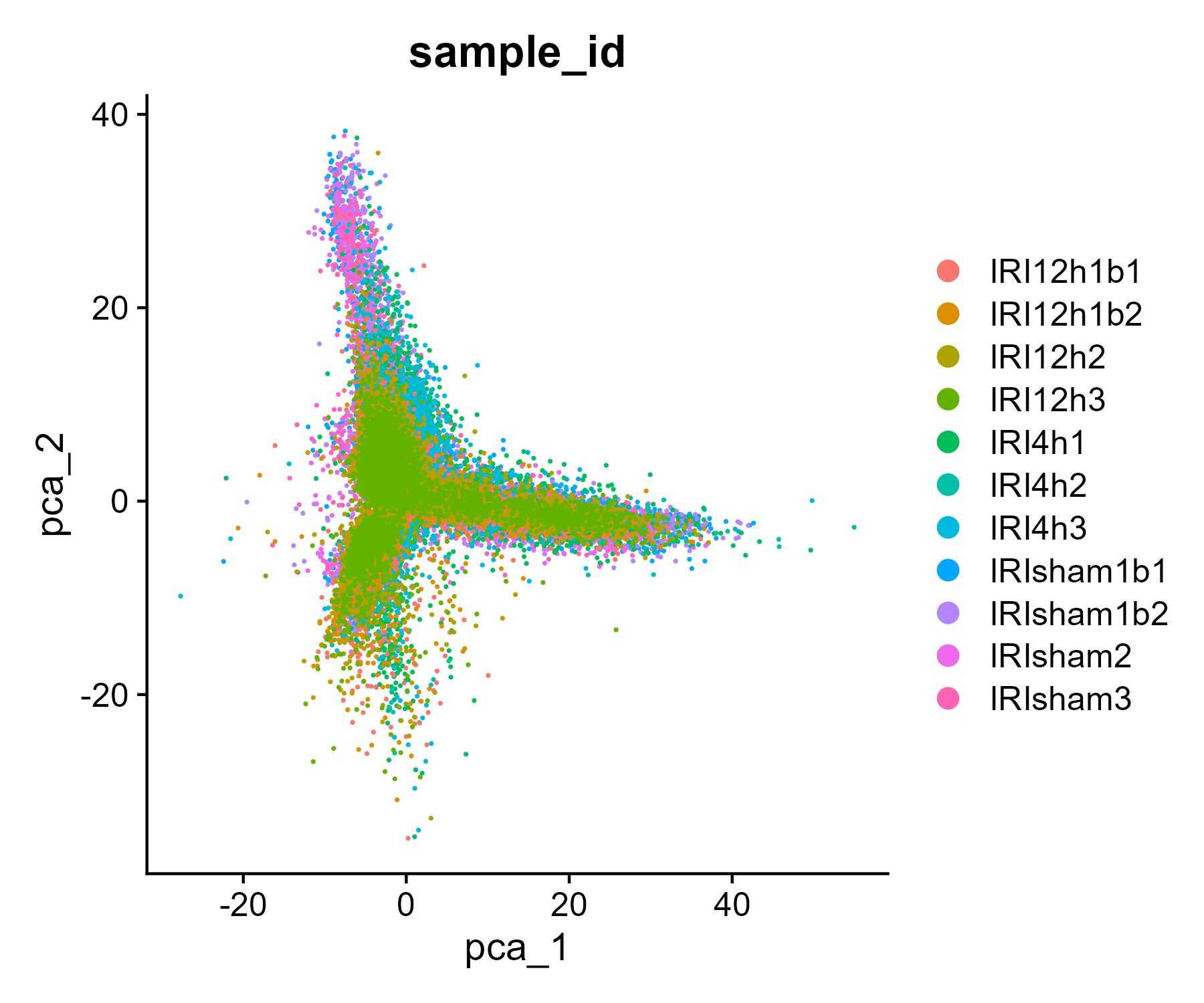


**Figure S3-011. Single-cell transcriptome analysis, step03_Integration: 03 pca by sample post**


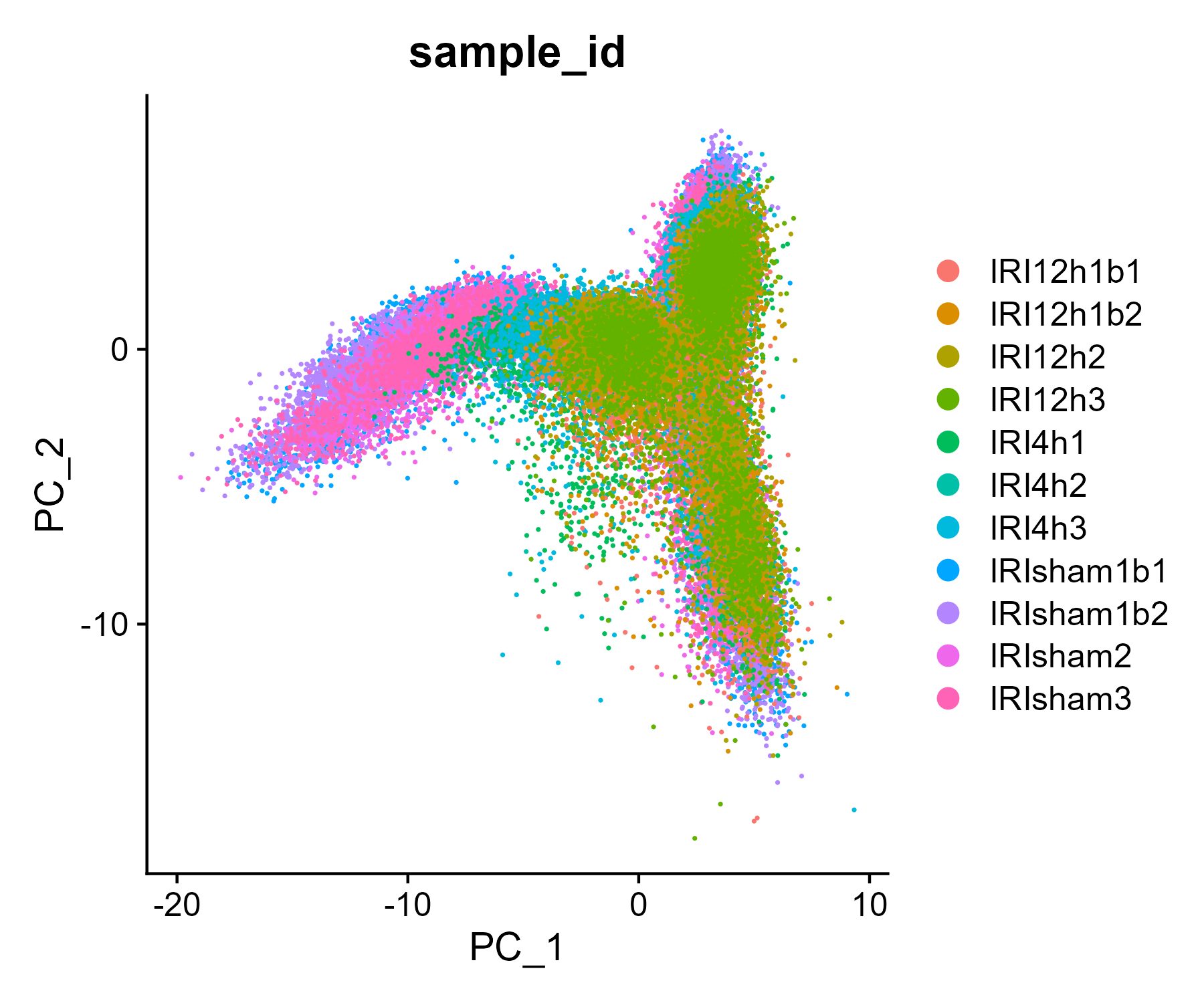


**Figure S3-012. Single-cell transcriptome analysis, step03_Integration: 03 pca by sample pre**


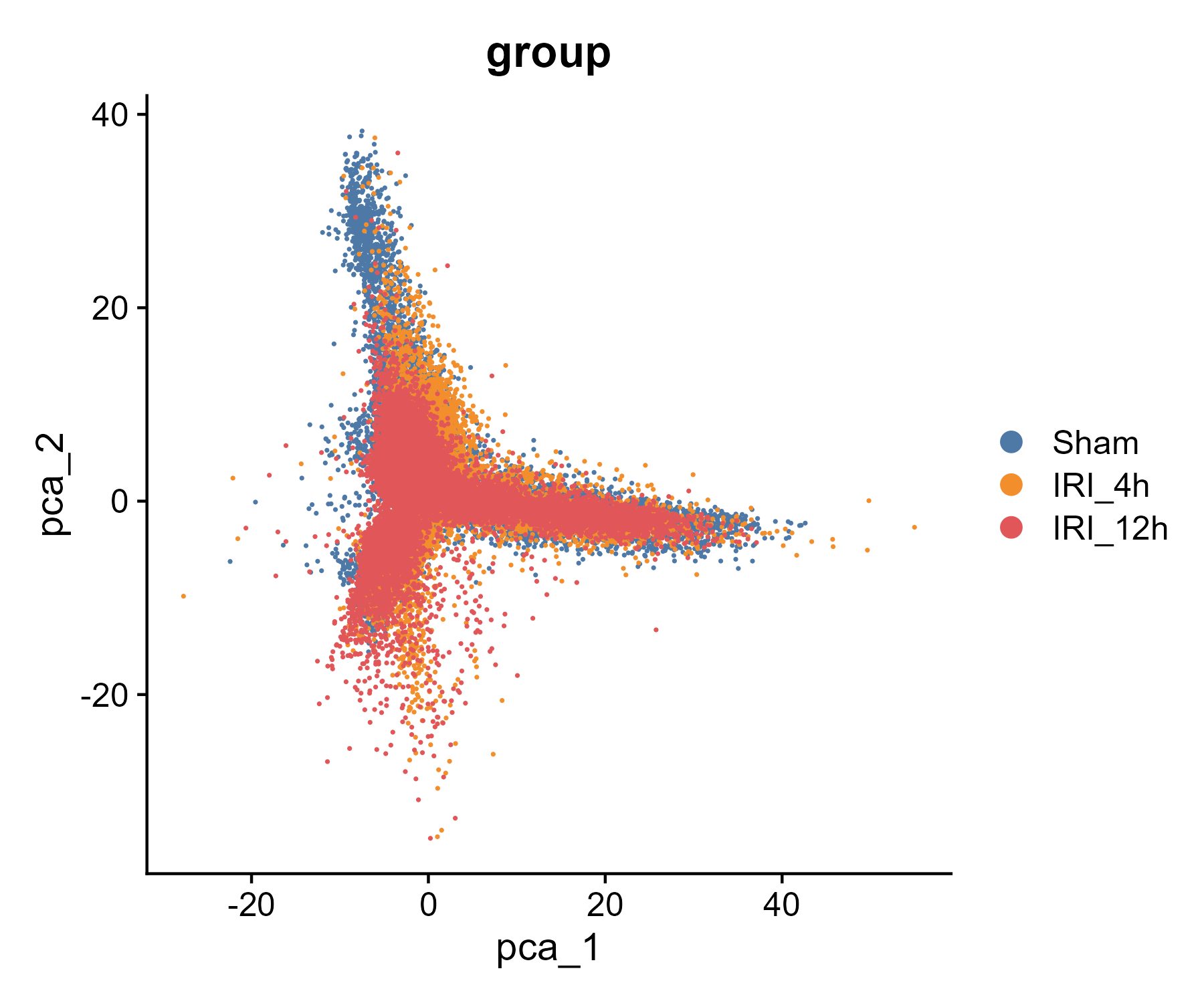


**Figure S3-013. Single-cell transcriptome analysis, step03_Integration: 03 pca scatter**


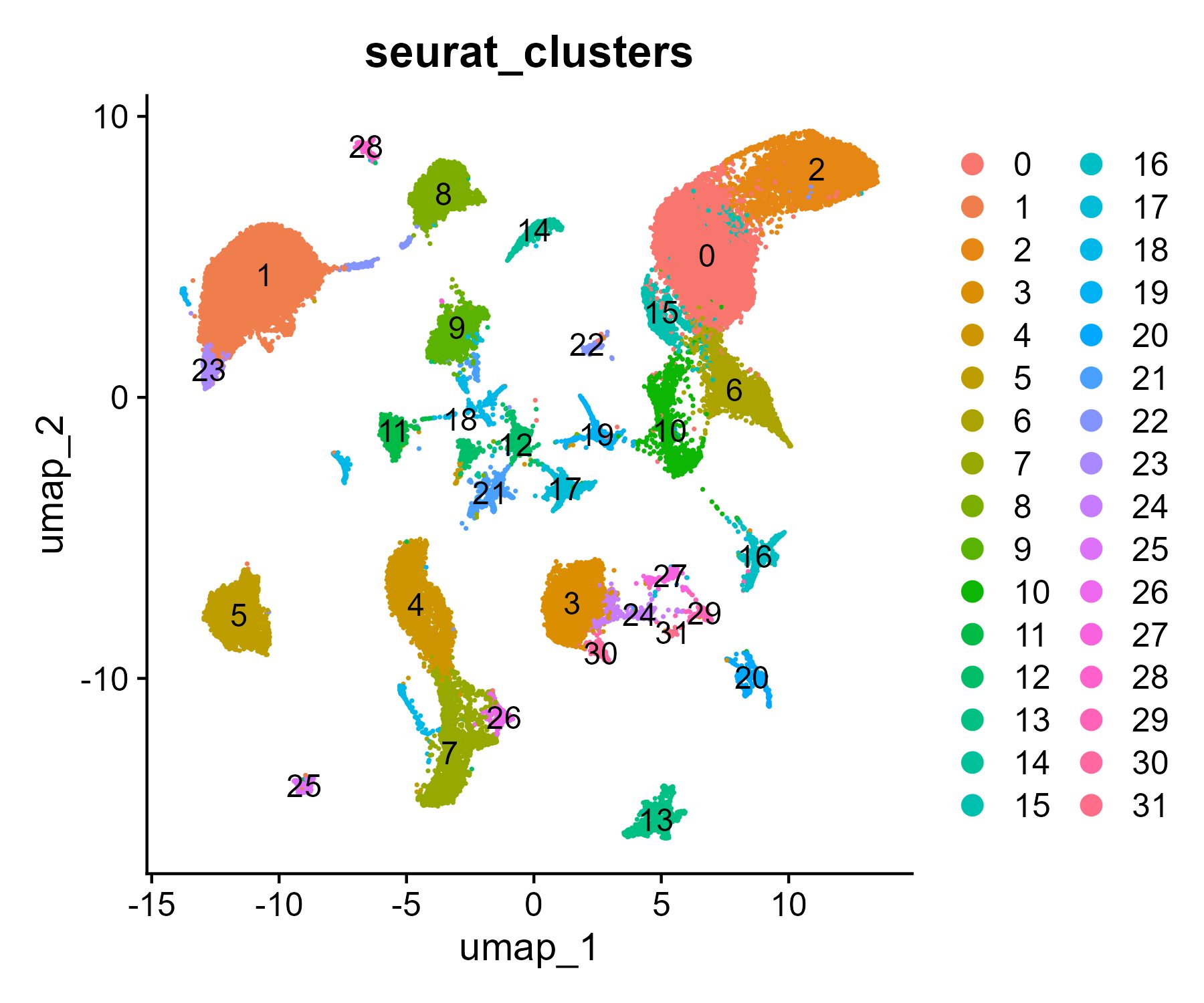


**Figure S3-014. Single-cell transcriptome analysis, step03_Integration: 03 umap by cluster**


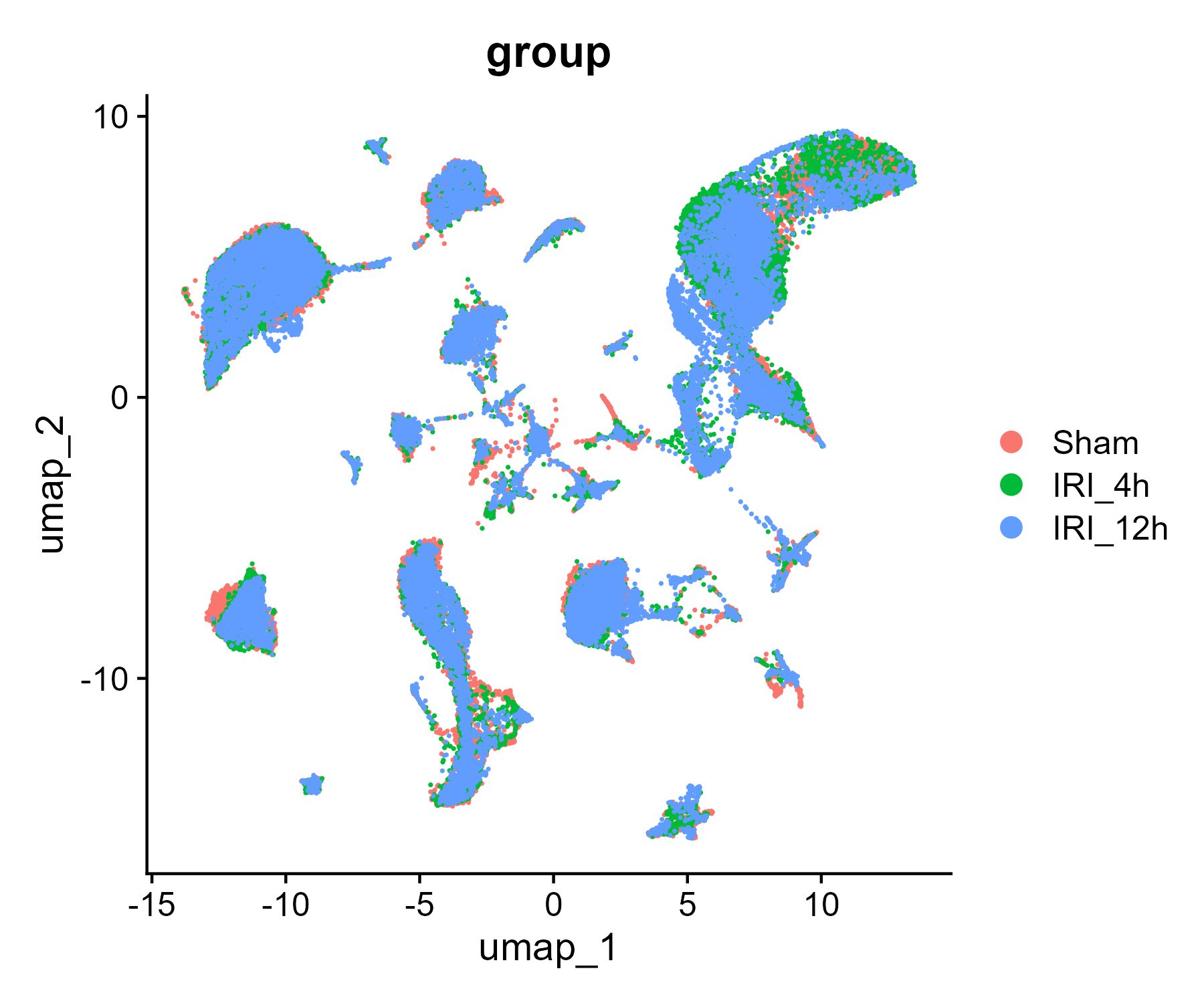


**Figure S3-015. Single-cell transcriptome analysis, step03_Integration: 03 umap by group**


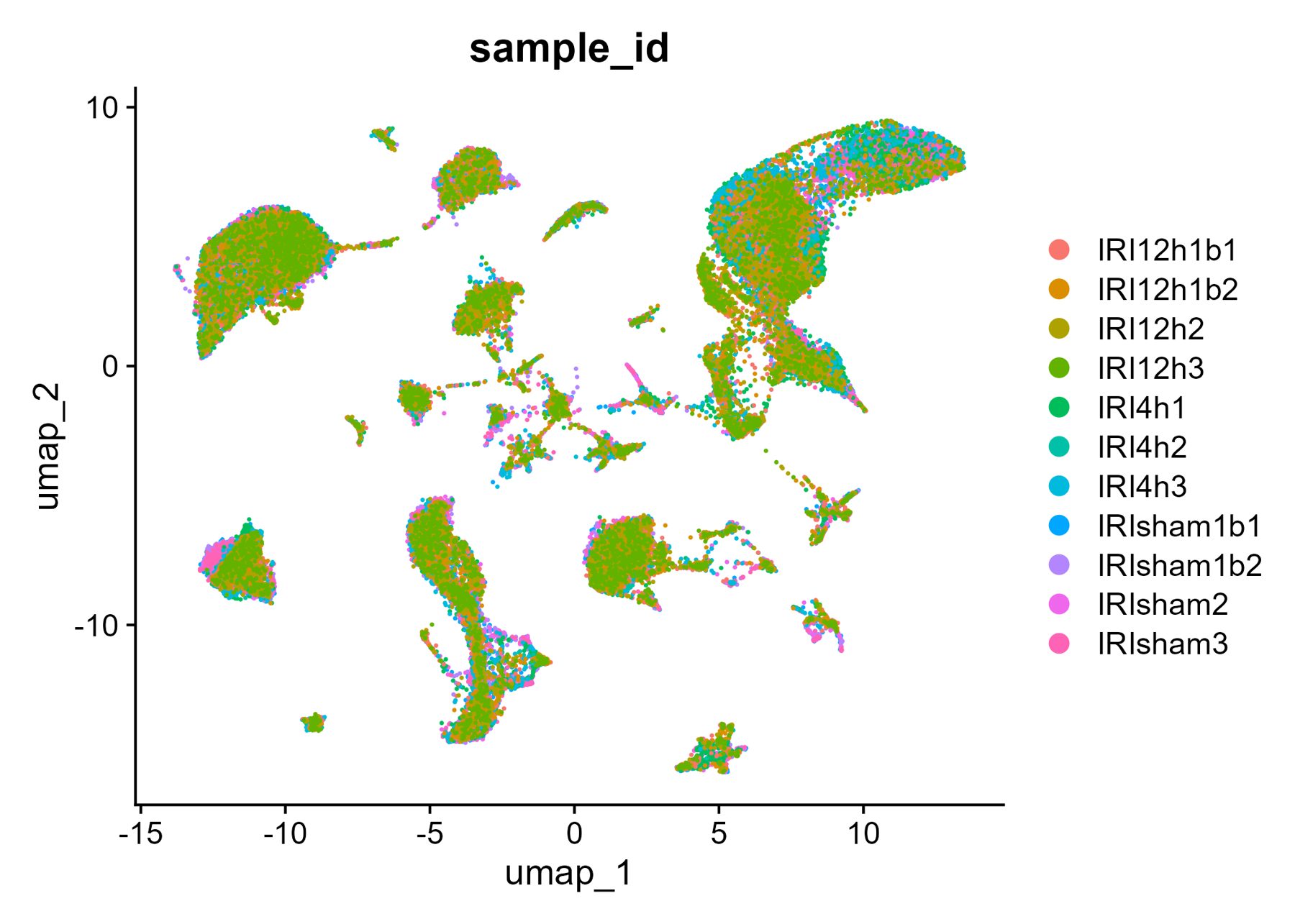


**Figure S3-016. Single-cell transcriptome analysis, step03_Integration: 03 umap by sample**


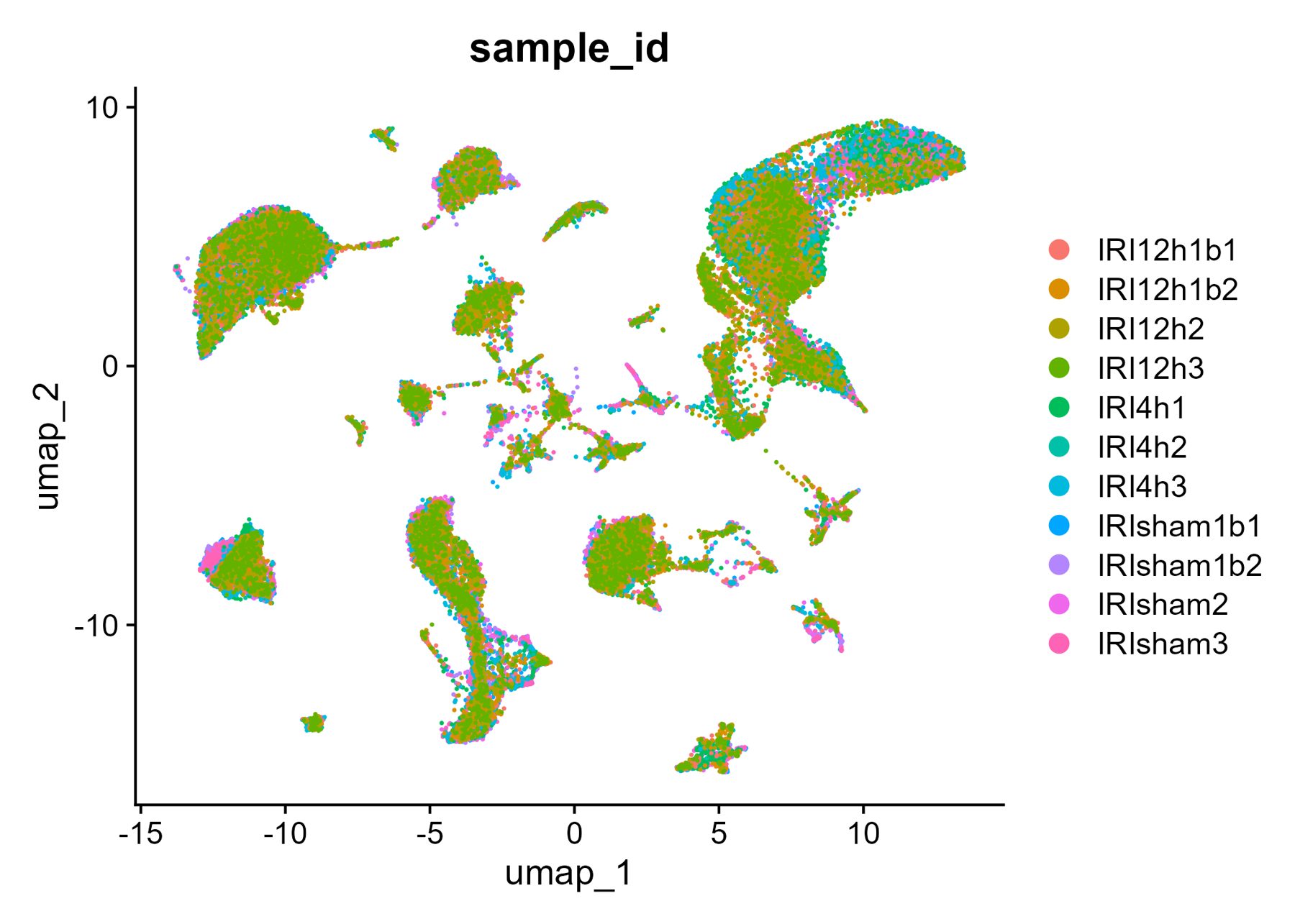


**Figure S3-017. Single-cell transcriptome analysis, step03_Integration: 03 umap by sample post**


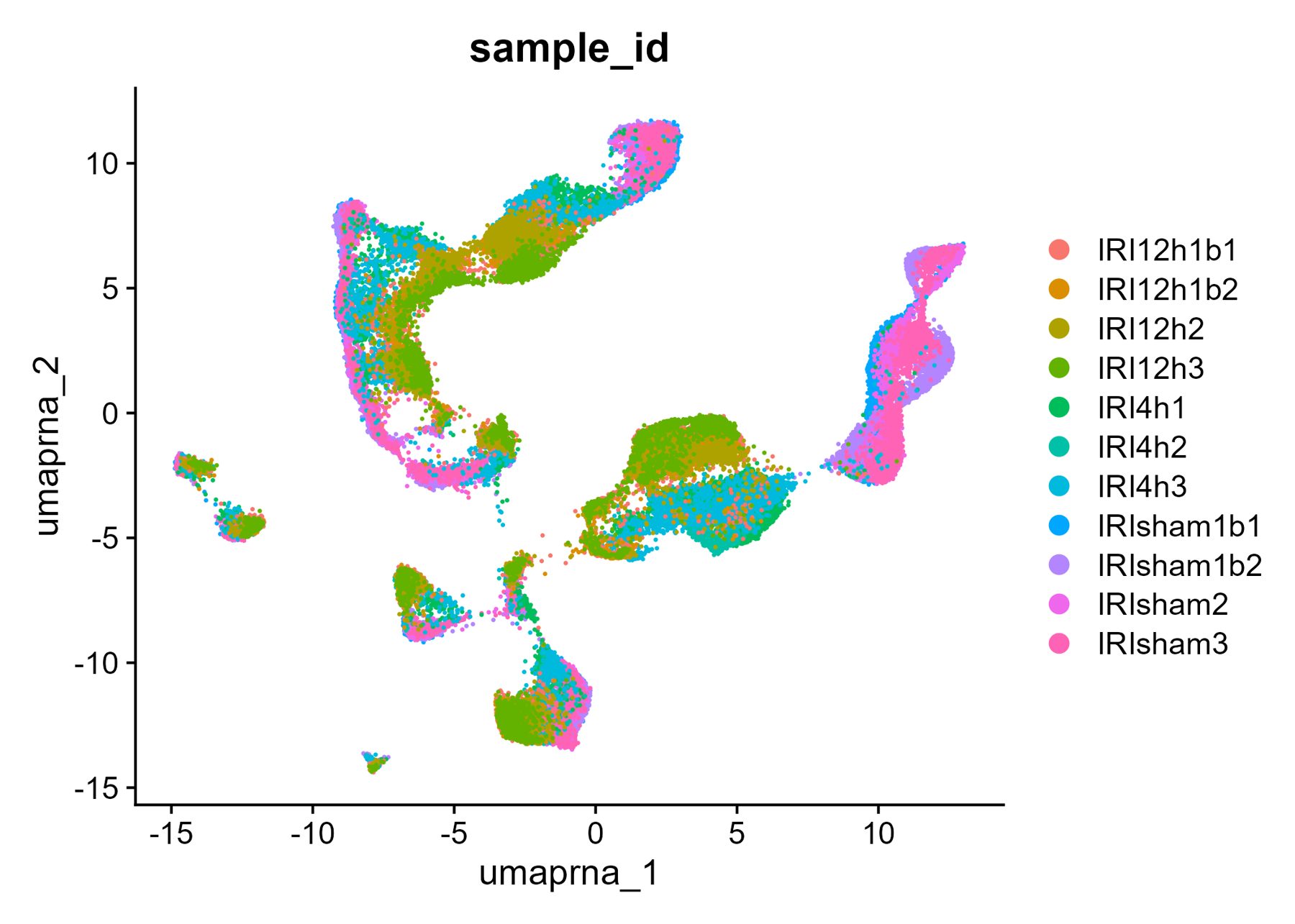


**Figure S3-018. Single-cell transcriptome analysis, step03_Integration: 03 umap by sample pre**


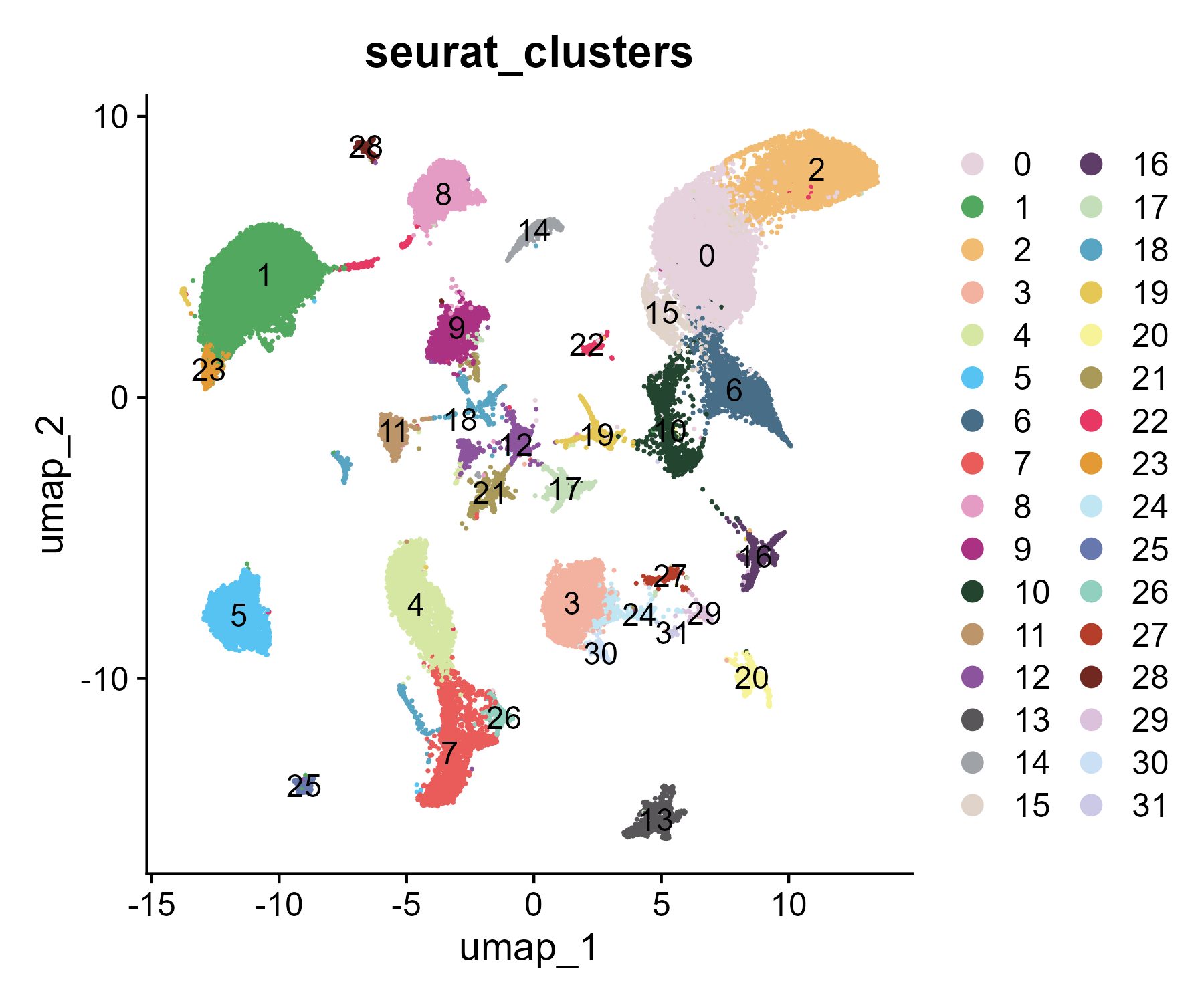


**Figure S3-019. Single-cell transcriptome analysis, step03_Integration: 03 umap cluster**


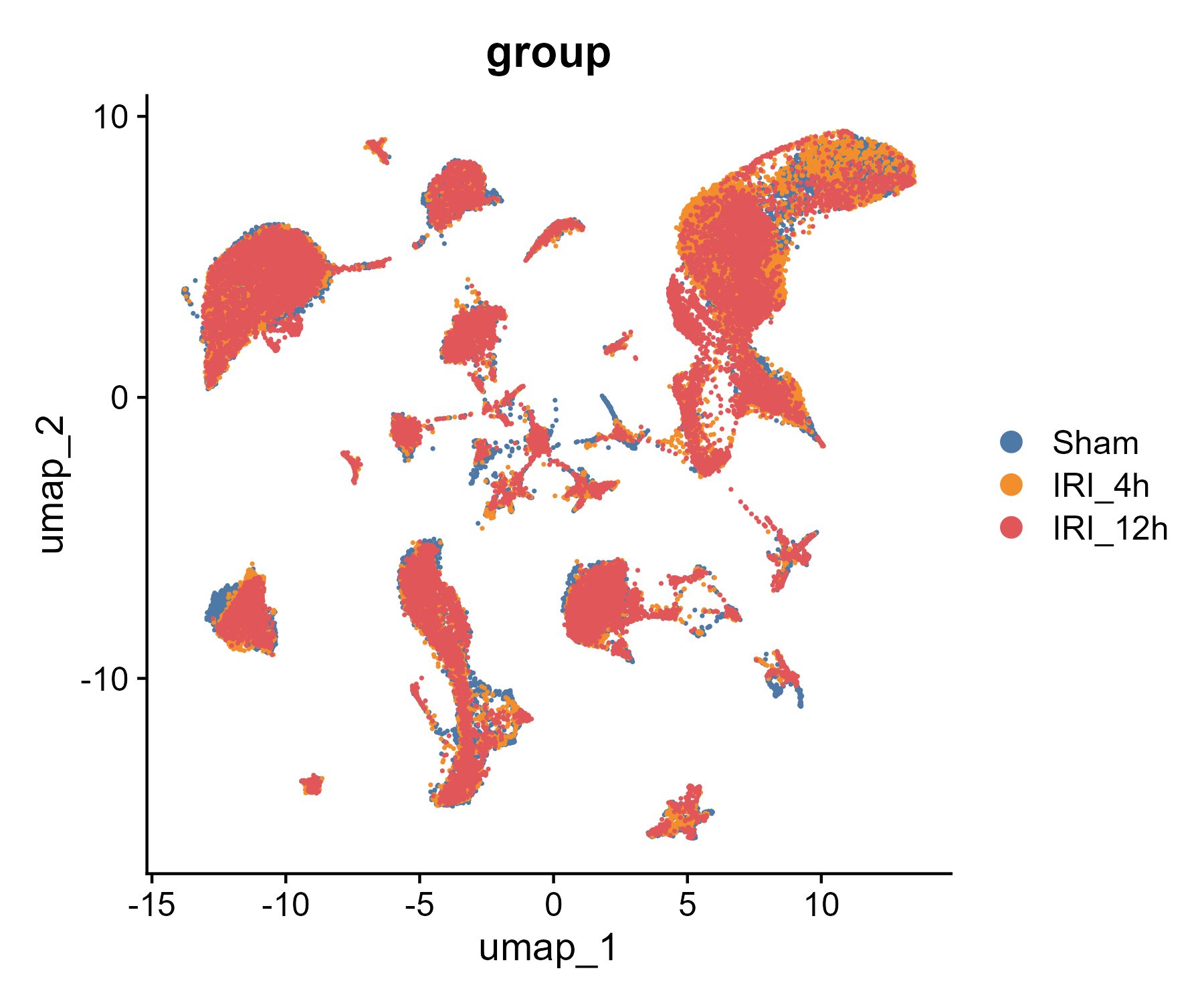


**Figure S3-020. Single-cell transcriptome analysis, step03_Integration: 03 umap group**


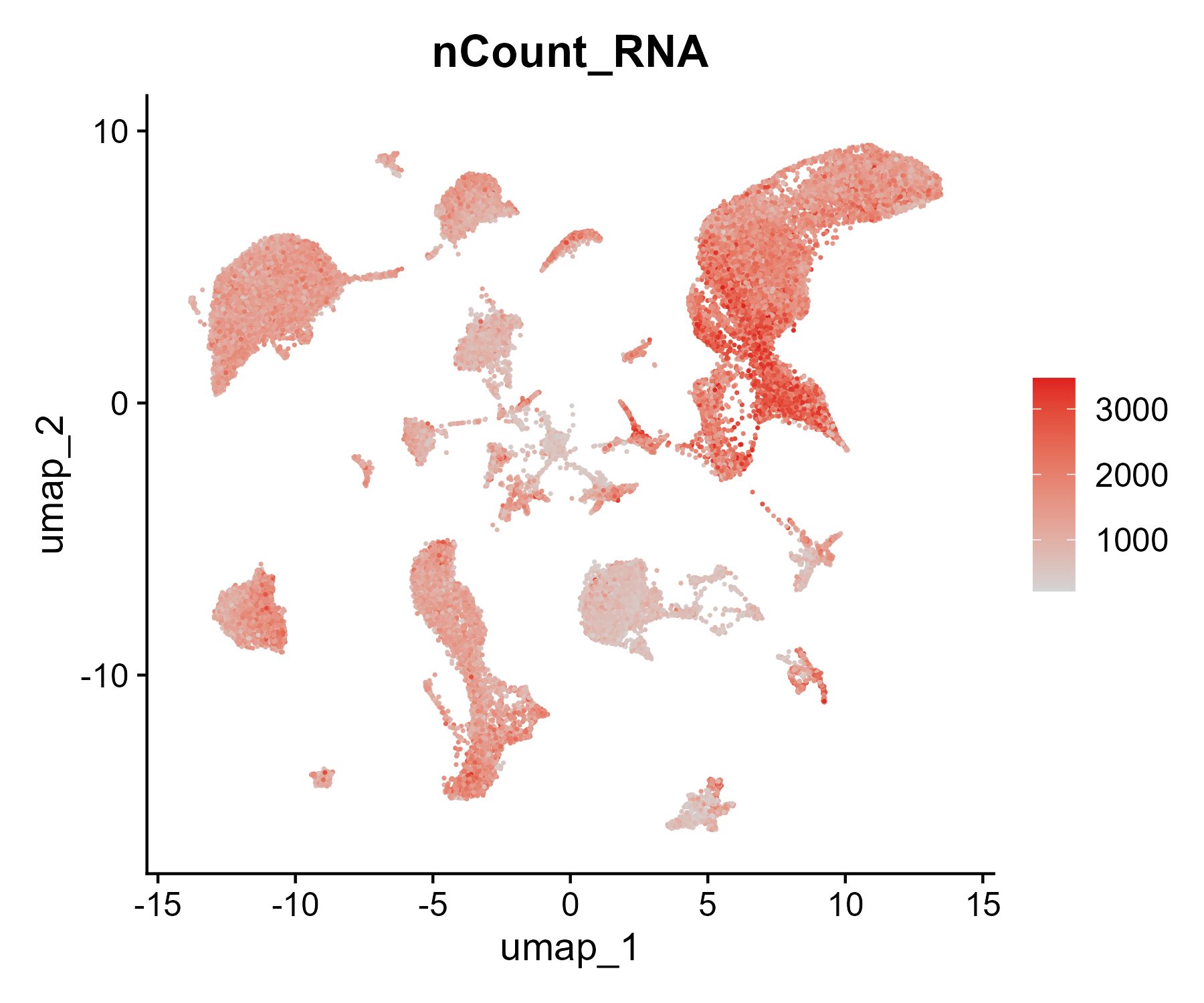


**Figure S3-021. Single-cell transcriptome analysis, step03_Integration: 03 umap nCount**


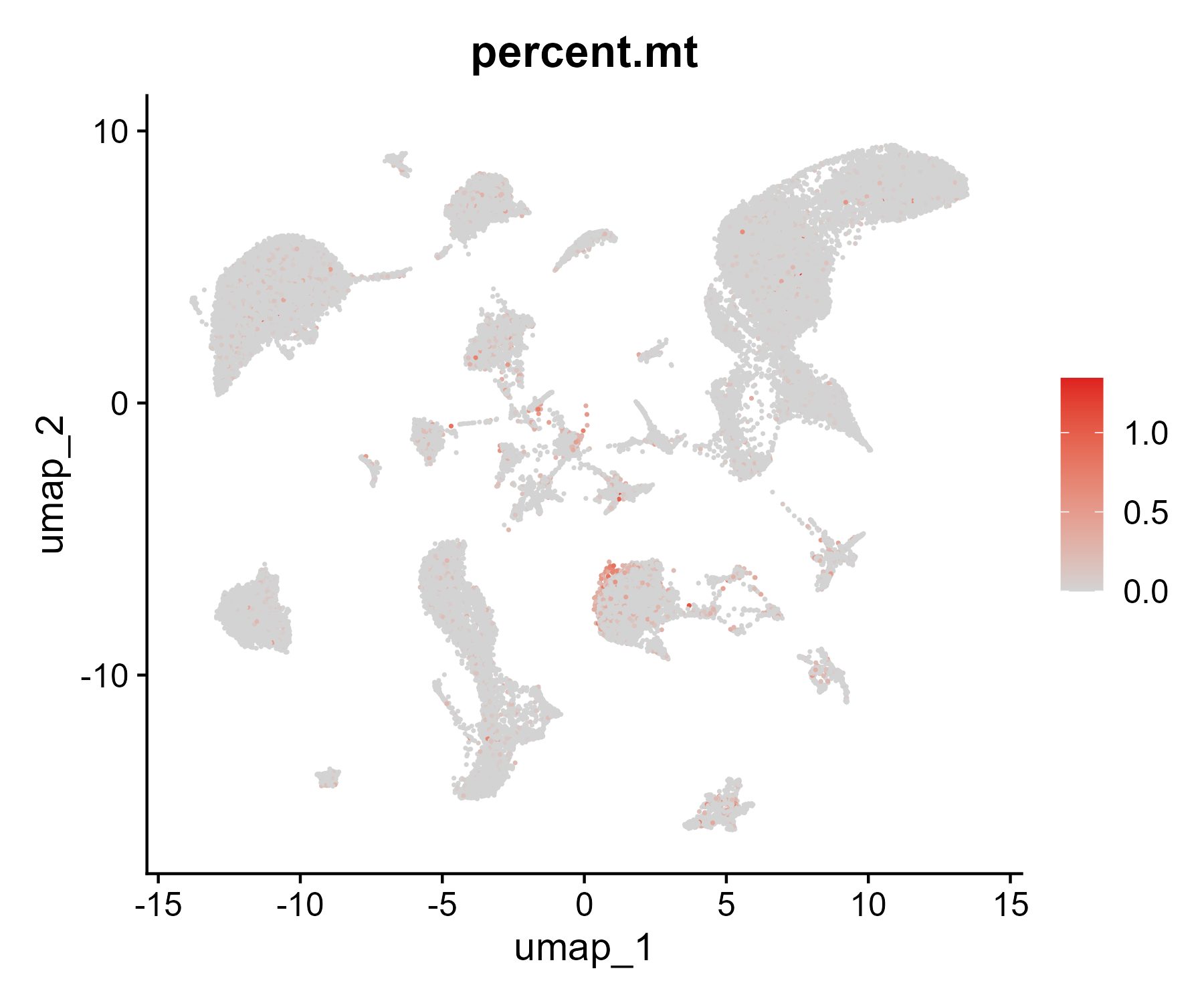


**Figure S3-022. Single-cell transcriptome analysis, step03_Integration: 03 umap percentmt**


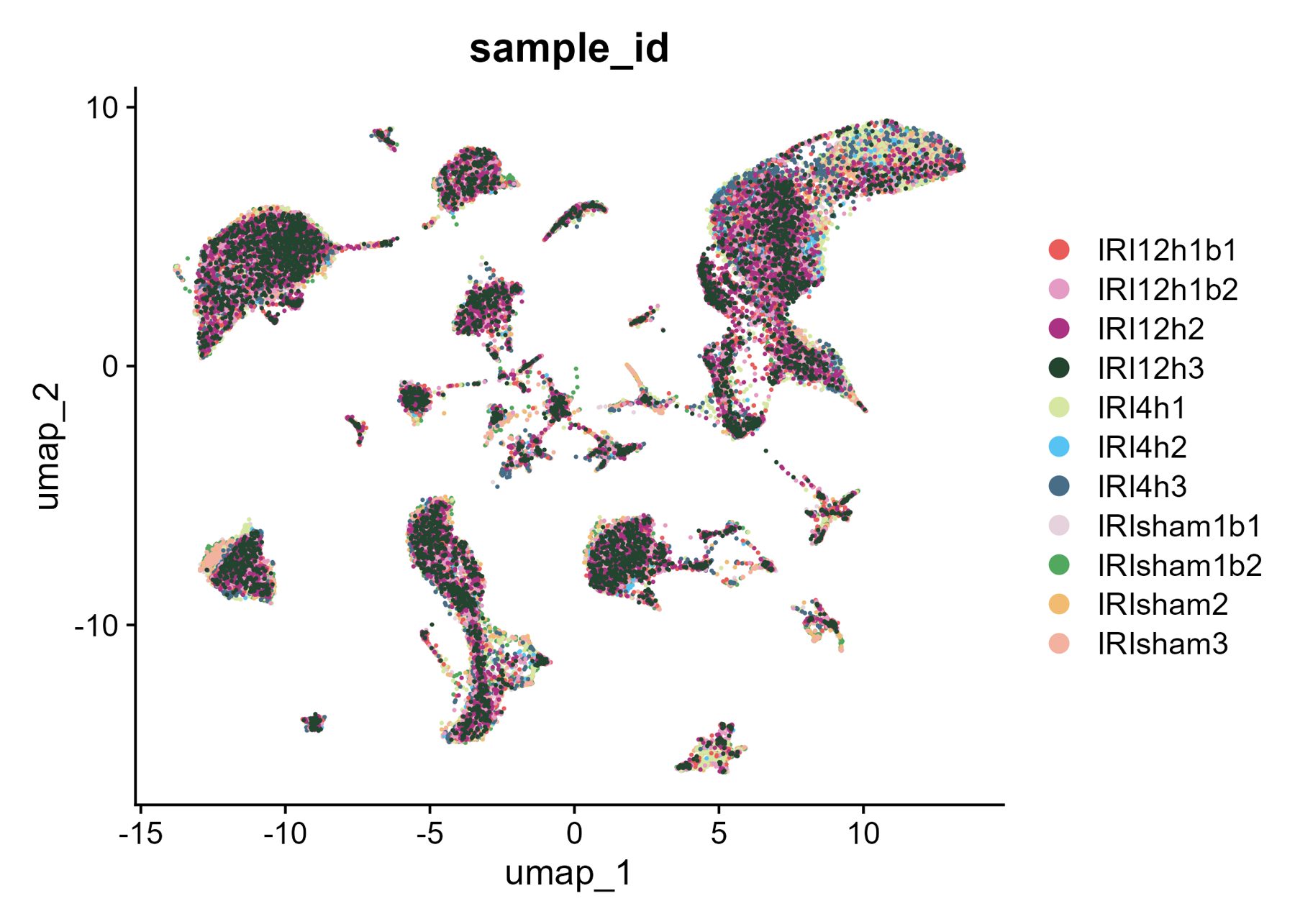


**Figure S3-023. Single-cell transcriptome analysis, step03_Integration: 03 umap sample**

# Section: step04_Celltype


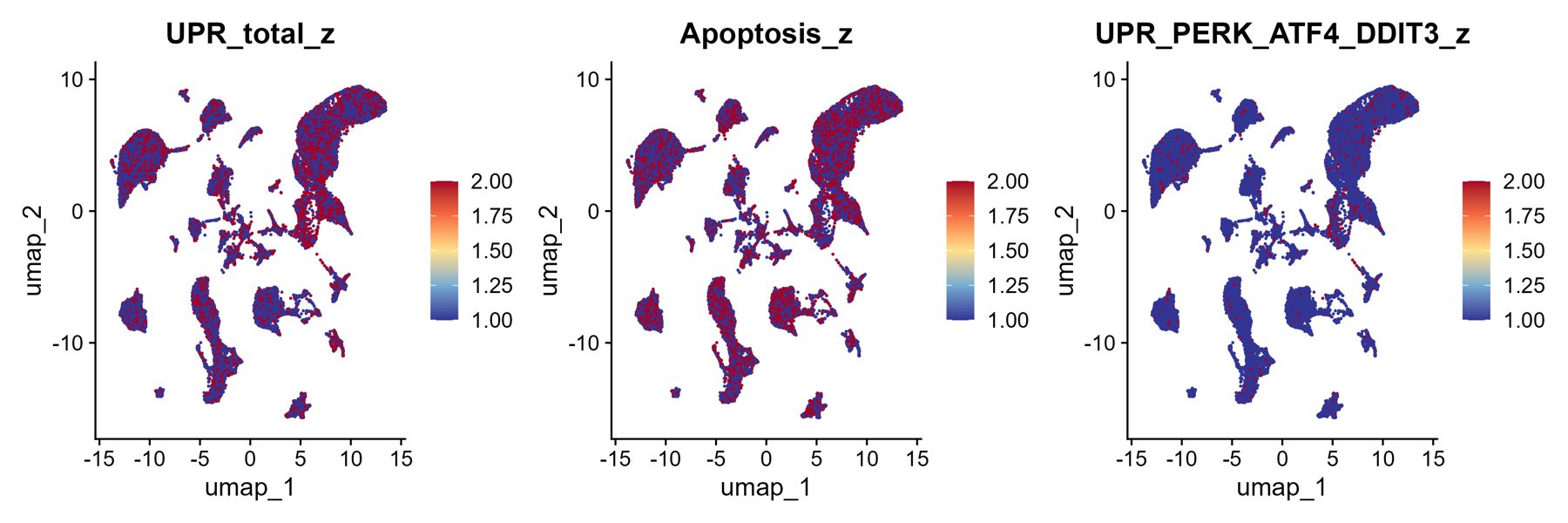


**Figure S3-024. Single-cell transcriptome analysis, step04_Celltype: 04 allcells score featureplot**


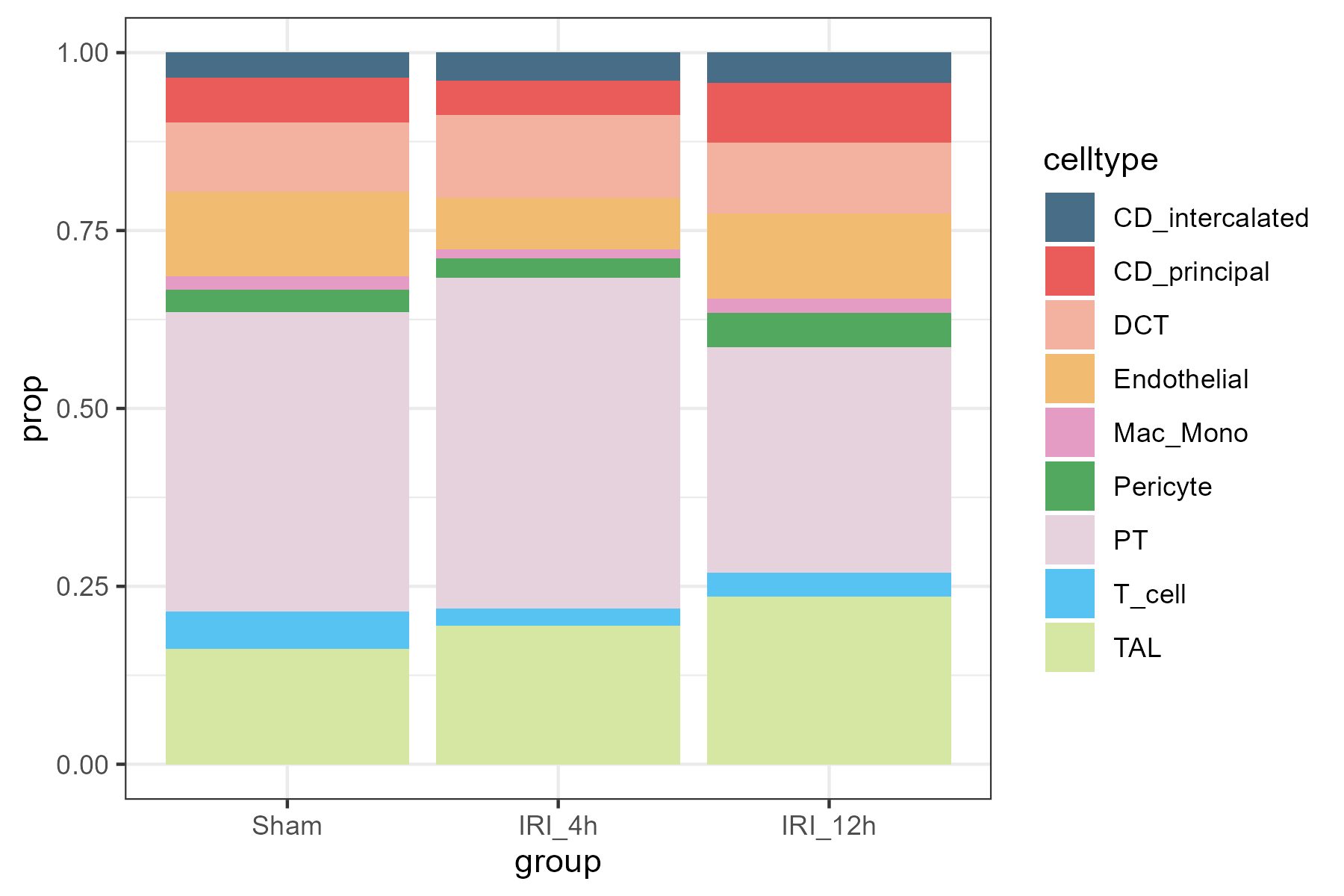


**Figure S3-025. Single-cell transcriptome analysis, step04_Celltype: 04 celltype by group stackedbar**


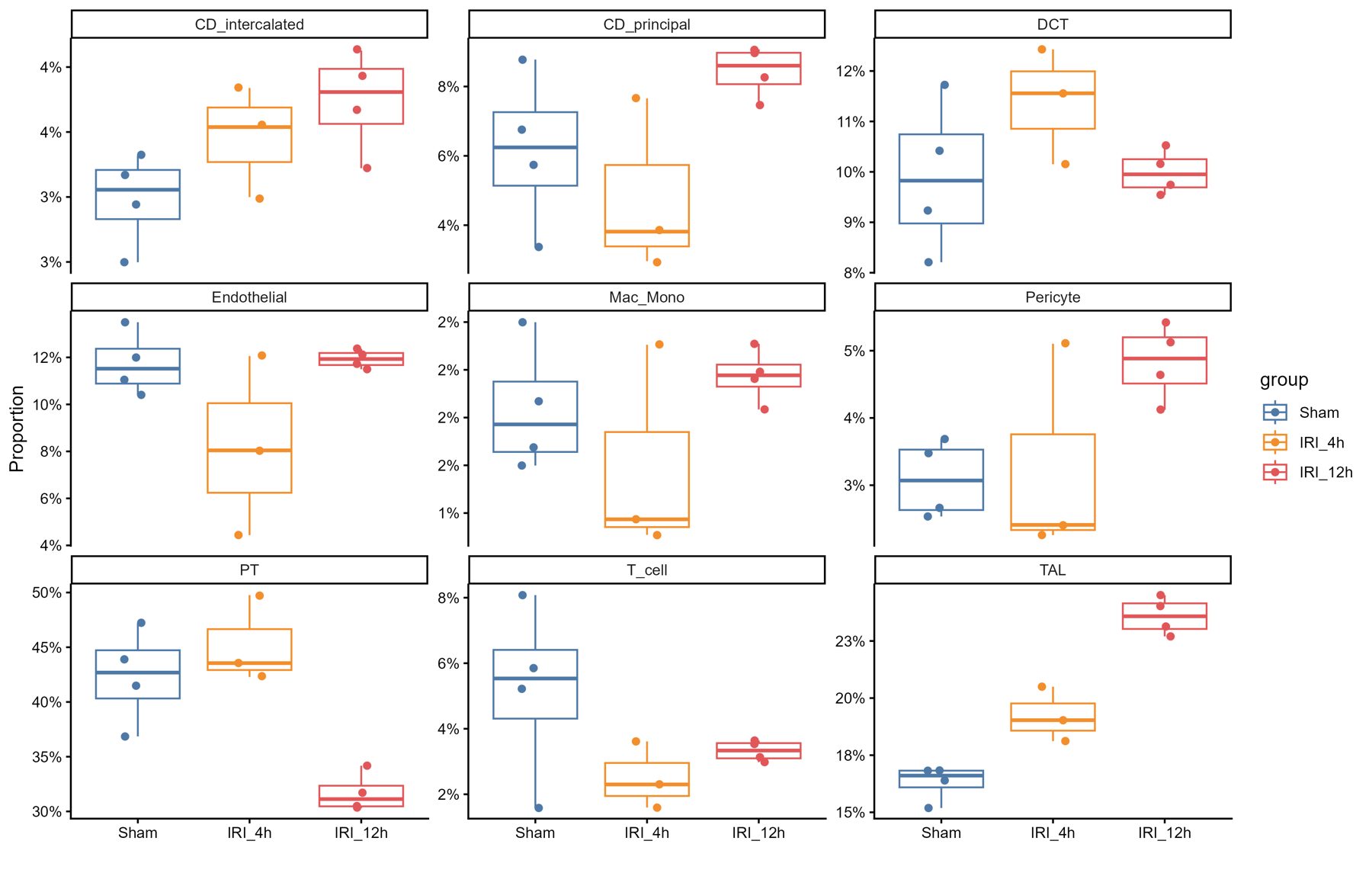


**Figure S3-026. Single-cell transcriptome analysis, step04_Celltype: 04 celltype prop by group boxplot**


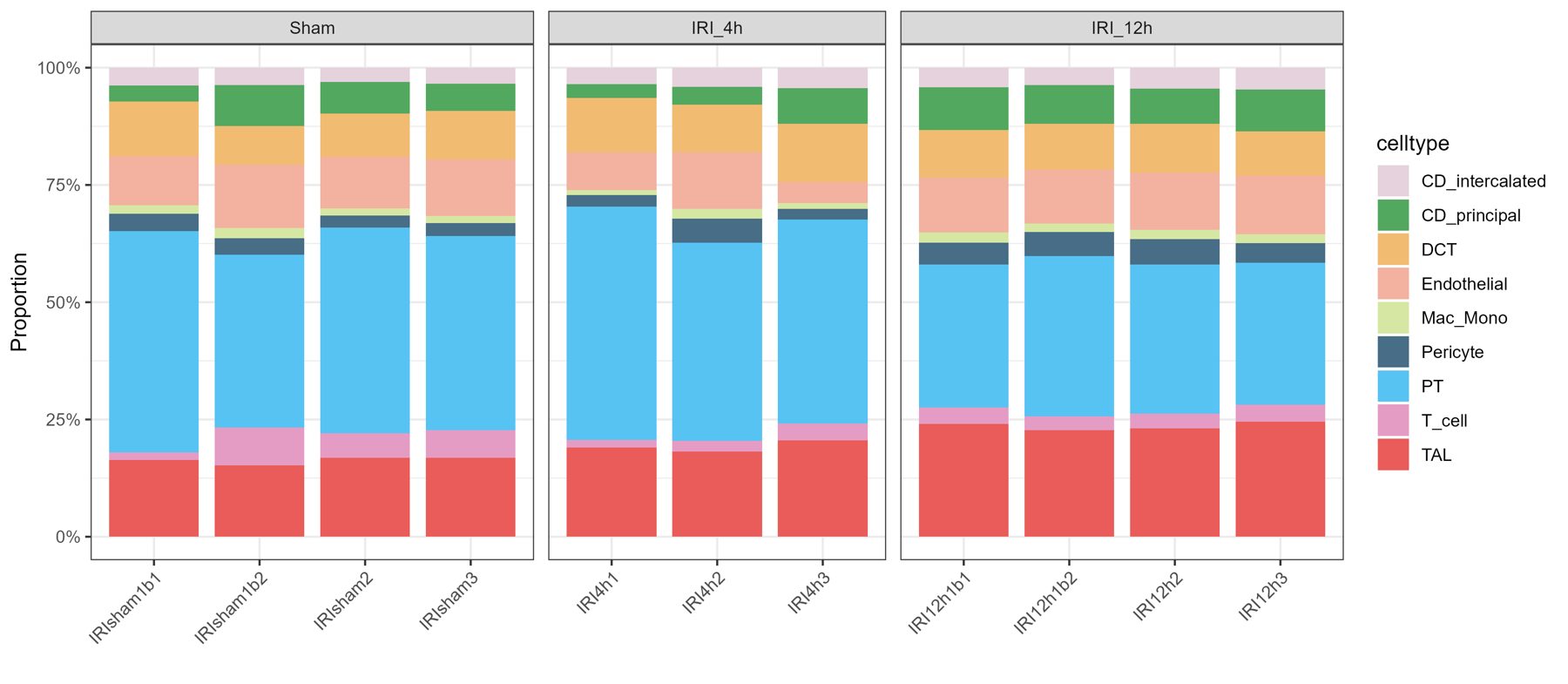


**Figure S3-027. Single-cell transcriptome analysis, step04_Celltype: 04 celltype prop by sample stackedbar**


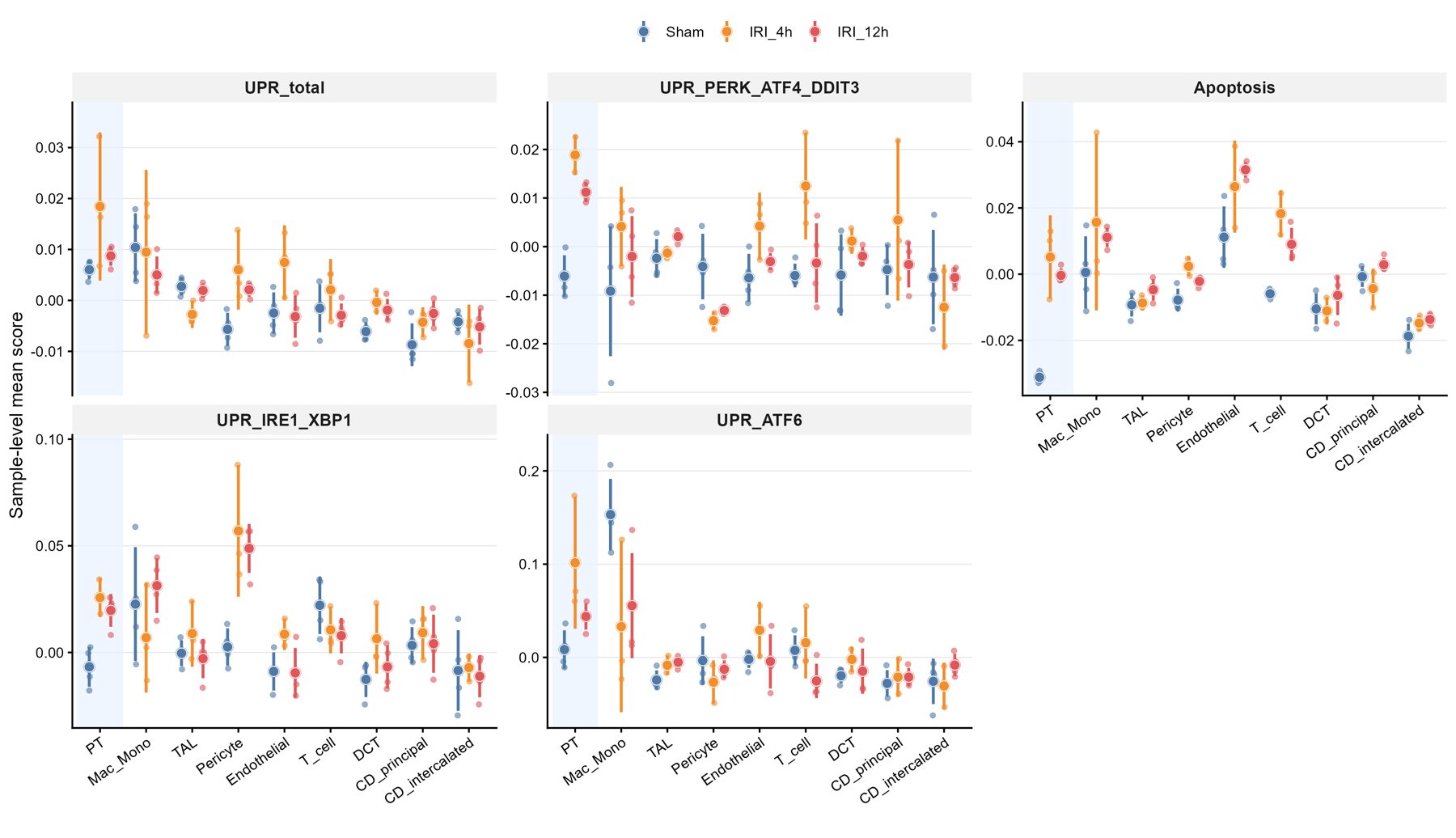


**Figure S3-028. Single-cell transcriptome analysis, step04_Celltype: 04 celltype score samplelevel boxplot**


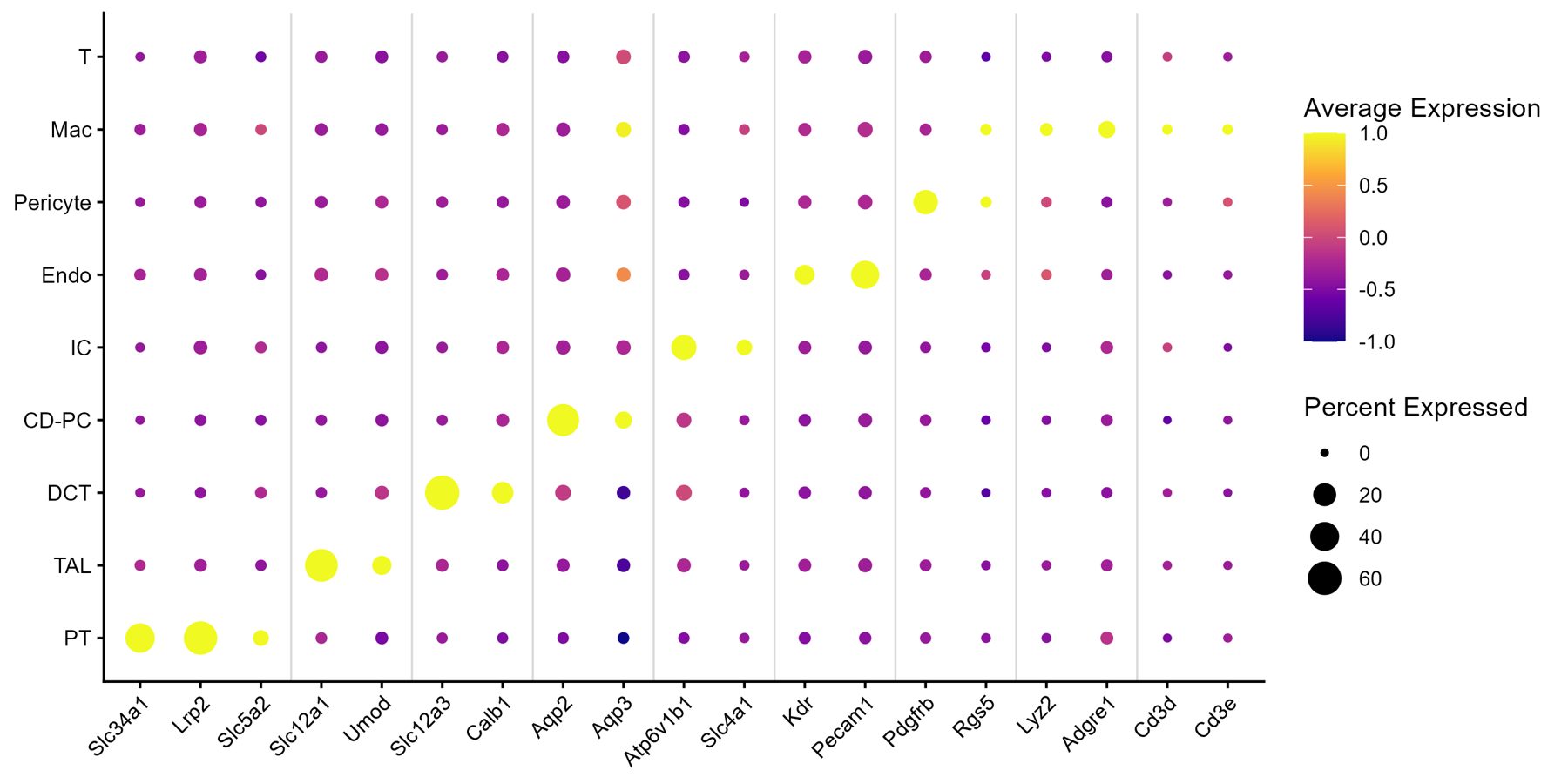


**Figure S3-029. Single-cell transcriptome analysis, step04_Celltype: 04 dotplot markers global**


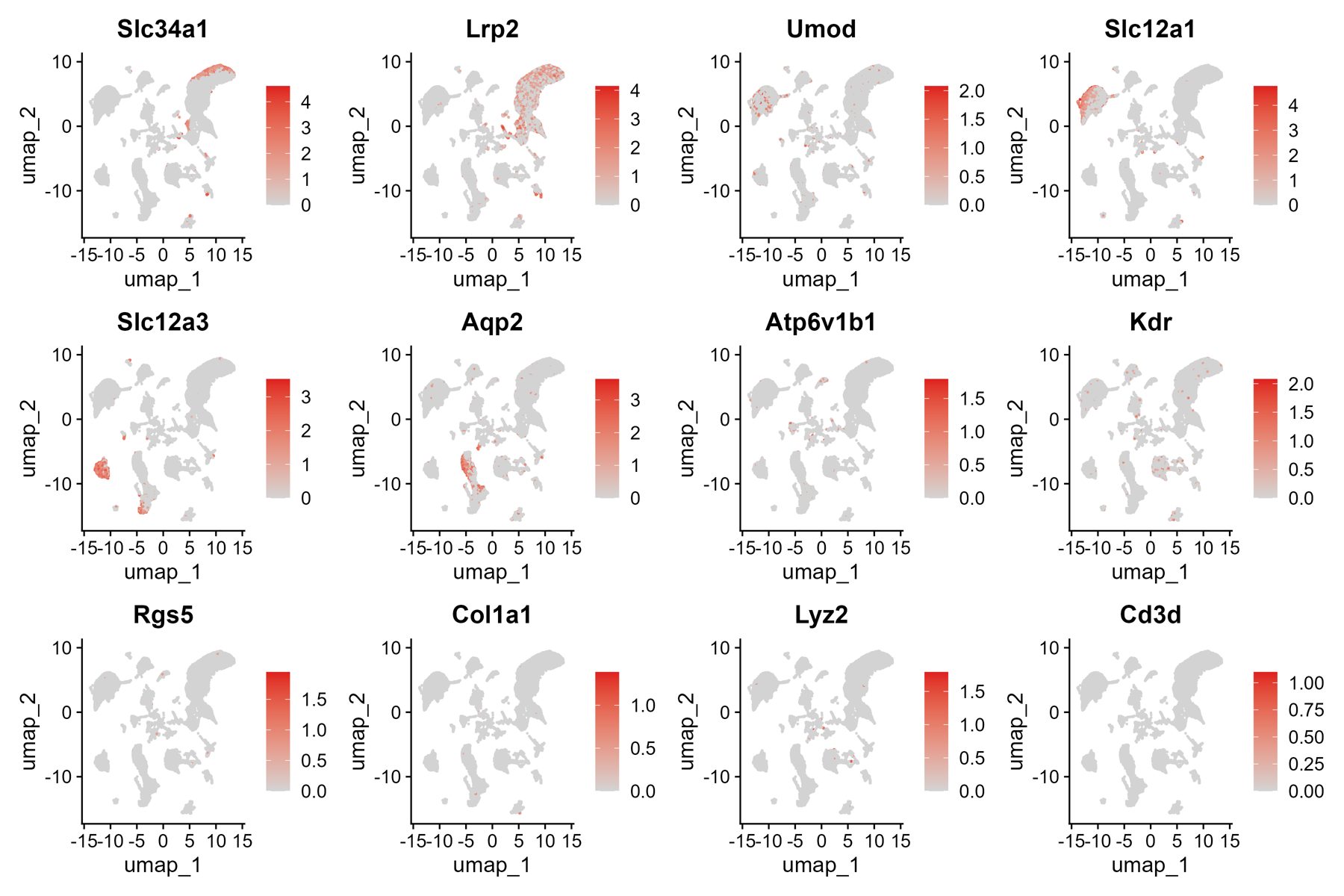


**Figure S3-030. Single-cell transcriptome analysis, step04_Celltype: 04 featureplot global markers**


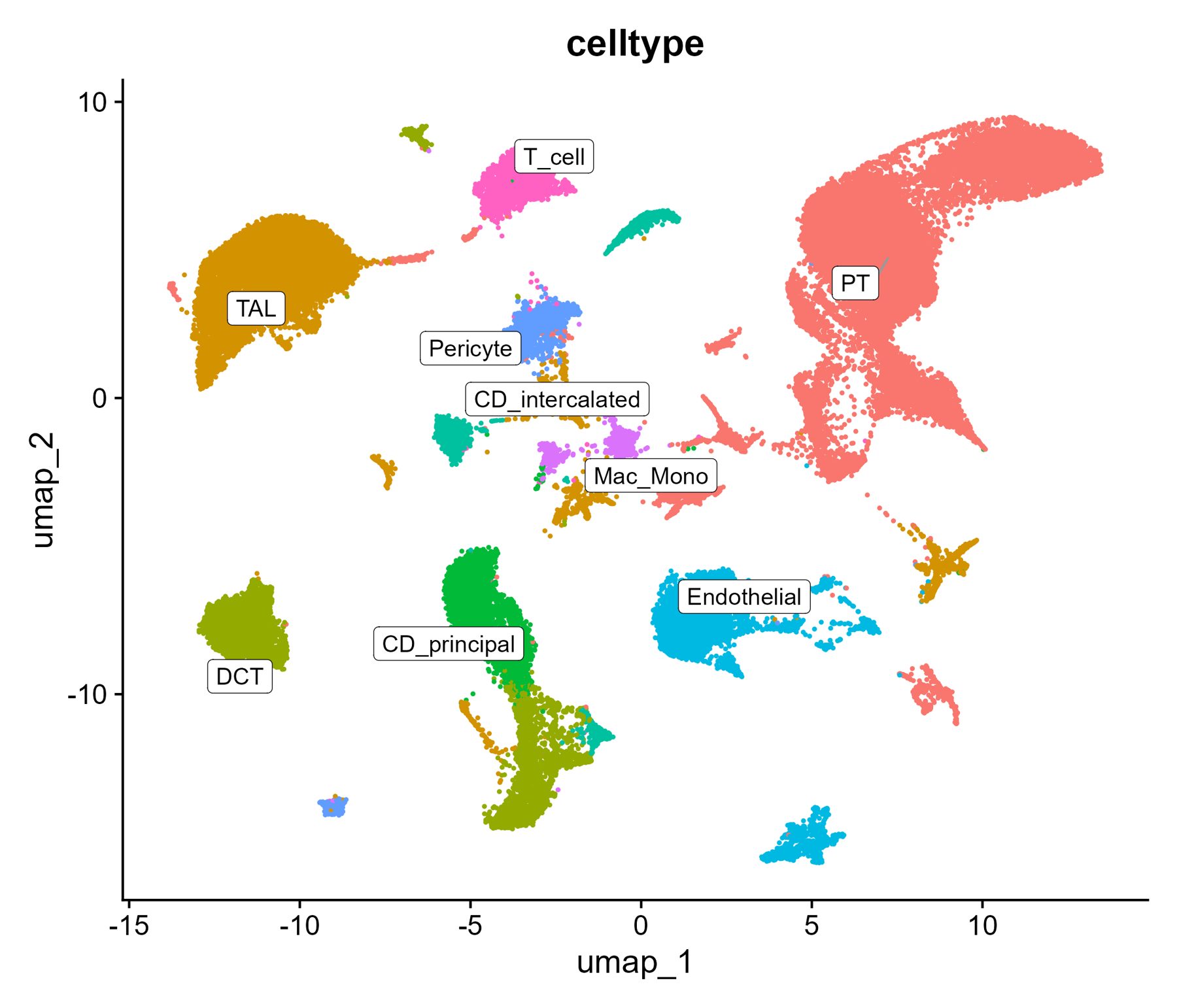


**Figure S3-031. Single-cell transcriptome analysis, step04_Celltype: 04 umap celltype**

# Section: step05_PT


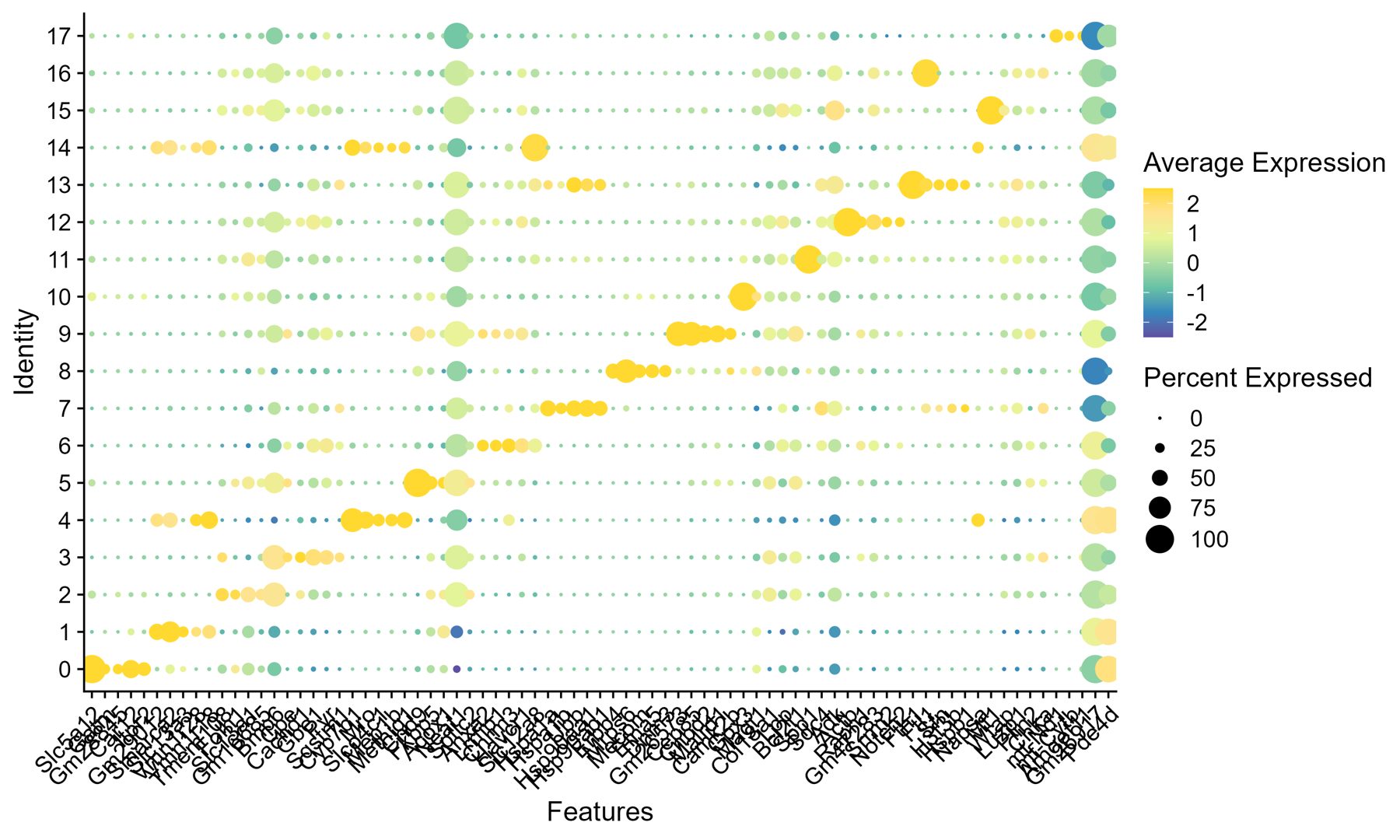


**Figure S3-032. Single-cell transcriptome analysis, step05_PT: 05 PT dotplot topmarkers**


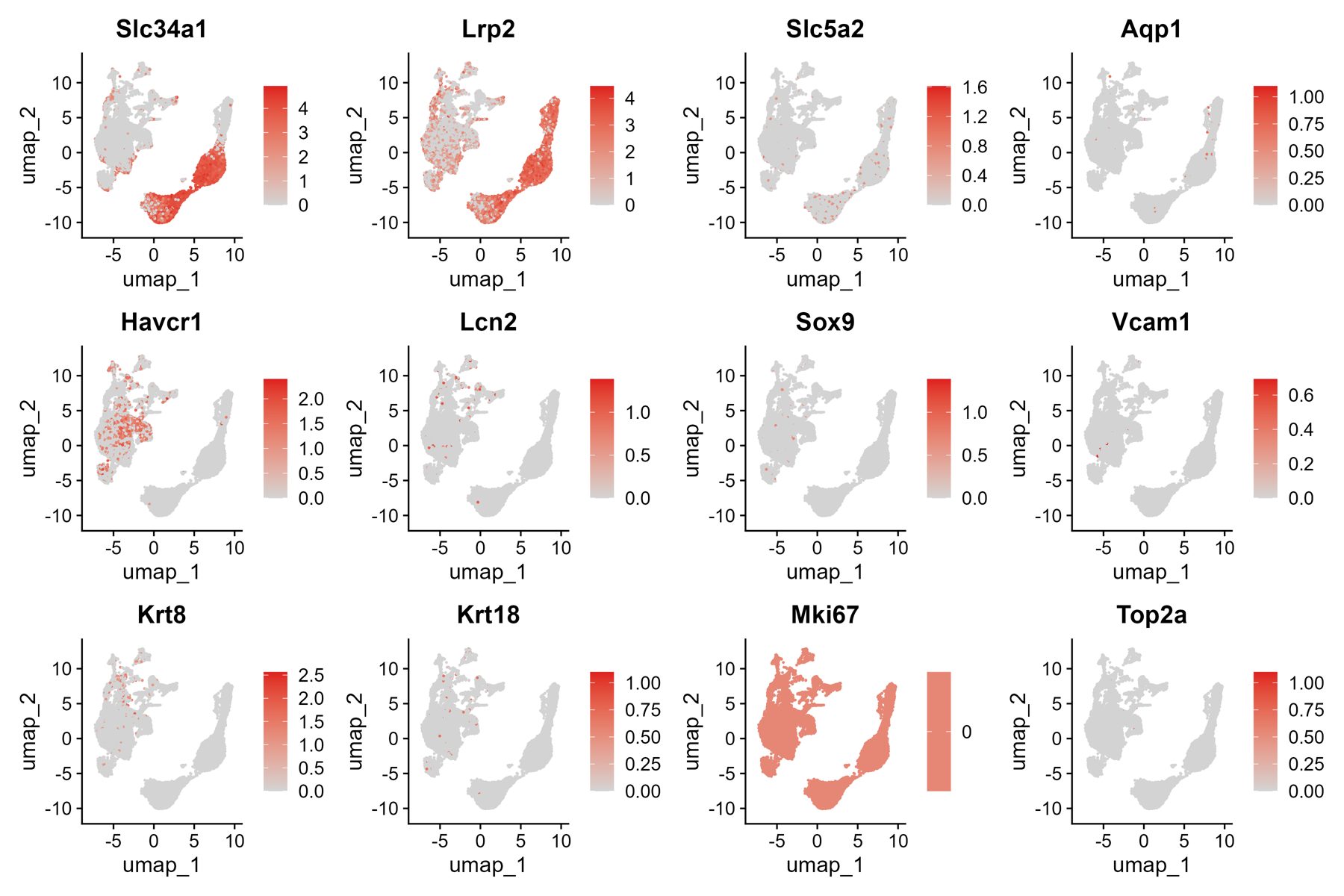


**Figure S3-033. Single-cell transcriptome analysis, step05_PT: 05 PT featureplot keymarkers**


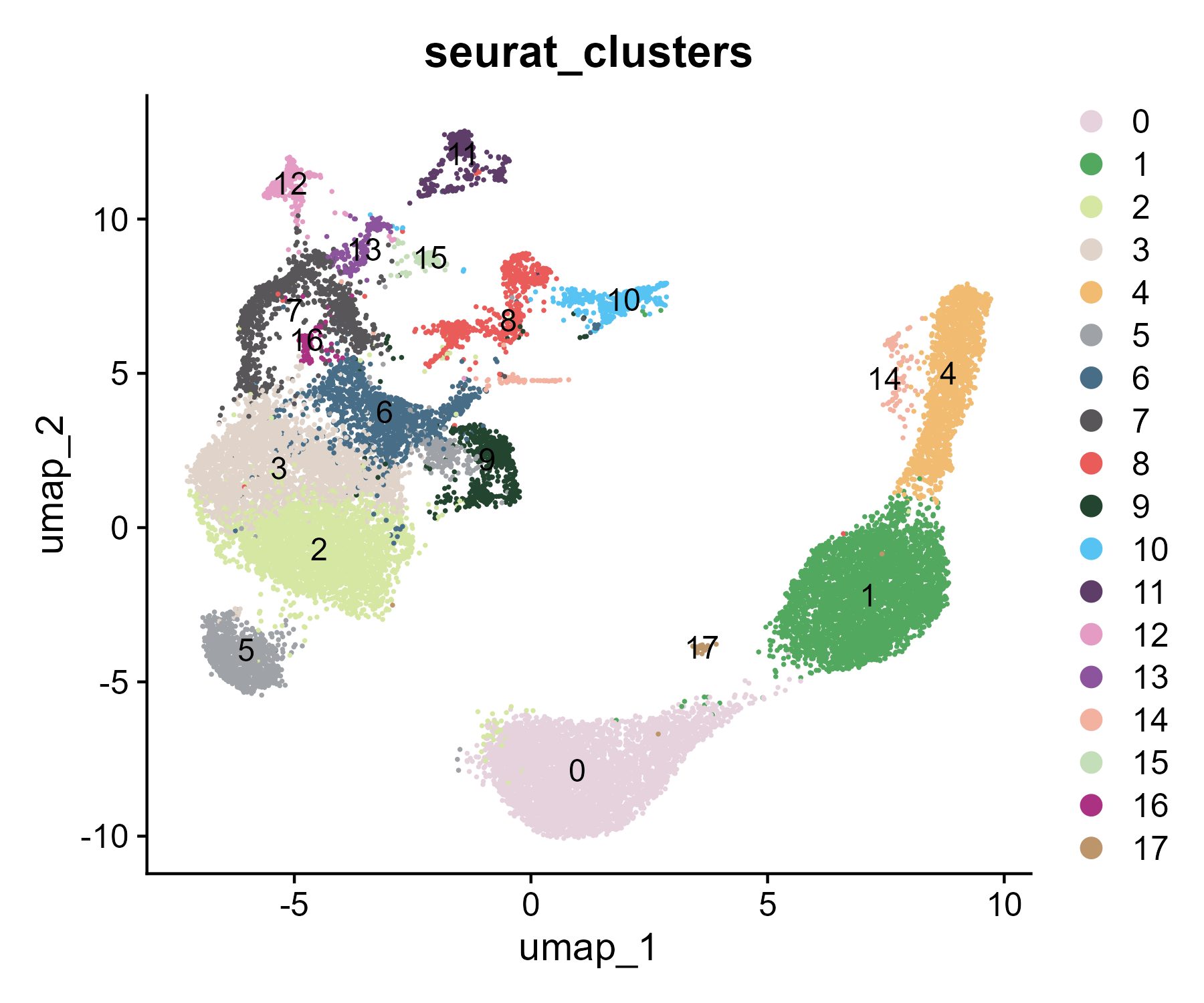


**Figure S3-034. Single-cell transcriptome analysis, step05_PT: 05 PT umap cluster**


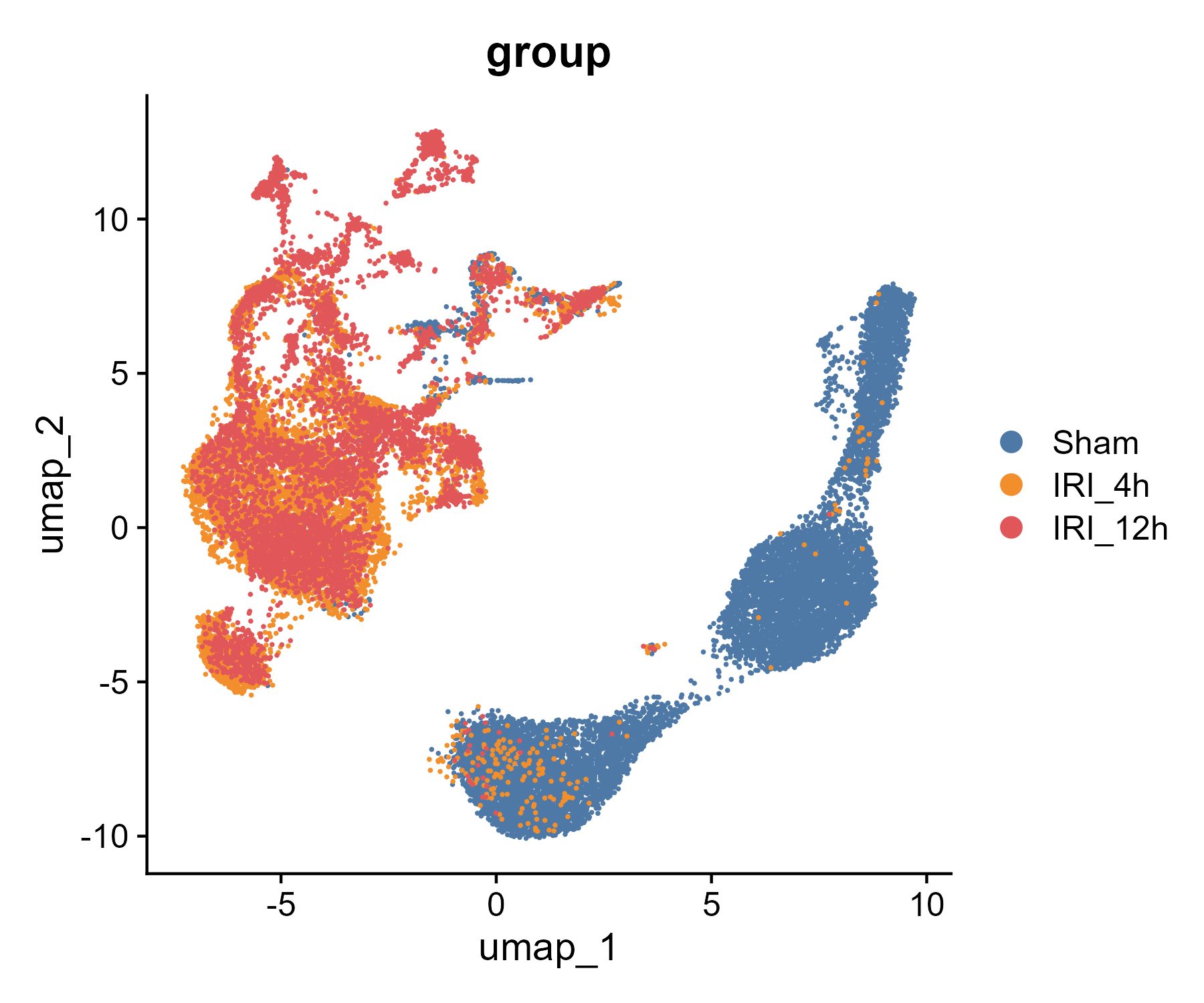


**Figure S3-035. Single-cell transcriptome analysis, step05_PT: 05 PT umap group**


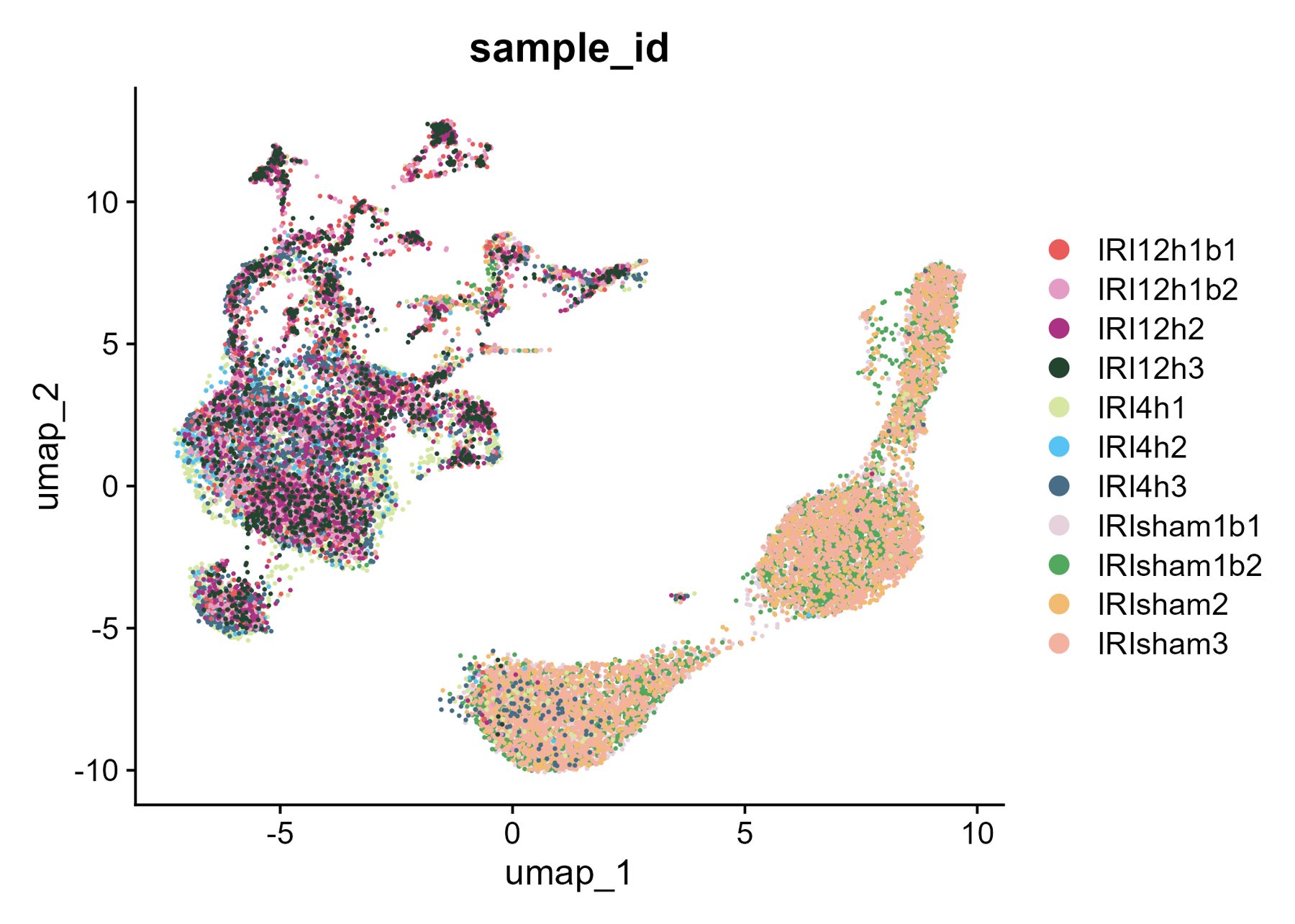


**Figure S3-036. Single-cell transcriptome analysis, step05_PT: 05 PT umap sample**

# Section: step06_PT_state


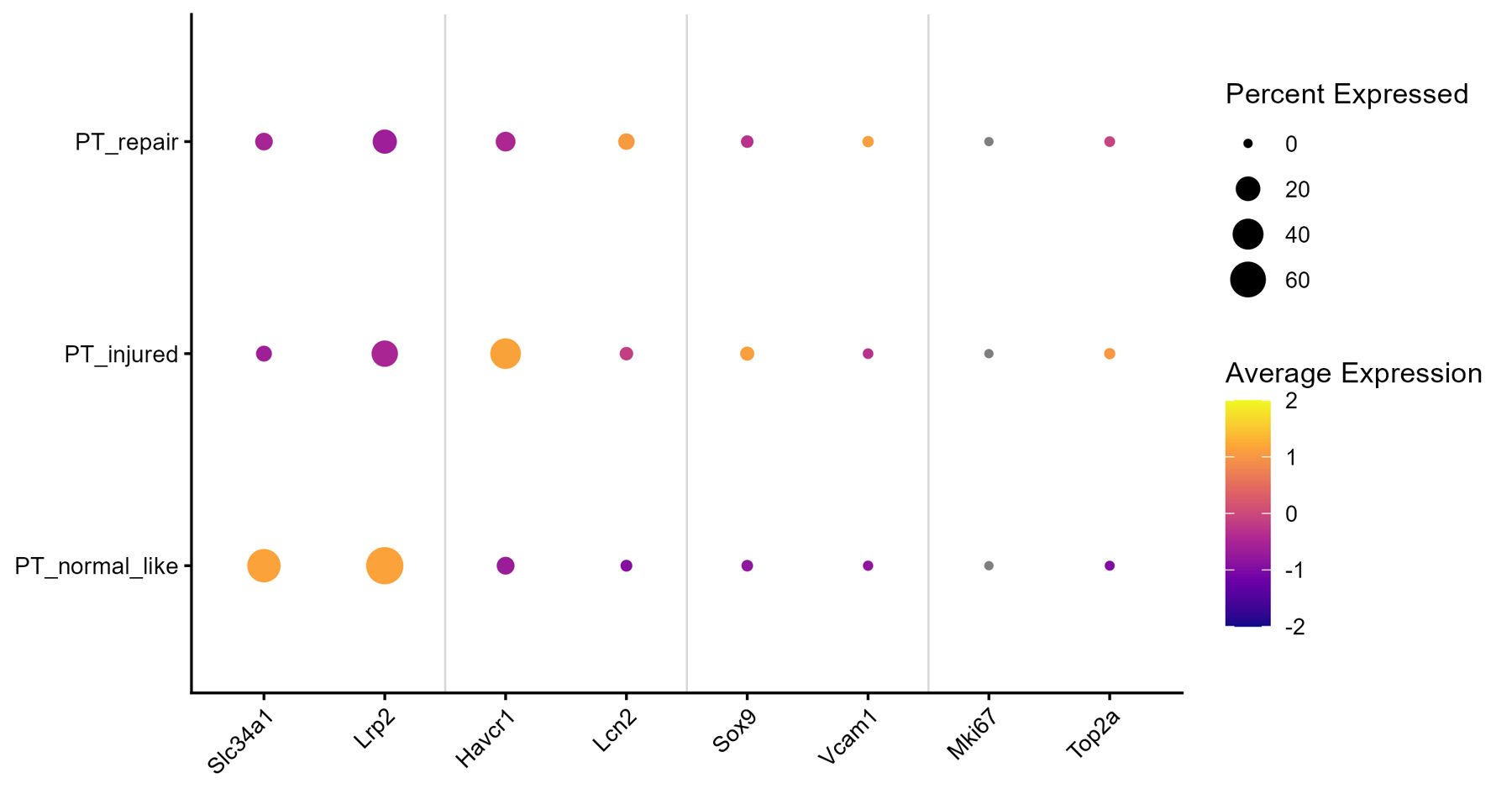


**Figure S3-037. Single-cell transcriptome analysis, step06_PT_state: 06 PT state marker dotplot**


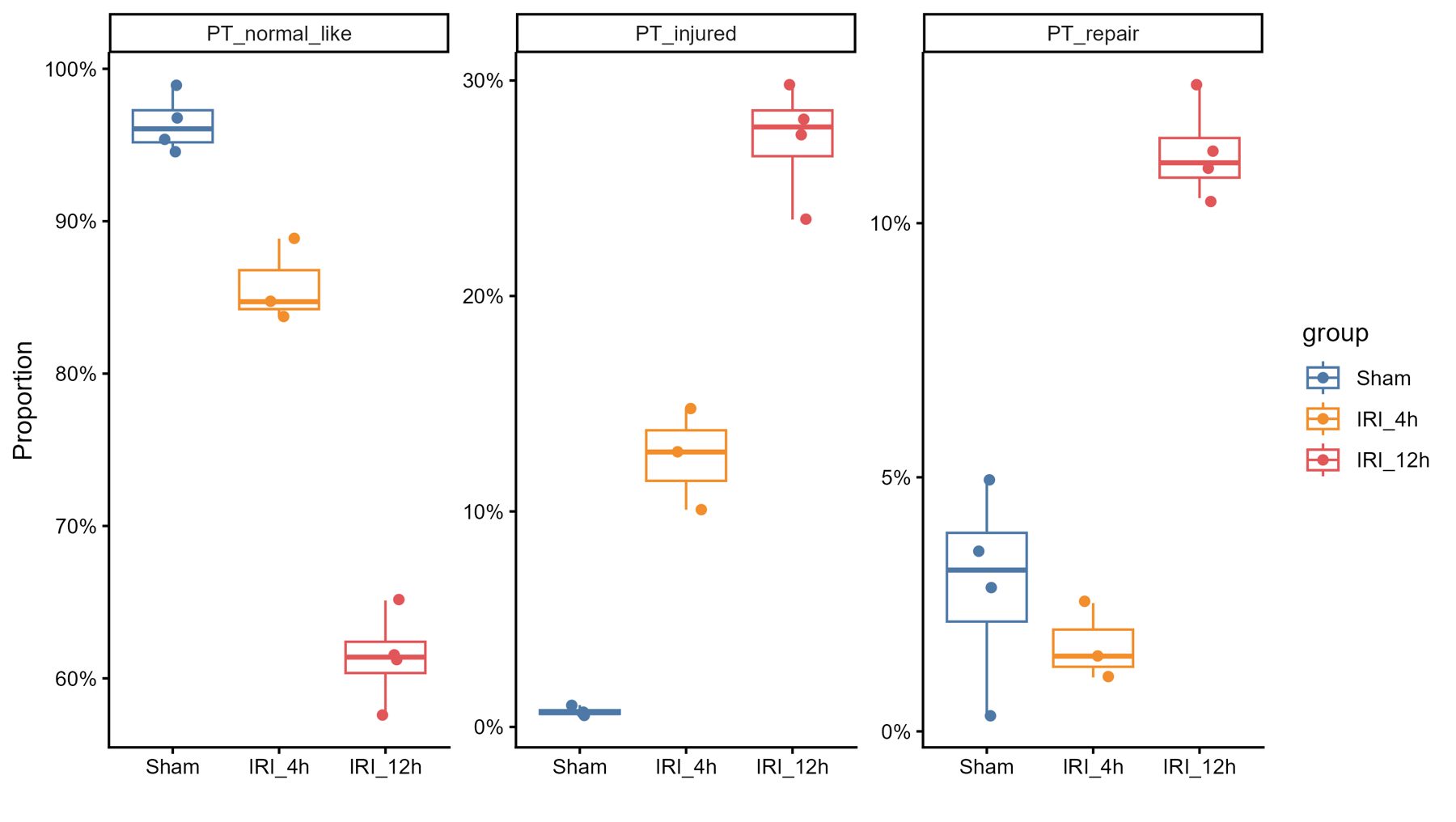


**Figure S3-038. Single-cell transcriptome analysis, step06_PT_state: 06 PT state prop by group boxplot**


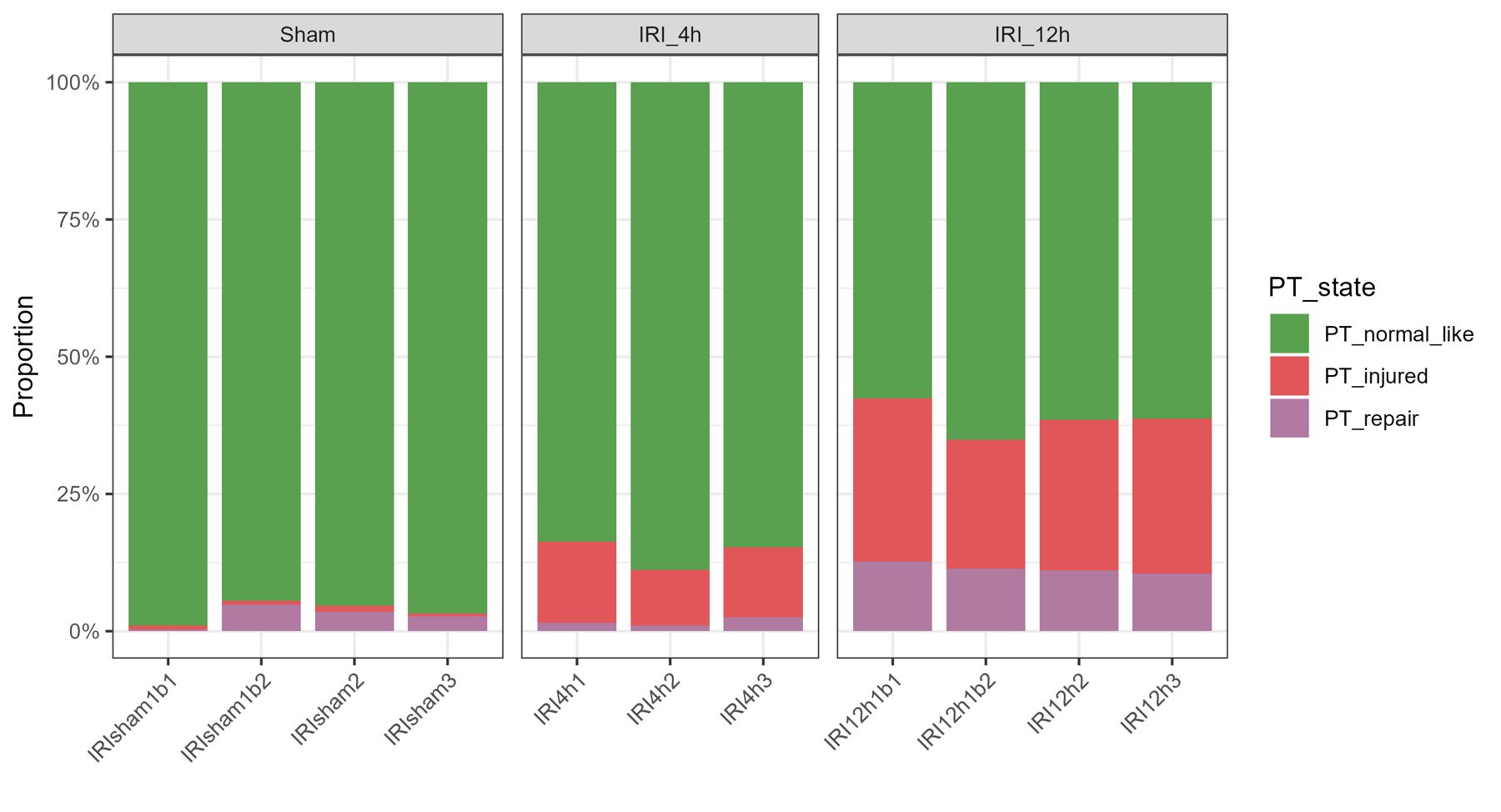


**Figure S3-039. Single-cell transcriptome analysis, step06_PT_state: 06 PT state prop by sample stackedbar**


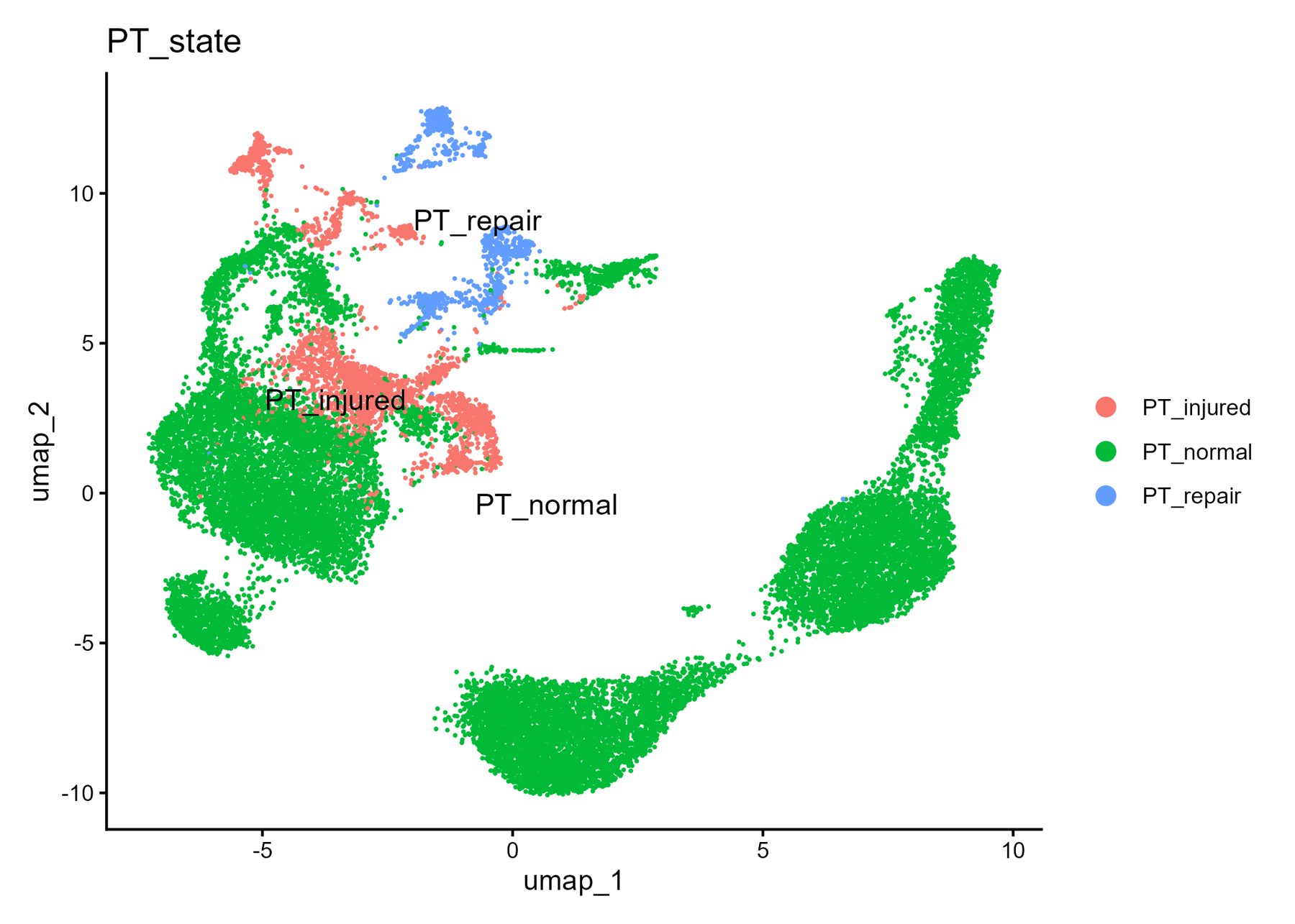


**Figure S3-040. Single-cell transcriptome analysis, step06_PT_state: 06 PT state umap**


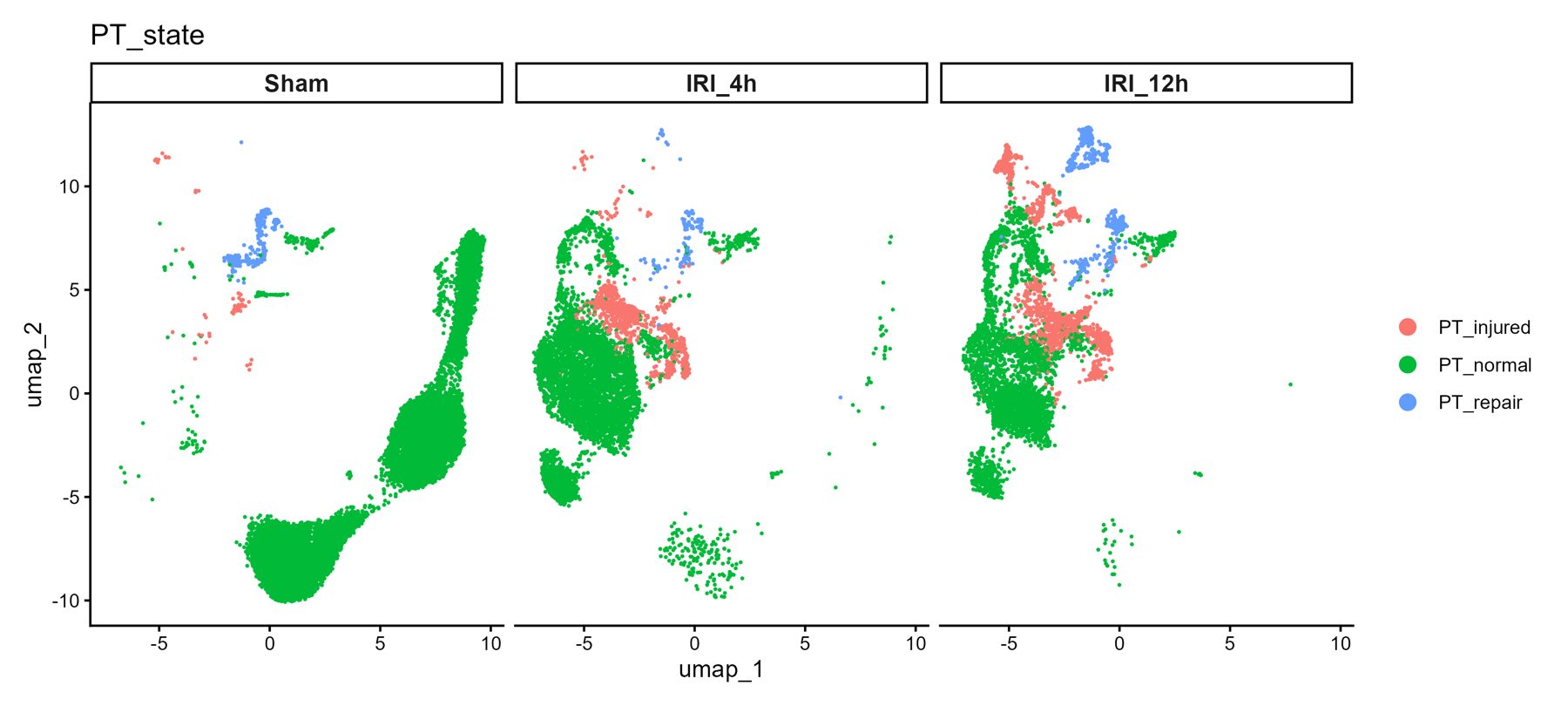


**Figure S3-041. Single-cell transcriptome analysis, step06_PT_state: 06 PT state umap splitby group**


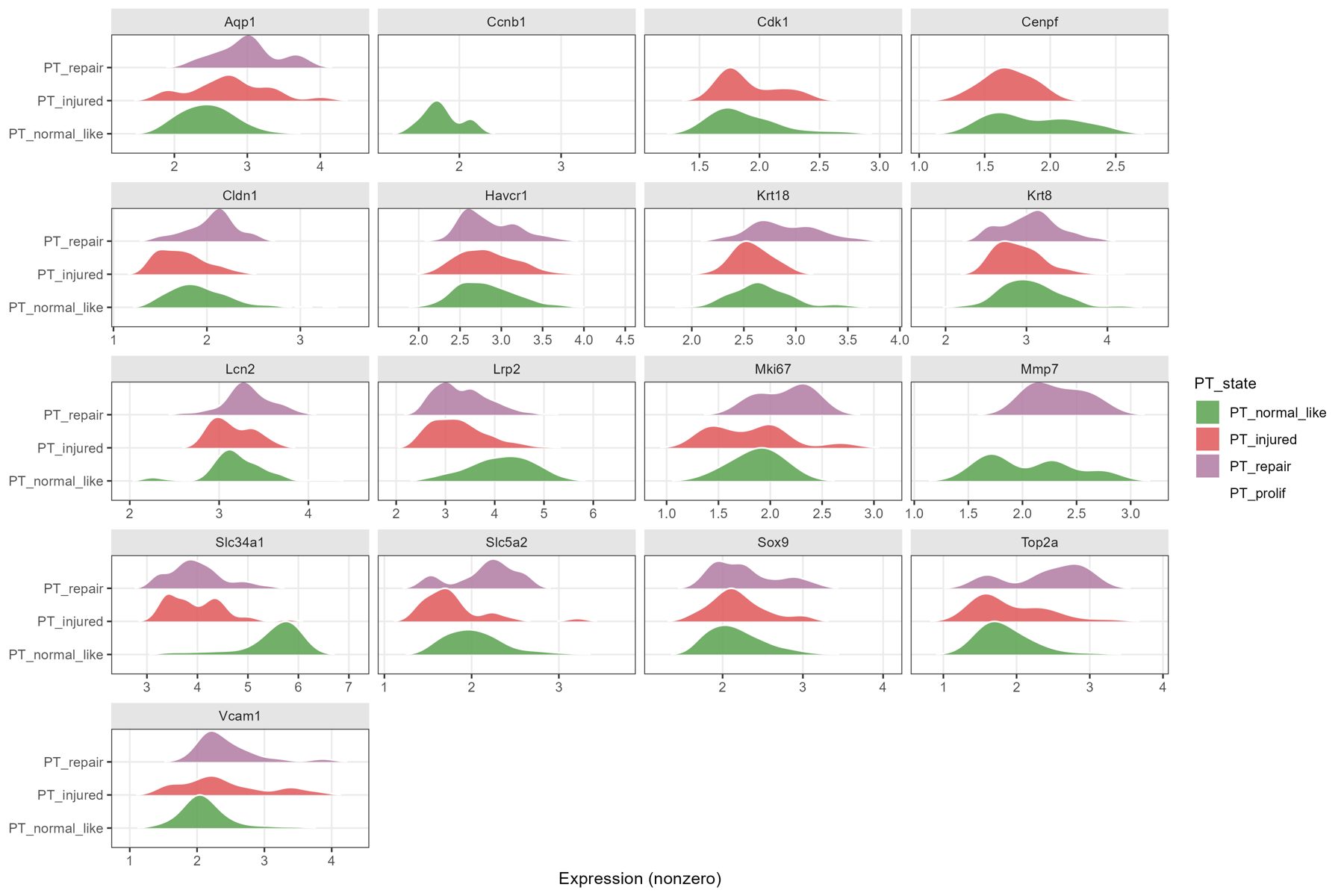


**Figure S3-042. Single-cell transcriptome analysis, step06_PT_state: 06 PT state violin keymarkers**

# Section: step07_injured_ratio


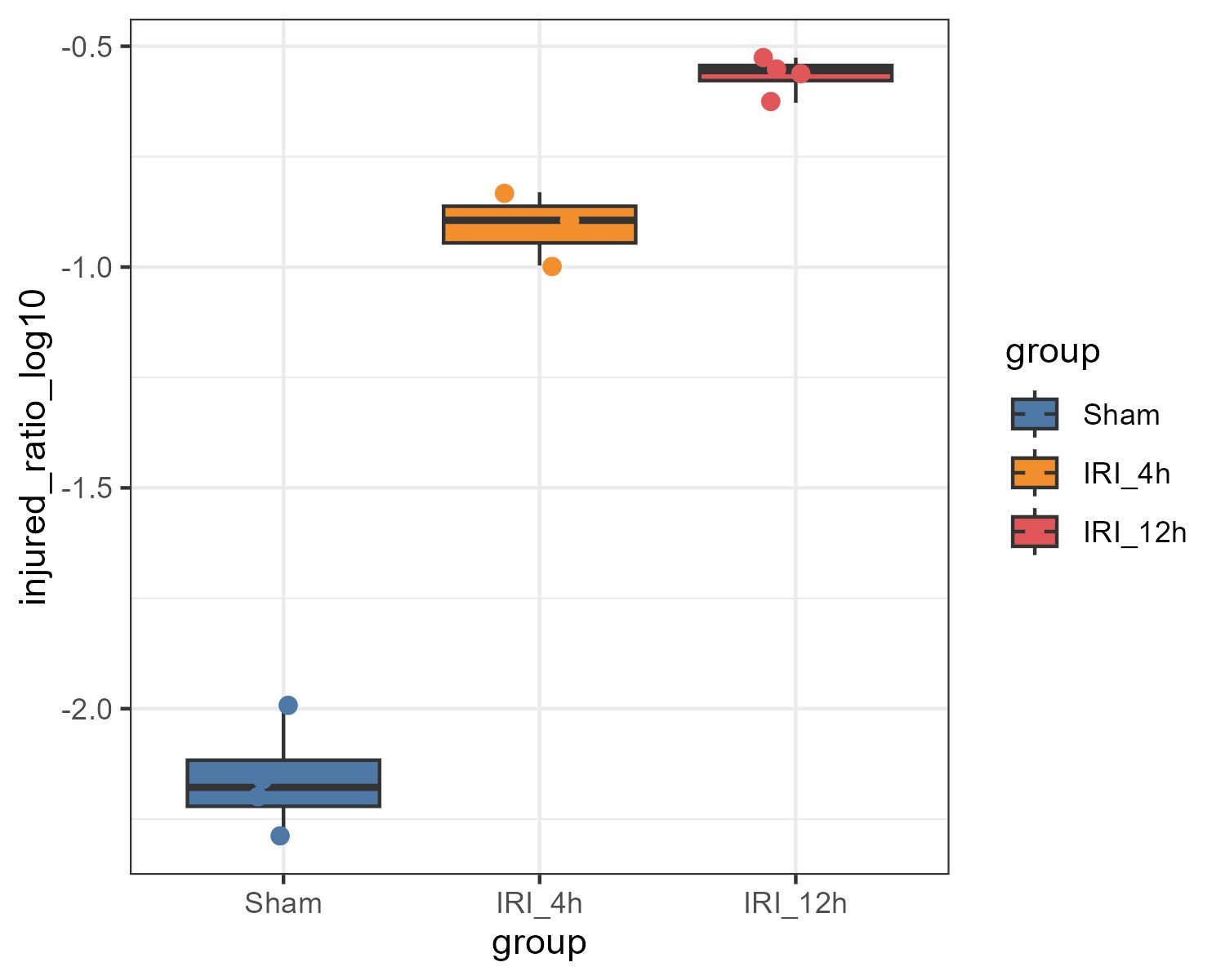


**Figure S3-043. Single-cell transcriptome analysis, step07_injured_ratio: 07 injured ratio log10 point boxplot**


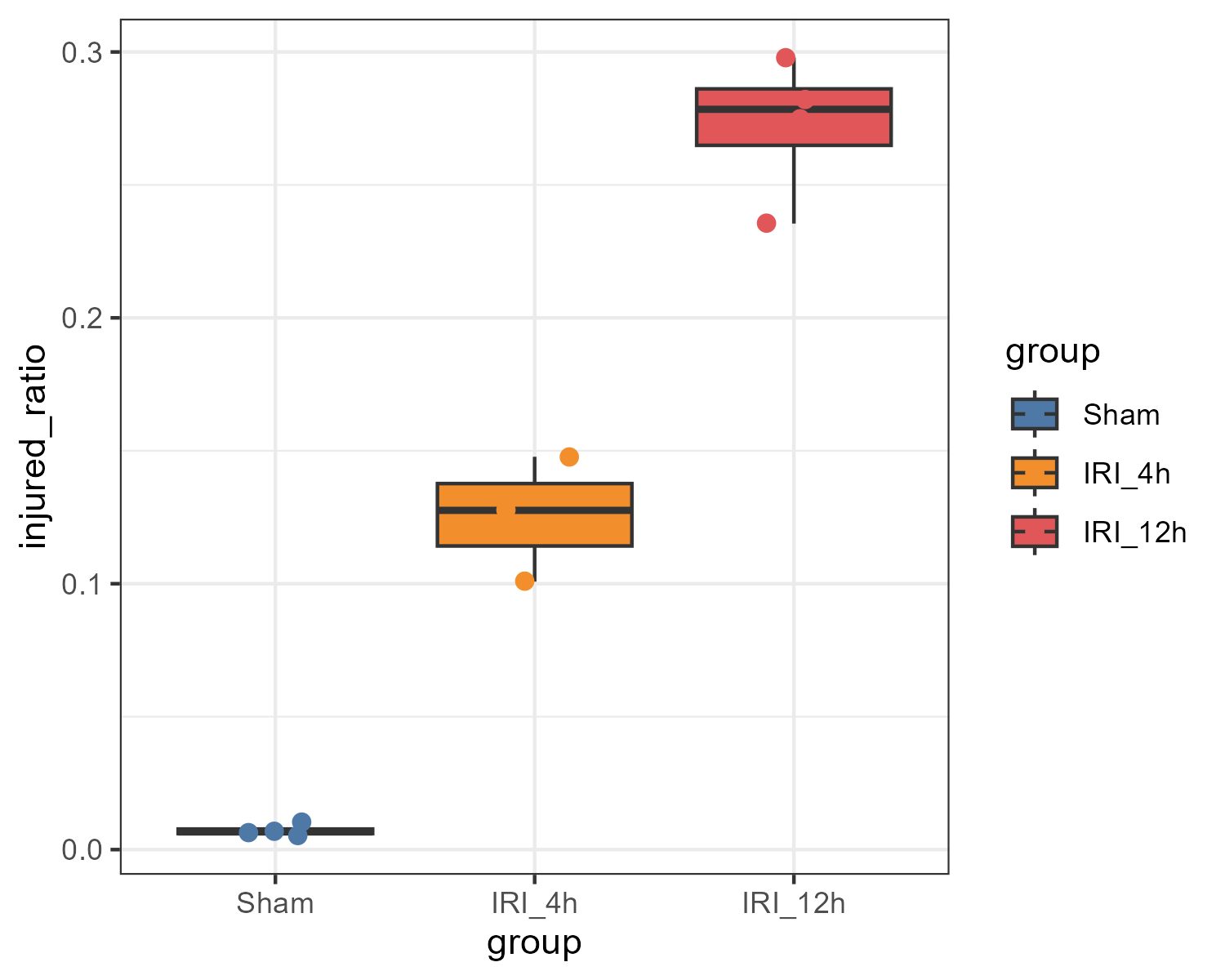


**Figure S3-044. Single-cell transcriptome analysis, step07_injured_ratio: 07 injured ratio point boxplot**


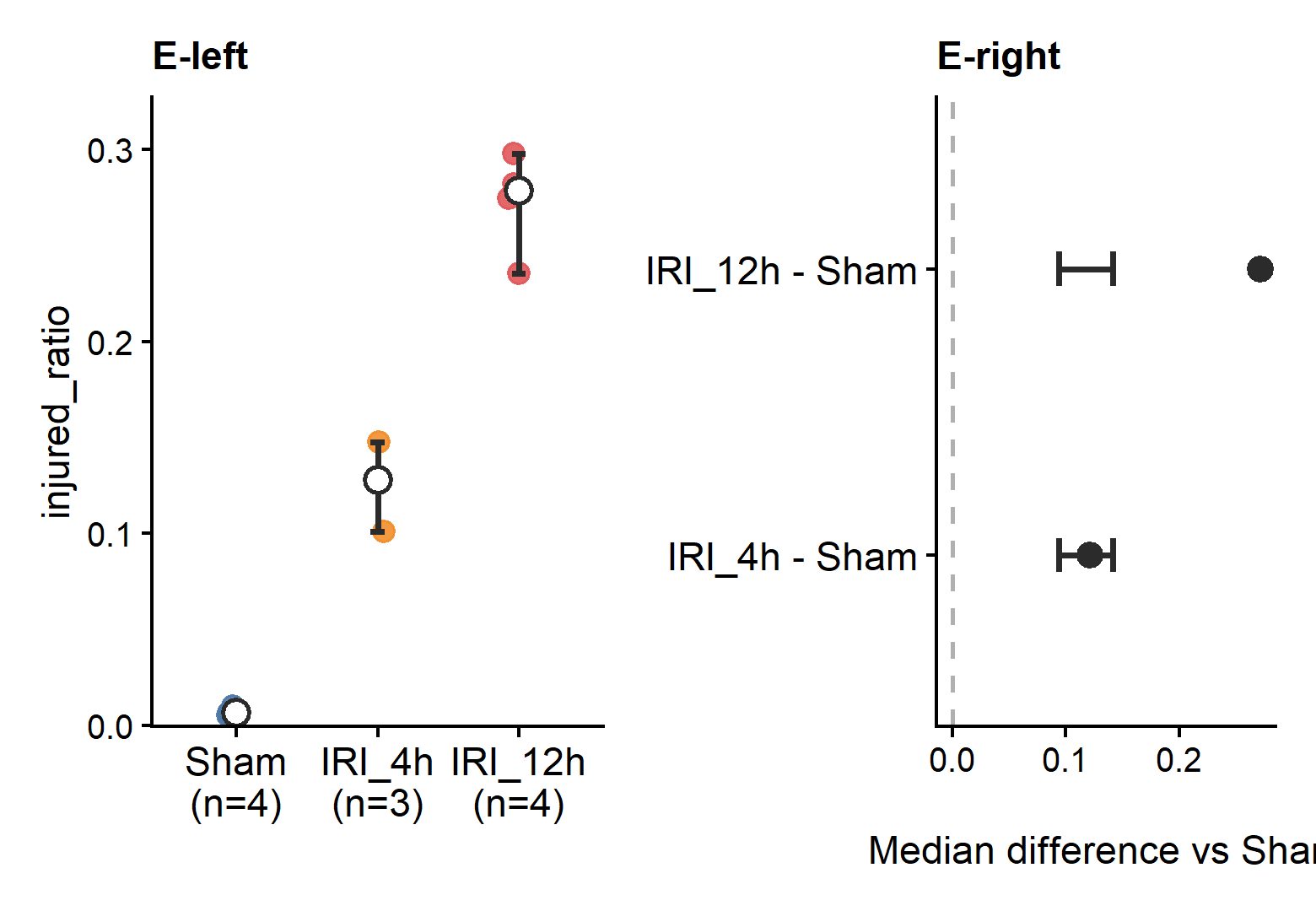


**Figure S3-045. Single-cell transcriptome analysis, step07_injured_ratio: 07 injured ratio point boxplot with CI**

# Section: step08_scores


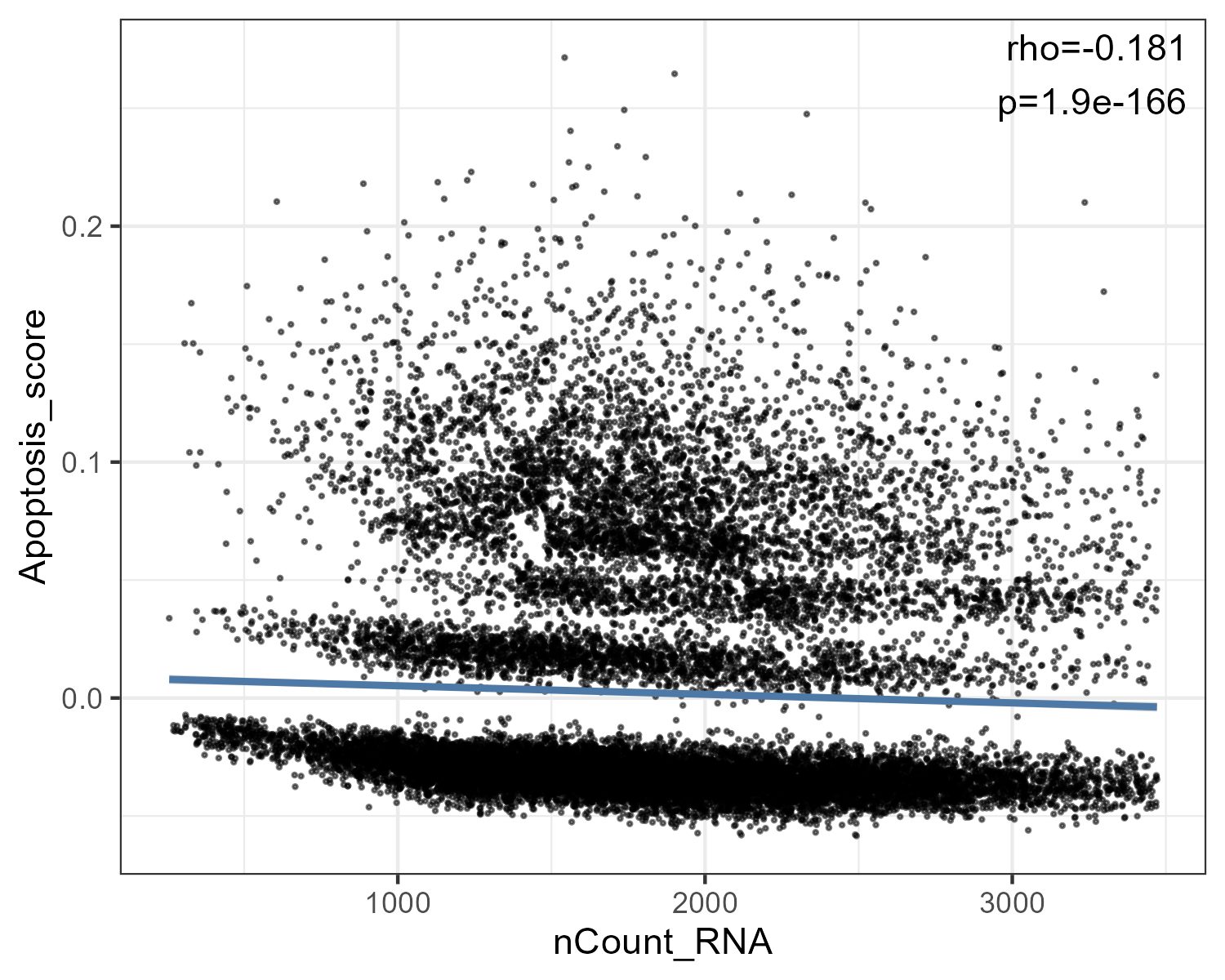


**Figure S3-046. Single-cell transcriptome analysis, step08_scores: 08 Apop score vs nCount**


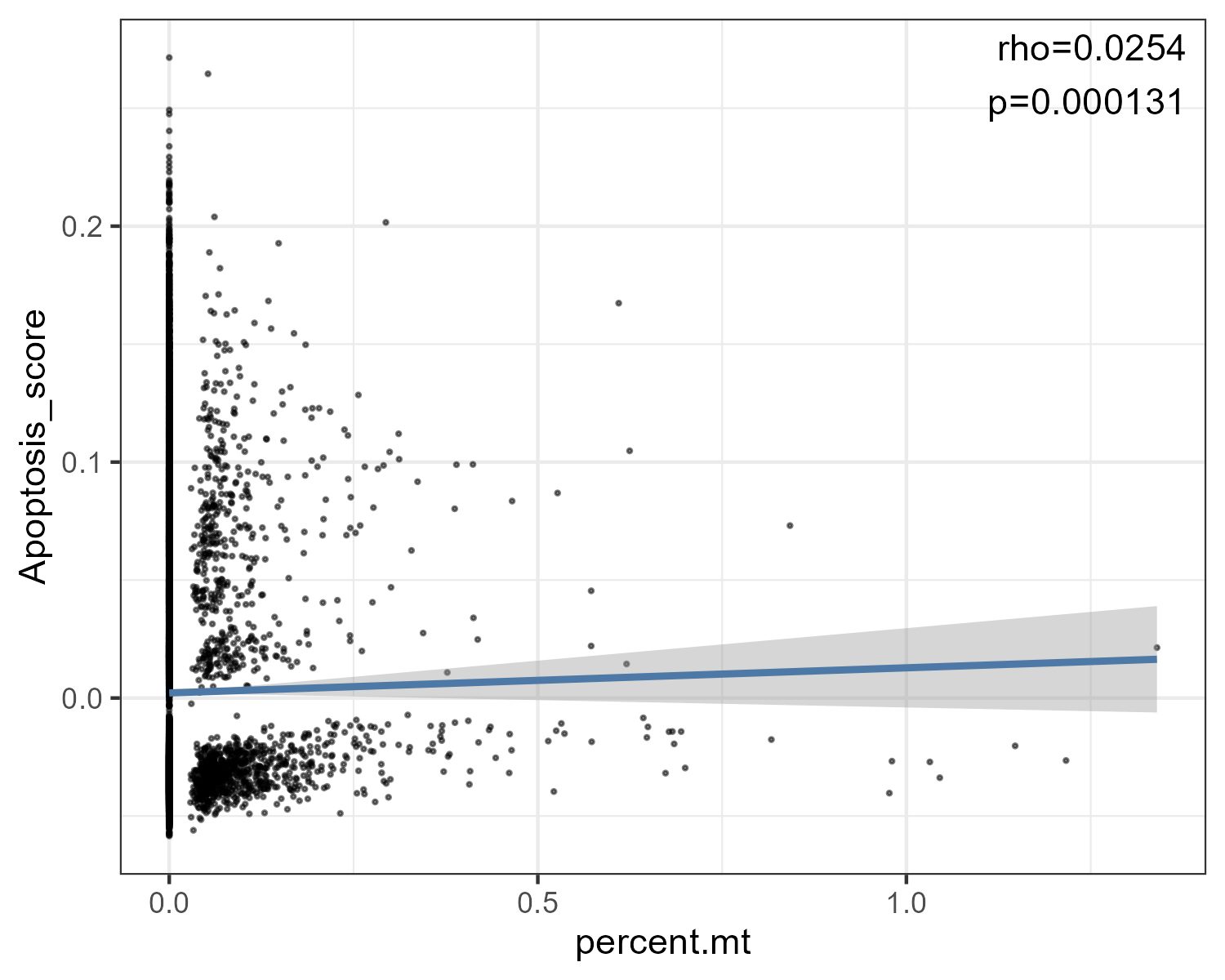


**Figure S3-047. Single-cell transcriptome analysis, step08_scores: 08 Apop score vs percentmt**


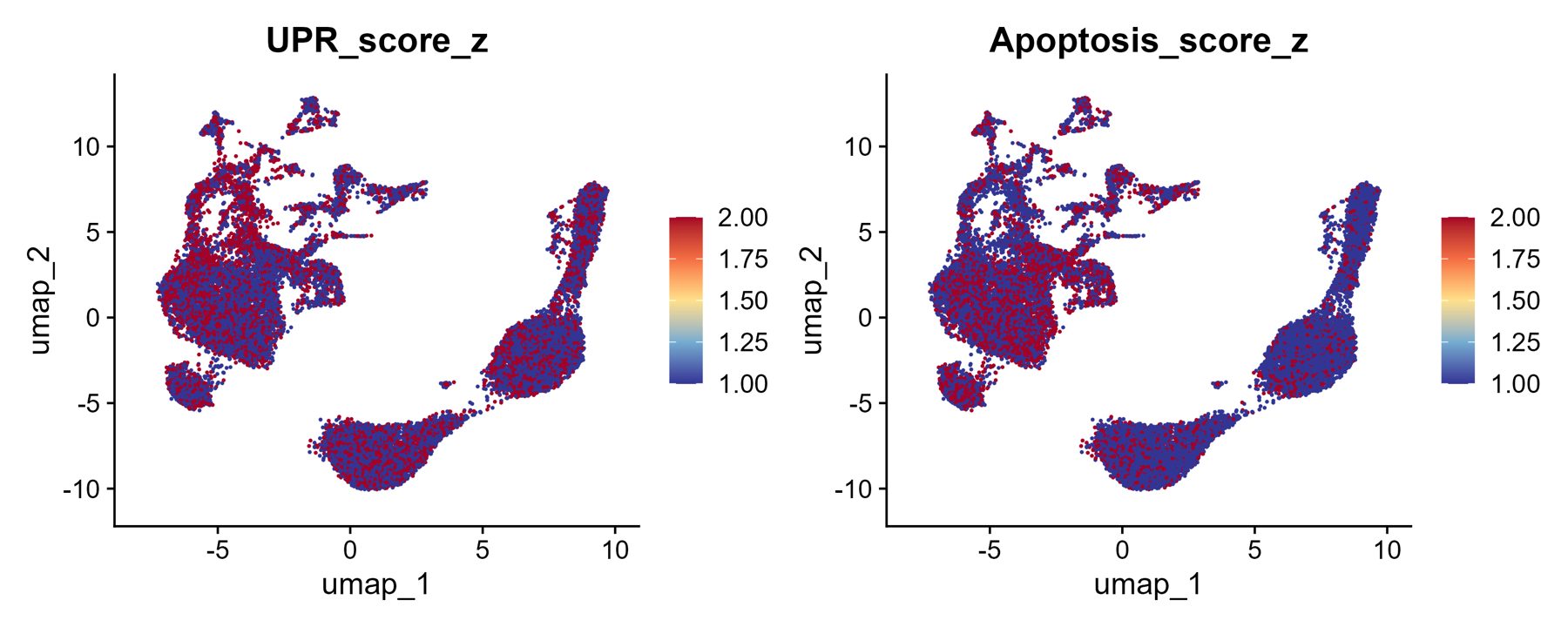


**Figure S3-048. Single-cell transcriptome analysis, step08_scores: 08 celllevel score featureplot on PT umap**


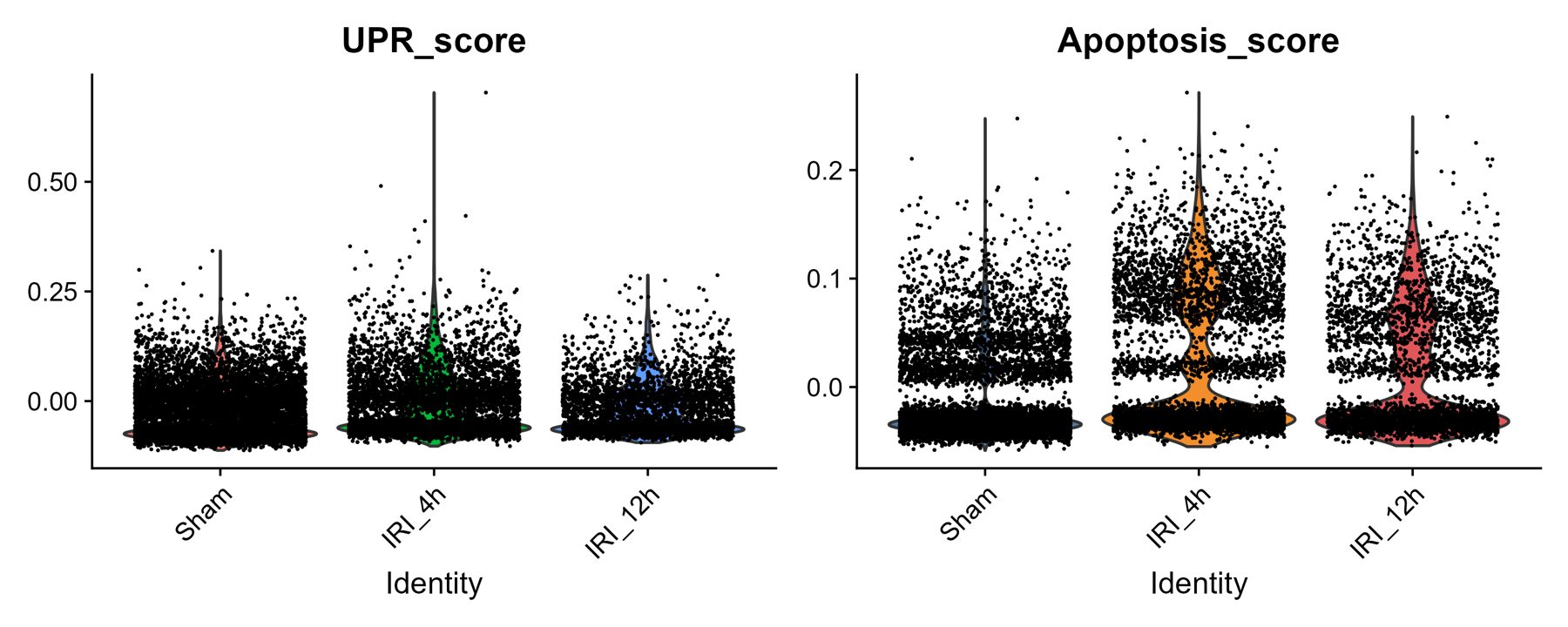


**Figure S3-049. Single-cell transcriptome analysis, step08_scores: 08 celllevel score PTnormal like splitby group**


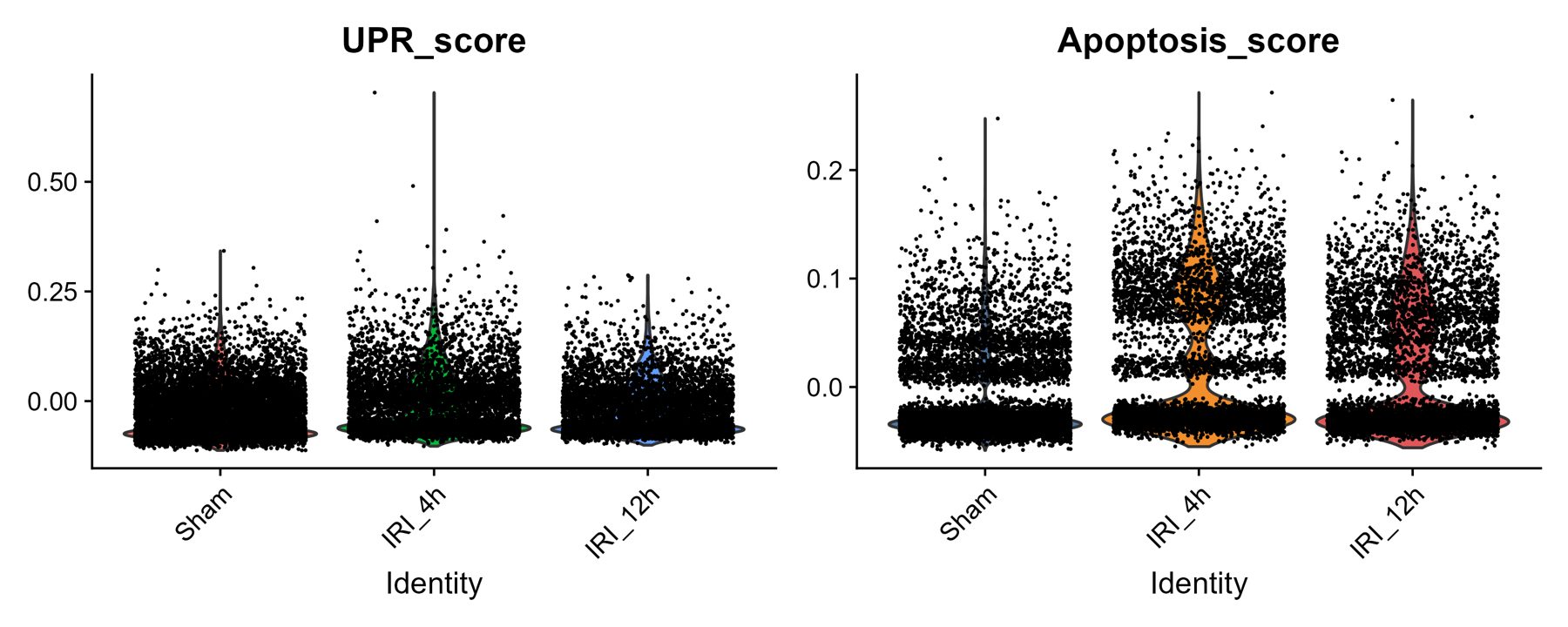


**Figure S3-050. Single-cell transcriptome analysis, step08_scores: 08 celllevel score violin by group**


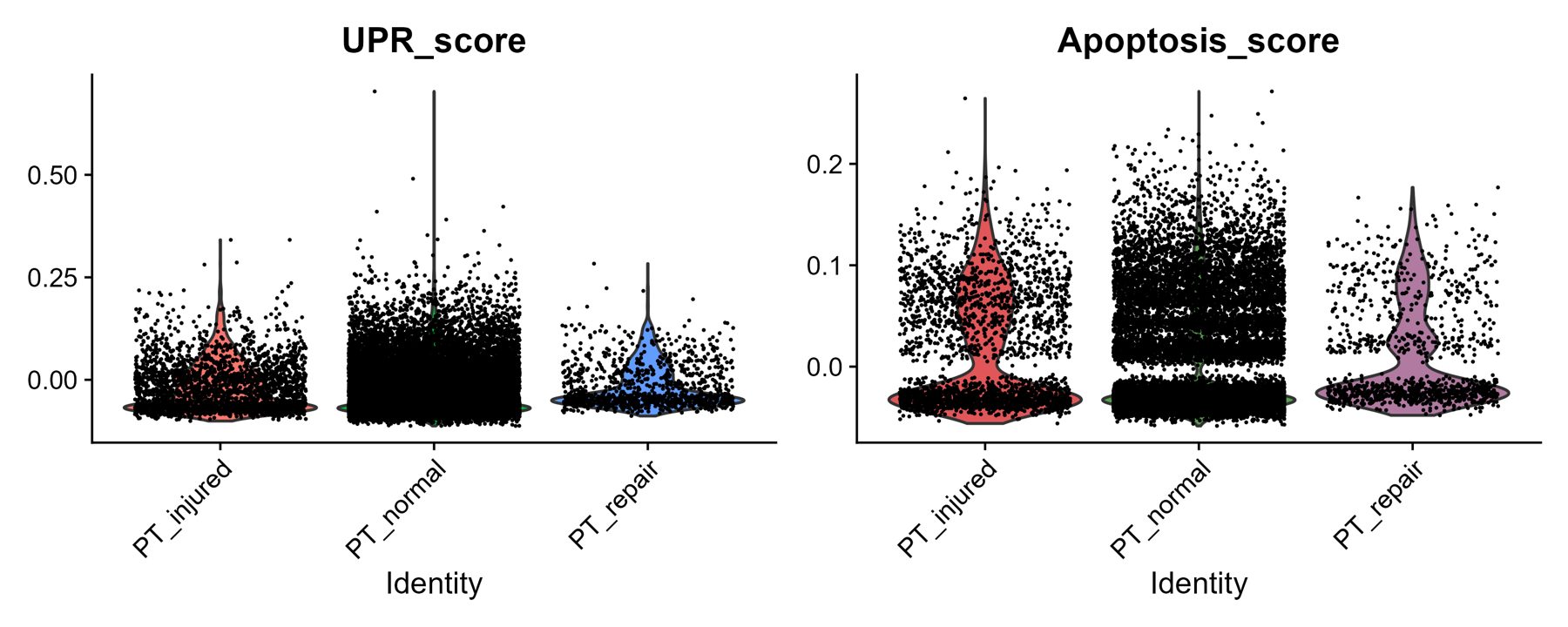


**Figure S3-051. Single-cell transcriptome analysis, step08_scores: 08 celllevel score violin by PTstate**


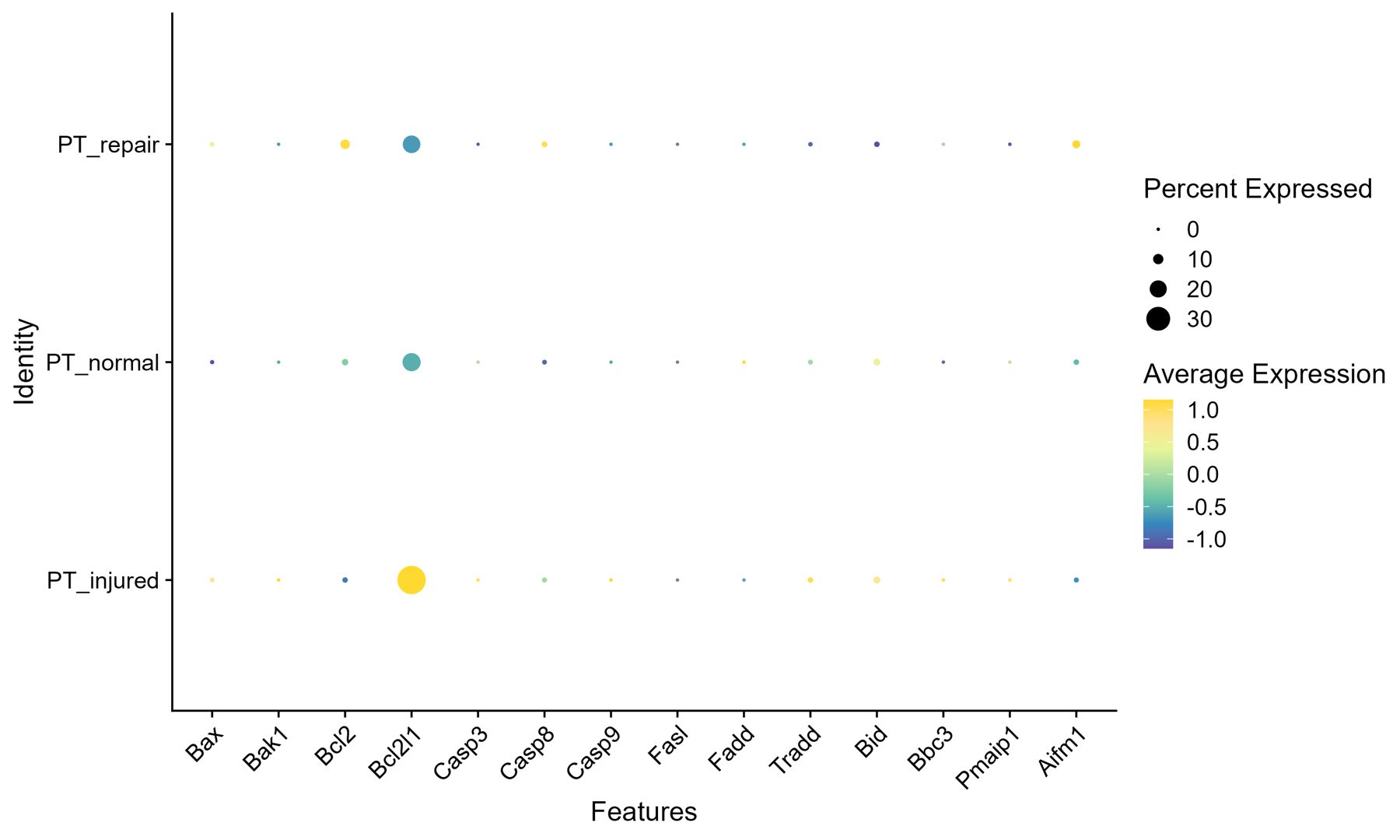


**Figure S3-052. Single-cell transcriptome analysis, step08_scores: 08 PT Apop markers dotplot**


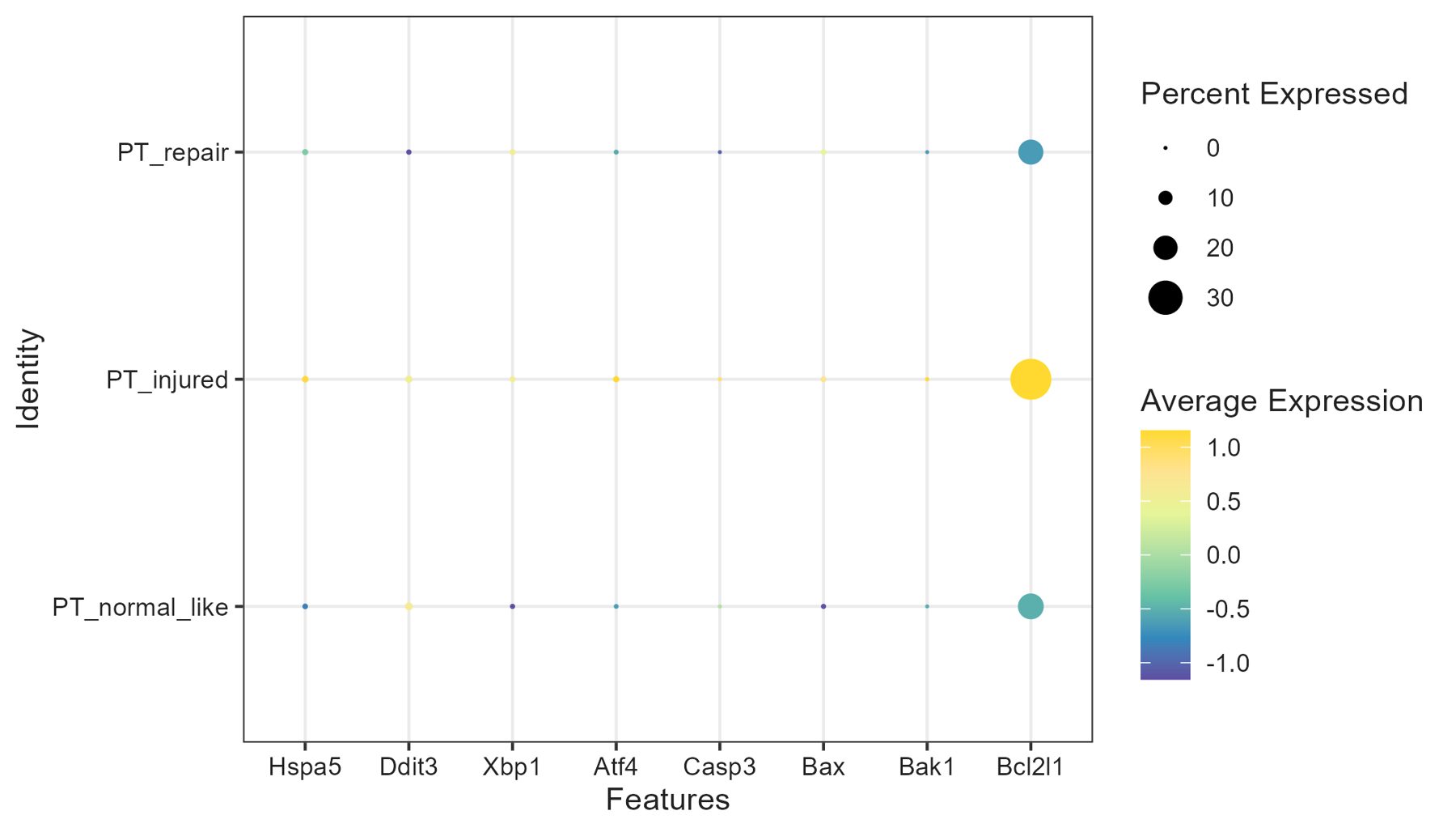


**Figure S3-053. Single-cell transcriptome analysis, step08_scores: 08 PT keygenes dotplot**


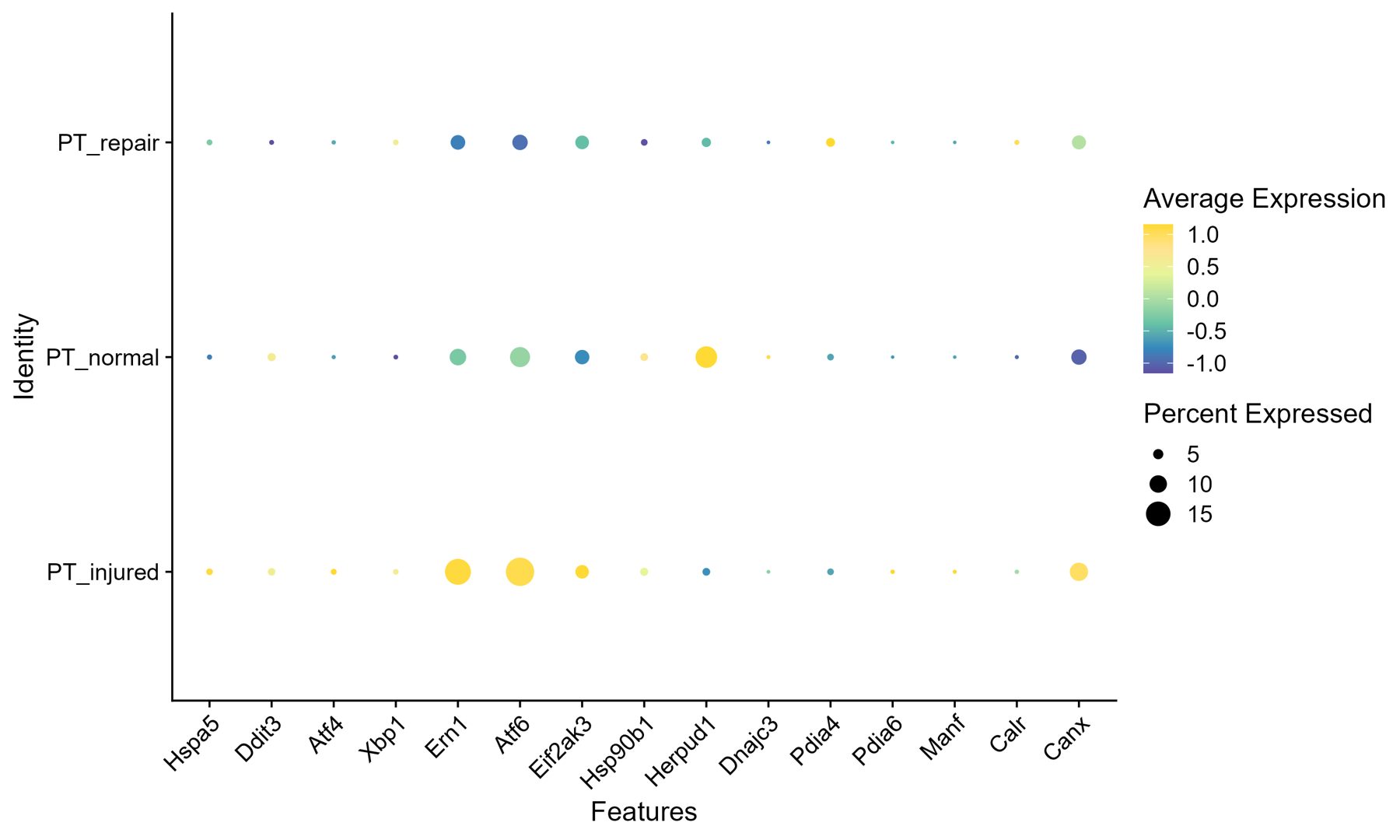


**Figure S3-054. Single-cell transcriptome analysis, step08_scores: 08 PT UPR markers dotplot**


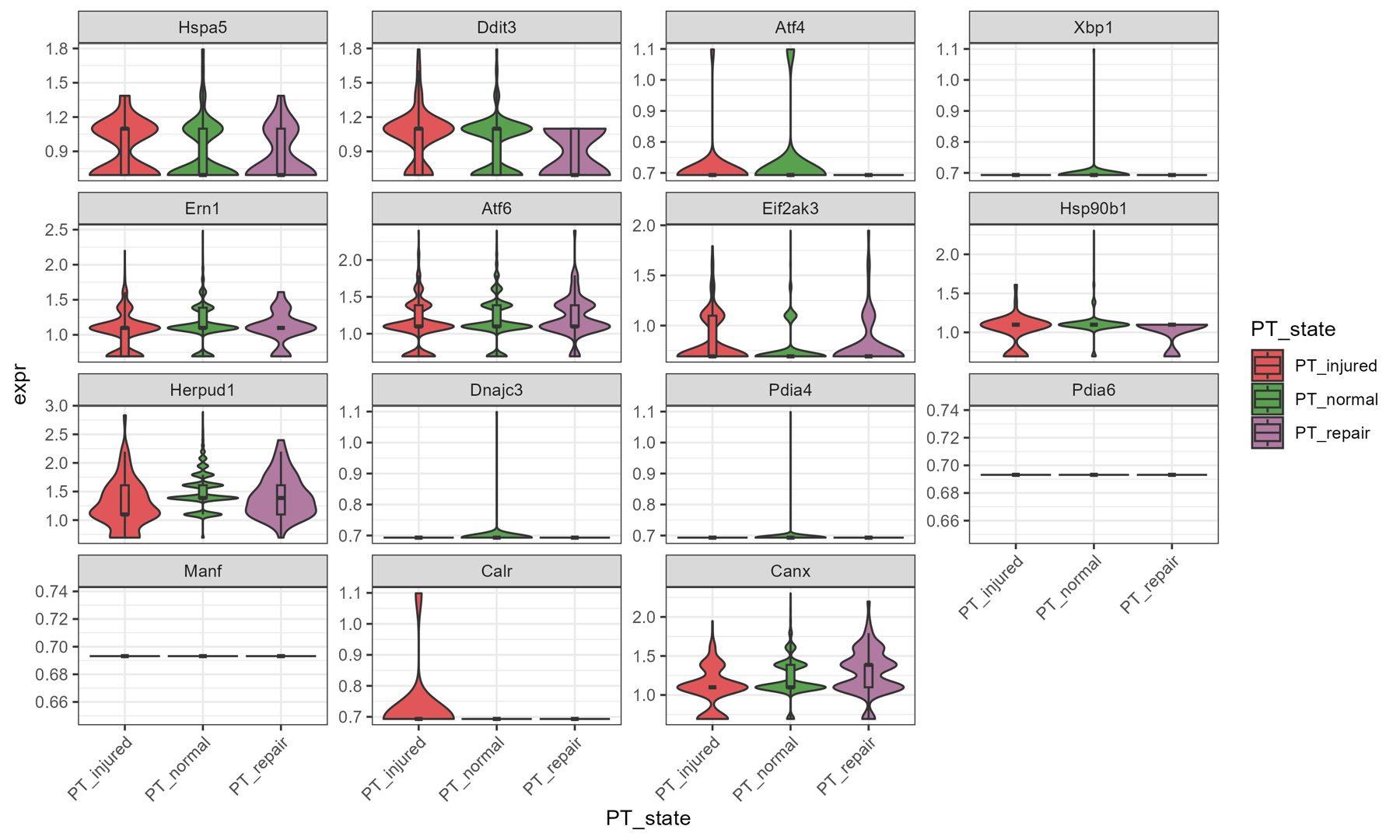


**Figure S3-055. Single-cell transcriptome analysis, step08_scores: 08 PT UPR markers violin nonzero**


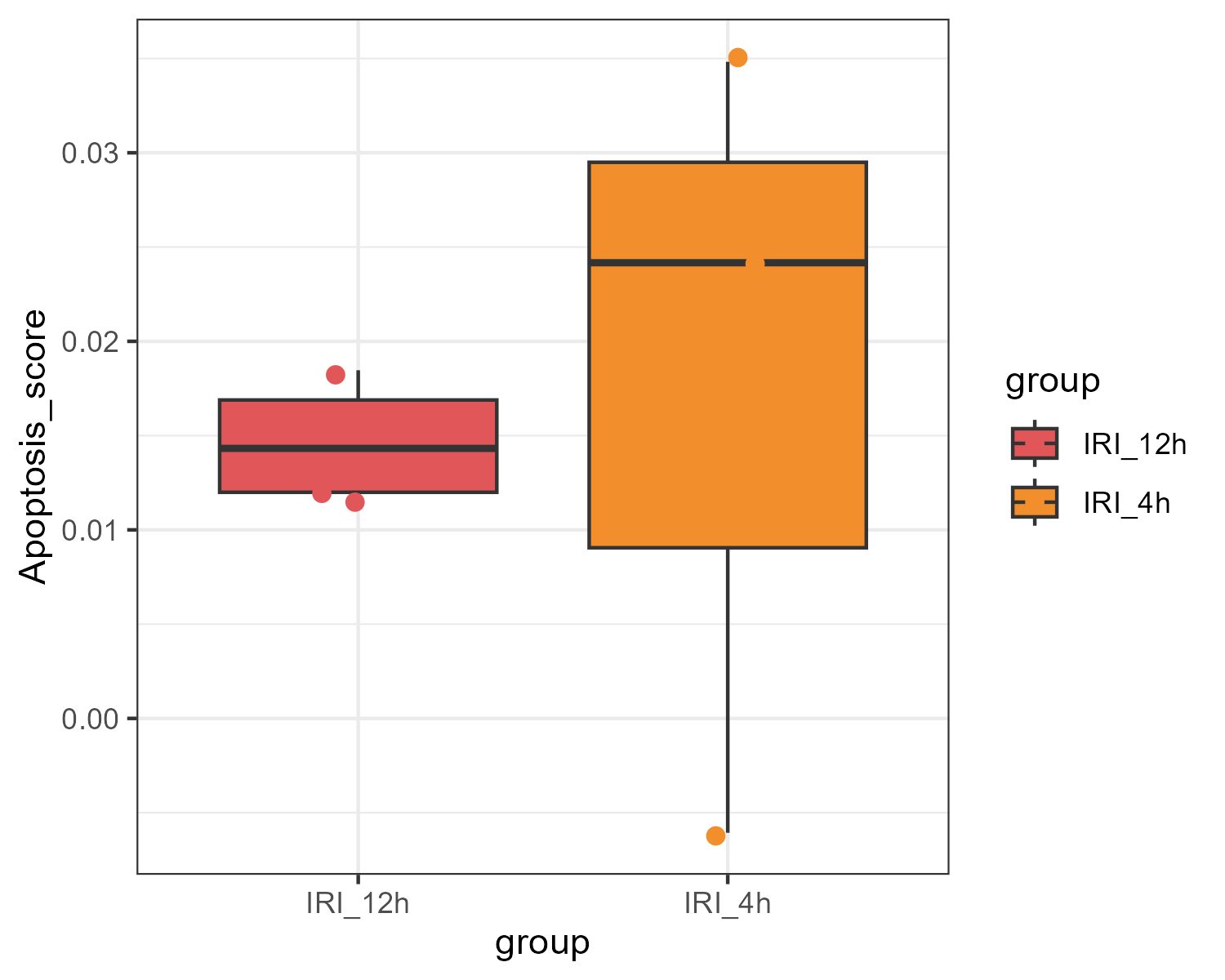


**Figure S3-056. Single-cell transcriptome analysis, step08_scores: 08 samplelevel Apop PTinjured within IRI**


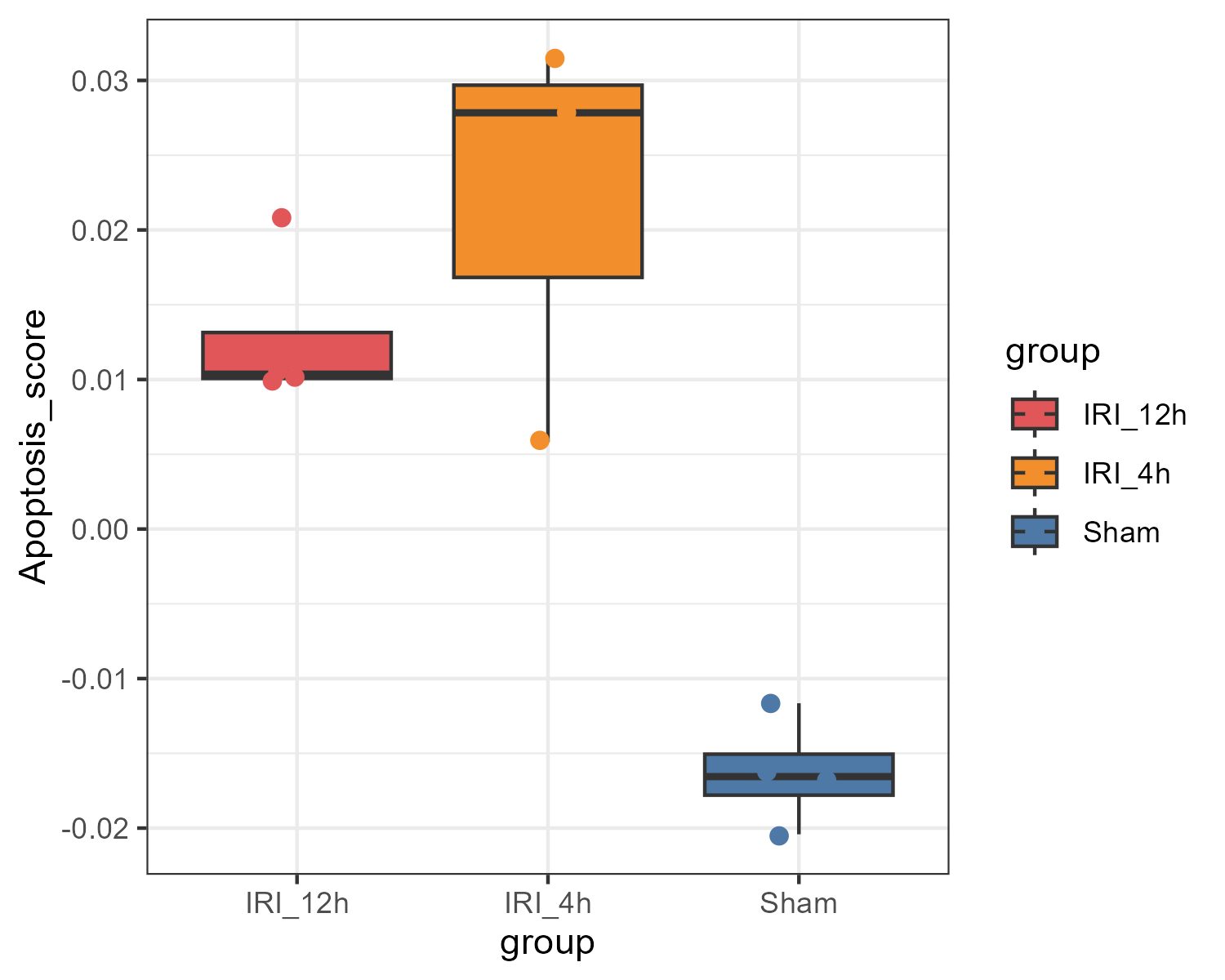


**Figure S3-057. Single-cell transcriptome analysis, step08_scores: 08 samplelevel Apop PTnormal by group**


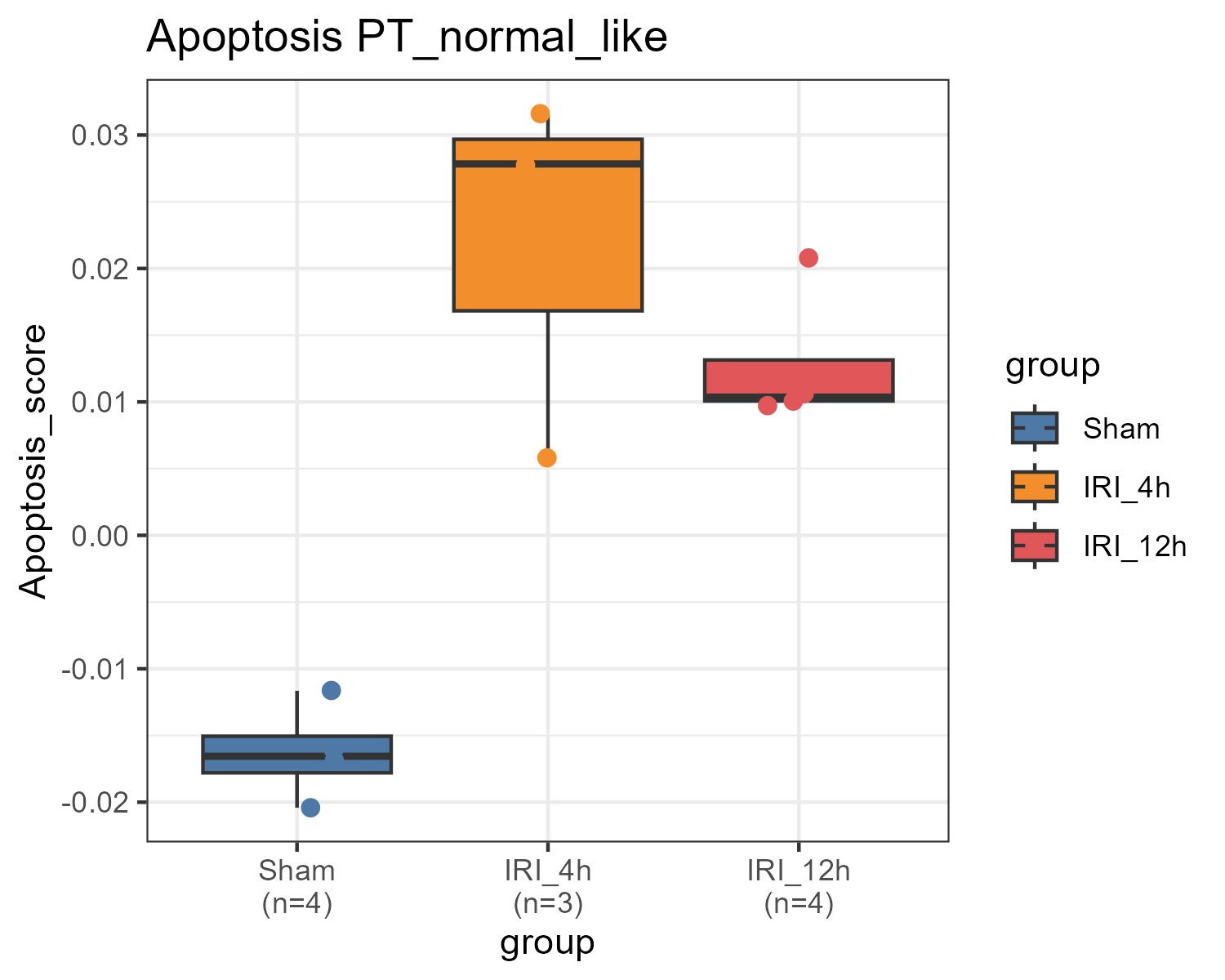


**Figure S3-058. Single-cell transcriptome analysis, step08_scores: 08 samplelevel Apop PTnormal like by group**


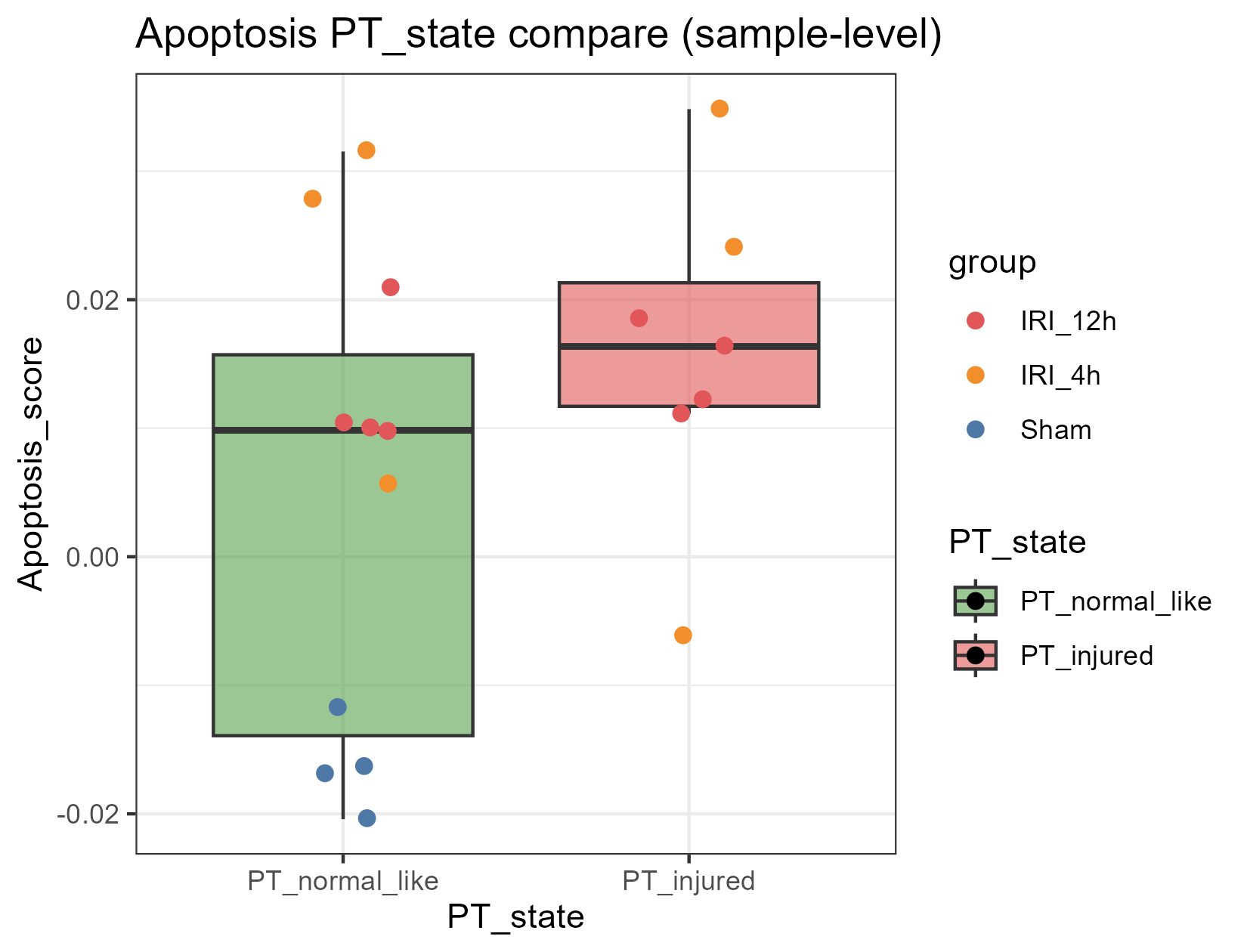


**Figure S3-059. Single-cell transcriptome analysis, step08_scores: 08 samplelevel Apop PTstate compare**


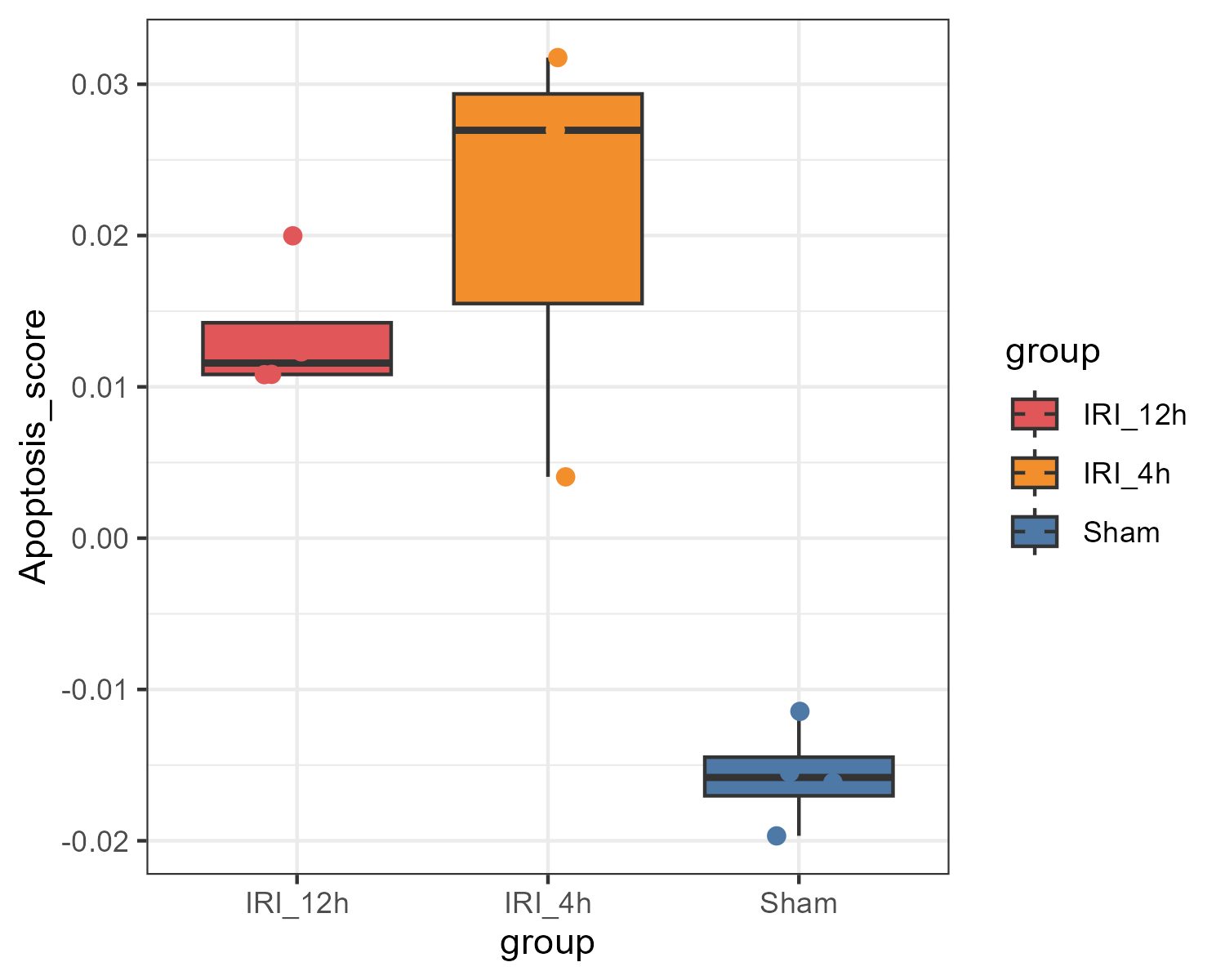


**Figure S3-060. Single-cell transcriptome analysis, step08_scores: 08 samplelevel Apop PTtotal by group**


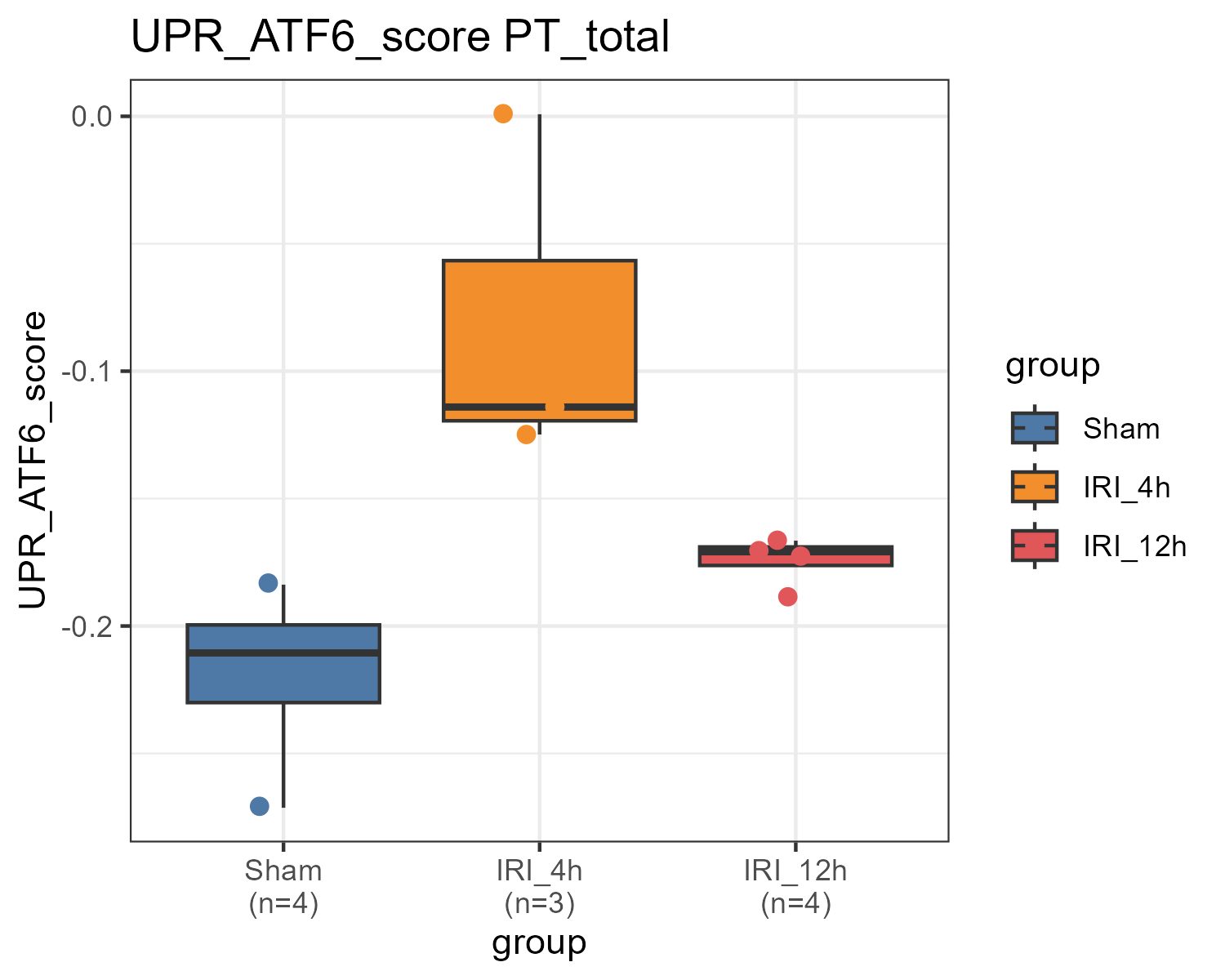


**Figure S3-061. Single-cell transcriptome analysis, step08_scores: 08 samplelevel UPR ATF6 score PTtotal by group**


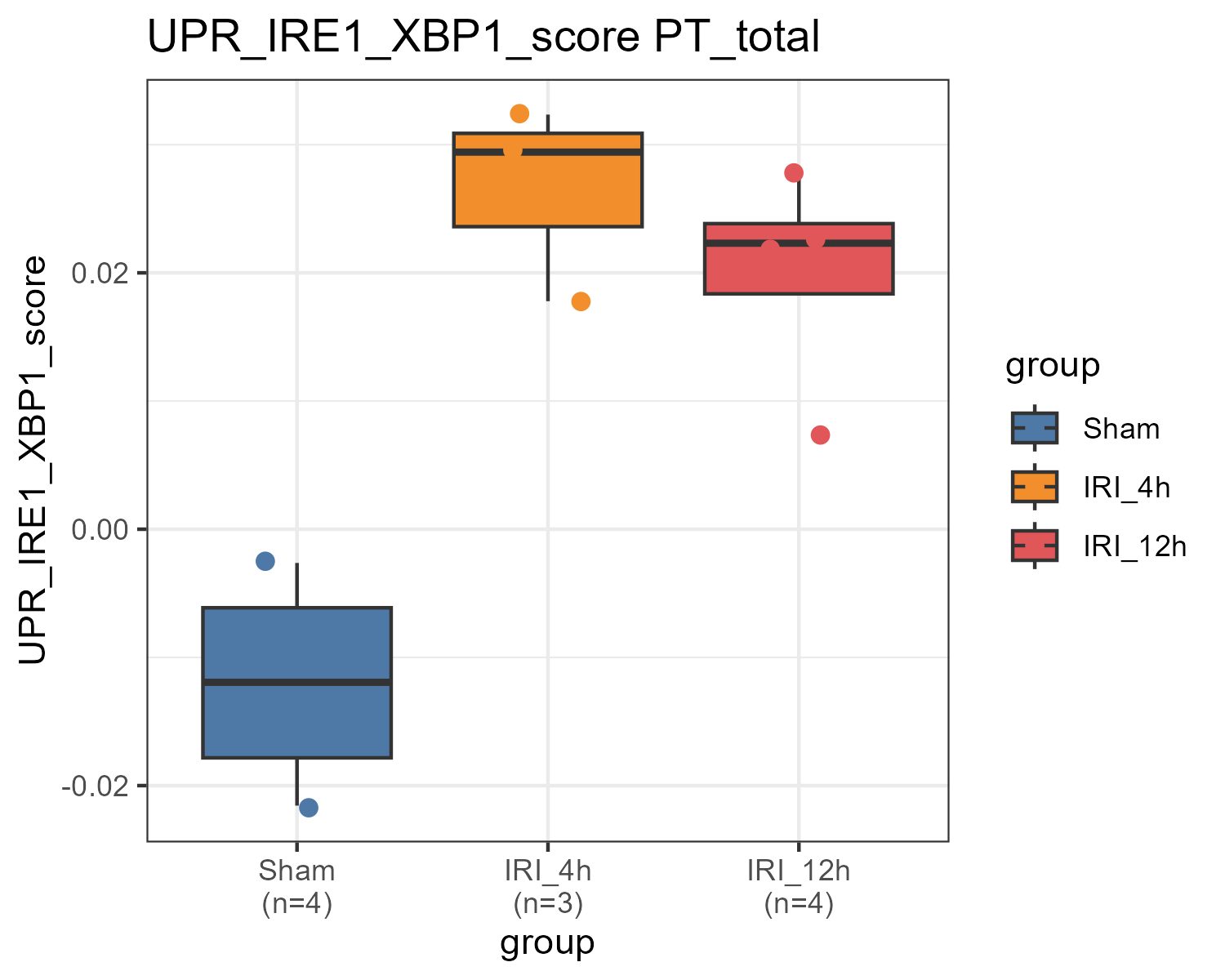


**Figure S3-062. Single-cell transcriptome analysis, step08_scores: 08 samplelevel UPR IRE1 XBP1 score PTtotal by group**


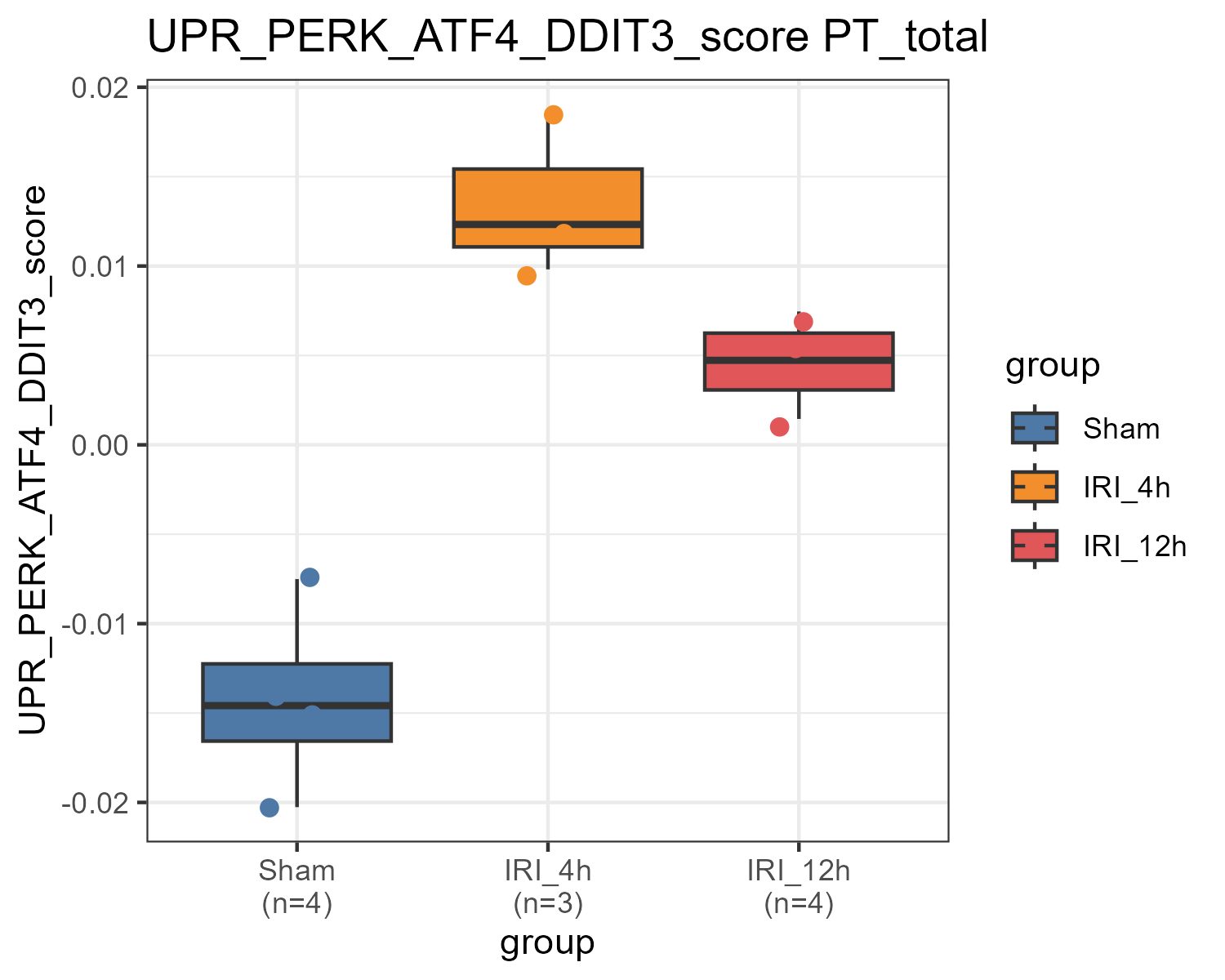


**Figure S3-063. Single-cell transcriptome analysis, step08_scores: 08 samplelevel UPR PERK ATF4 DDIT3 score PTtotal by group**


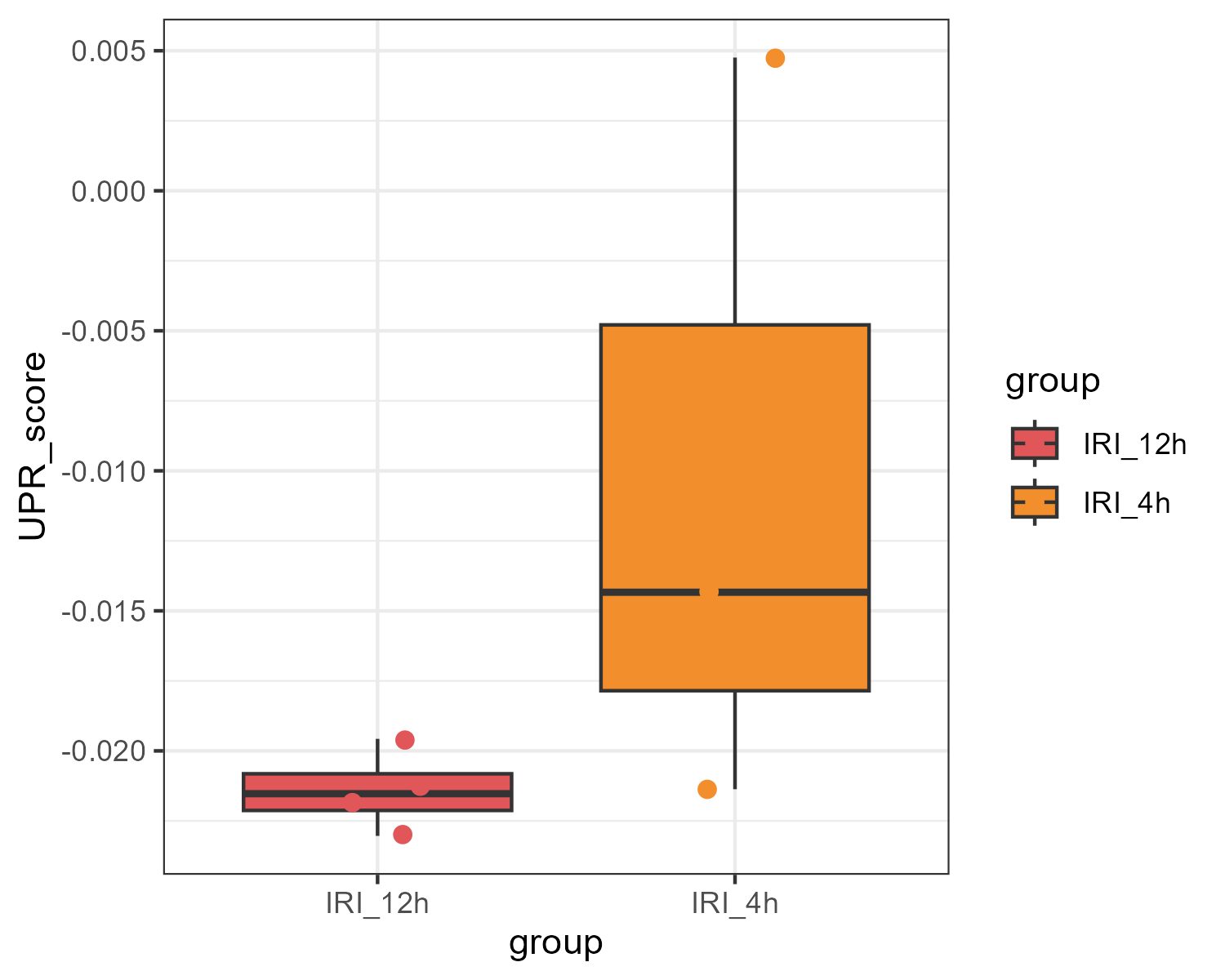


**Figure S3-064. Single-cell transcriptome analysis, step08_scores: 08 samplelevel UPR PTinjured within IRI**


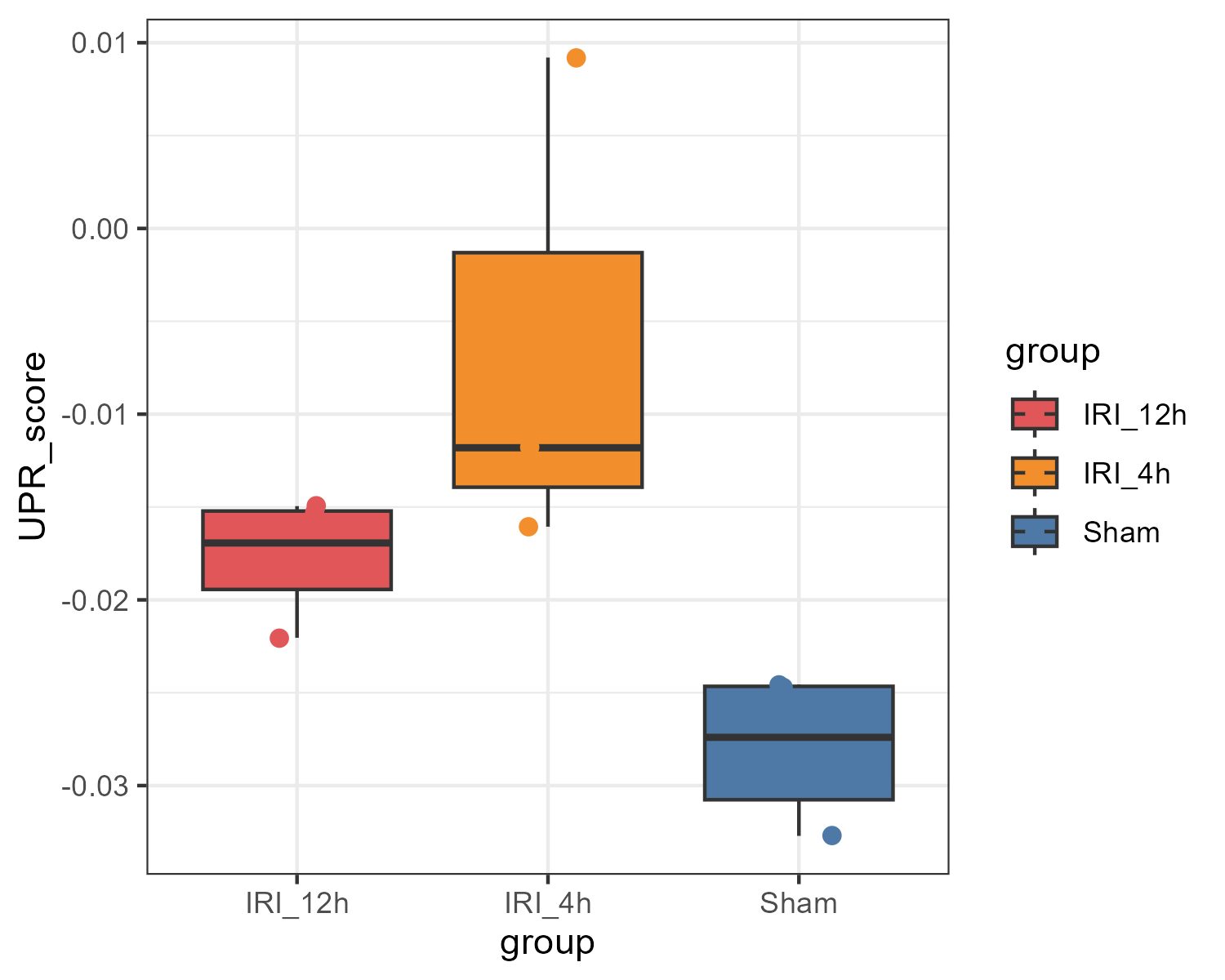


**Figure S3-065. Single-cell transcriptome analysis, step08_scores: 08 samplelevel UPR PTnormal by group**


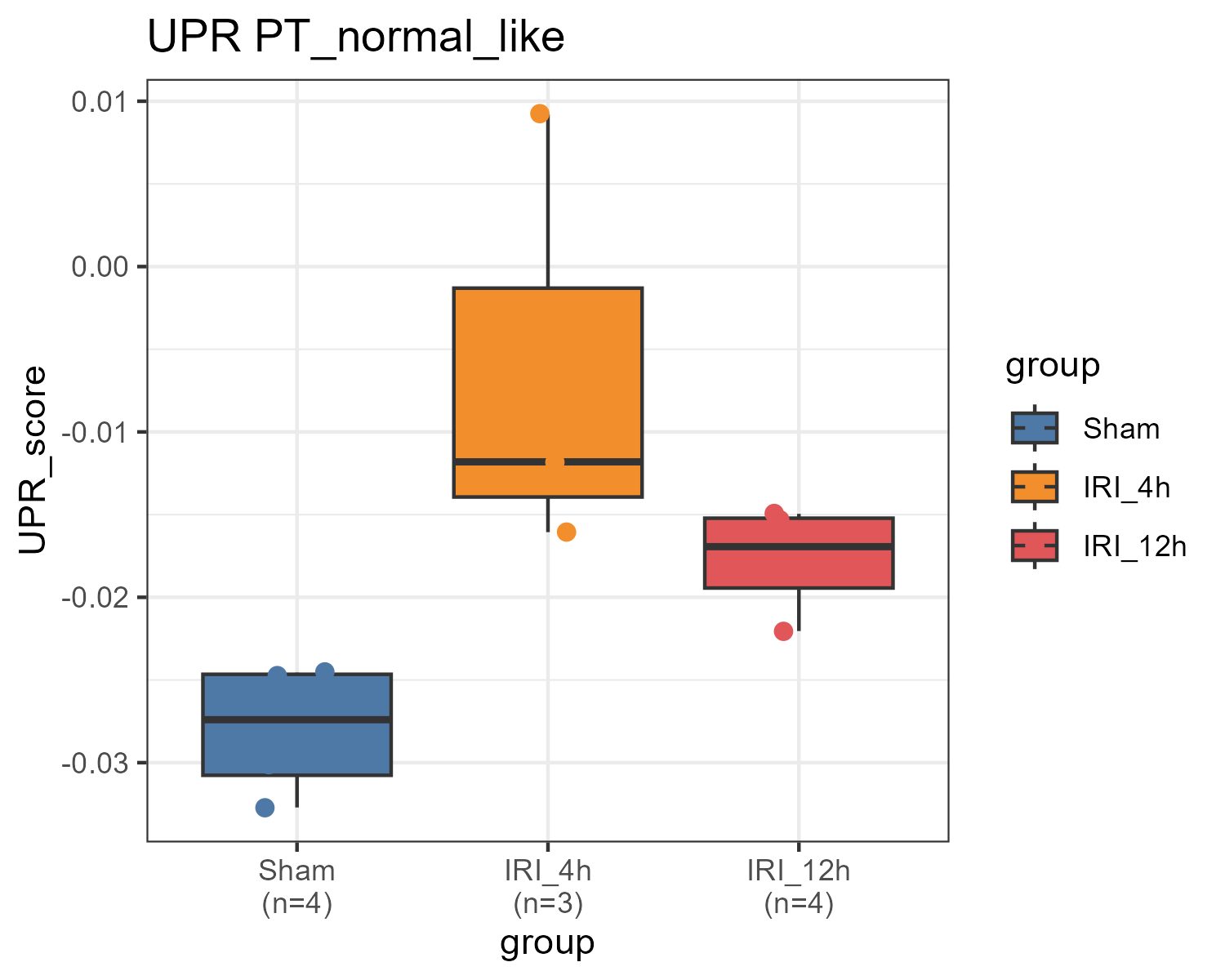


**Figure S3-066. Single-cell transcriptome analysis, step08_scores: 08 samplelevel UPR PTnormal like by group**


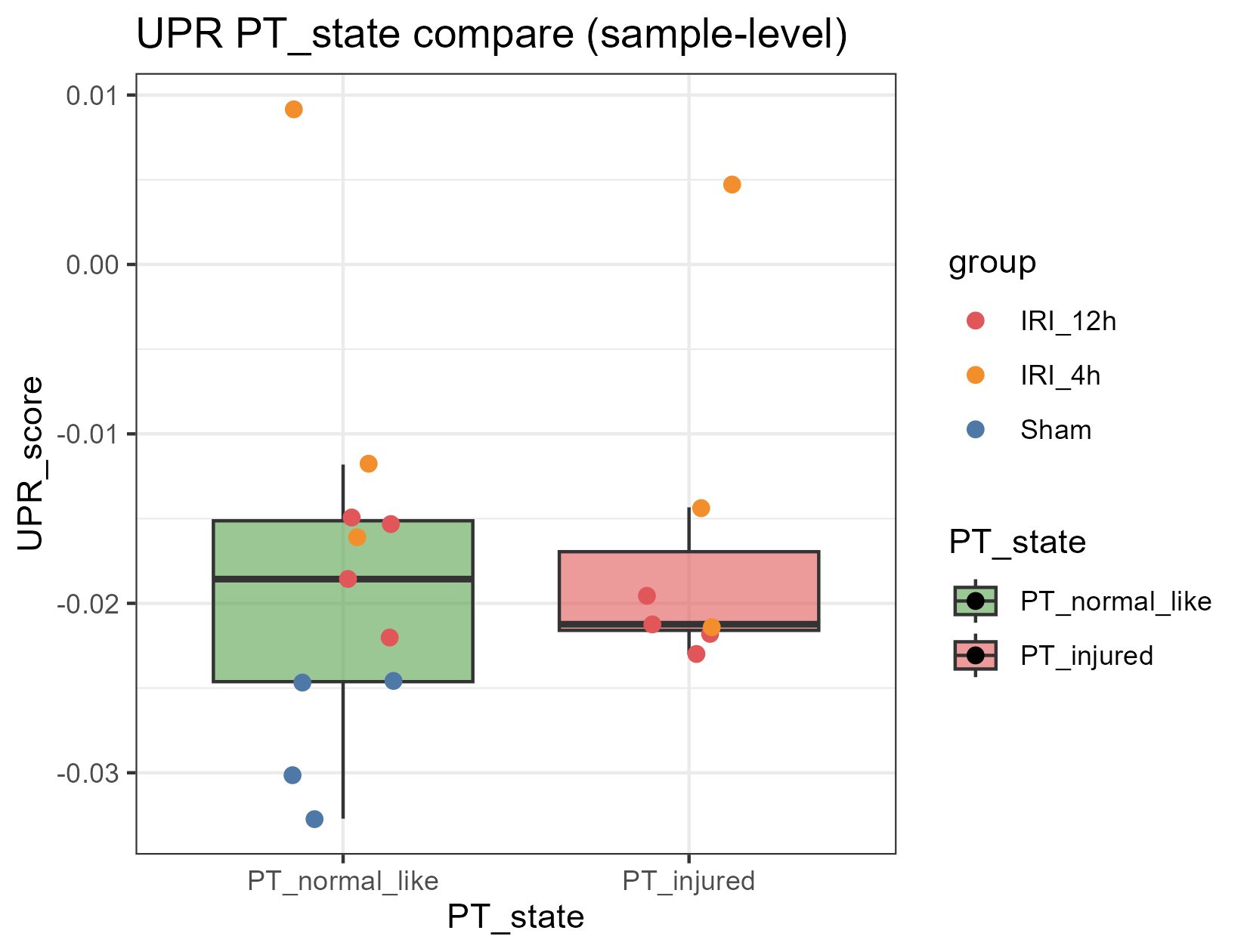


**Figure S3-067. Single-cell transcriptome analysis, step08_scores: 08 samplelevel UPR PTstate compare**


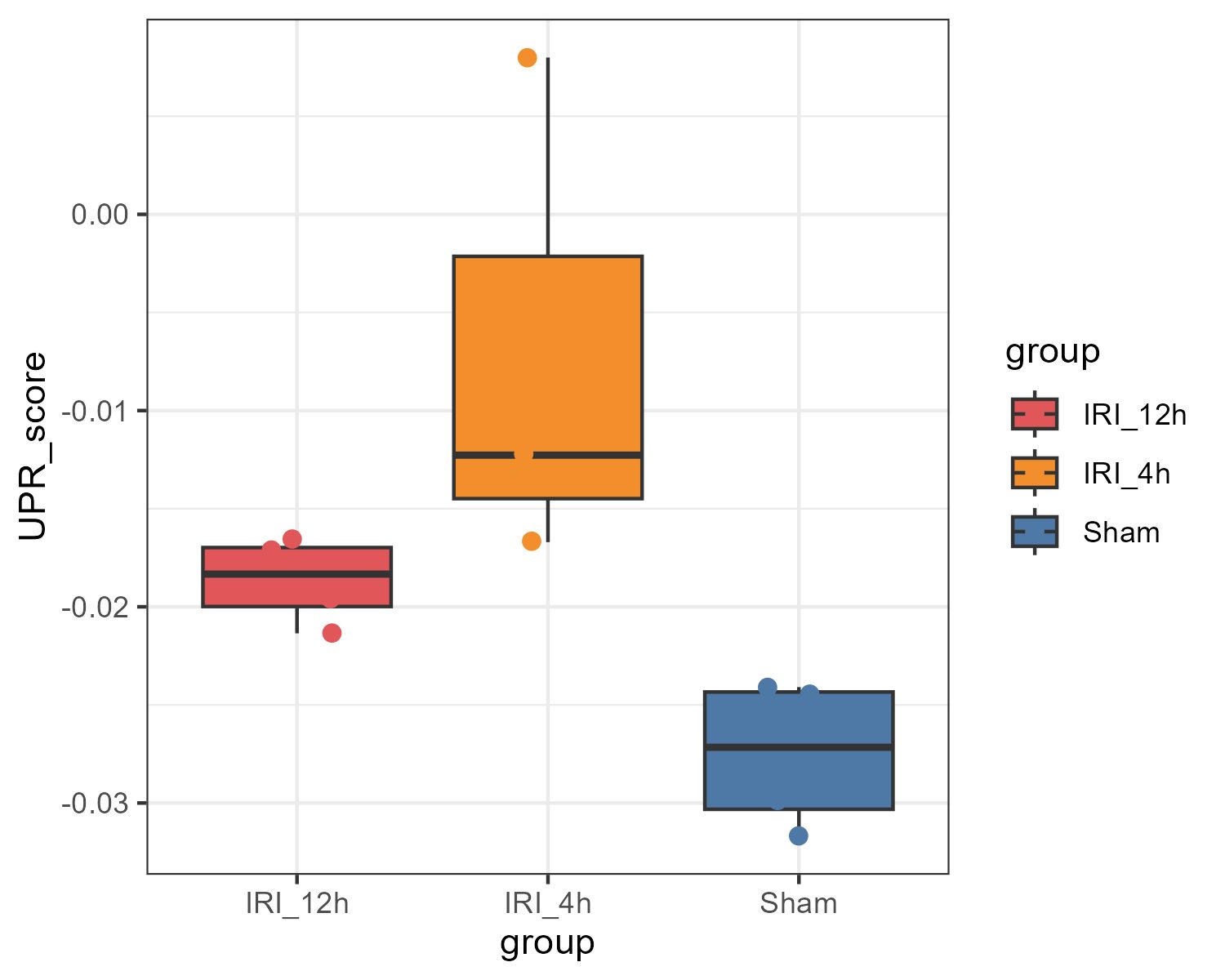


**Figure S3-068. Single-cell transcriptome analysis, step08_scores: 08 samplelevel UPR PTtotal by group**


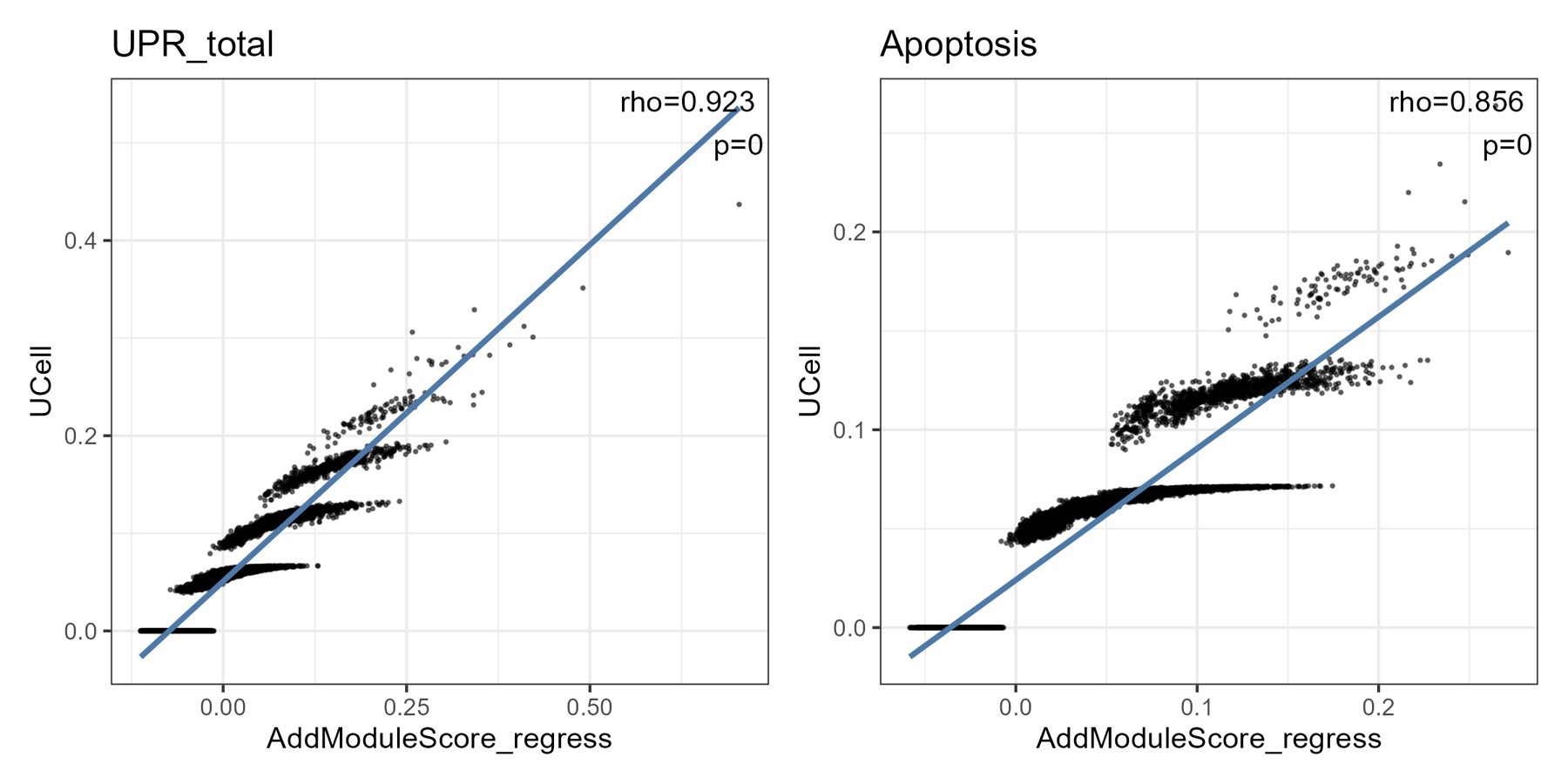


**Figure S3-069. Single-cell transcriptome analysis, step08_scores: 08 score method scatter**


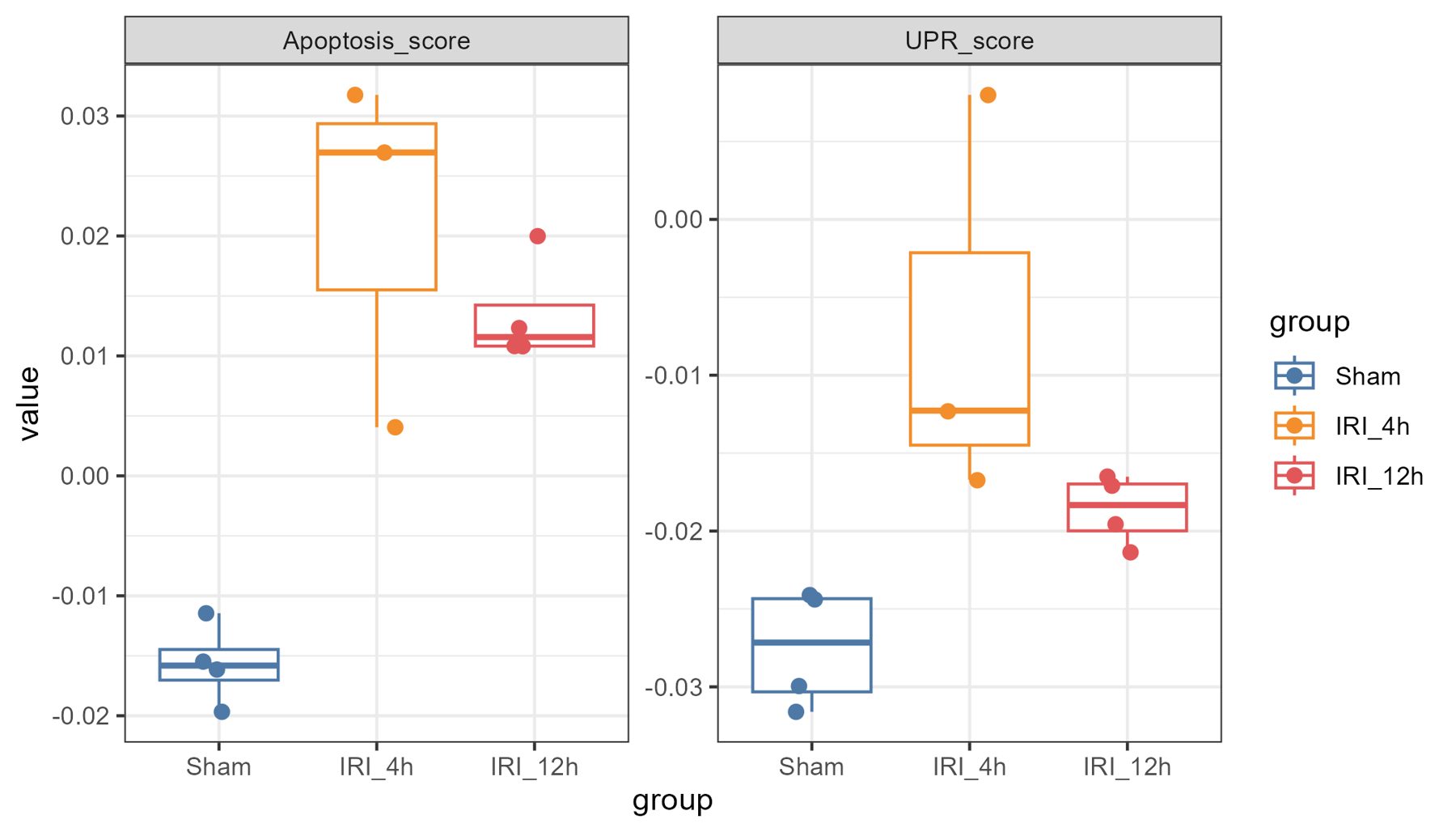


**Figure S3-070. Single-cell transcriptome analysis, step08_scores: 08 scores boxplot by group**


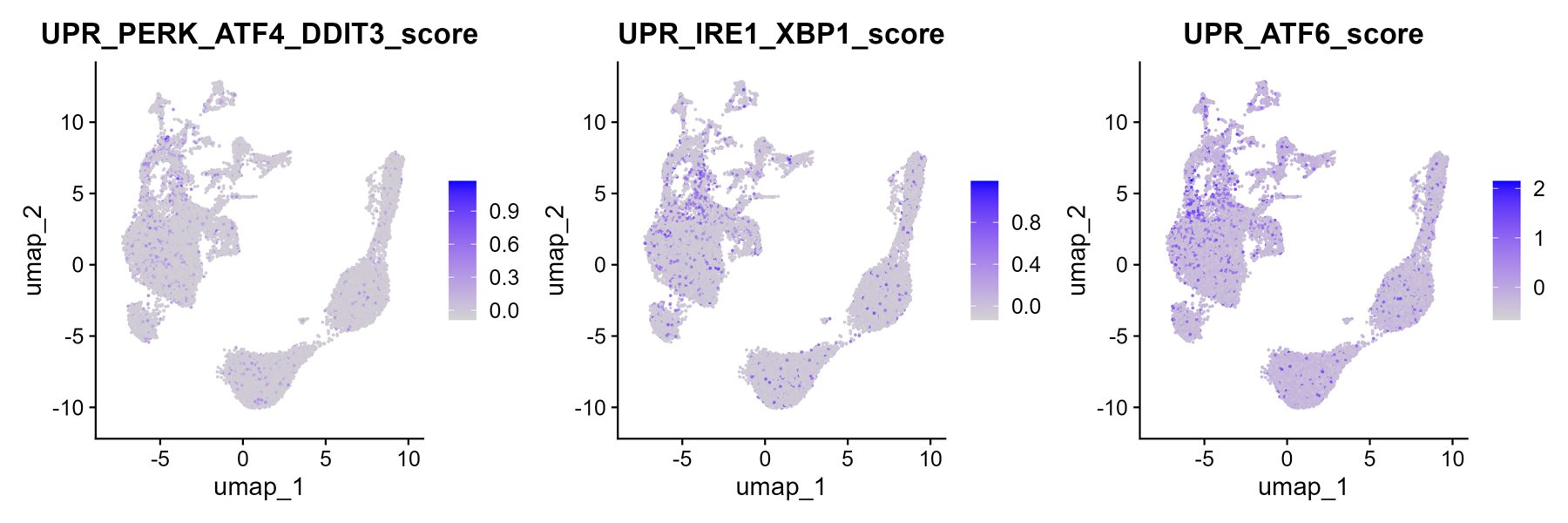


**Figure S3-071. Single-cell transcriptome analysis, step08_scores: 08 UPR branch featureplot on PT umap**


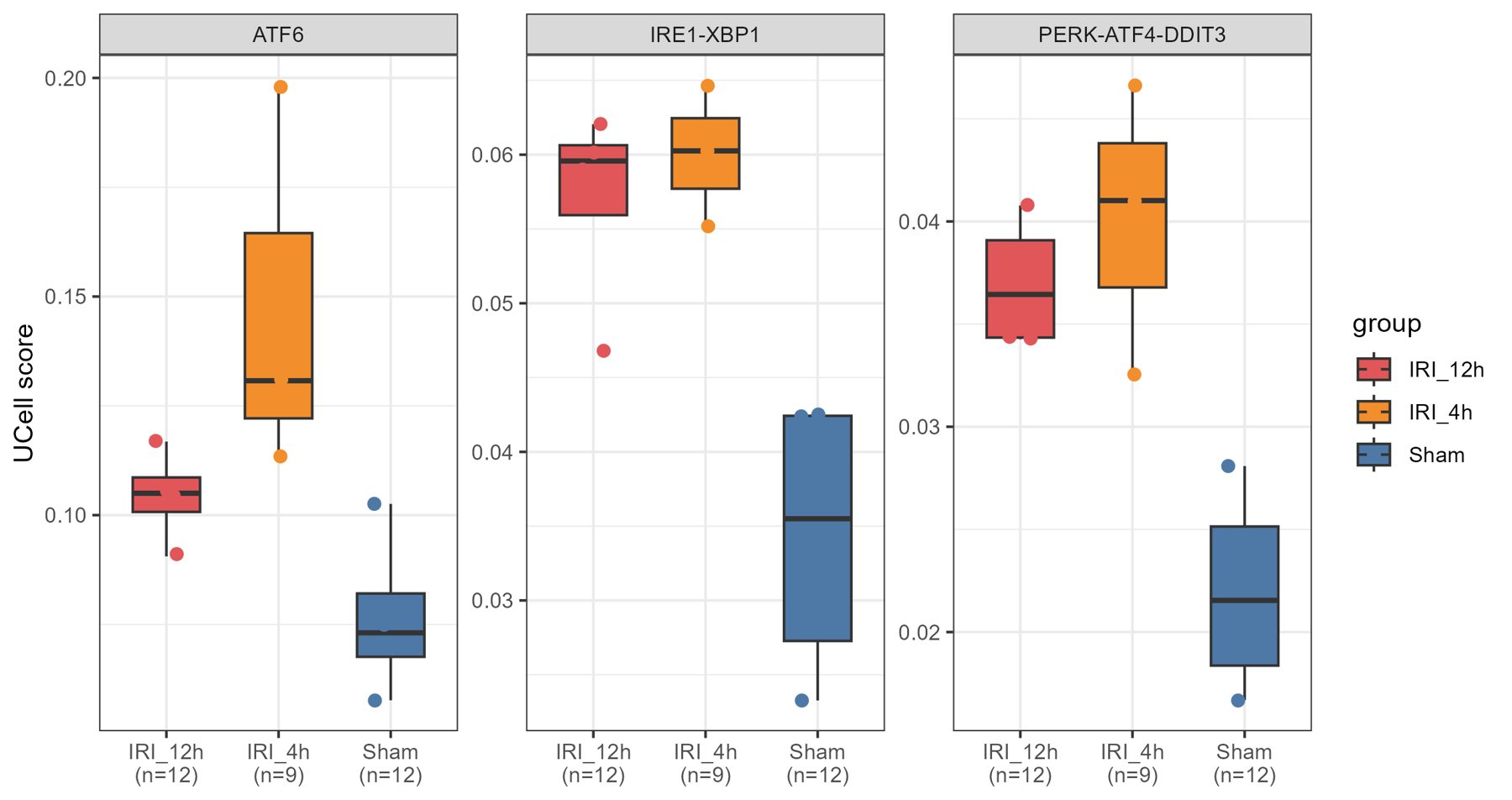


**Figure S3-072. Single-cell transcriptome analysis, step08_scores: 08 UPR branch samplelevel by group**


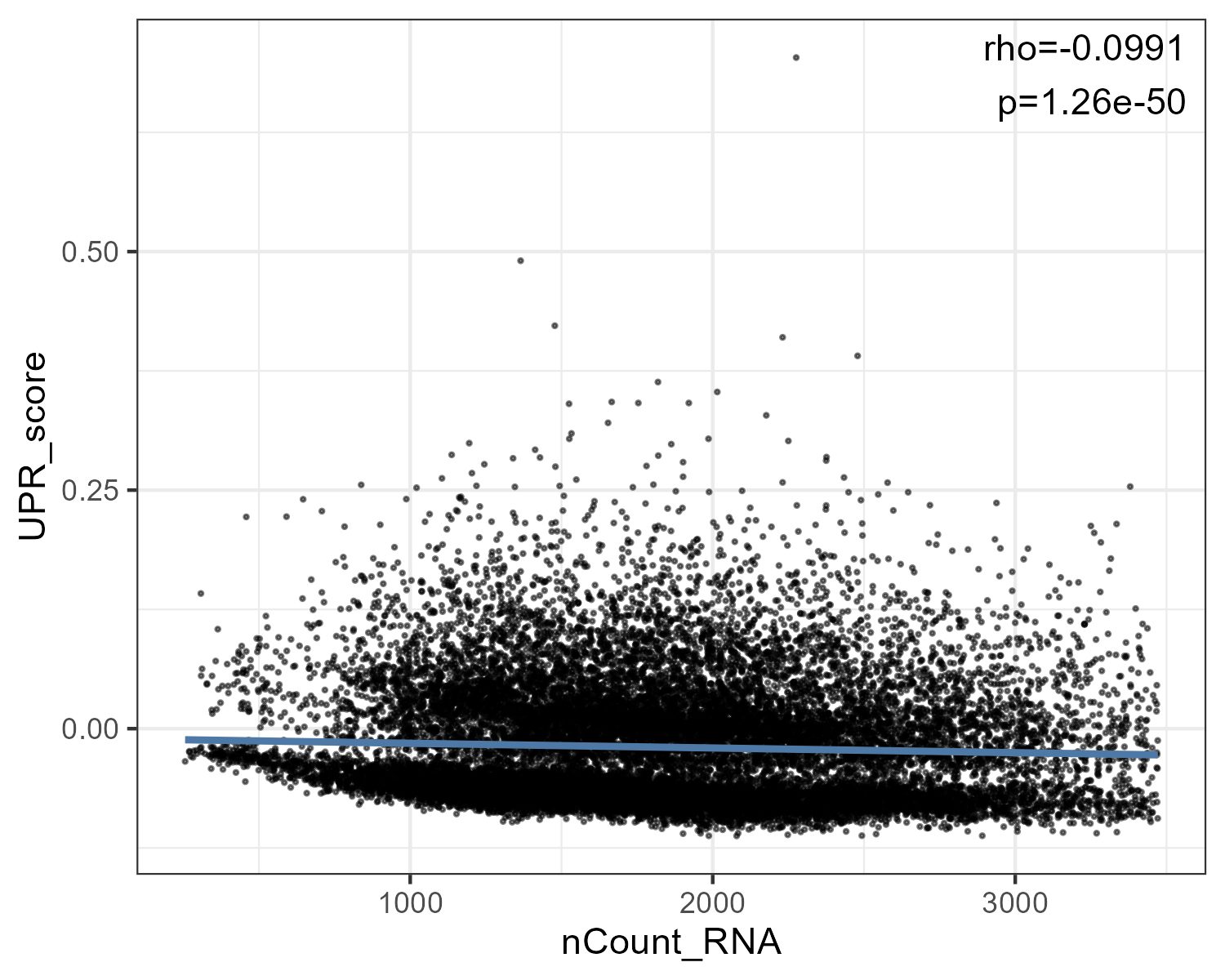


**Figure S3-073. Single-cell transcriptome analysis, step08_scores: 08 UPR score vs nCount**


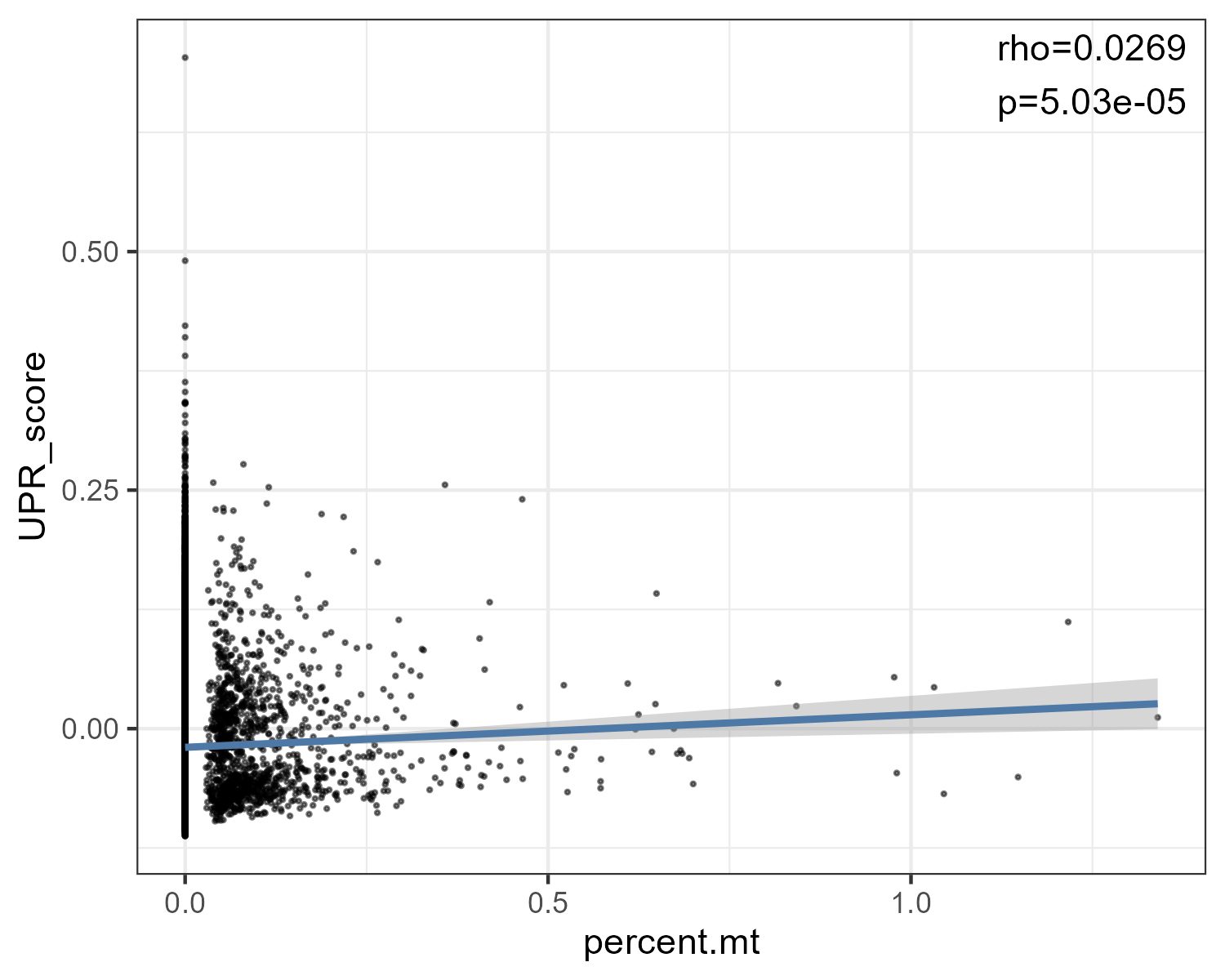


**Figure S3-074. Single-cell transcriptome analysis, step08_scores: 08 UPR score vs percentmt**

# Section: step08_scores/QC


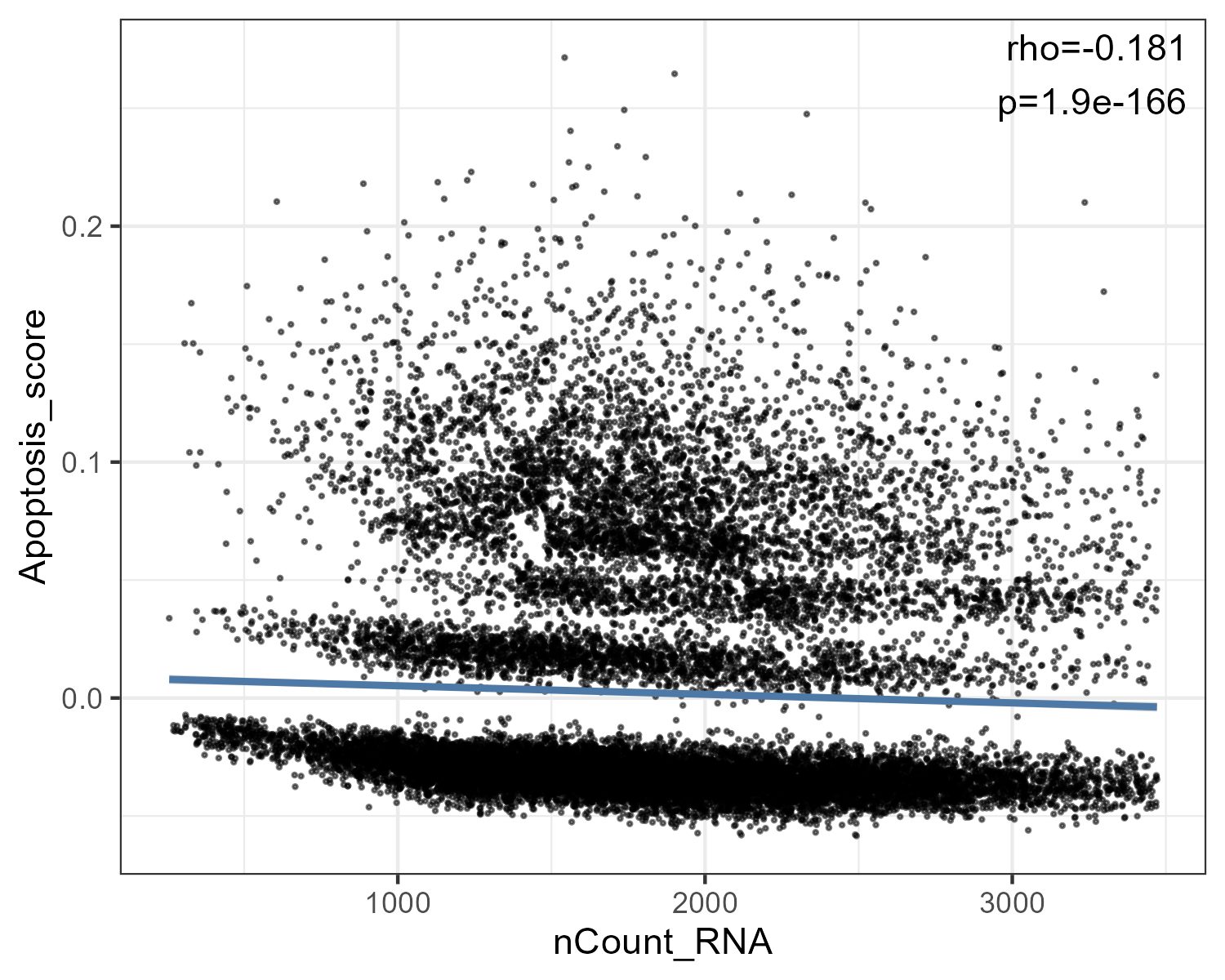


**Figure S3-075. Single-cell transcriptome analysis, step08_scores: 08 Apop score vs nCount**


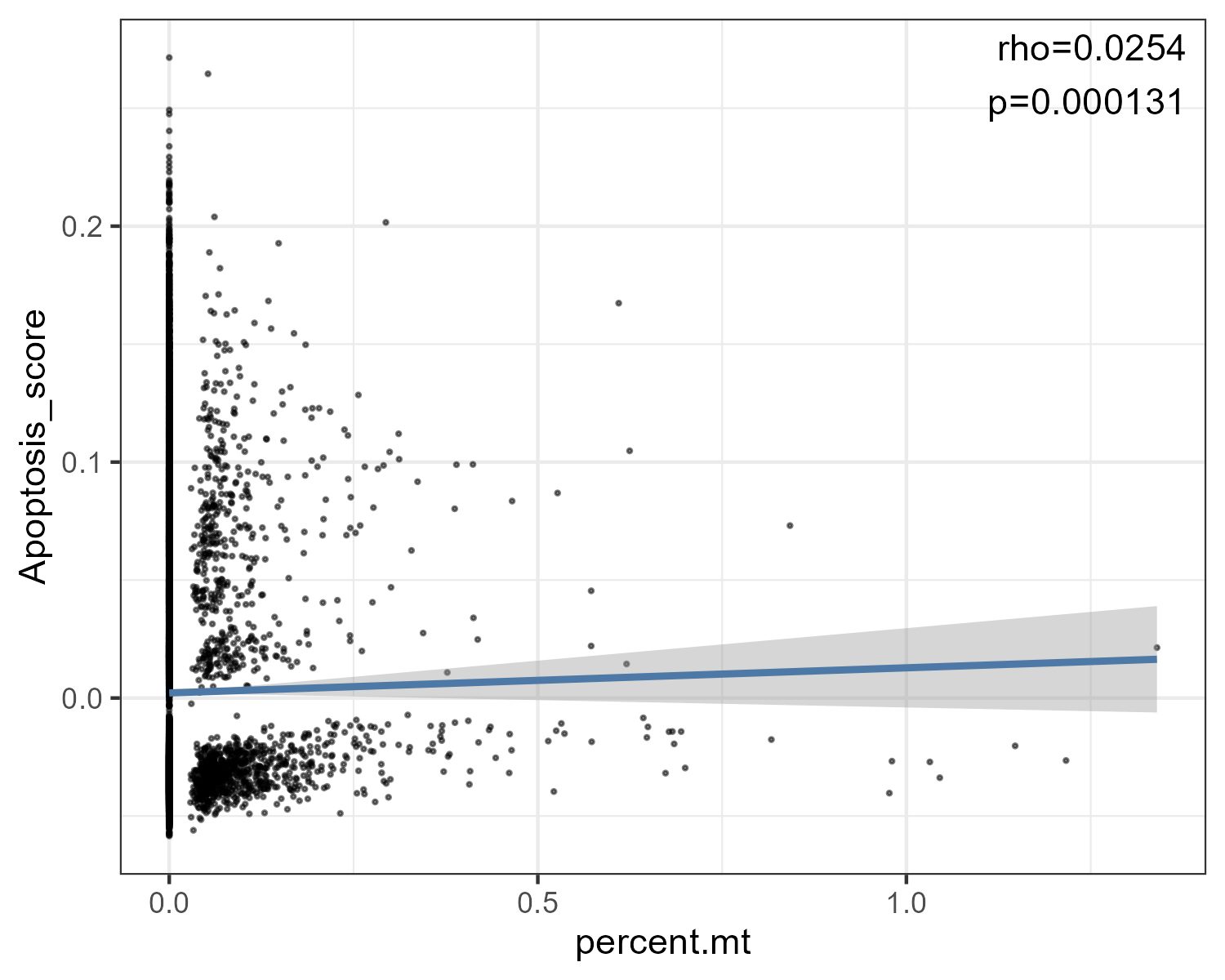


**Figure S3-076. Single-cell transcriptome analysis, step08_scores: 08 Apop score vs percentmt**


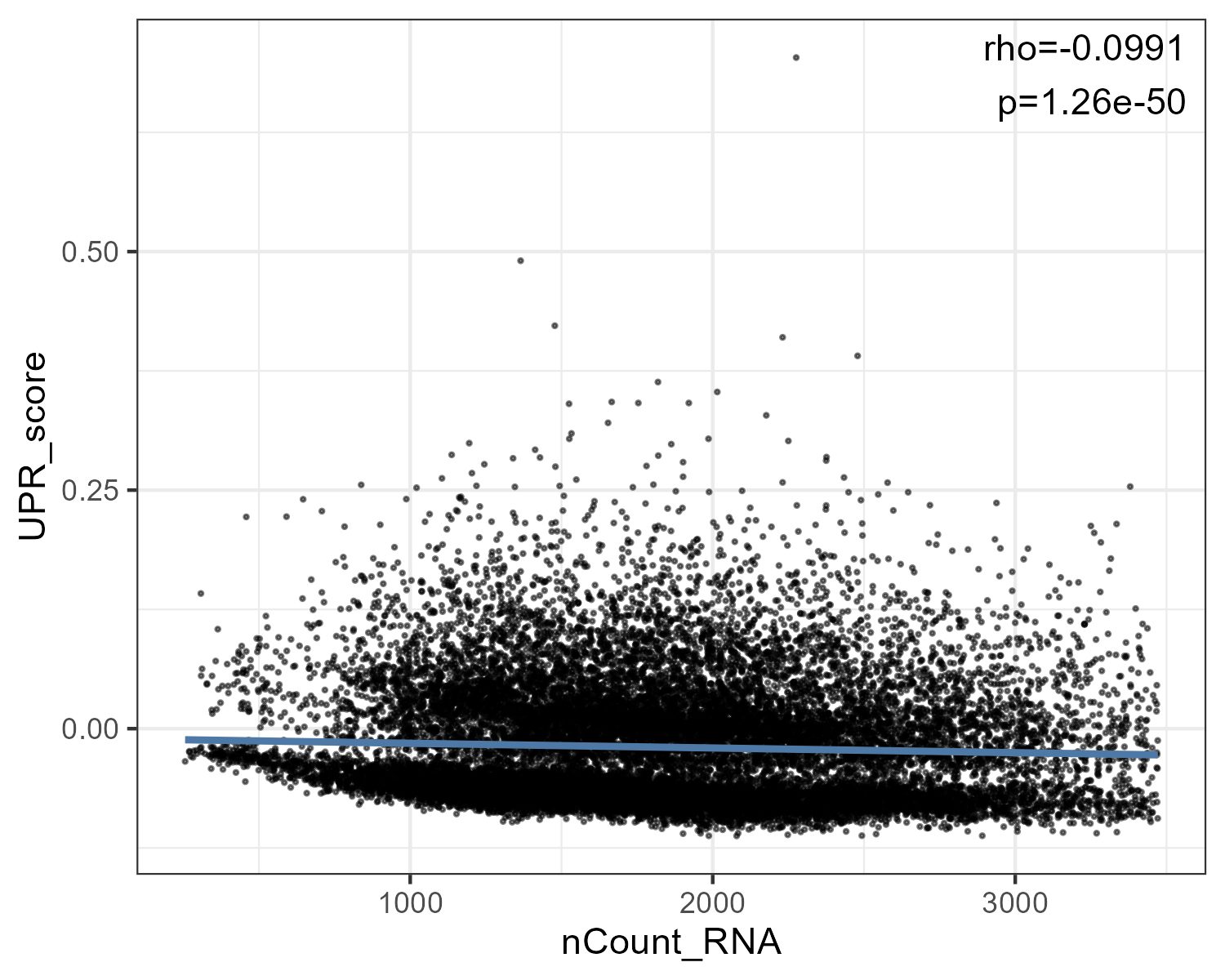


**Figure S3-077. Single-cell transcriptome analysis, step08_scores: 08 UPR score vs nCount**


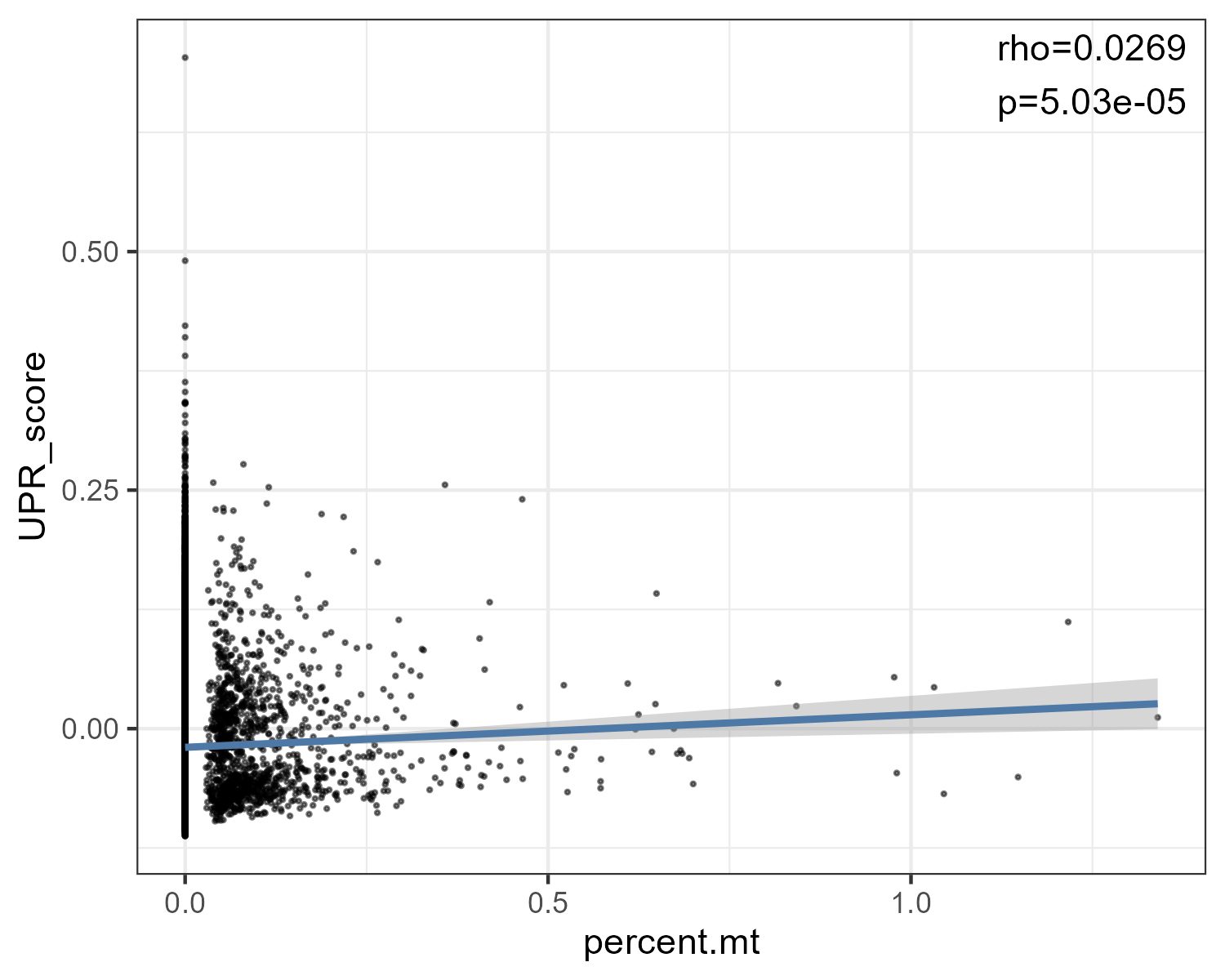


**Figure S3-078. Single-cell transcriptome analysis, step08_scores: 08 UPR score vs percentmt**

# Section: step09_pseudobulk


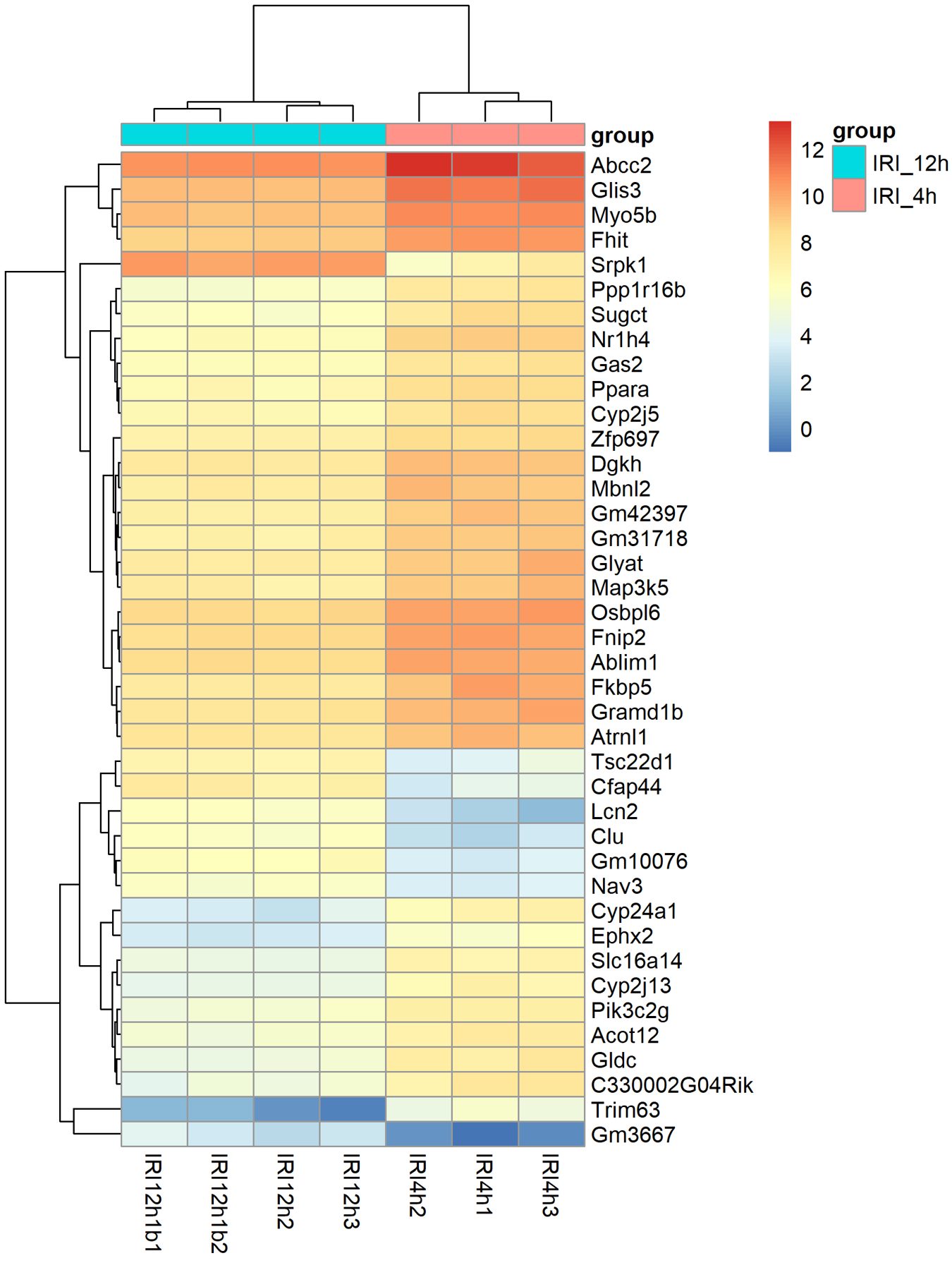


**Figure S3-079. Single-cell transcriptome analysis, step09_pseudobulk: 09 DE PT IRI 4h vs IRI 12h heatmap topgenes**


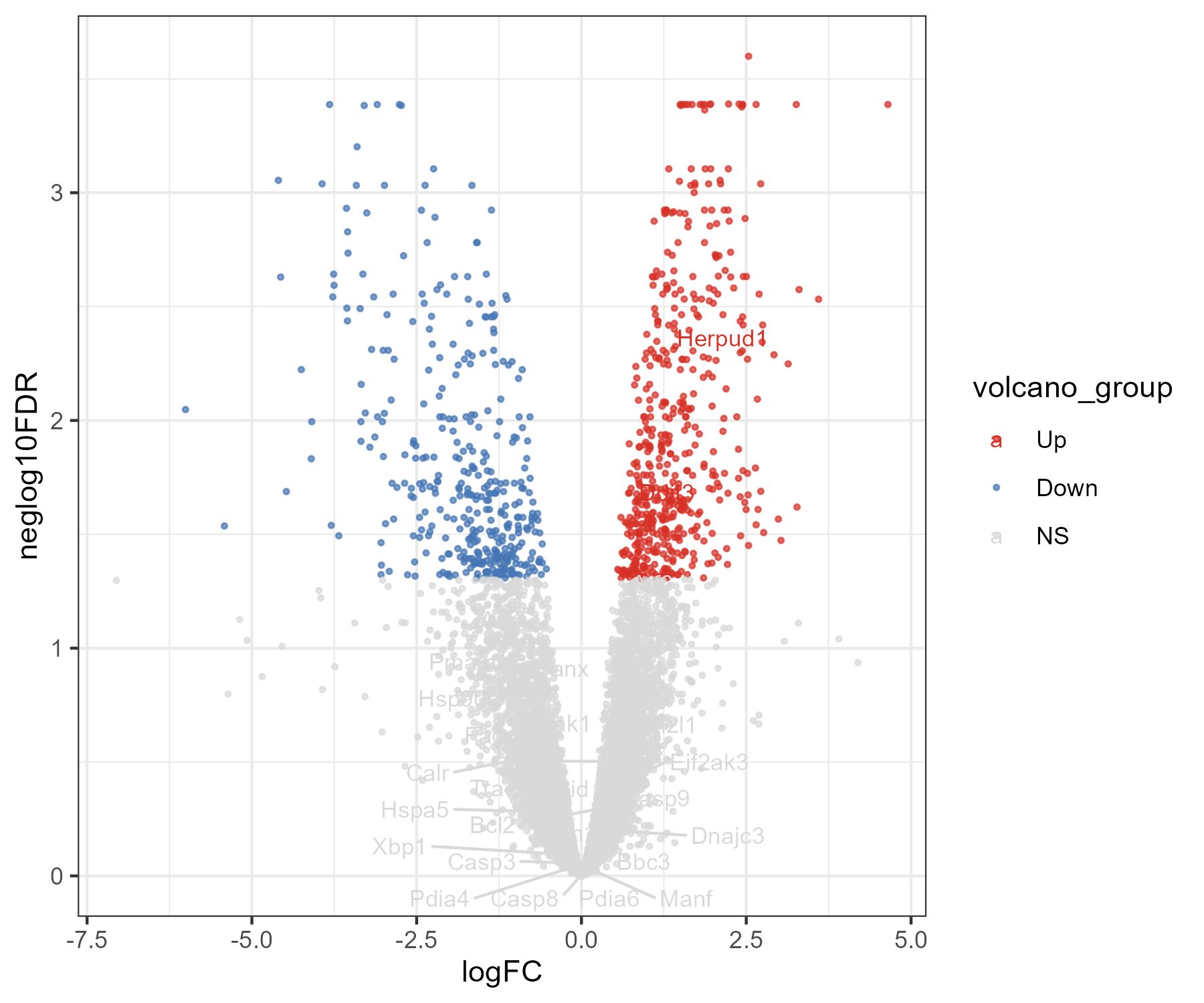


**Figure S3-080. Single-cell transcriptome analysis, step09_pseudobulk: 09 DE PT IRI 4h vs IRI 12h volcano**


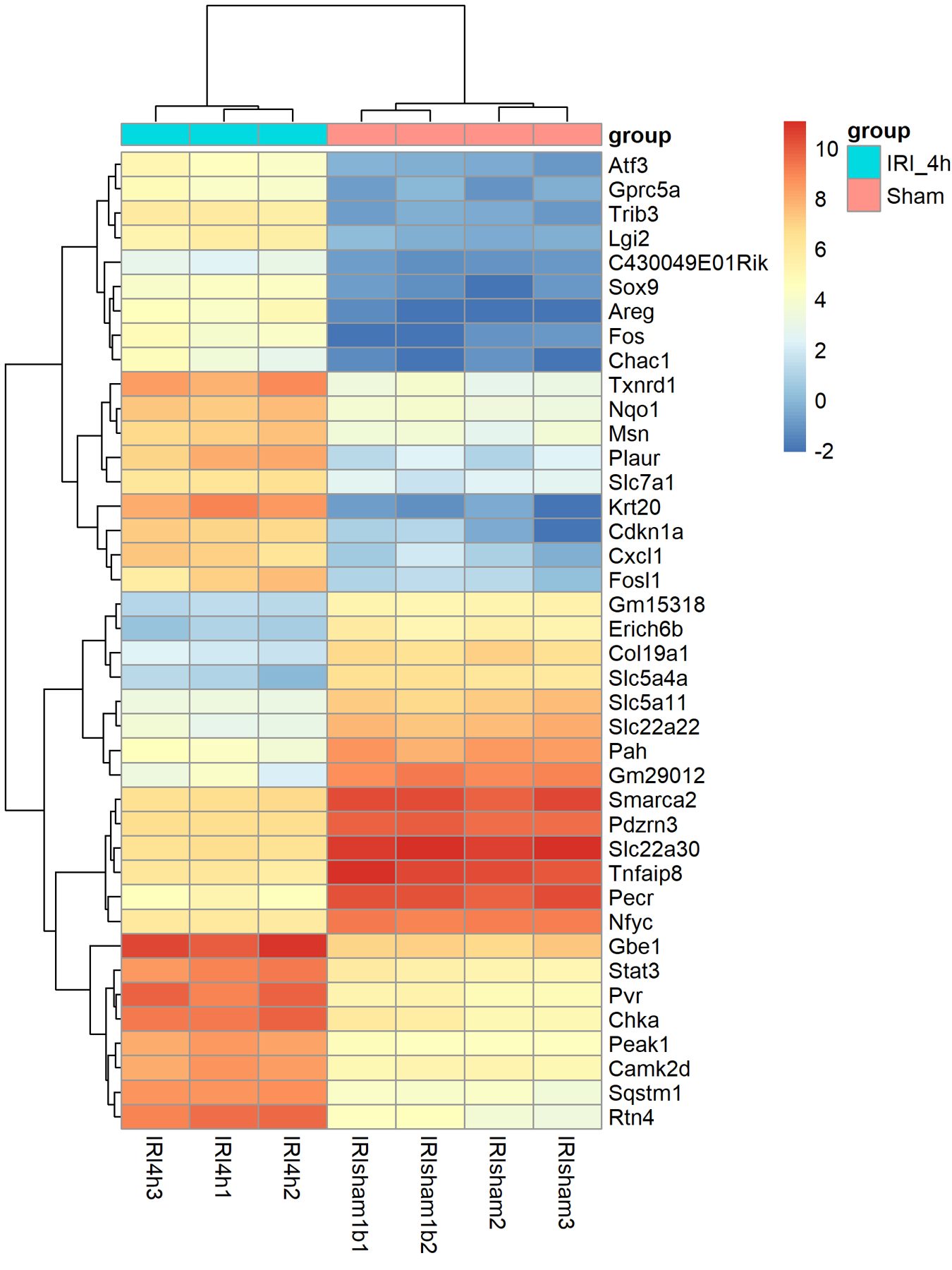


**Figure S3-081. Single-cell transcriptome analysis, step09_pseudobulk: 09 DE PT Sham vs IRI 4h heatmap topgenes**


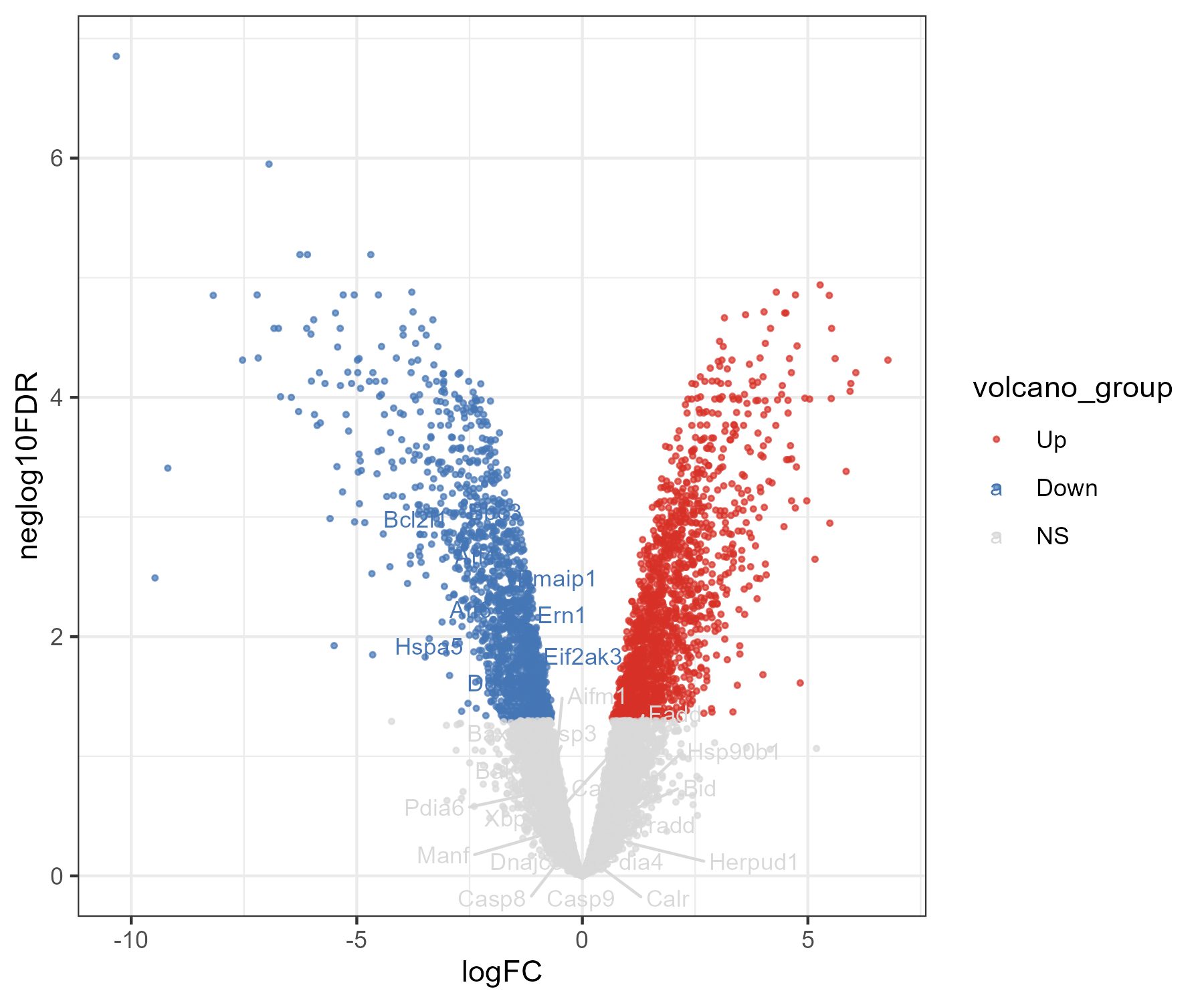


**Figure S3-082. Single-cell transcriptome analysis, step09_pseudobulk: 09 DE PT Sham vs IRI 4h volcano**


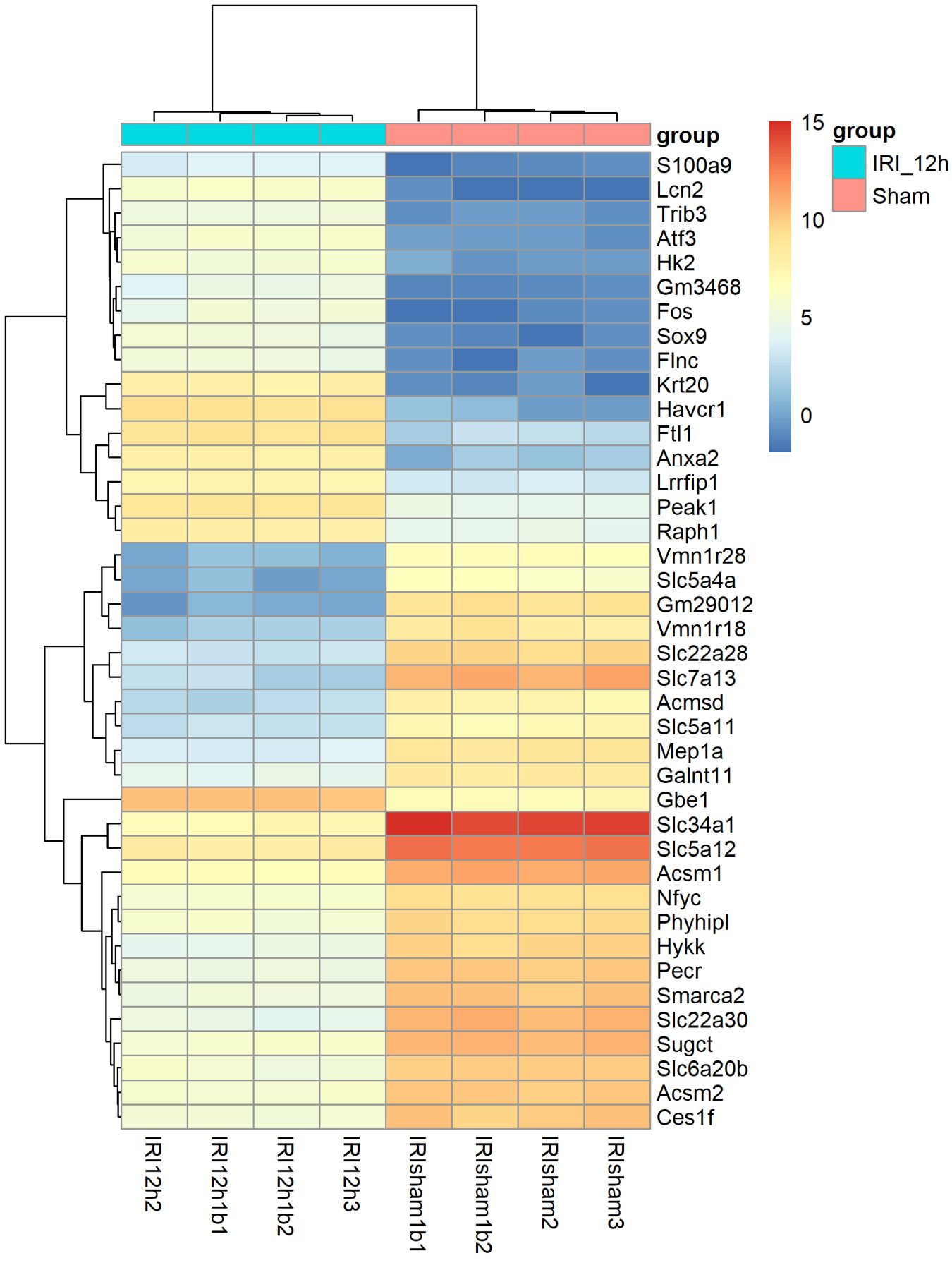


**Figure S3-083. Single-cell transcriptome analysis, step09_pseudobulk: 09 DE PT Sham vs IRI 12h heatmap topgenes**


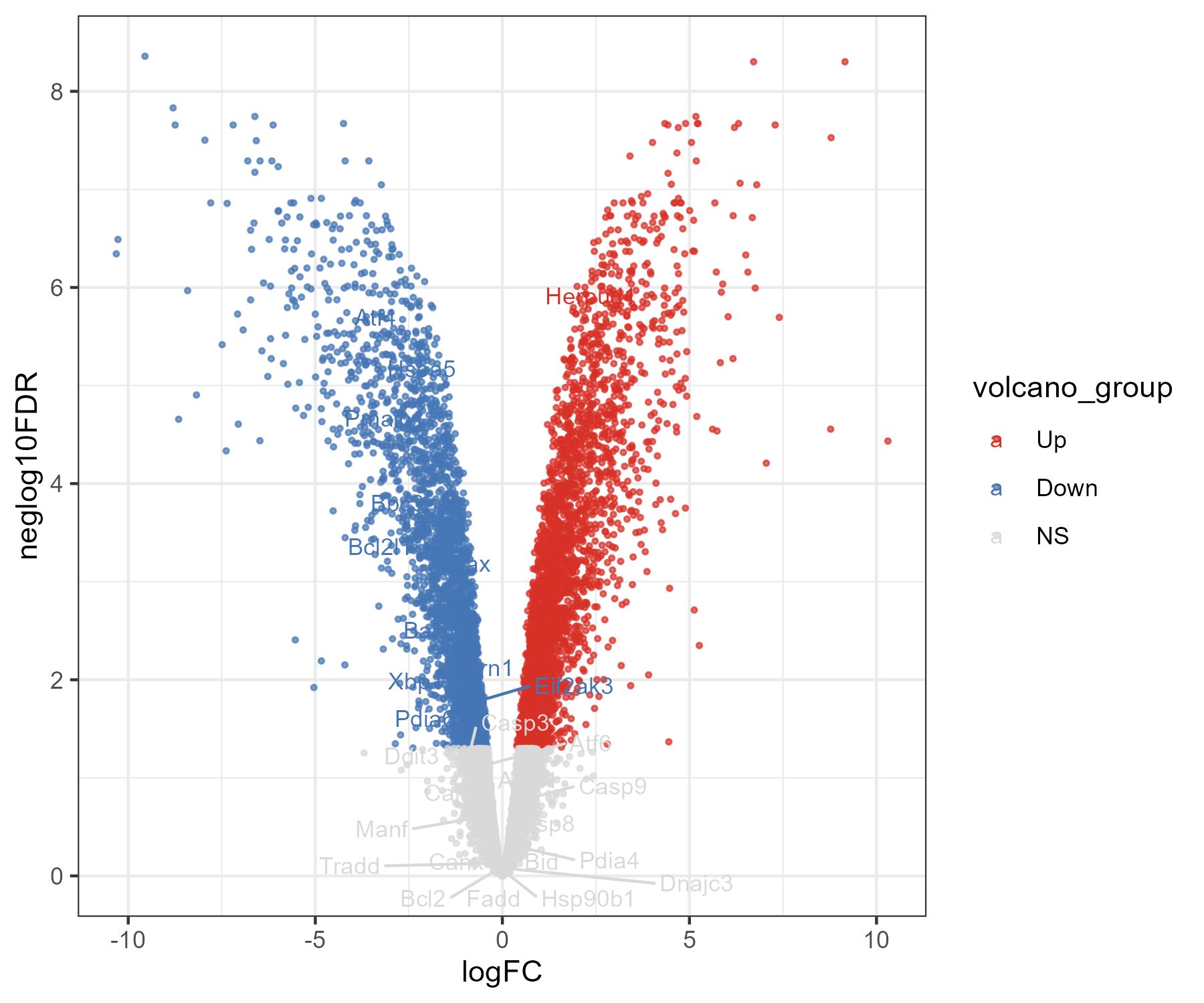


**Figure S3-084. Single-cell transcriptome analysis, step09_pseudobulk: 09 DE PT Sham vs IRI 12h volcano**


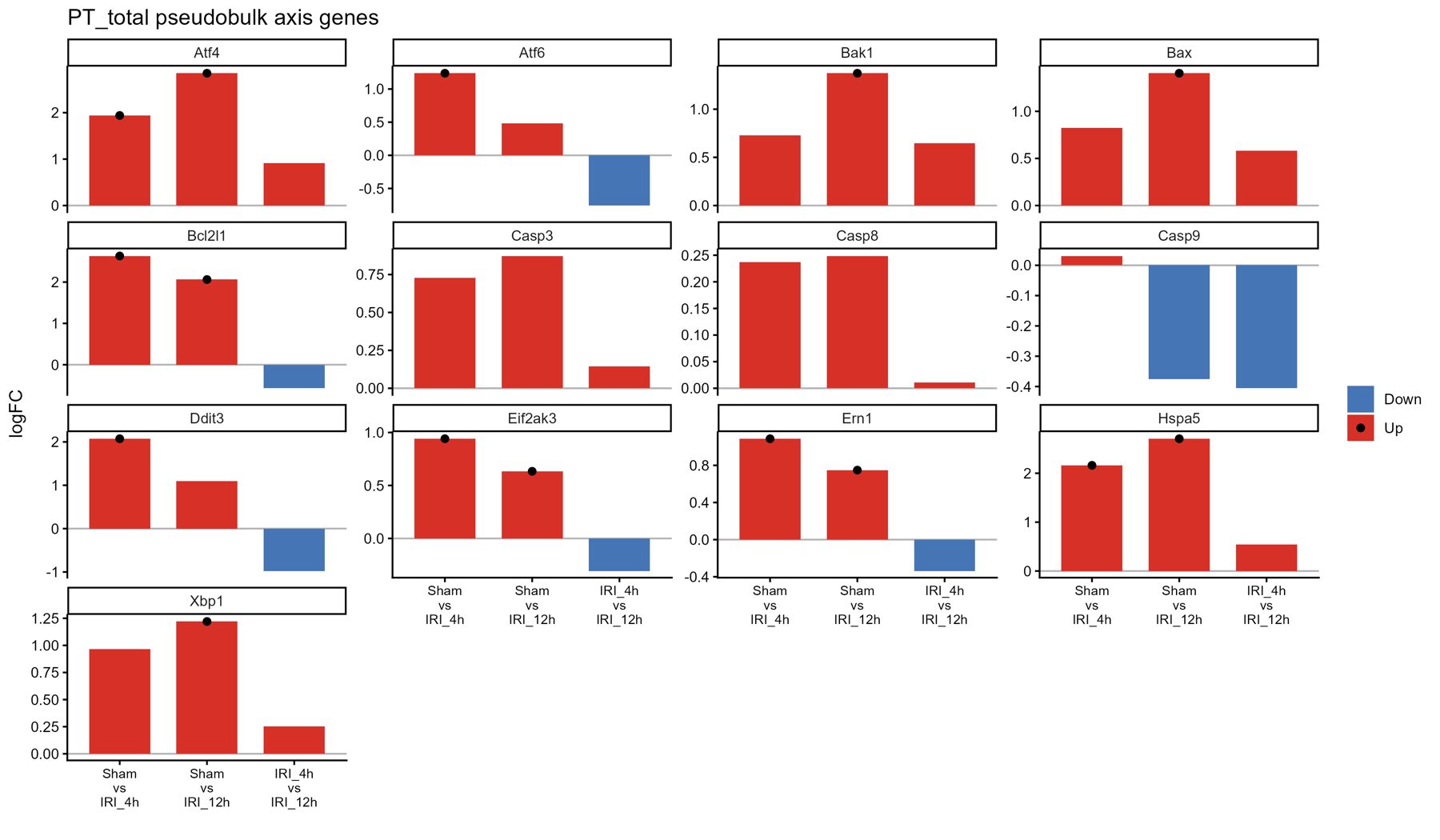


**Figure S3-085. Single-cell transcriptome analysis, step09_pseudobulk: 09 PTtotal axis genes logFC barplot**


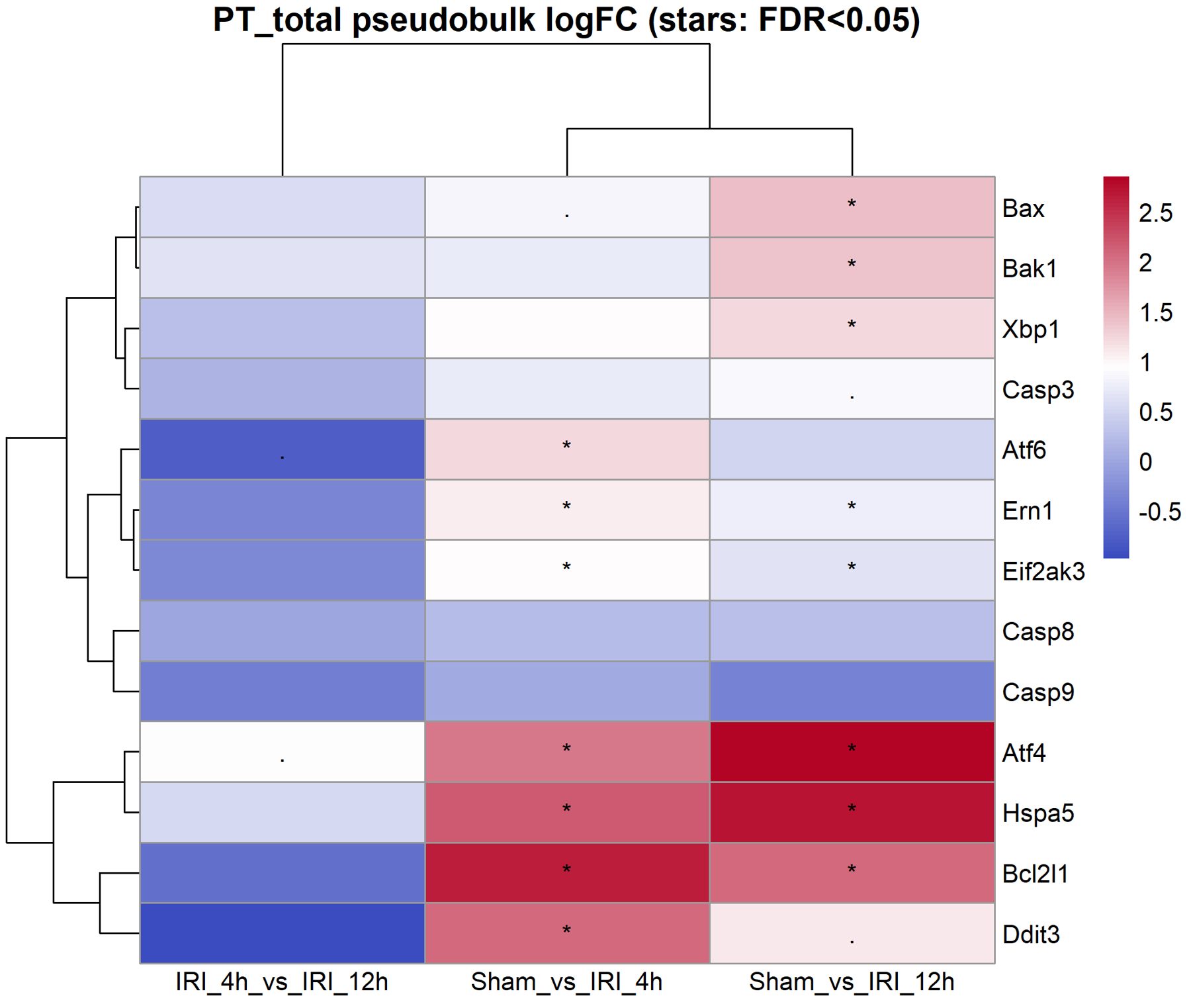


**Figure S3-086. Single-cell transcriptome analysis, step09_pseudobulk: 09 PTtotal axis genes logFC heatmap**

# Section: step10_enrichment


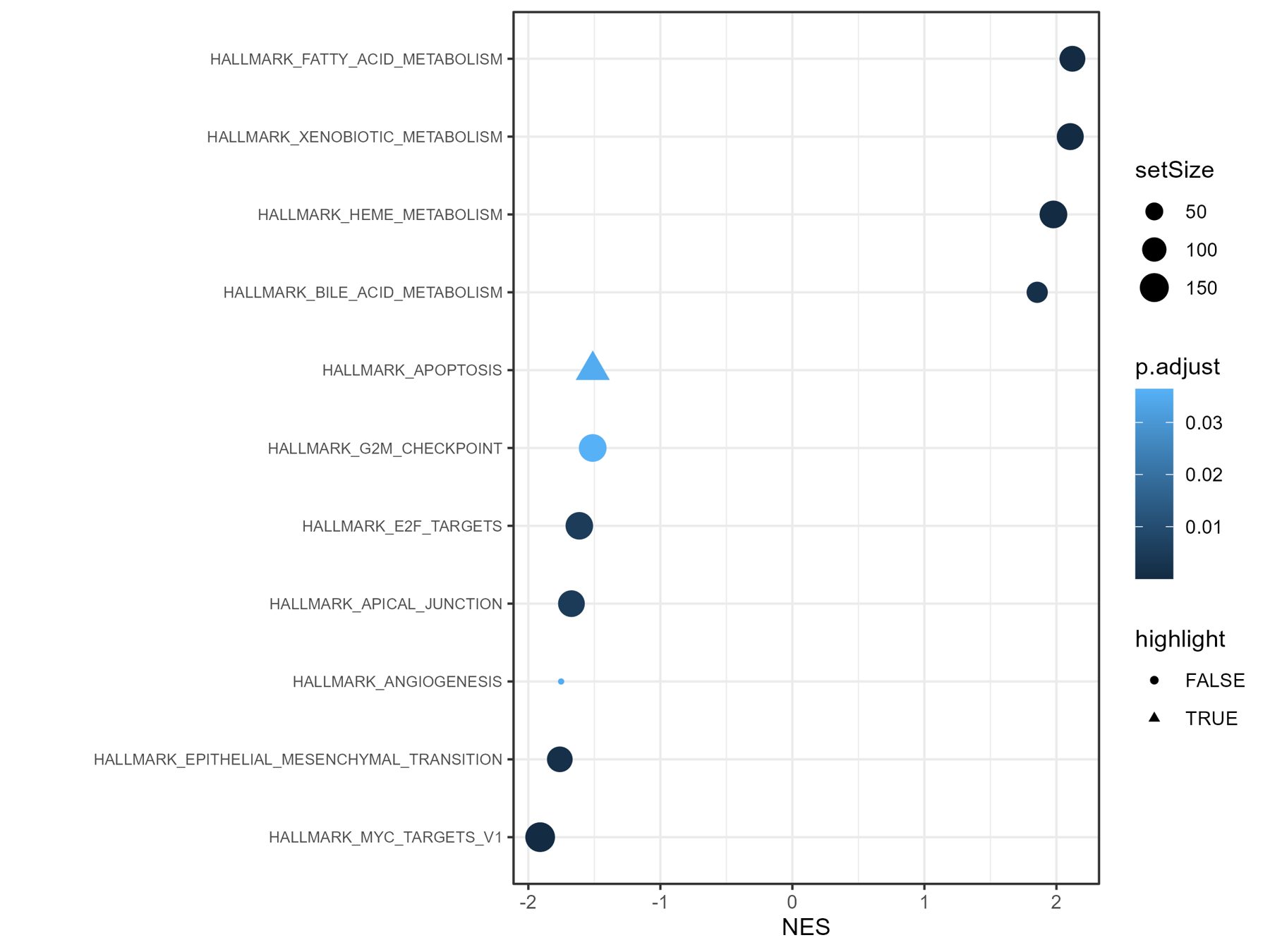


**Figure S3-087. Single-cell transcriptome analysis, step10_enrichment: 10 GSEA HALLMARK top20 dotplot PT IRI 4h vs IRI 12h**


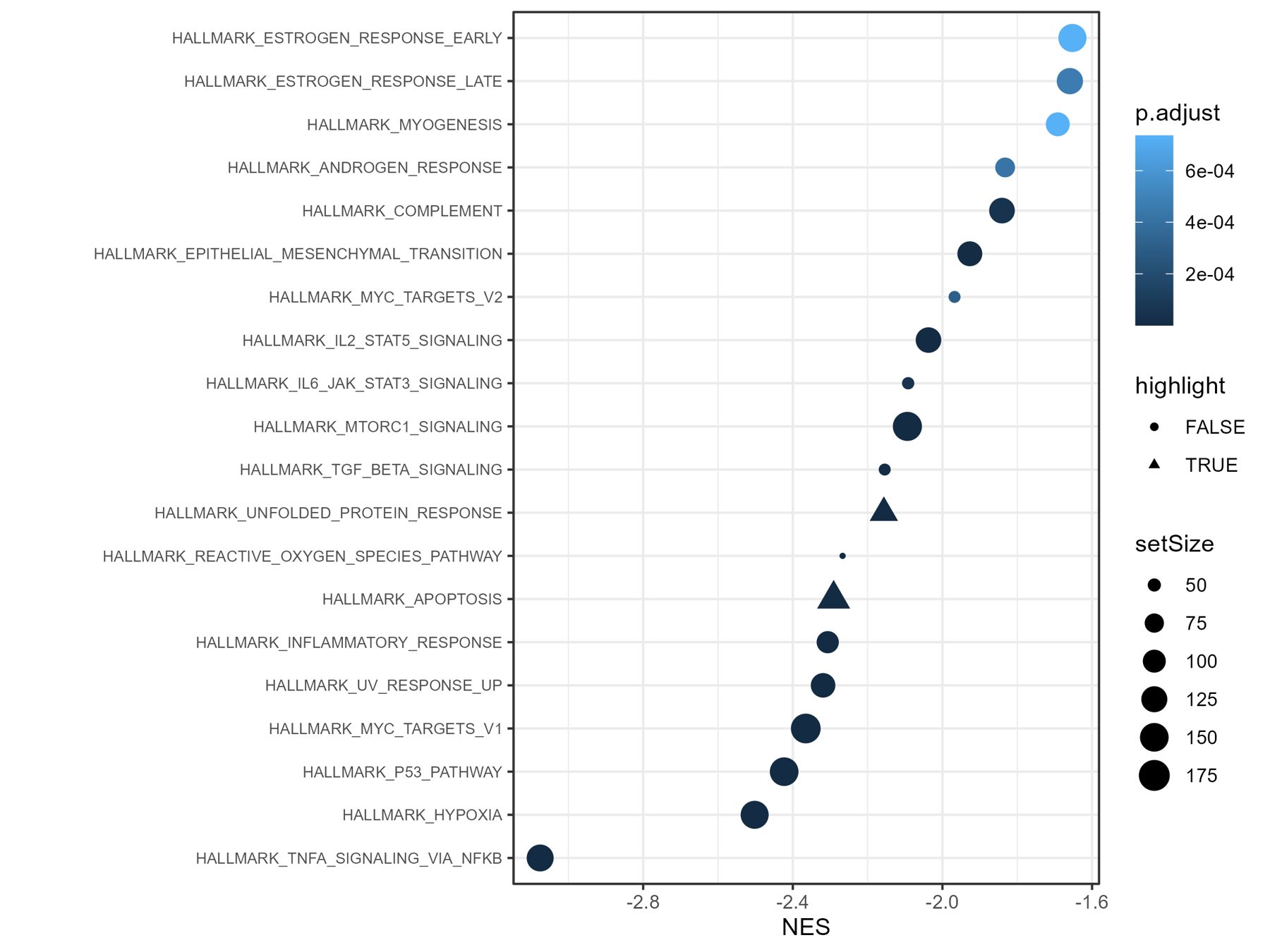


**Figure S3-088. Single-cell transcriptome analysis, step10_enrichment: 10 GSEA HALLMARK top20 dotplot PT Sham vs IRI 4h**


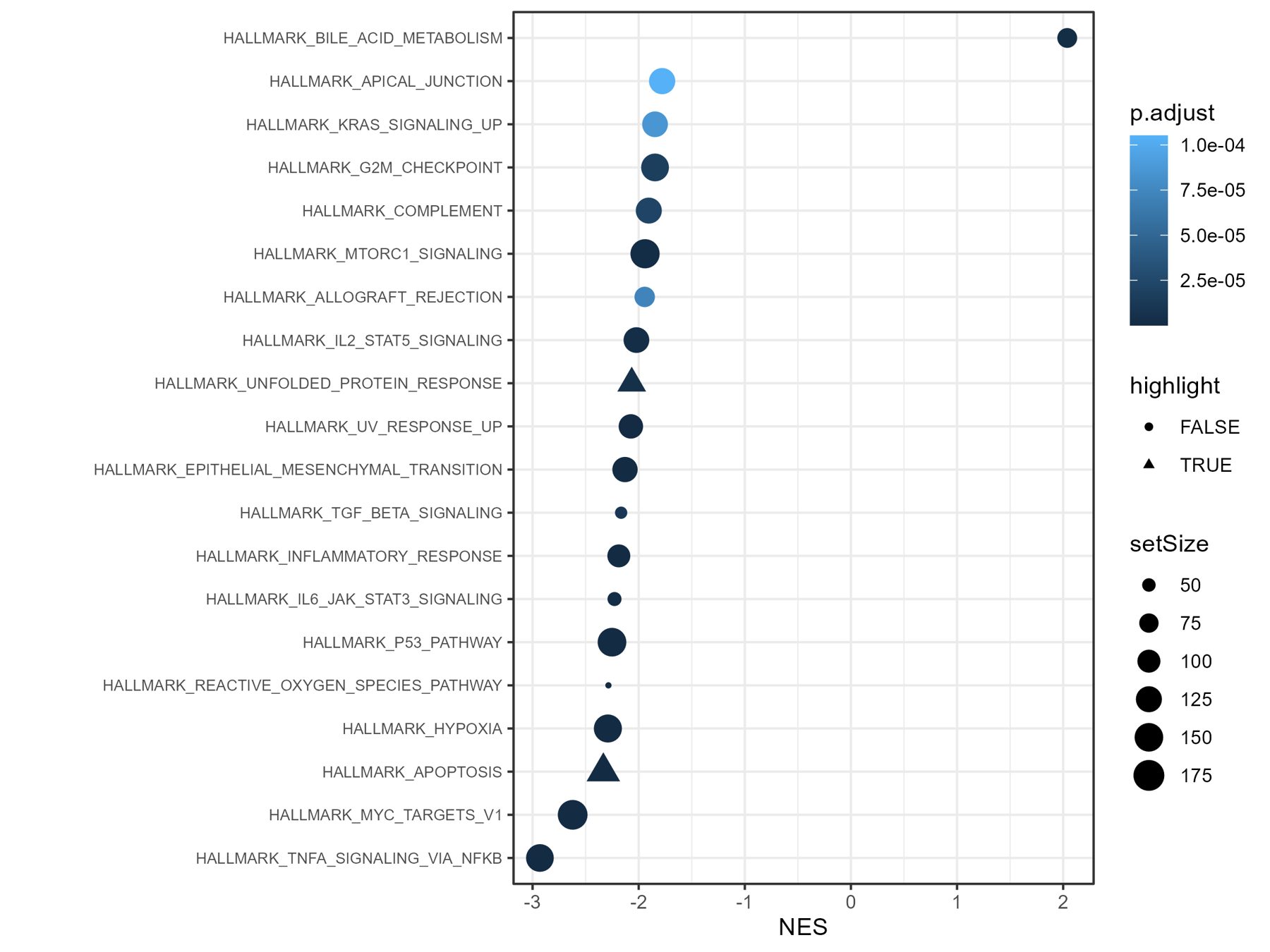


**Figure S3-089. Single-cell transcriptome analysis, step10_enrichment: 10 GSEA HALLMARK top20 dotplot PT Sham vs IRI 12h**


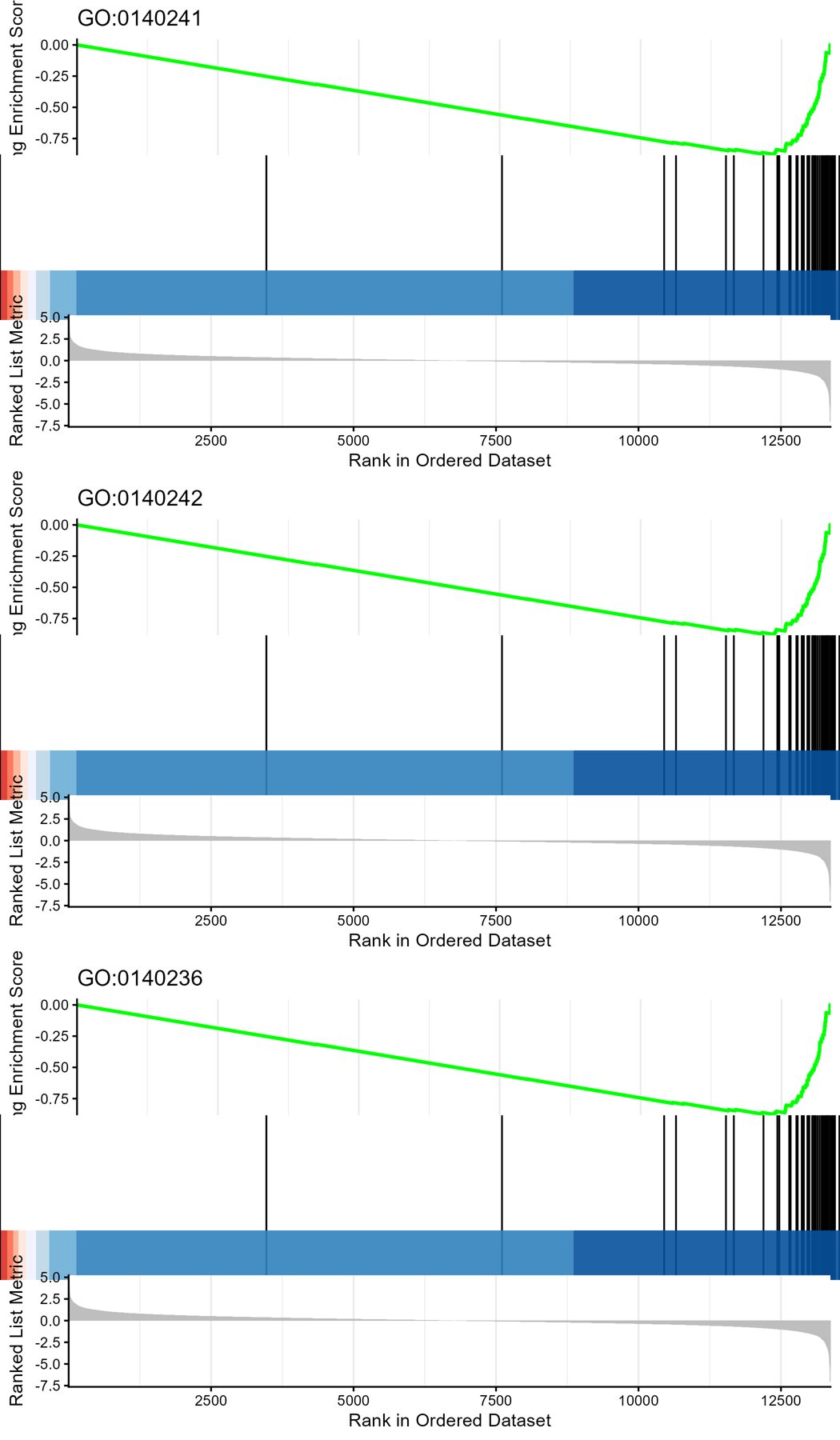


**Figure S3-090. Single-cell transcriptome analysis, step10_enrichment: 10 GSEA top pathway curves PT IRI 4h vs IRI 12h**


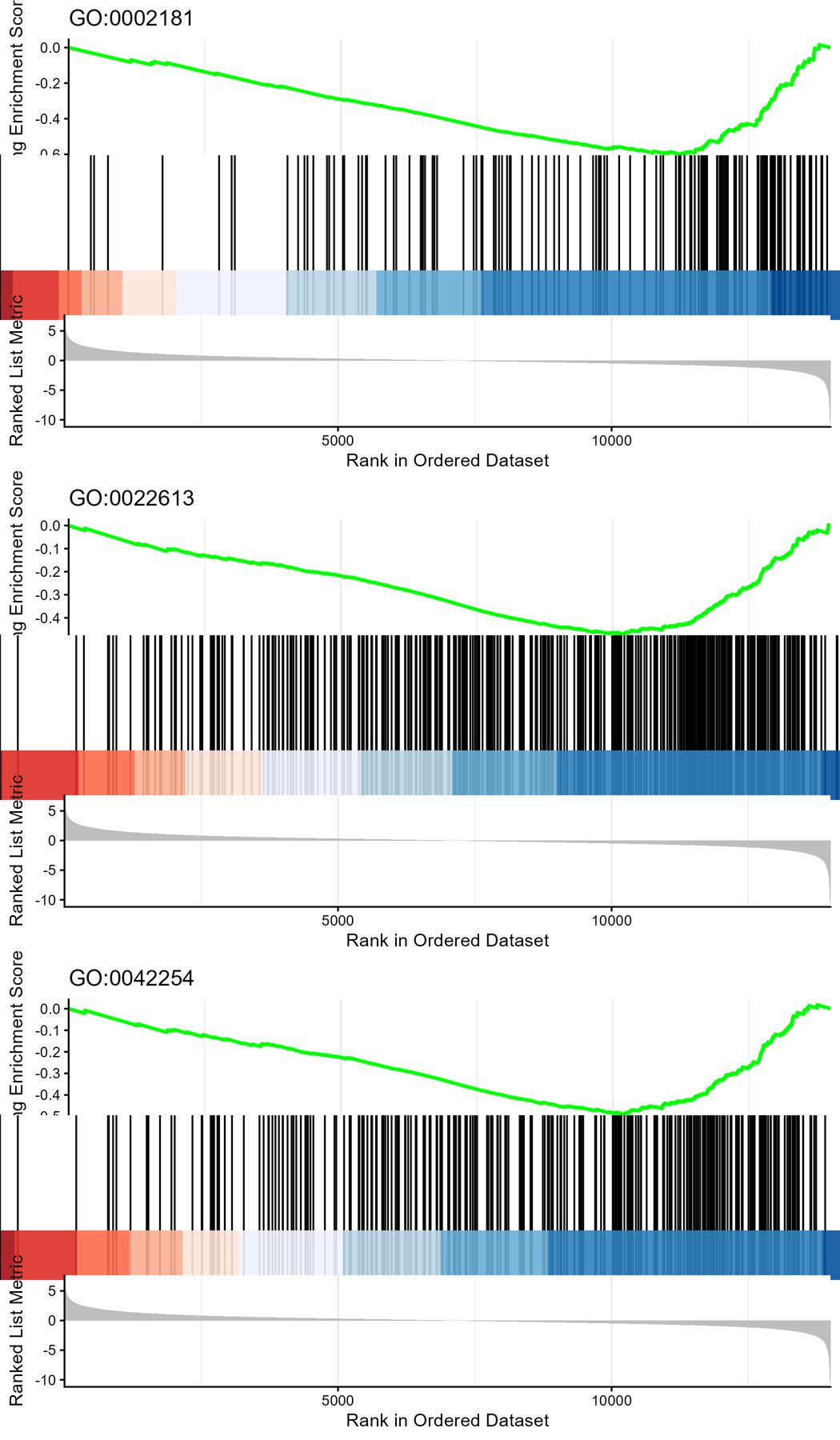


**Figure S3-091. Single-cell transcriptome analysis, step10_enrichment: 10 GSEA top pathway curves PT Sham vs IRI 4h**


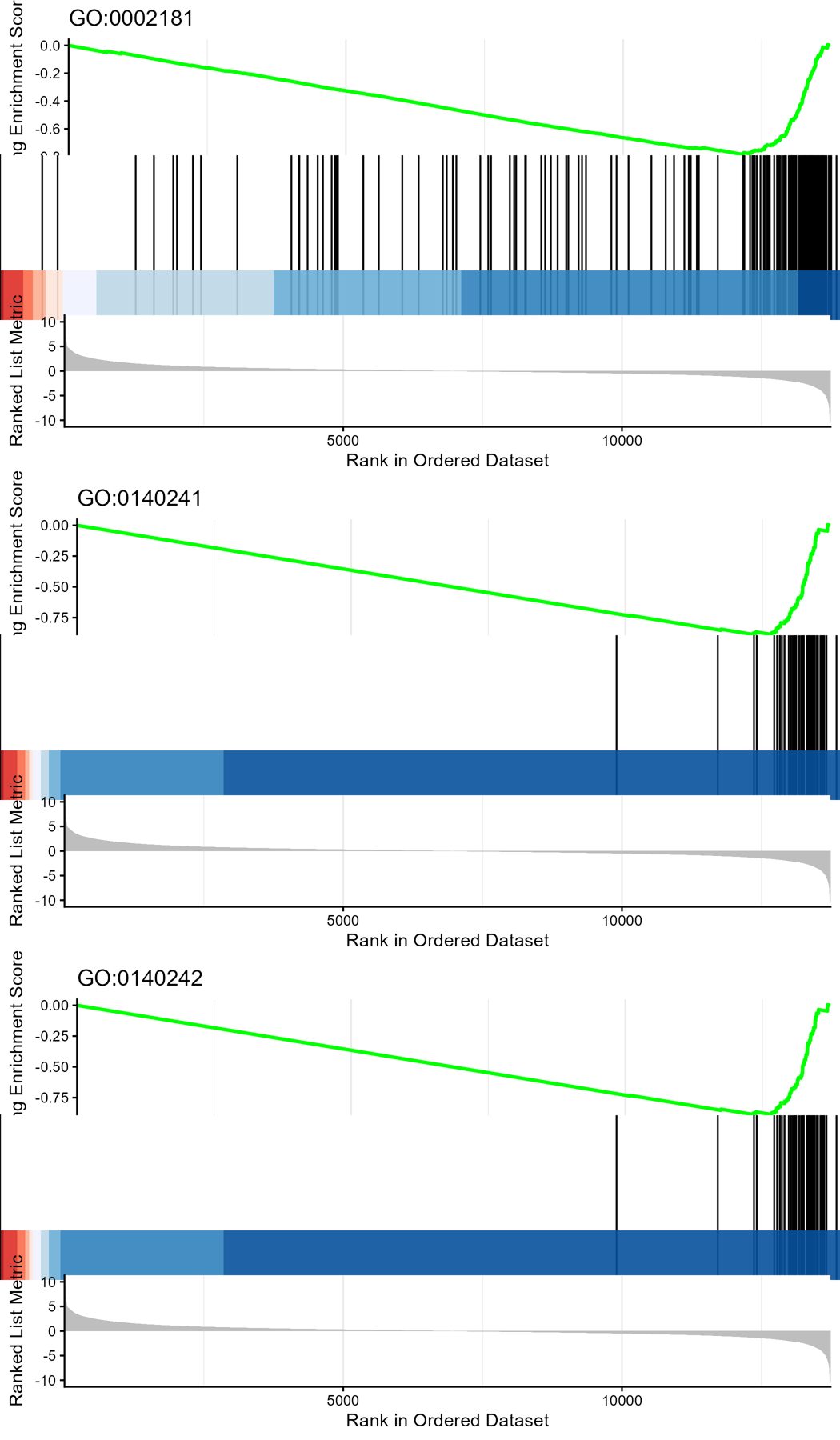


**Figure S3-092. Single-cell transcriptome analysis, step10_enrichment: 10 GSEA top pathway curves PT Sham vs IRI 12h**

# Section: step10_enrichment/10_leading_edge

This section has 3 blank-like image(s), which were omitted.


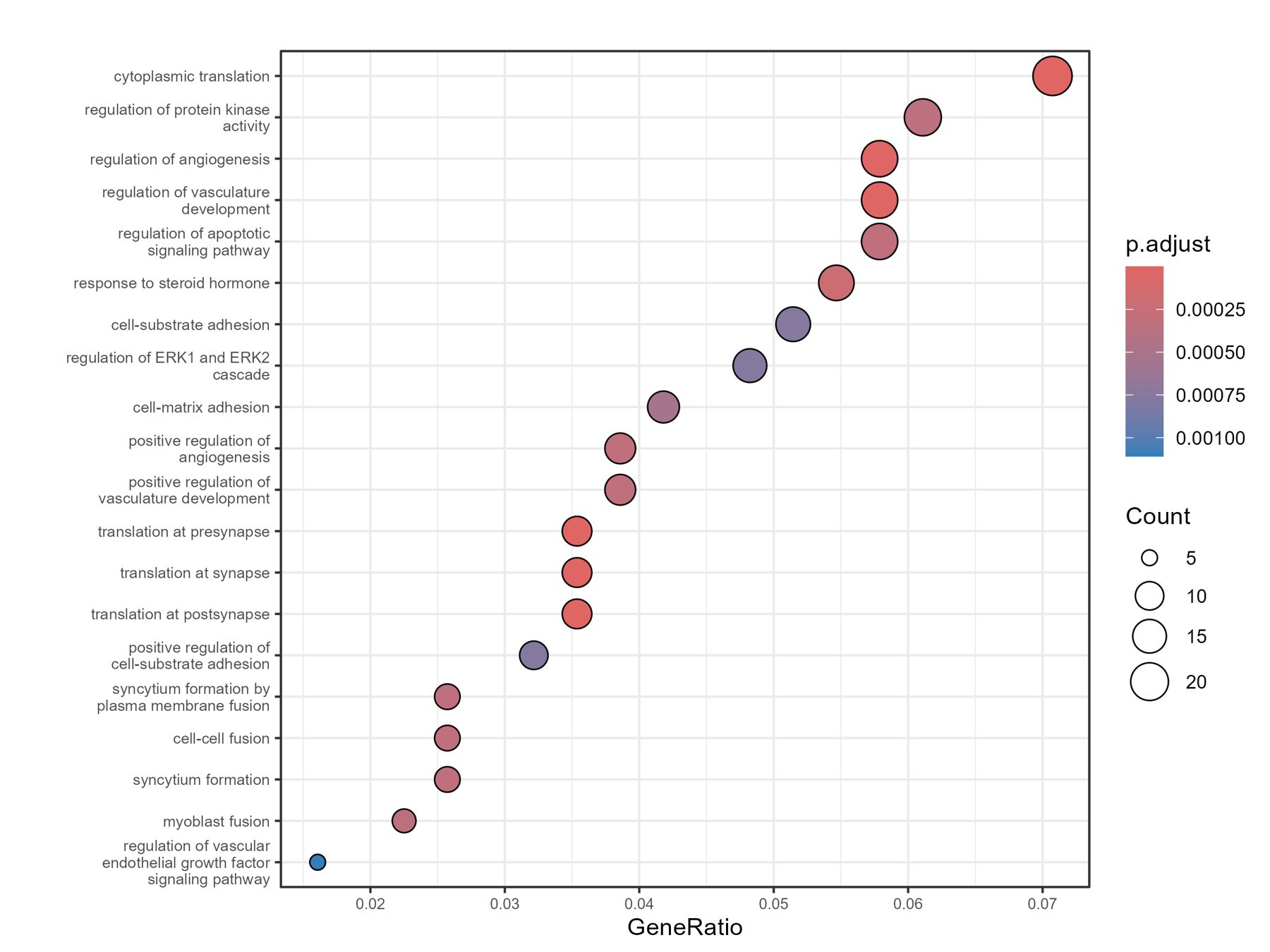


**Figure S3-093. Single-cell transcriptome analysis, step10_enrichment: 10 GO DOWN PT IRI 4h vs IRI 12h dotplot**


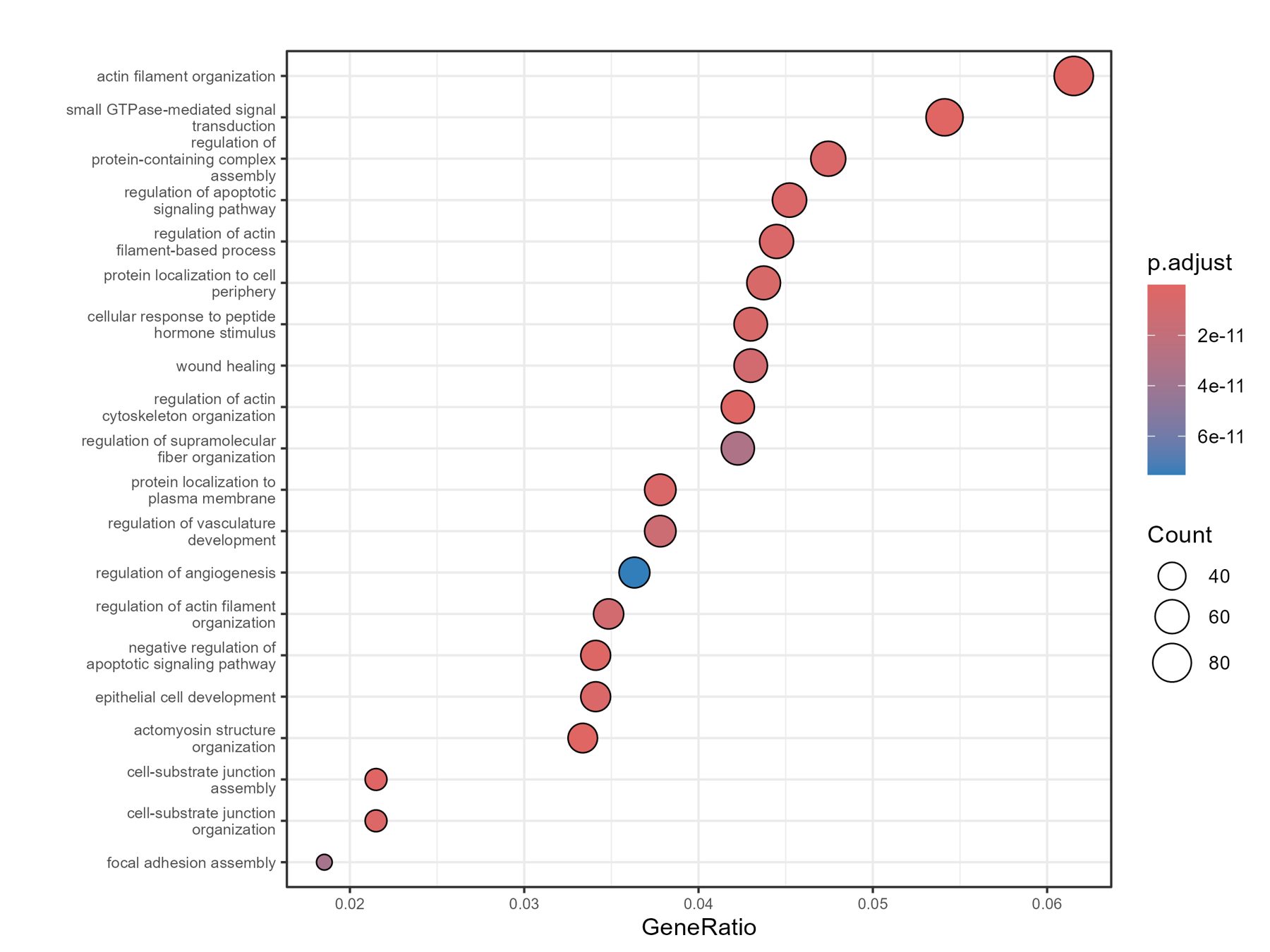


**Figure S3-094. Single-cell transcriptome analysis, step10_enrichment: 10 GO DOWN PT Sham vs IRI 4h dotplot**


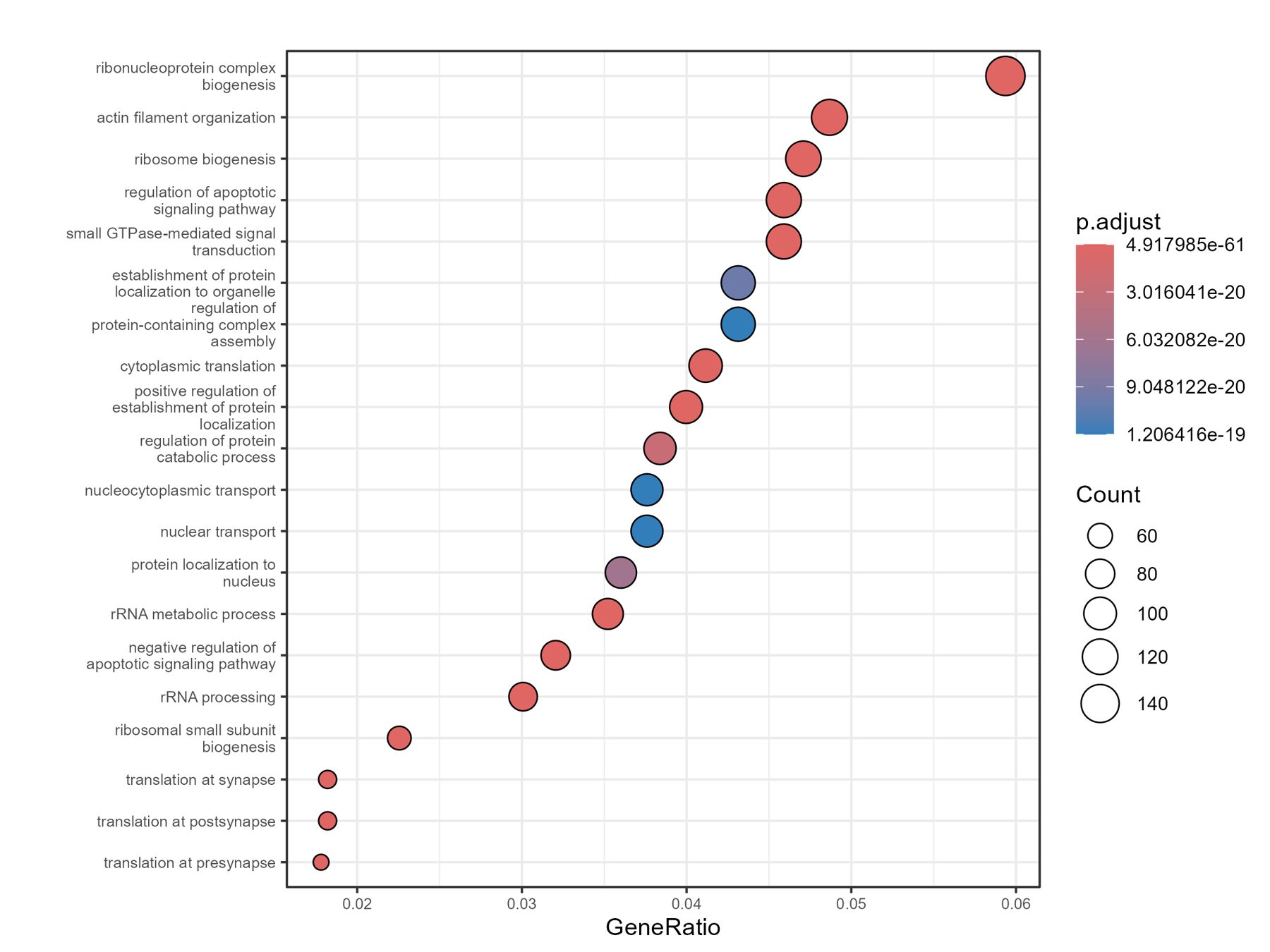


**Figure S3-095. Single-cell transcriptome analysis, step10_enrichment: 10 GO DOWN PT Sham vs IRI 12h dotplot**


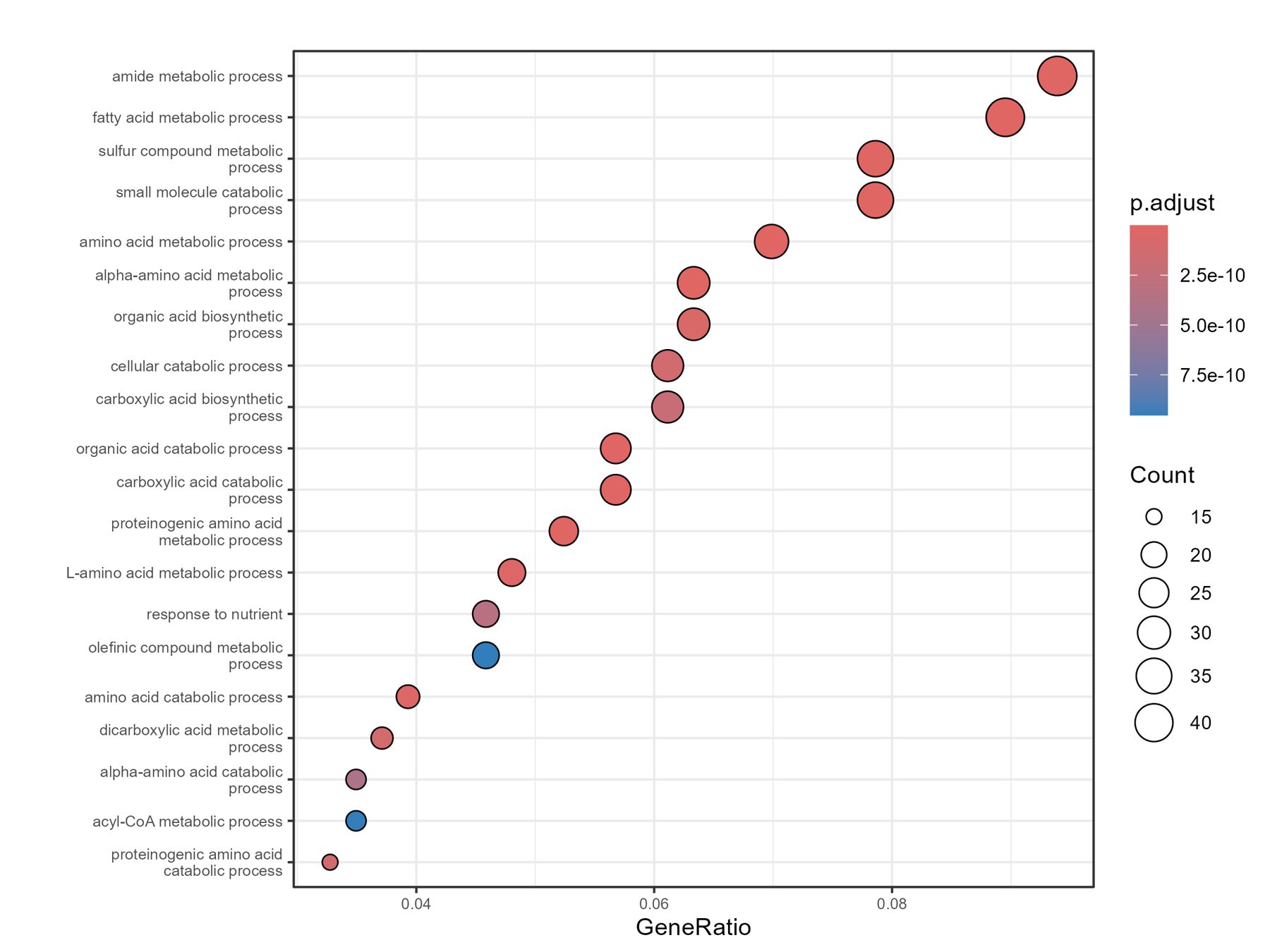


**Figure S3-096. Single-cell transcriptome analysis, step10_enrichment: 10 GO UP PT IRI 4h vs IRI 12h dotplot**


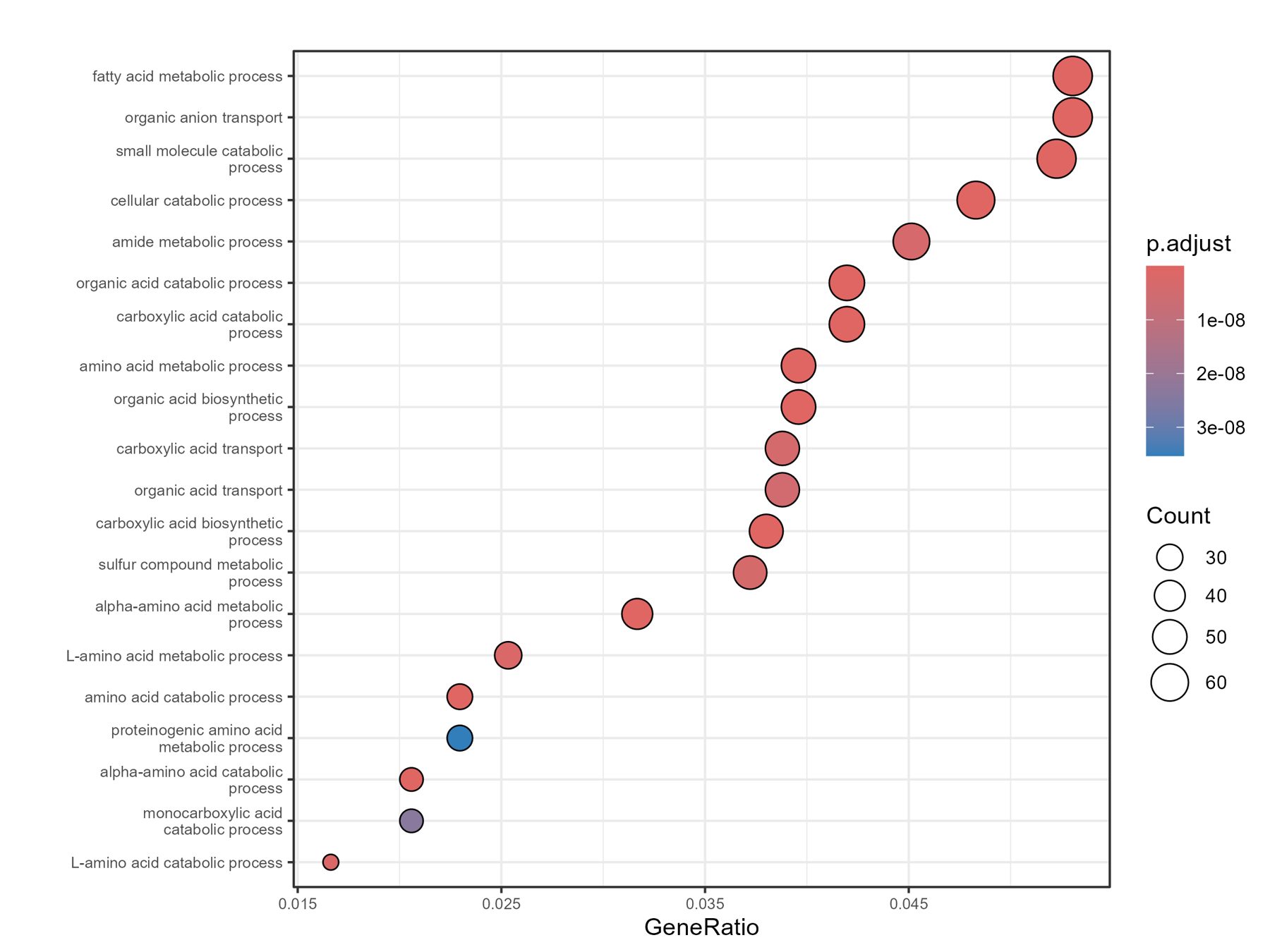


**Figure S3-097. Single-cell transcriptome analysis, step10_enrichment: 10 GO UP PT Sham vs IRI 4h dotplot**


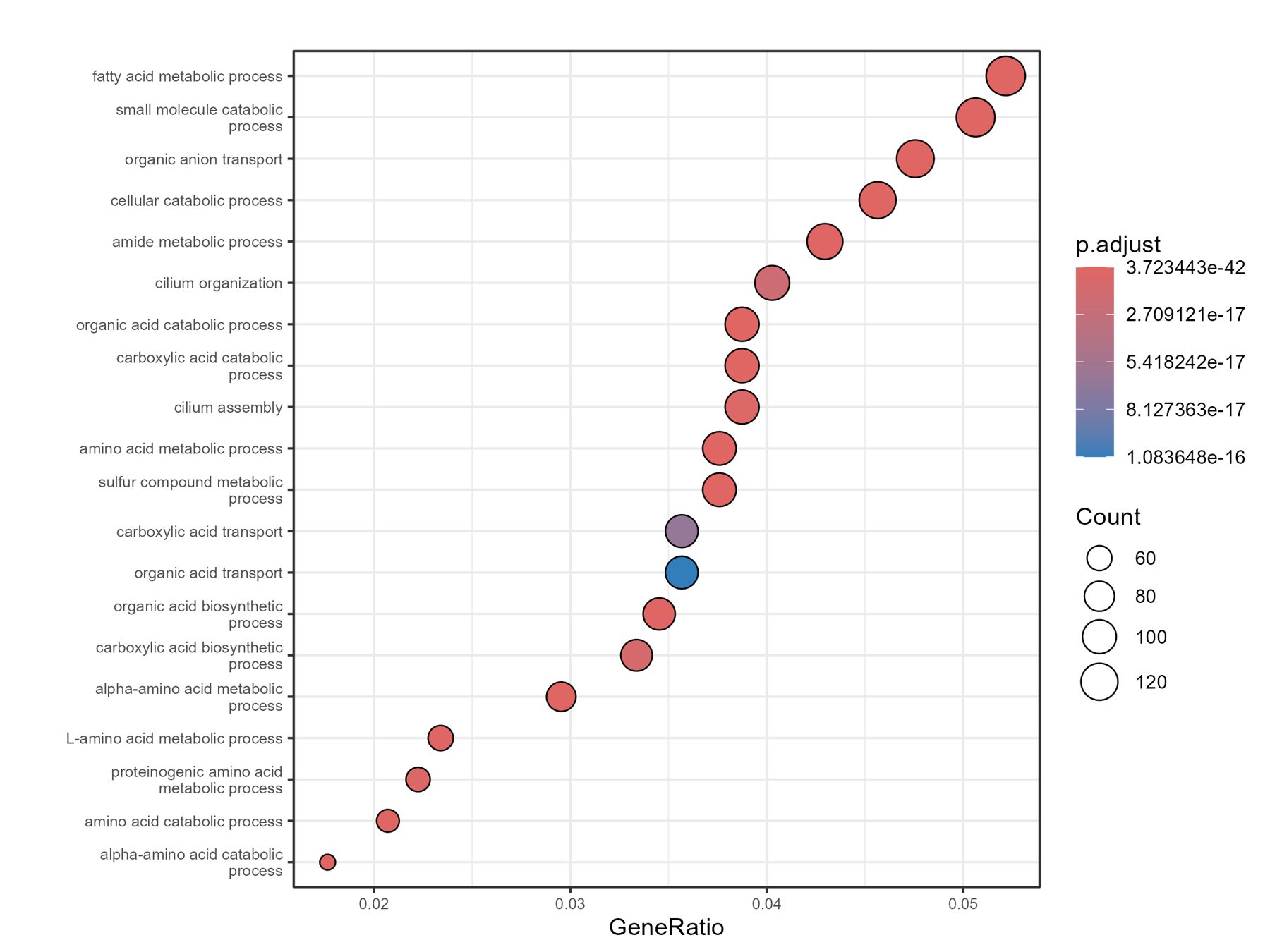


**Figure S3-098. Single-cell transcriptome analysis, step10_enrichment: 10 GO UP PT Sham vs IRI 12h dotplot**


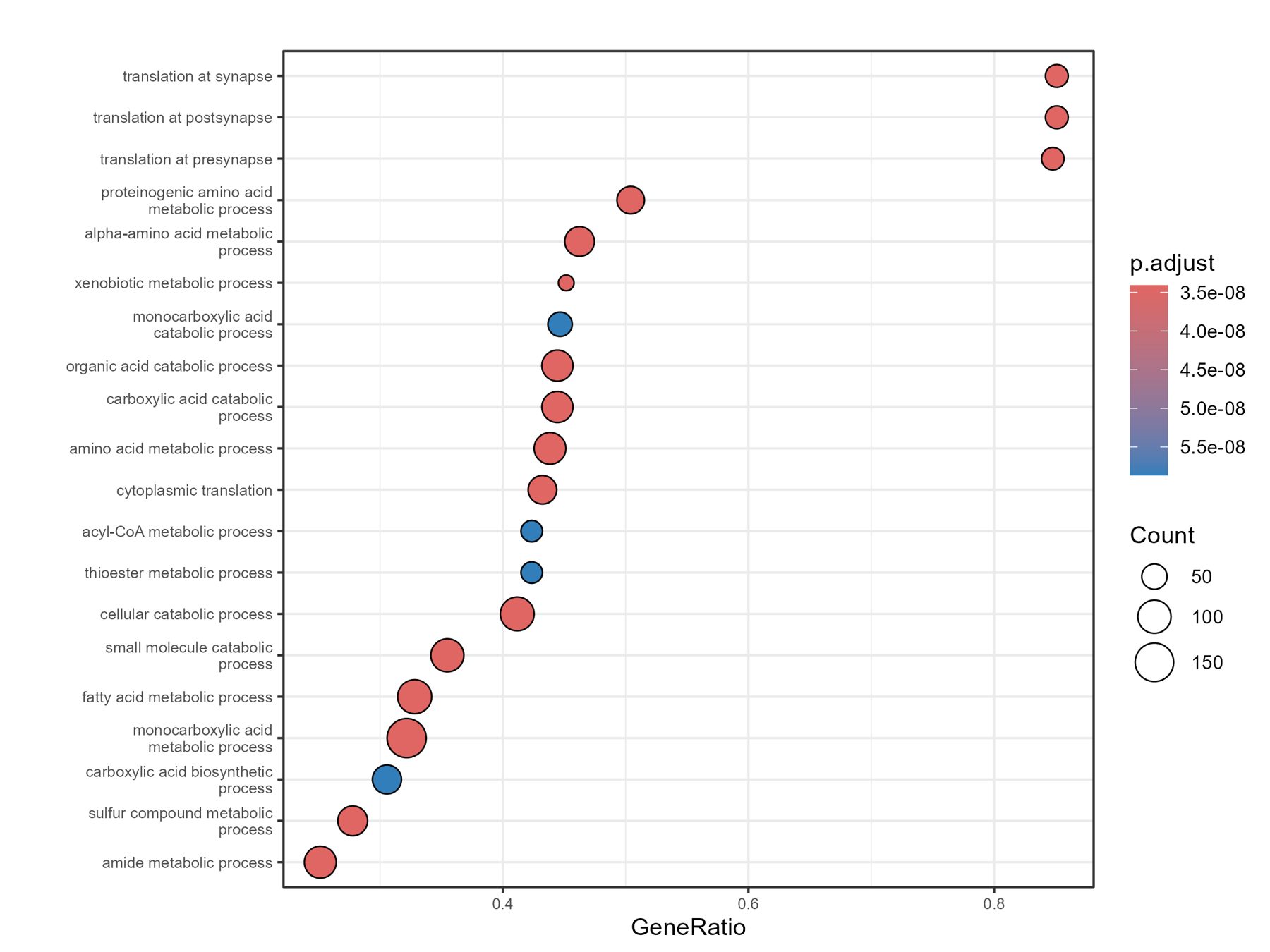


**Figure S3-099. Single-cell transcriptome analysis, step10_enrichment: 10 GSEA GO PT IRI 4h vs IRI 12h dotplot**


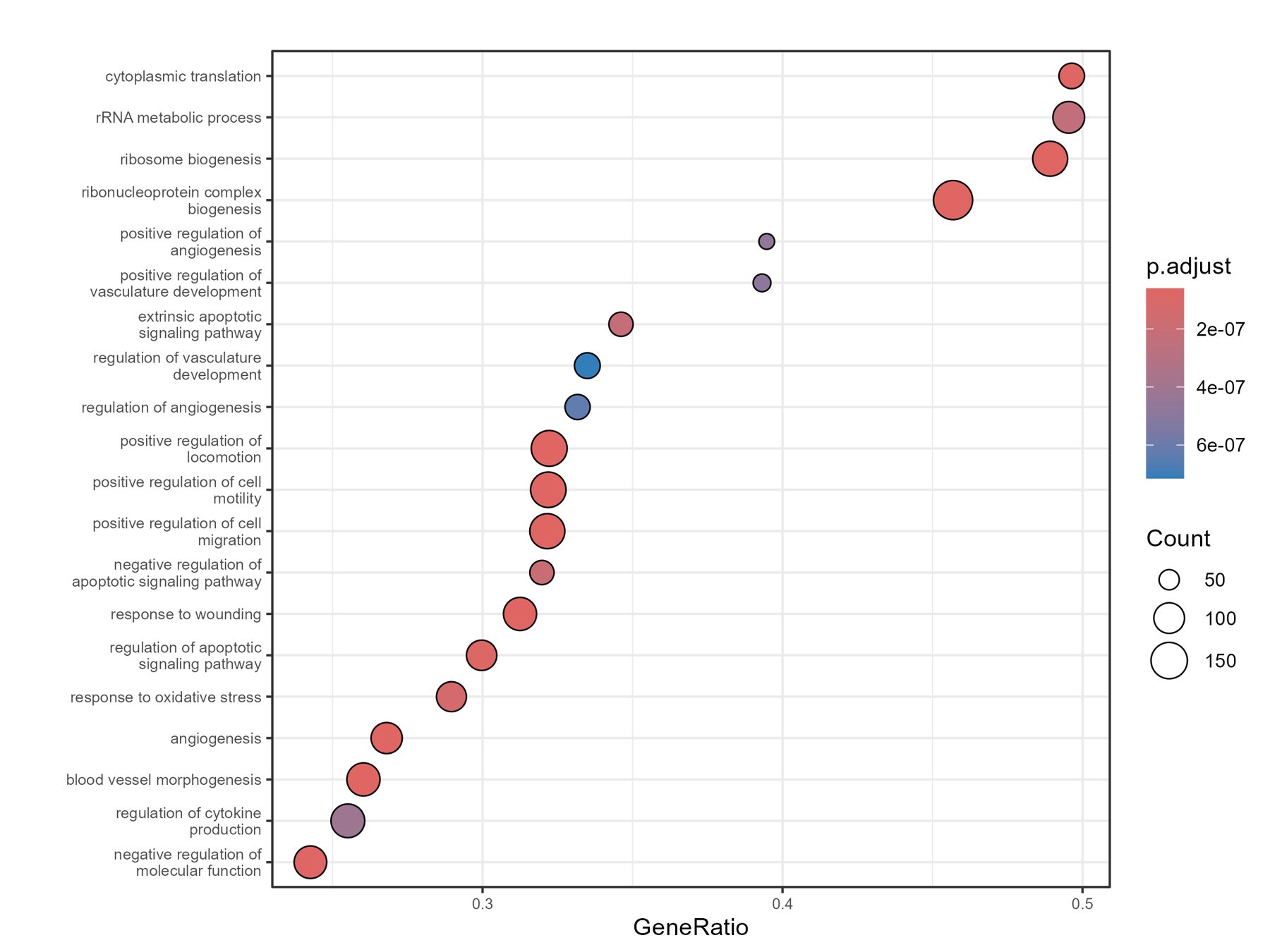


**Figure S3-100. Single-cell transcriptome analysis, step10_enrichment: 10 GSEA GO PT Sham vs IRI 4h dotplot**

**Figure S3-101. Single-cell transcriptome analysis, step10_enrichment: 10 GSEA GO PT Sham vs IRI 12h dotplot**

**Figure S3-102. Single-cell transcriptome analysis, step10_enrichment: 10 KEGG DOWN PT IRI 4h vs IRI 12h dotplot**

**Figure S3-103. Single-cell transcriptome analysis, step10_enrichment: 10 KEGG DOWN PT Sham vs IRI 4h dotplot**

**Figure S3-104. Single-cell transcriptome analysis, step10_enrichment: 10 KEGG DOWN PT Sham vs IRI 12h dotplot**

**Figure S3-105. Single-cell transcriptome analysis, step10_enrichment: 10 KEGG UP PT IRI 4h vs IRI 12h dotplot**

**Figure S3-106. Single-cell transcriptome analysis, step10_enrichment: 10 KEGG UP PT Sham vs IRI 4h dotplot**

**Figure S3-107. Single-cell transcriptome analysis, step10_enrichment: 10 KEGG UP PT Sham vs IRI 12h dotplot**

# Section: step11_cellchat

This section has 2 blank-like image(s), which were omitted.

**Figure S3-108. Single-cell transcriptome analysis, step11_cellchat: 11 cellchat bubble Endothelial to PTinjured topLR**

**Figure S3-109. Single-cell transcriptome analysis, step11_cellchat: 11 cellchat bubble Endothelial to PTinjured topLR IRI 12h**

**Figure S3-110. Single-cell transcriptome analysis, step11_cellchat: 11 cellchat bubble Mac Mono to PTinjured topLR**

**Figure S3-111. Single-cell transcriptome analysis, step11_cellchat: 11 cellchat bubble Mac Mono to PTinjured topLR IRI 12h**

**Figure S3-112. Single-cell transcriptome analysis, step11_cellchat: 11 cellchat compare interaction strength**

**Figure S3-113. Single-cell transcriptome analysis, step11_cellchat: 11 cellchat compare number interactions**

**Figure S3-114. Single-cell transcriptome analysis, step11_cellchat: 11 cellchat diff network IRI 4h vs IRI 12h**

**Figure S3-115. Single-cell transcriptome analysis, step11_cellchat: 11 cellchat diff network Sham vs IRI 4h**

**Figure S3-116. Single-cell transcriptome analysis, step11_cellchat: 11 cellchat diff network Sham vs IRI 12h**

**Figure S3-117. Single-cell transcriptome analysis, step11_cellchat: 11 cellchat incoming strength to PTinjured by sender**

**Figure S3-118. Single-cell transcriptome analysis, step11_cellchat: 11 cellchat net circle IRI 4h**

**Figure S3-119. Single-cell transcriptome analysis, step11_cellchat: 11 cellchat net circle IRI 12h**

**Figure S3-120. Single-cell transcriptome analysis, step11_cellchat: 11 cellchat net circle Sham**

**Figure S3-121. Single-cell transcriptome analysis, step11_cellchat: 11 cellchat topLR expression violin**

# Section: step12_keygenes

**Figure S3-122. Single-cell transcriptome analysis, step12_keygenes: 12 axis genes featureplot PT umap**

**Figure S3-123. Single-cell transcriptome analysis, step12_keygenes: 12 axis genes violin group**

**Figure S3-124. Single-cell transcriptome analysis, step12_keygenes: 12 axis genes violin PTstate**

**Figure S3-125. Single-cell transcriptome analysis, step12_keygenes: 12 keygenes dotplot group**

**Figure S3-126. Single-cell transcriptome analysis, step12_keygenes: 12 keygenes dotplot PTstate**

**Figure S3-127. Single-cell transcriptome analysis, step12_keygenes: 12 keygenes featureplot PT umap**

**Figure S3-128. Single-cell transcriptome analysis, step12_keygenes: 12 keygenes violin group**

**Figure S3-129. Single-cell transcriptome analysis, step12_keygenes: 12 keygenes violin PTstate**

# Section: step13_pseudotime

**Figure S3-130. Single-cell transcriptome analysis, step13_pseudotime: 13 PT meanApop vs pseudotime samplelevel**

**Figure S3-131. Single-cell transcriptome analysis, step13_pseudotime: 13 PT meanATF6score vs pseudotime samplelevel**

**Figure S3-132. Single-cell transcriptome analysis, step13_pseudotime: 13 PT meanIRE1score vs pseudotime samplelevel**

**Figure S3-133. Single-cell transcriptome analysis, step13_pseudotime: 13 PT meanPERKscore vs pseudotime samplelevel**

**Figure S3-134. Single-cell transcriptome analysis, step13_pseudotime: 13 PT meanUPR vs pseudotime samplelevel**

**Figure S3-135. Single-cell transcriptome analysis, step13_pseudotime: 13 PT pseudotime branch plots**

**Figure S3-136. Single-cell transcriptome analysis, step13_pseudotime: 13 PT pseudotime branch plots mainpath arrow**

**Figure S3-137. Single-cell transcriptome analysis, step13_pseudotime: 13 PT pseudotime branch plots mainpath start end**

**Figure S3-138. Single-cell transcriptome analysis, step13_pseudotime: 13 PT pseudotime by sample point boxplot**

**Figure S3-139. Single-cell transcriptome analysis, step13_pseudotime: 13 PT pseudotime dynamic genes heatmap**

**Figure S3-140. Single-cell transcriptome analysis, step13_pseudotime: 13 PT pseudotime ridge by group**

**Figure S3-141. Single-cell transcriptome analysis, step13_pseudotime: 13 PT pseudotime topgenes heatmap**

**Figure S3-142. Single-cell transcriptome analysis, step13_pseudotime: 13 PT pseudotime umap**

**Figure S3-143. Single-cell transcriptome analysis, step13_pseudotime: 13 PT pseudotime umap splitby group**

**Figure S3-144. Single-cell transcriptome analysis, step13_pseudotime: 13 PT pseudotime violin by PTstate**

**Figure S3-145. Single-cell transcriptome analysis, step13_pseudotime: 13 PT score vs pseudotime Apop**

**Figure S3-146. Single-cell transcriptome analysis, step13_pseudotime: 13 PT score vs pseudotime UPR**

# Section: step13_pseudotime/extra/monocle2_ddrtree/monocle2_ddrtree

**Figure S3-147. Single-cell transcriptome analysis, step13_pseudotime: m2 pseudotime ridge by group**

**Figure S3-148. Single-cell transcriptome analysis, step13_pseudotime: m2 pseudotime sample boxplot**

**Figure S3-149. Single-cell transcriptome analysis, step13_pseudotime: m2 pseudotime trajectory**

**Figure S3-150. Single-cell transcriptome analysis, step13_pseudotime: m2 trajectory by group**

**Figure S3-151. Single-cell transcriptome analysis, step13_pseudotime: m2 trajectory by PTstate**

# Section: step14_chip_integration

**Figure S3-152. Single-cell transcriptome analysis, step14_chip_integration: 14 pathway alignment bar**

**Figure S3-153. Single-cell transcriptome analysis, step14_chip_integration: 14 pathway alignment dotplot**

**Figure S3-154. Single-cell transcriptome analysis, step14_chip_integration: 14 pathway alignment heatmap**

**Figure S3-155. Single-cell transcriptome analysis, step14_chip_integration: 14 pathway alignment slope 4h**

**Figure S3-156. Single-cell transcriptome analysis, step14_chip_integration: 14 pathway alignment slope 12h**
