# Supplementary material for: Integrated transcriptomics identifies ER stress–associated apoptosis in post-resuscitation AKI and supports early Dl-3-n-butylphthalide–associated renoprotection in a porcine TCA model
Source: Front Pharmacol. 2026 Jun 4;17:1841271. doi: 10.3389/fphar.2026.1841271 (PMC13275486; doi:10.3389/fphar.2026.1841271)
Supplement: Supplementary file 6 [file Supplementaryfile2.docx]

**Supplementary File 2: Bulk Transcriptome Figures**

# Section: step1

This section has 2 blank-like image(s), which were omitted.


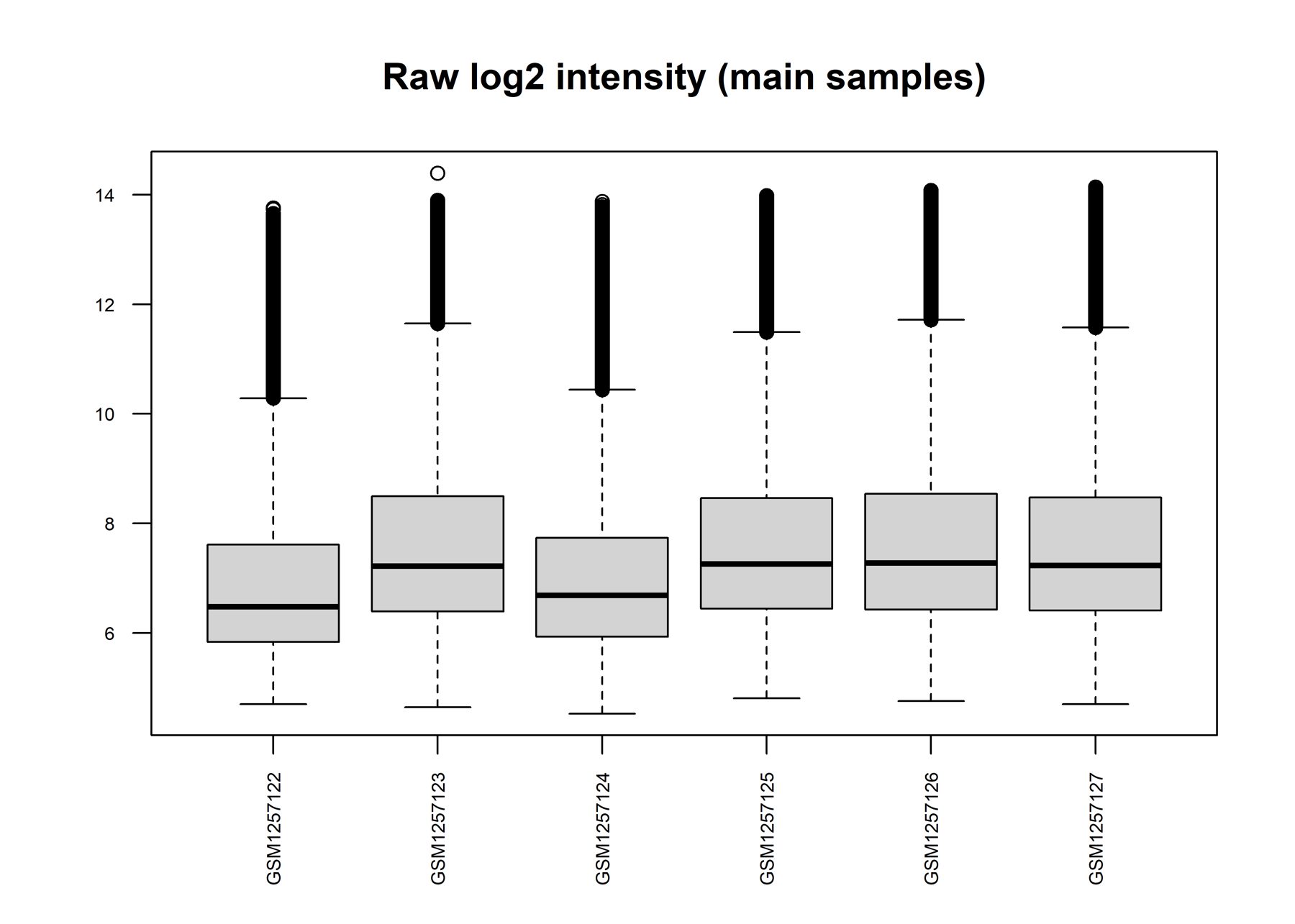


**Figure S2-001. Bulk transcriptome analysis, step1: bulk 01 boxplot raw intensity**


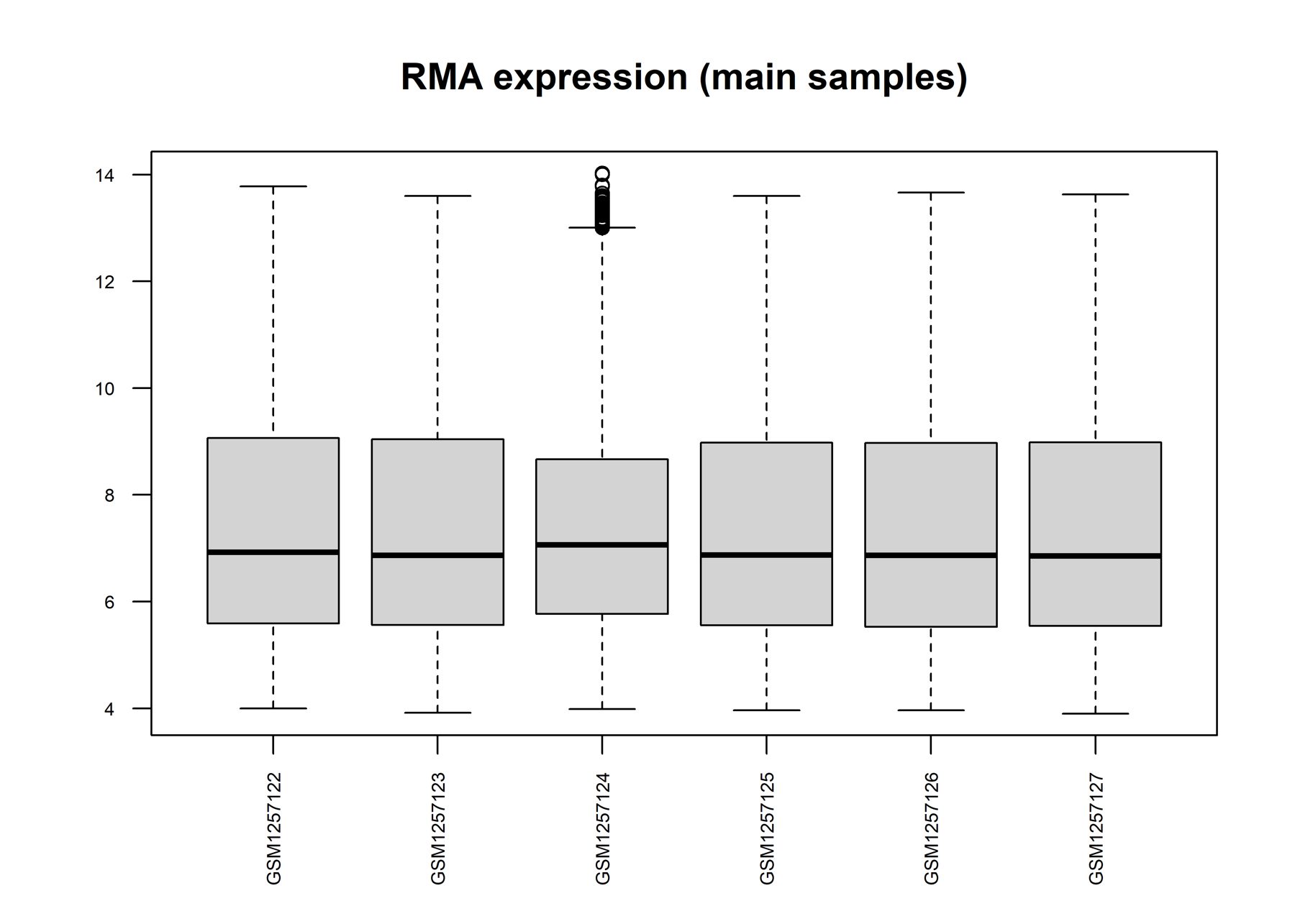


**Figure S2-002. Bulk transcriptome analysis, step1: bulk 01 boxplot rma expr**


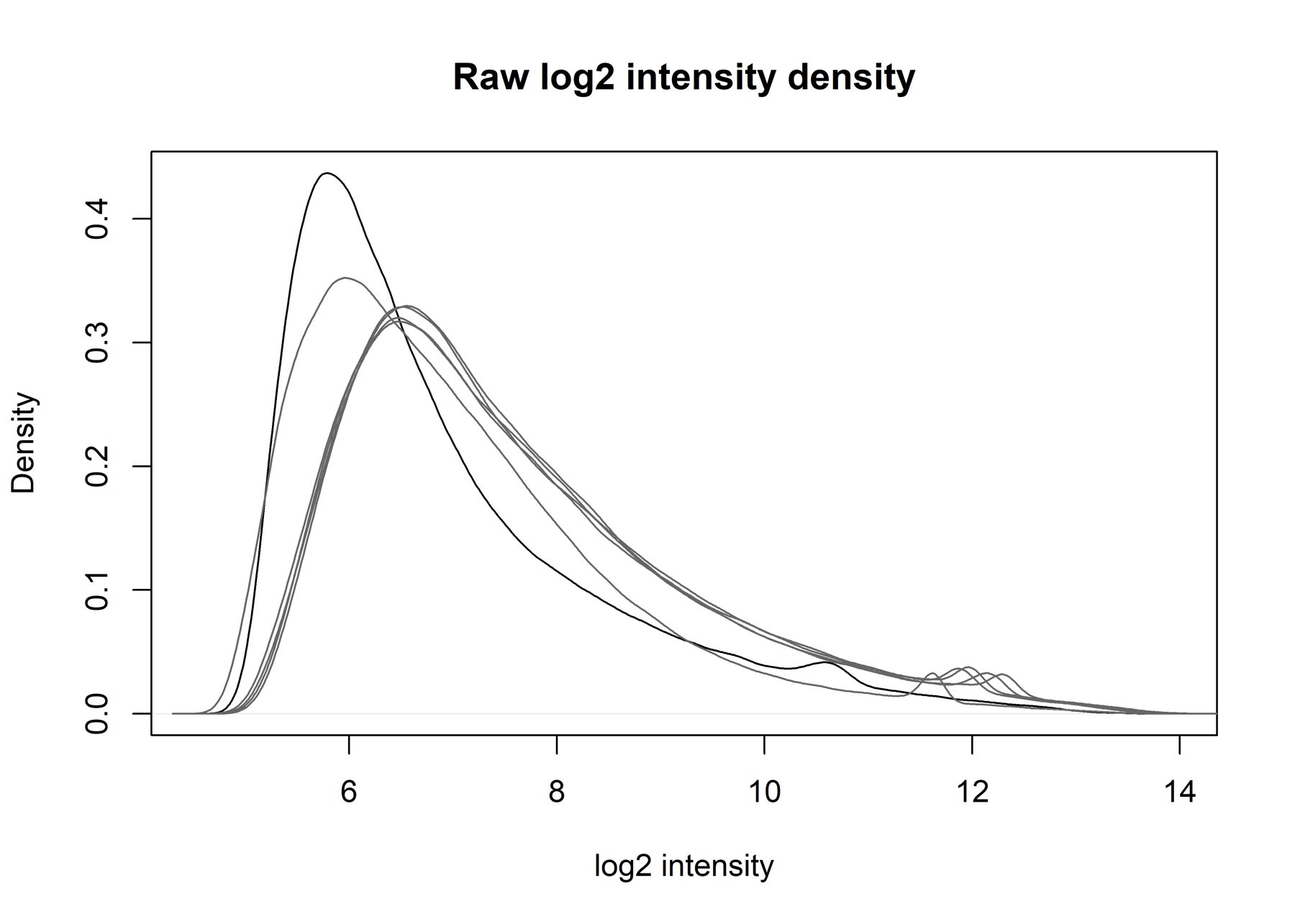


**Figure S2-003. Bulk transcriptome analysis, step1: bulk 01 density raw intensity**


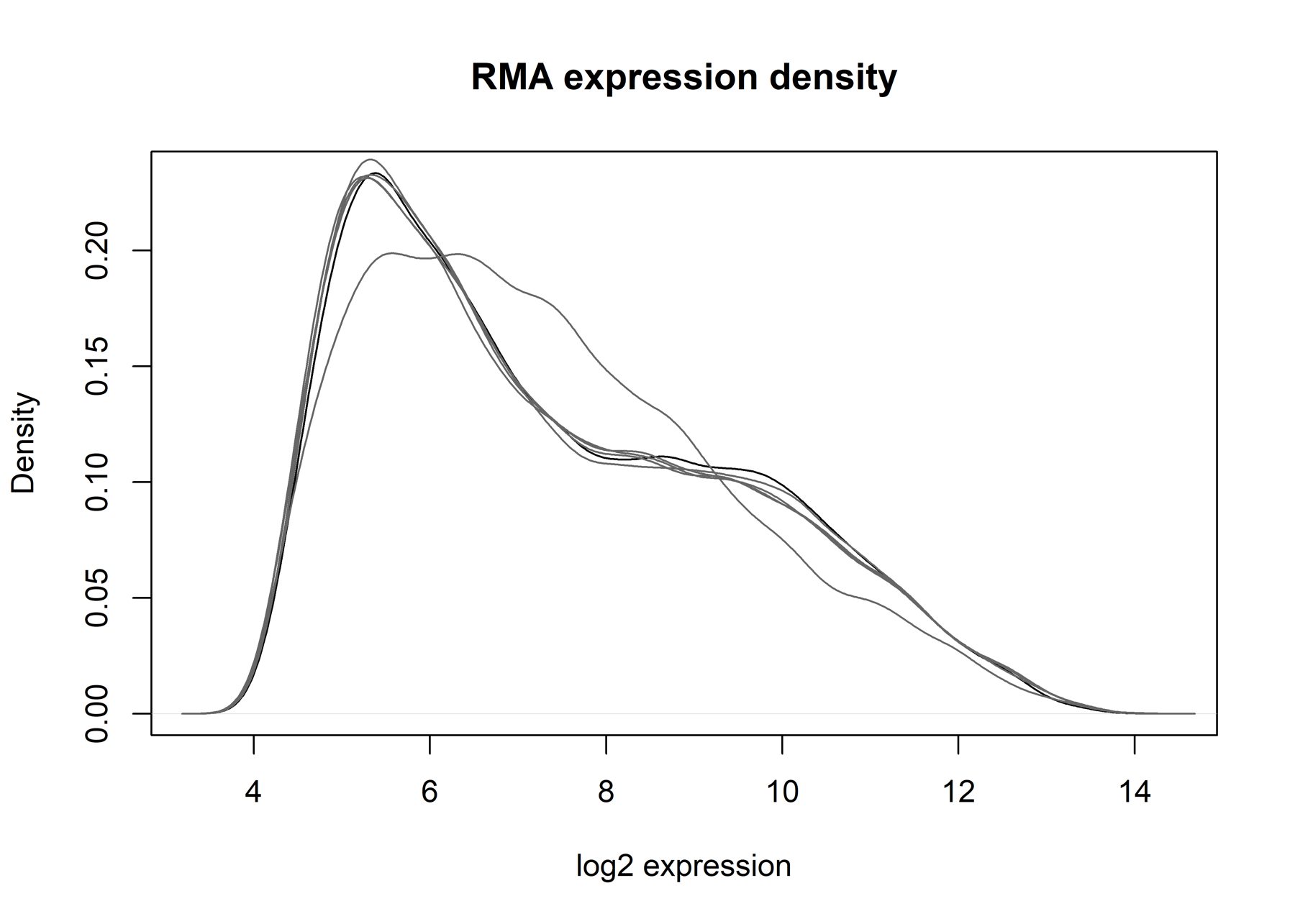


**Figure S2-004. Bulk transcriptome analysis, step1: bulk 01 density rma expr**


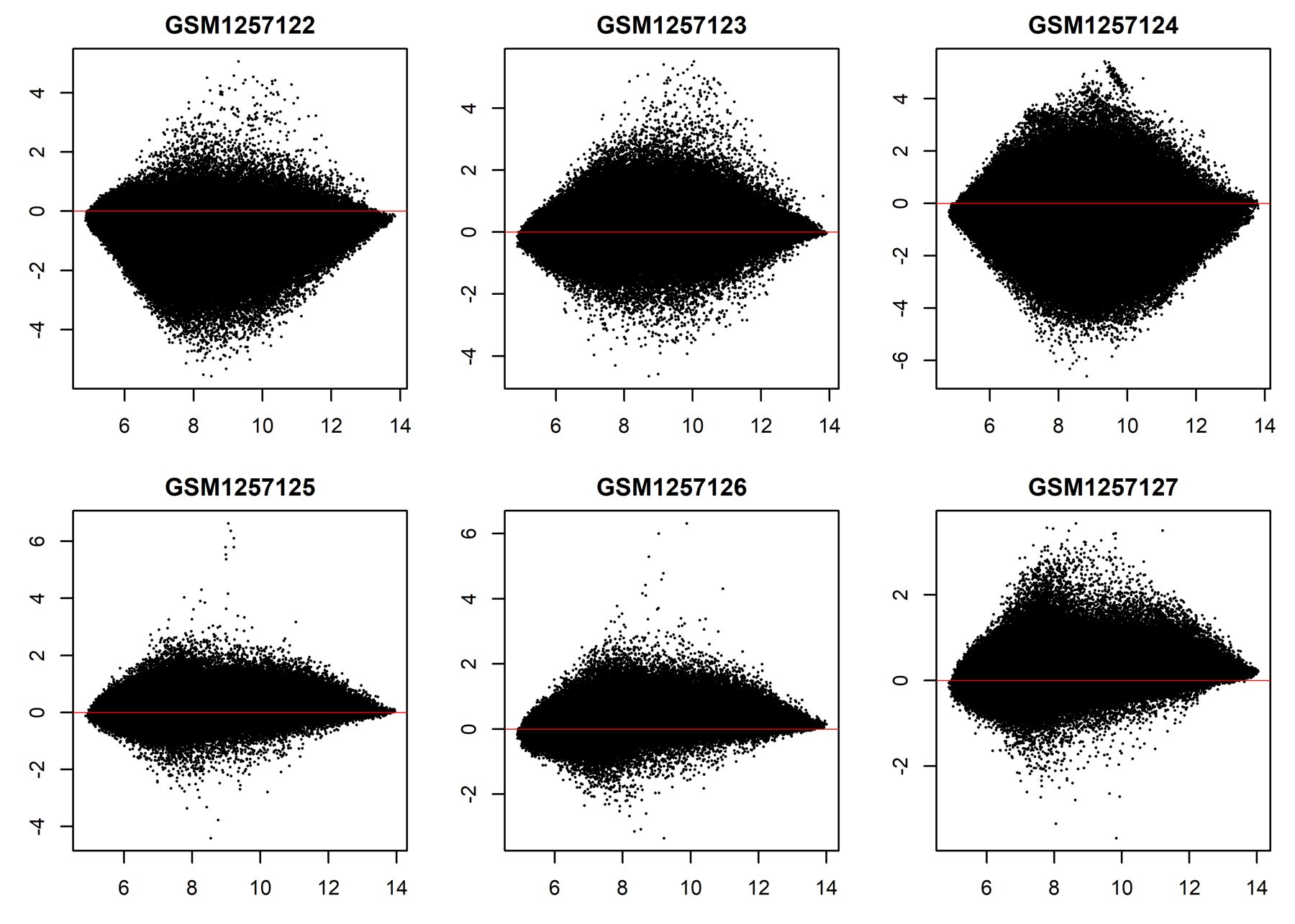


**Figure S2-005. Bulk transcriptome analysis, step1: bulk 01 MAplots raw**


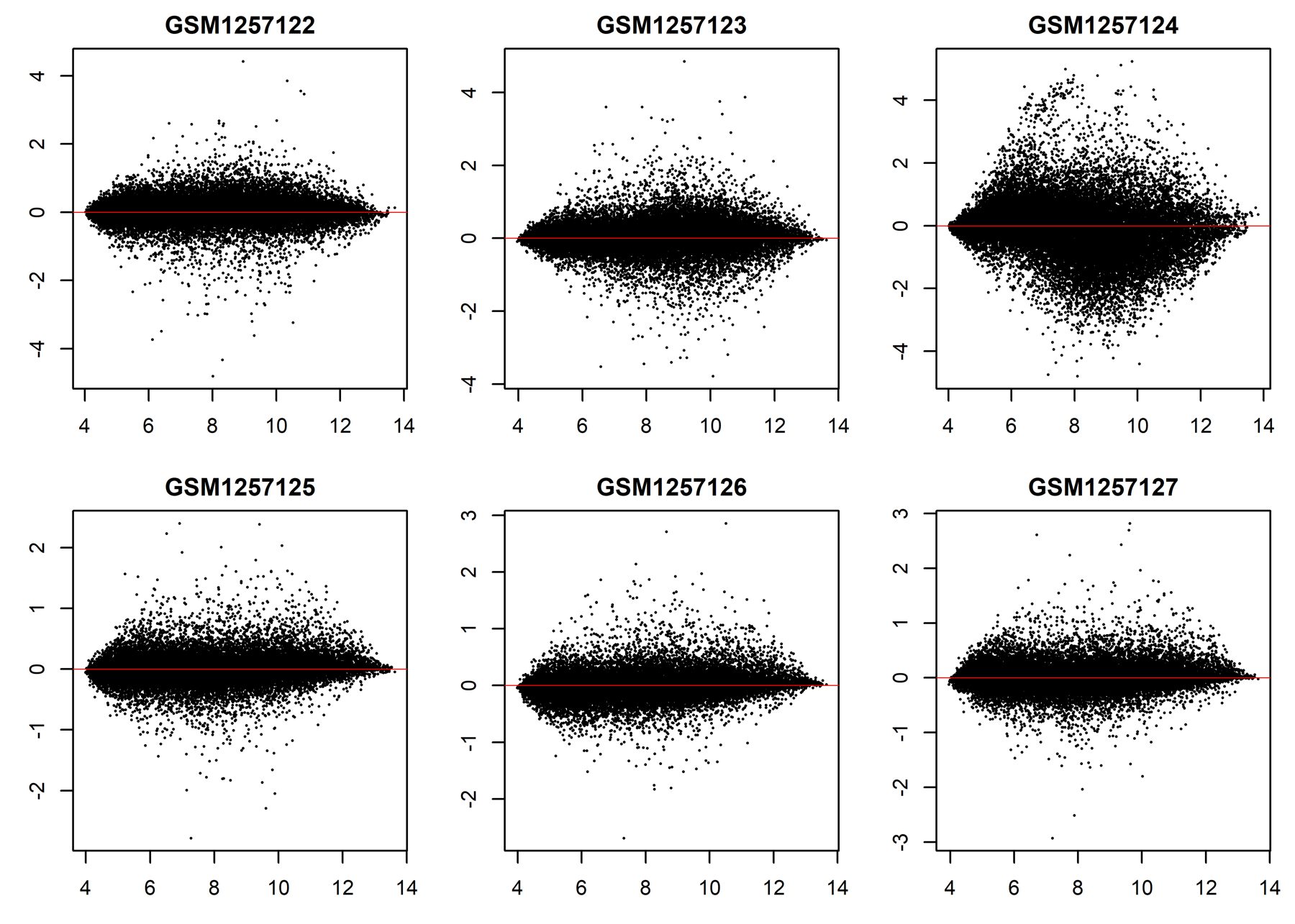


**Figure S2-006. Bulk transcriptome analysis, step1: bulk 01 MAplots rma**


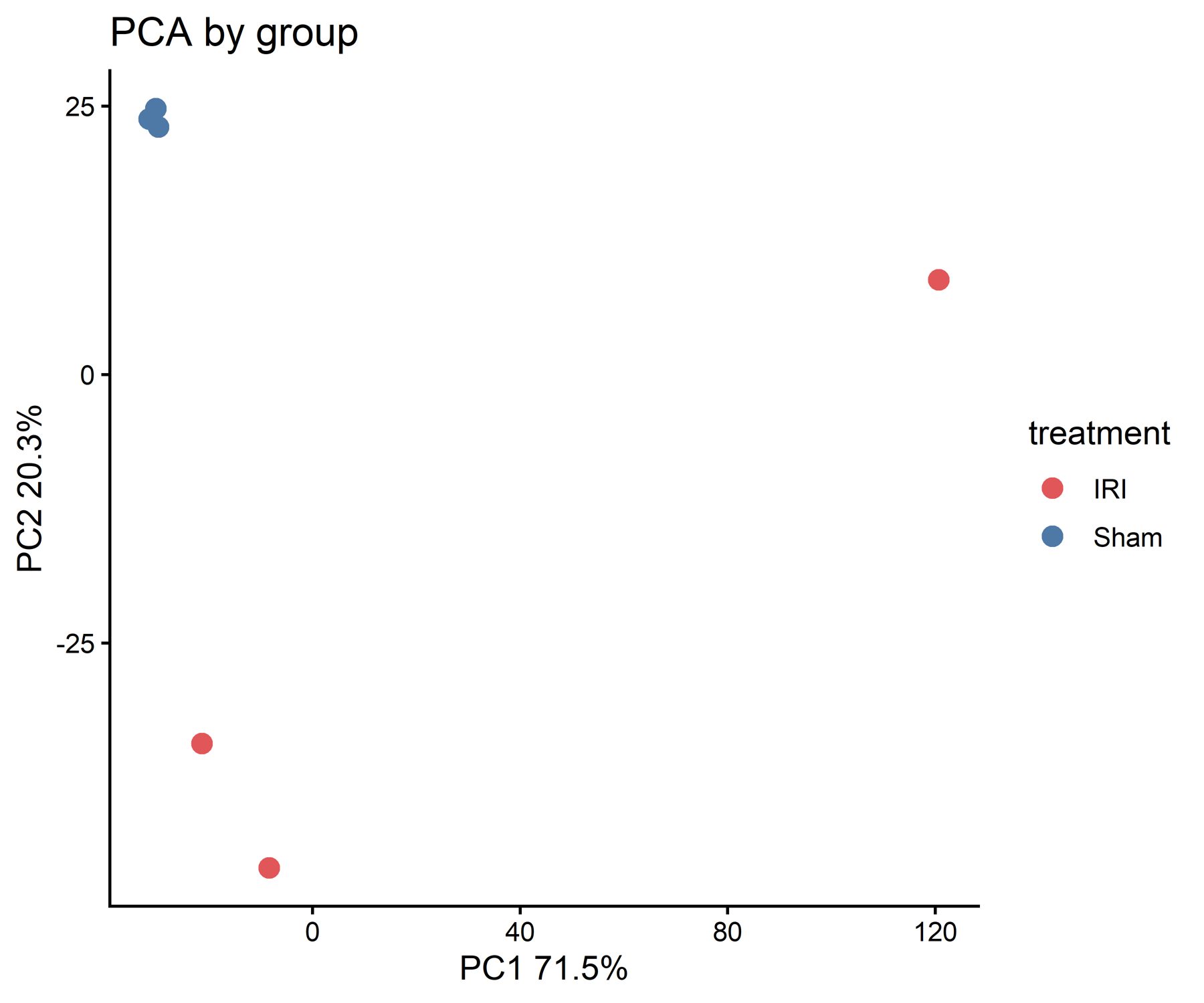


**Figure S2-007. Bulk transcriptome analysis, step1: bulk 01 PCA by group**


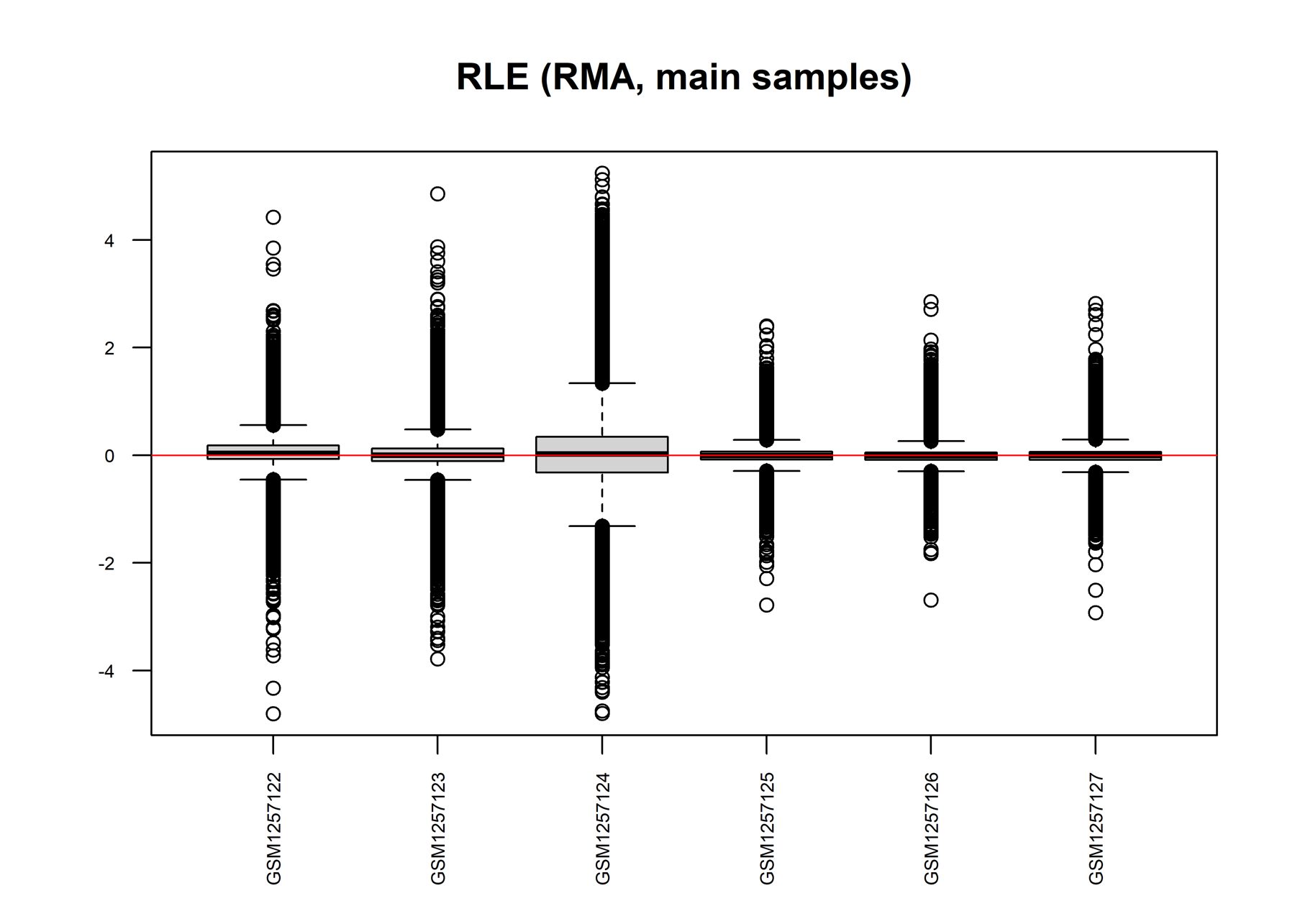


**Figure S2-008. Bulk transcriptome analysis, step1: bulk 01 RLE**


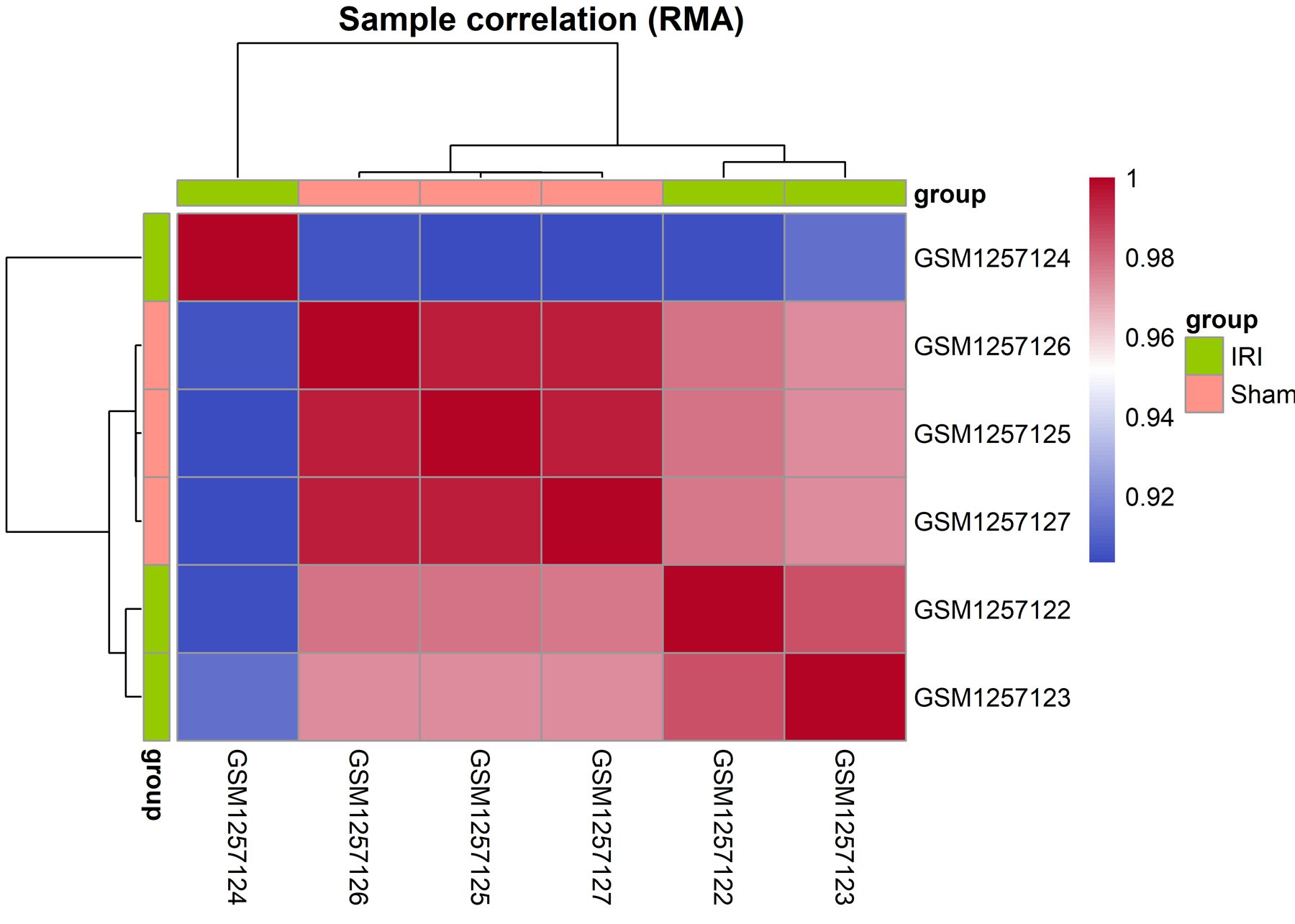


# Section: step2

This section has 6 blank-like image(s), which were omitted.


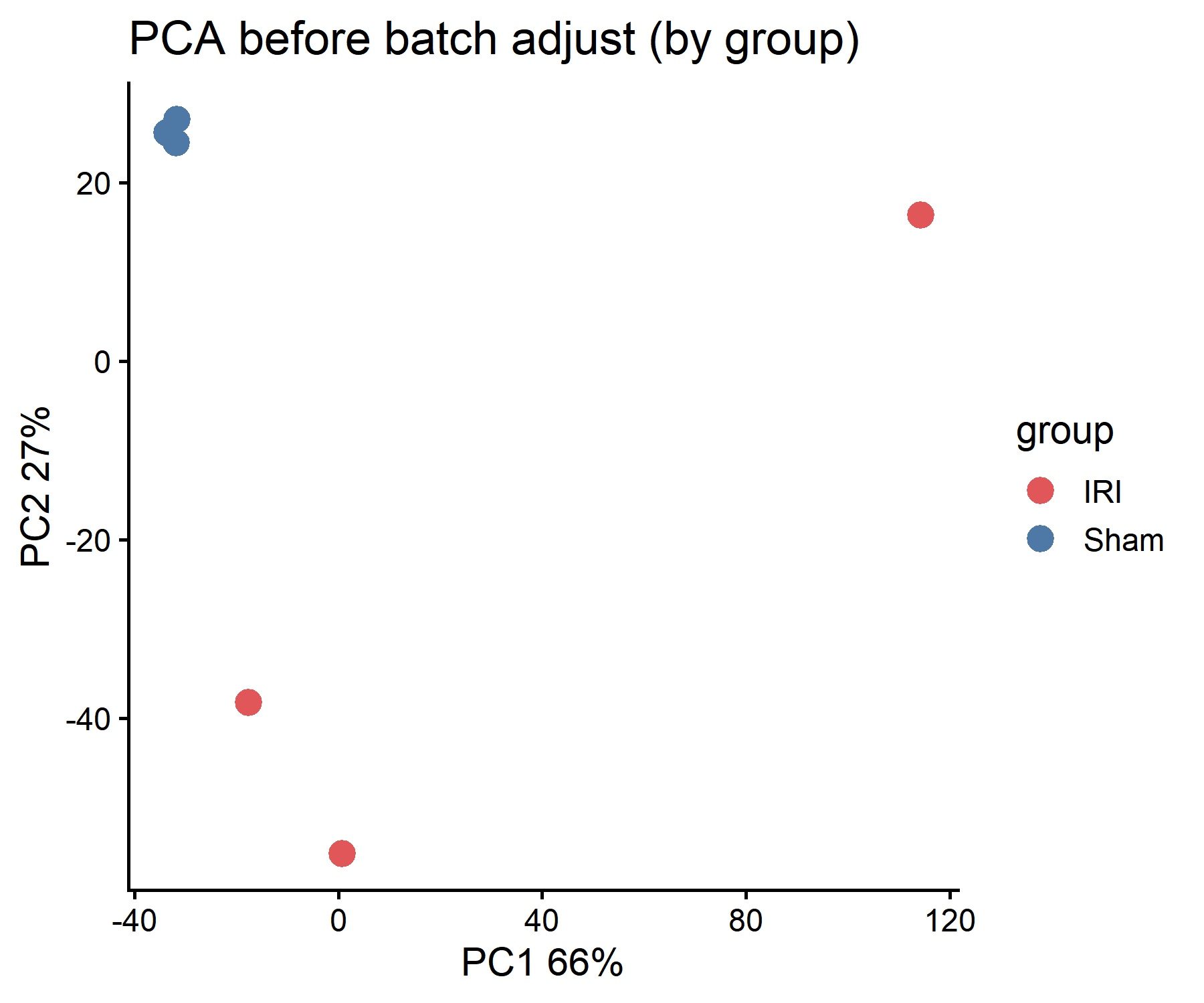


**Figure S2-010. Bulk transcriptome analysis, step2: bulk 02 PCA before batch adjust by group**


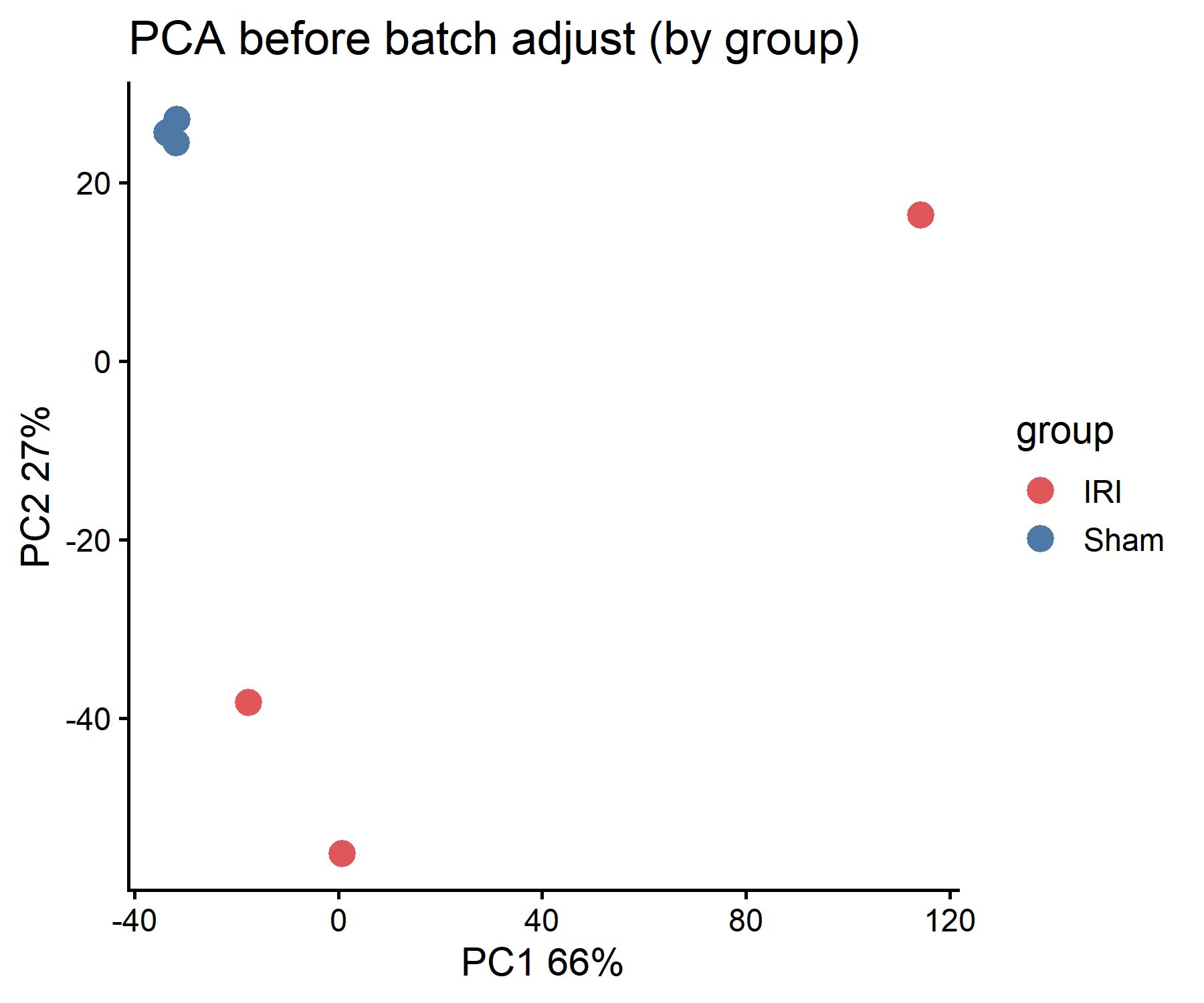


**Figure S2-011. Bulk transcriptome analysis, step2: bulk 02 PCA before batch adjust by group**

# Section: step3


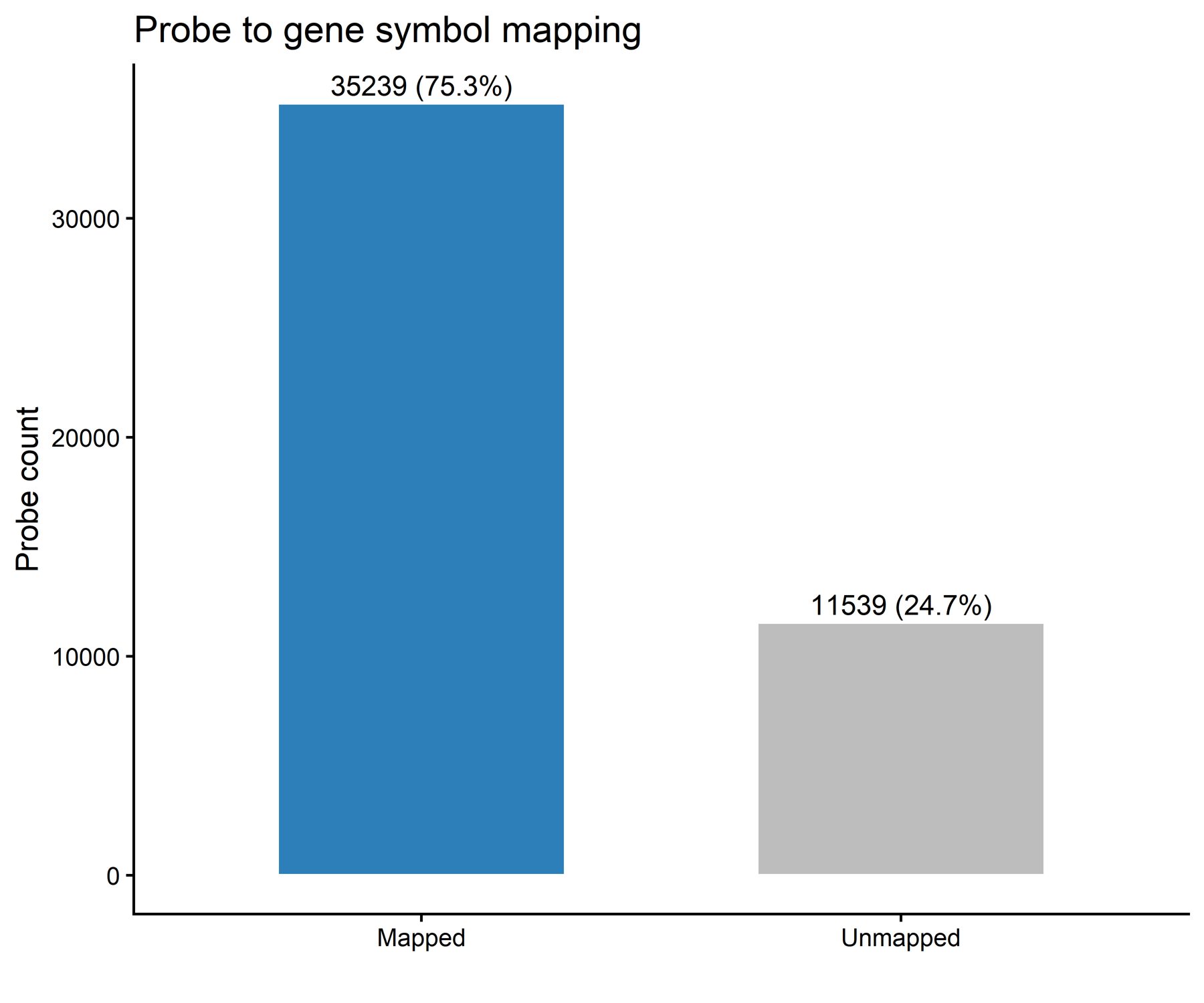


**Figure S2-012. Bulk transcriptome analysis, step3: bulk 03 probe mapping rate**


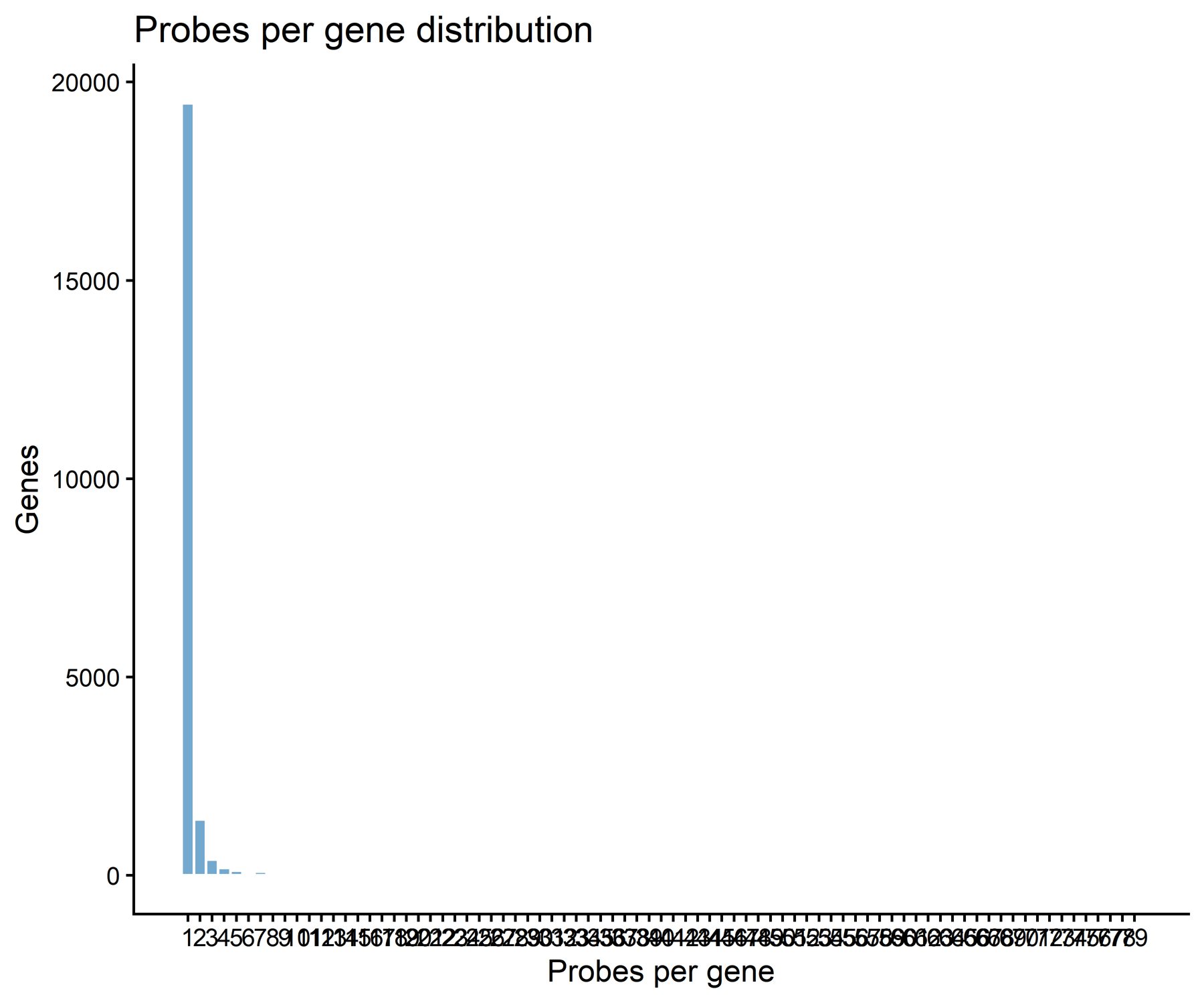


**Figure S2-013. Bulk transcriptome analysis, step3: bulk 03 probes per gene distribution**

# Section: step4


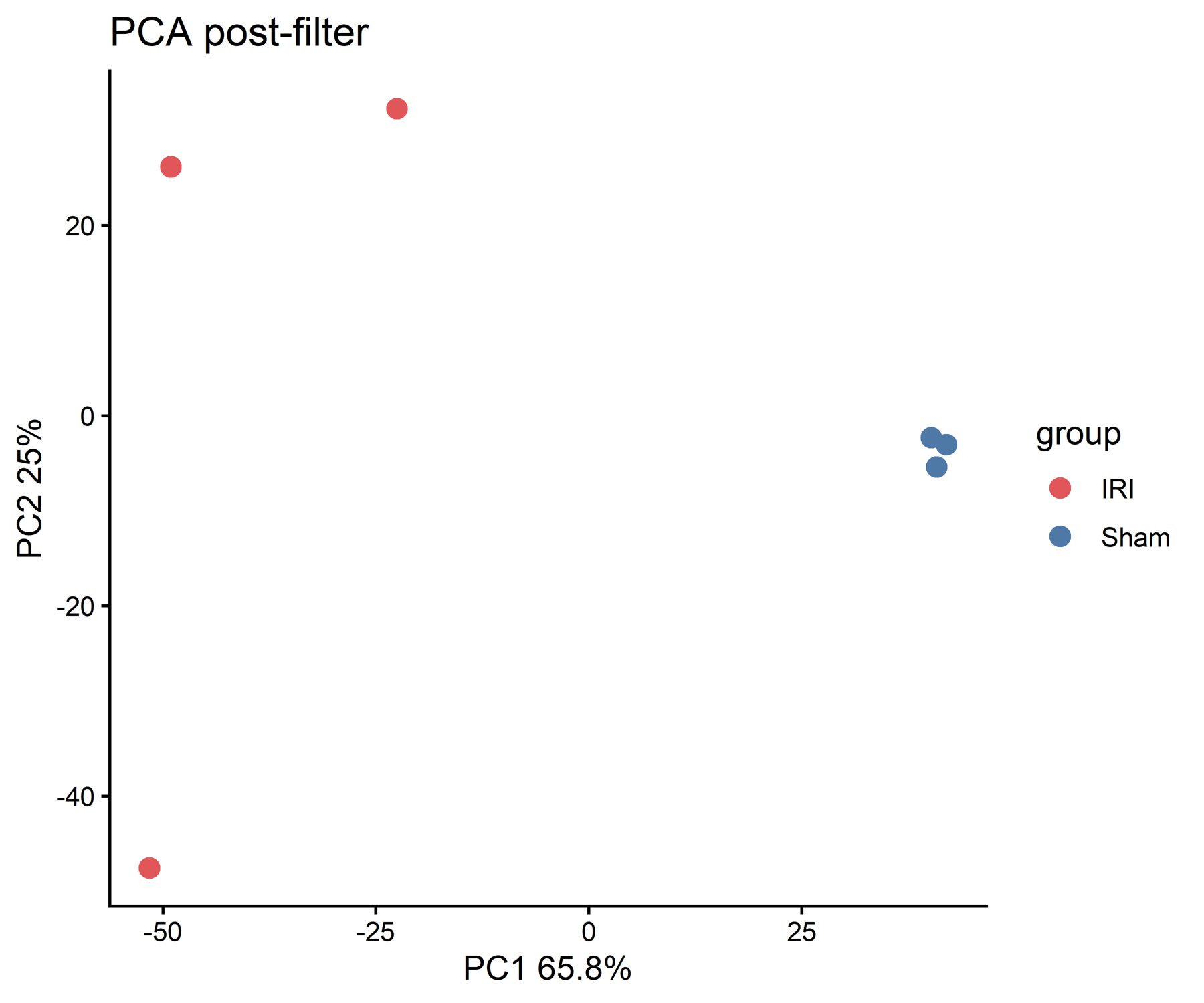


**Figure S2-014. Bulk transcriptome analysis, step4: bulk 04 PCA postfilter by group**


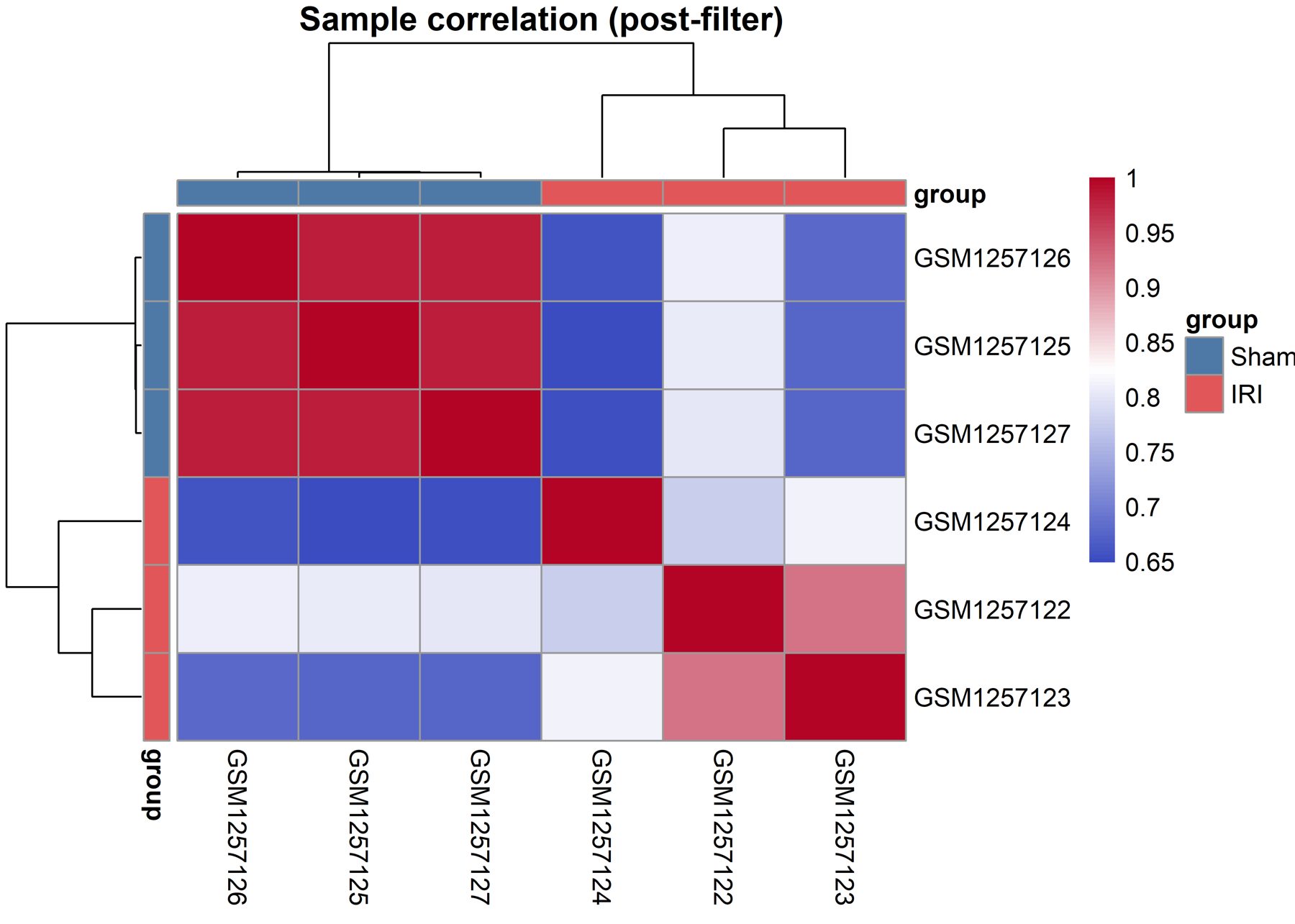


**Figure S2-015. Bulk transcriptome analysis, step4: bulk 04 sample correlation postfilter**

# Section: step5


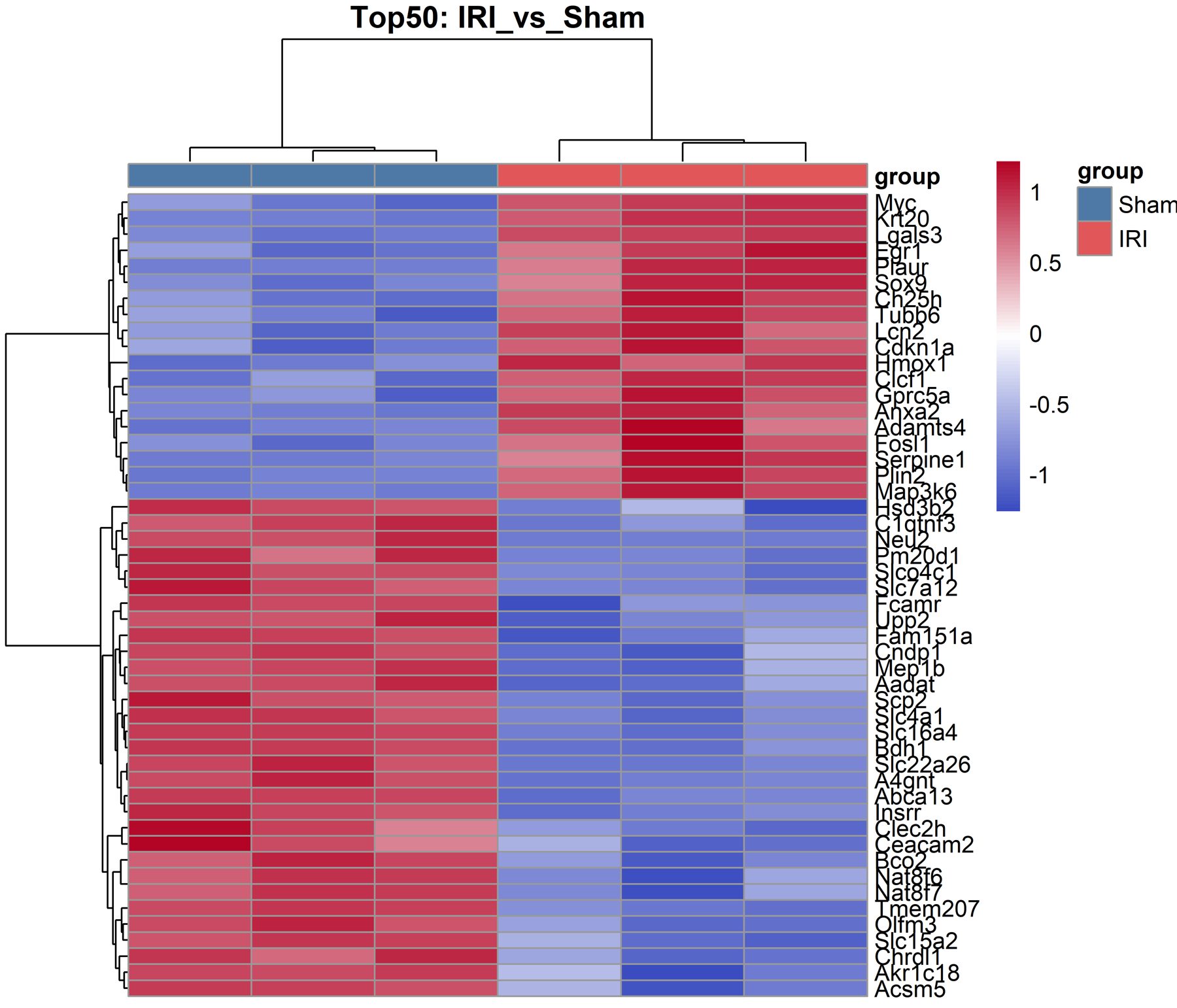


**Figure S2-016. Bulk transcriptome analysis, step5: bulk 05 heatmap top50 IRI vs Sham**


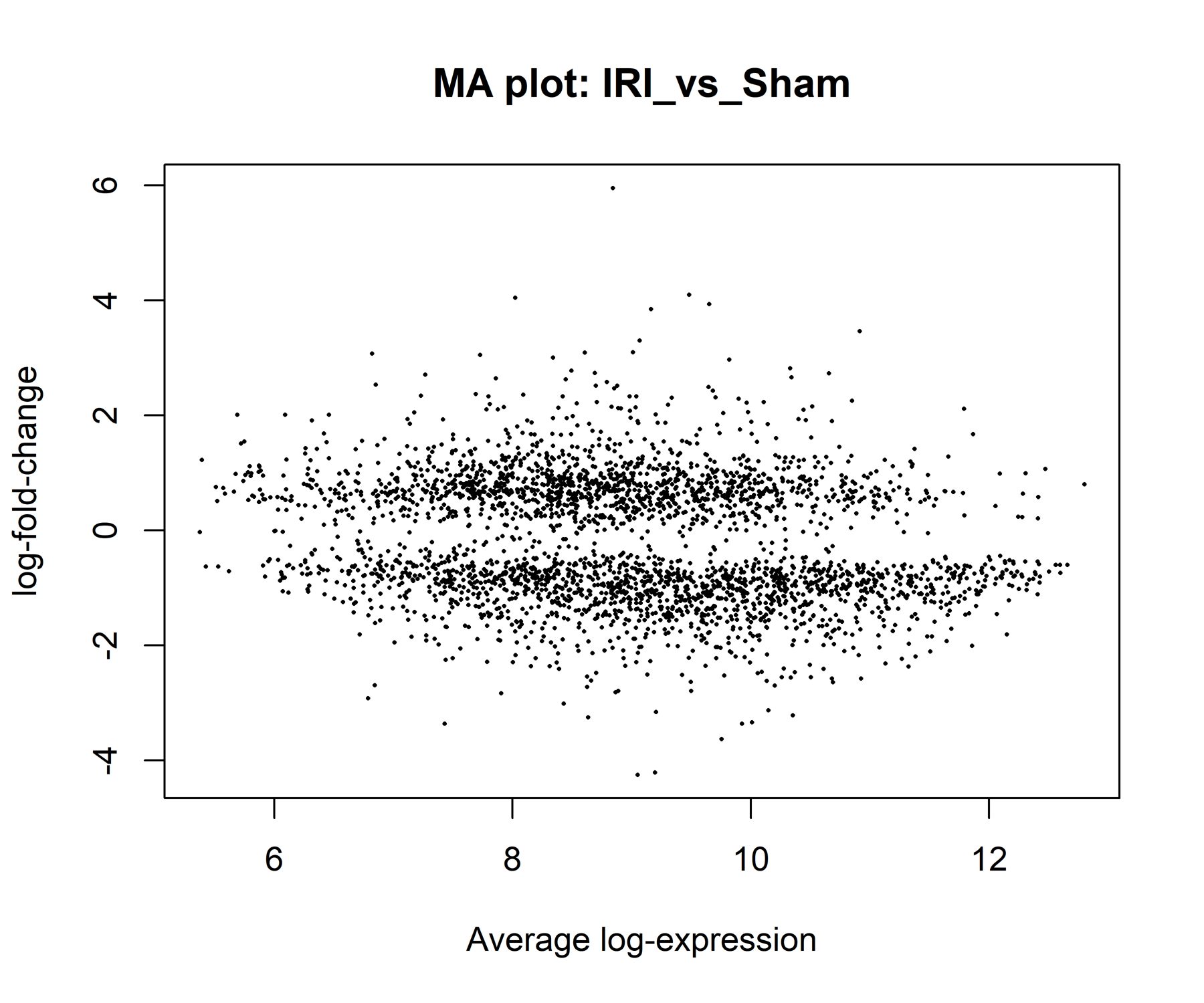


**Figure S2-017. Bulk transcriptome analysis, step5: bulk 05 MA IRI vs Sham**


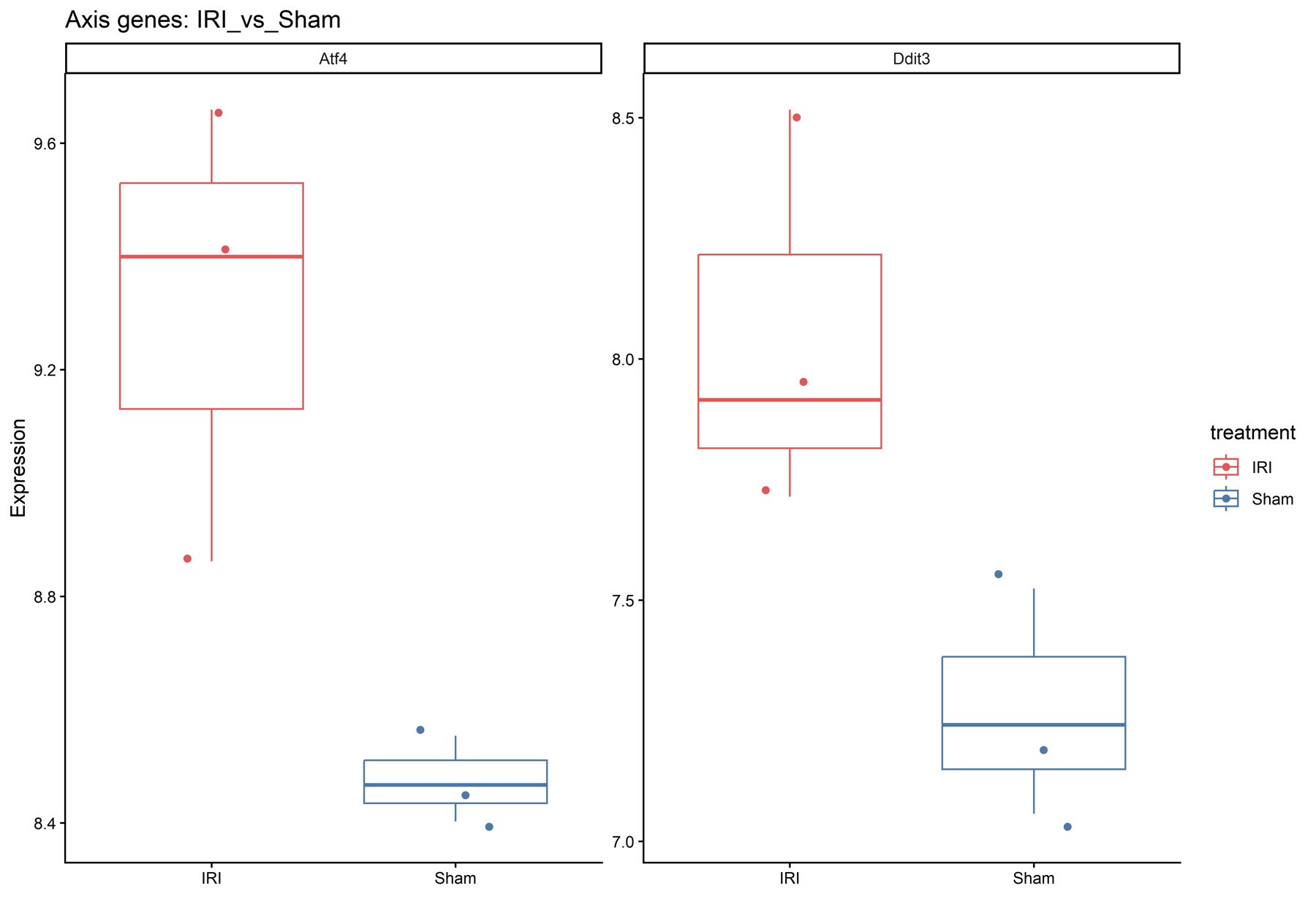


**Figure S2-018. Bulk transcriptome analysis, step5: bulk 05 topgenes boxplot IRI vs Sham**


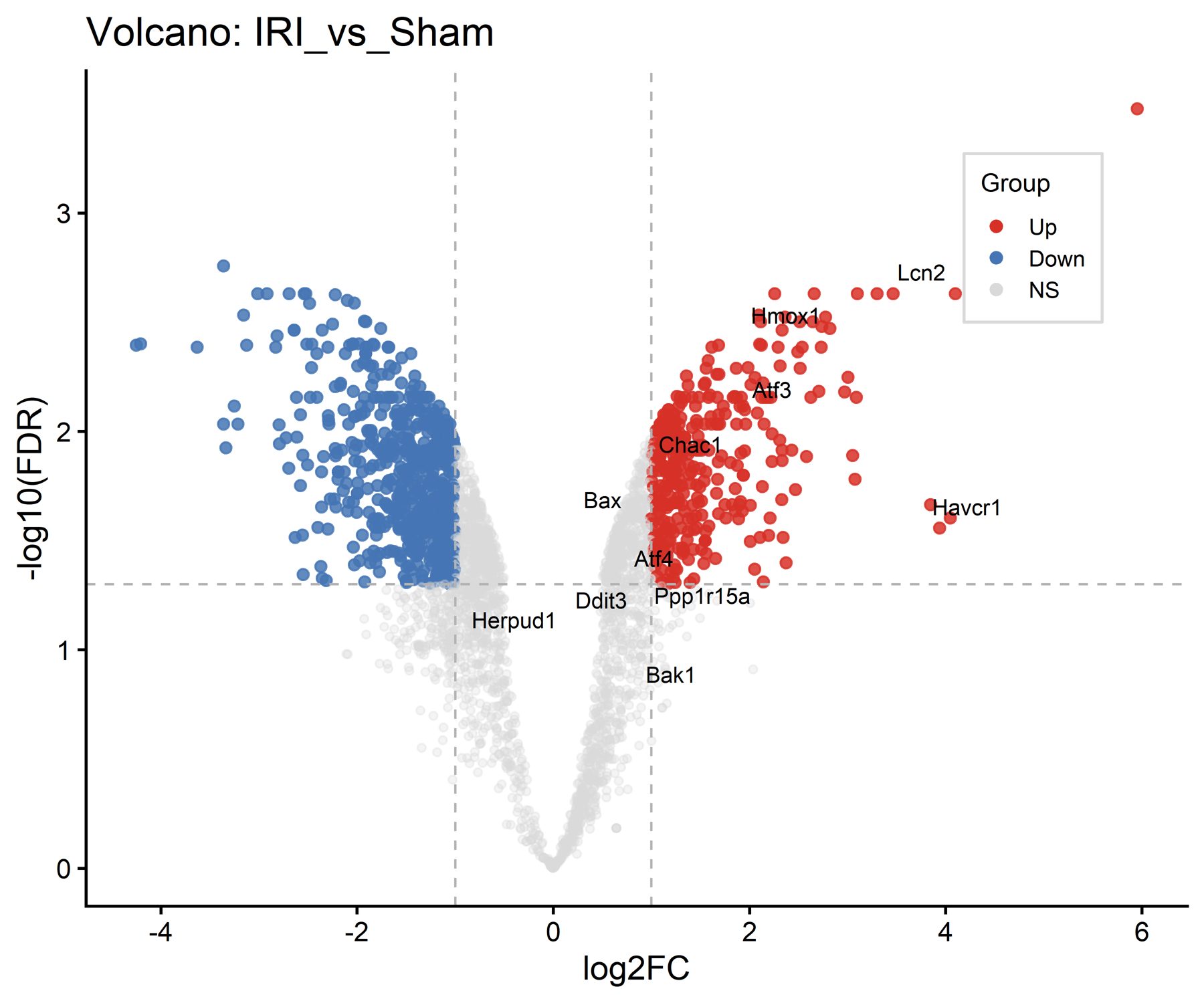


**Figure S2-019. Bulk transcriptome analysis, step5: bulk 05 volcano IRI vs Sham**

# Section: step6


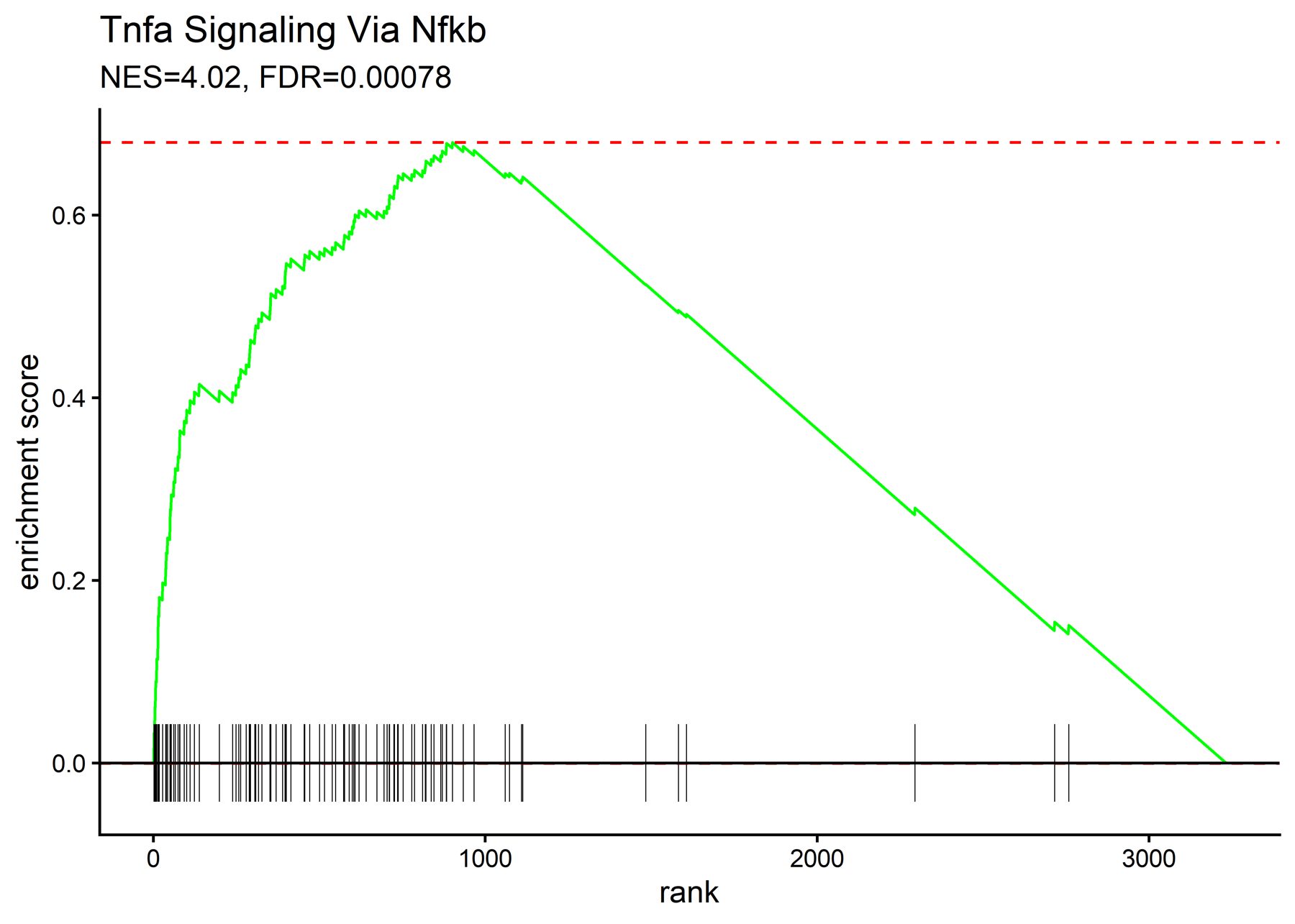


**Figure S2-020. Bulk transcriptome analysis, step6: bulk 06 GSEA curves IRI vs Sham**


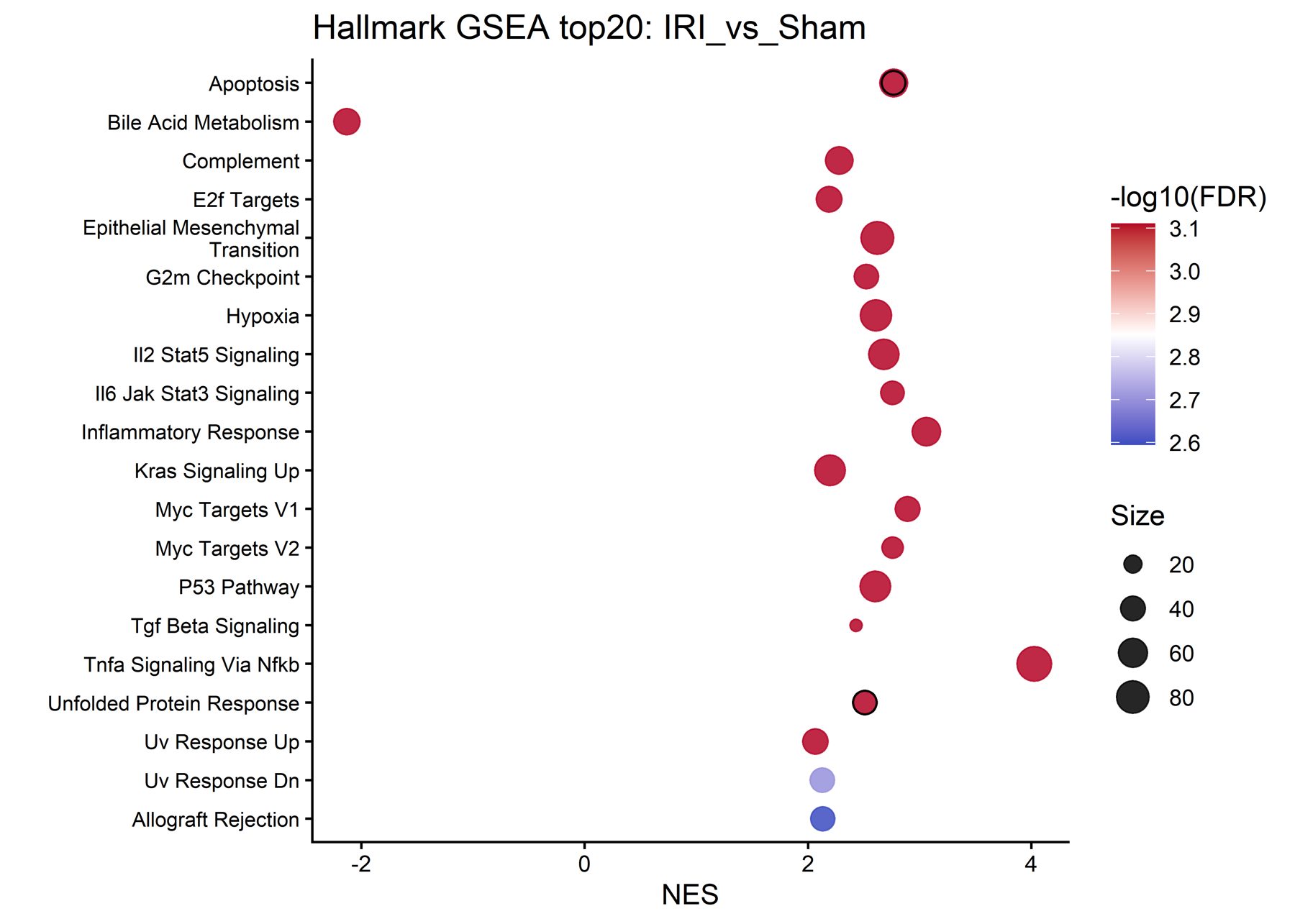


**Figure S2-021. Bulk transcriptome analysis, step6: bulk 06 GSEA Hallmark dotplot IRI vs Sham**

# Section: step7

This section has 1 blank-like image(s), which were omitted.


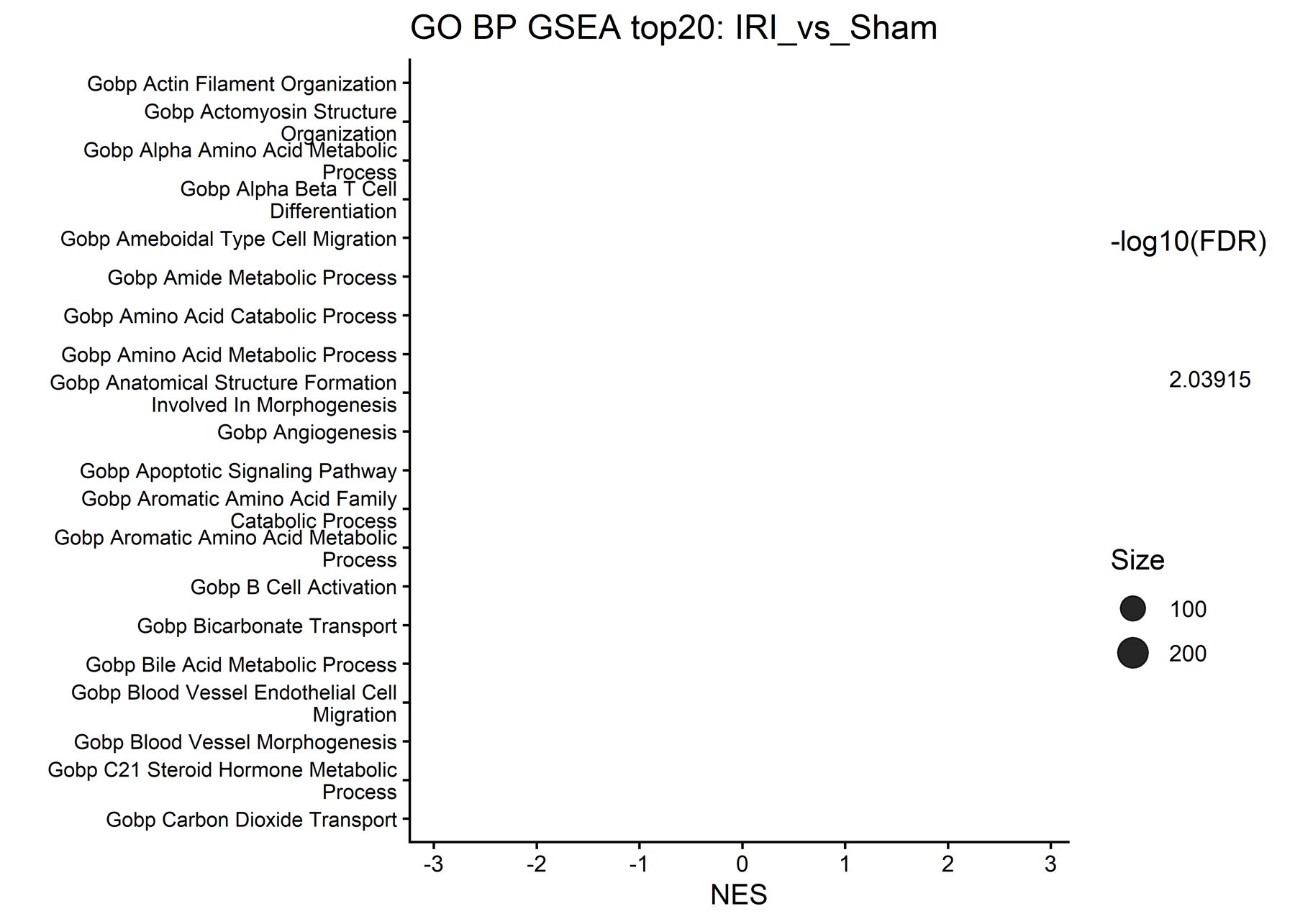


**Figure S2-022. Bulk transcriptome analysis, step7: bulk 07 GSEA GO dotplot IRI vs Sham**

# Section: step8


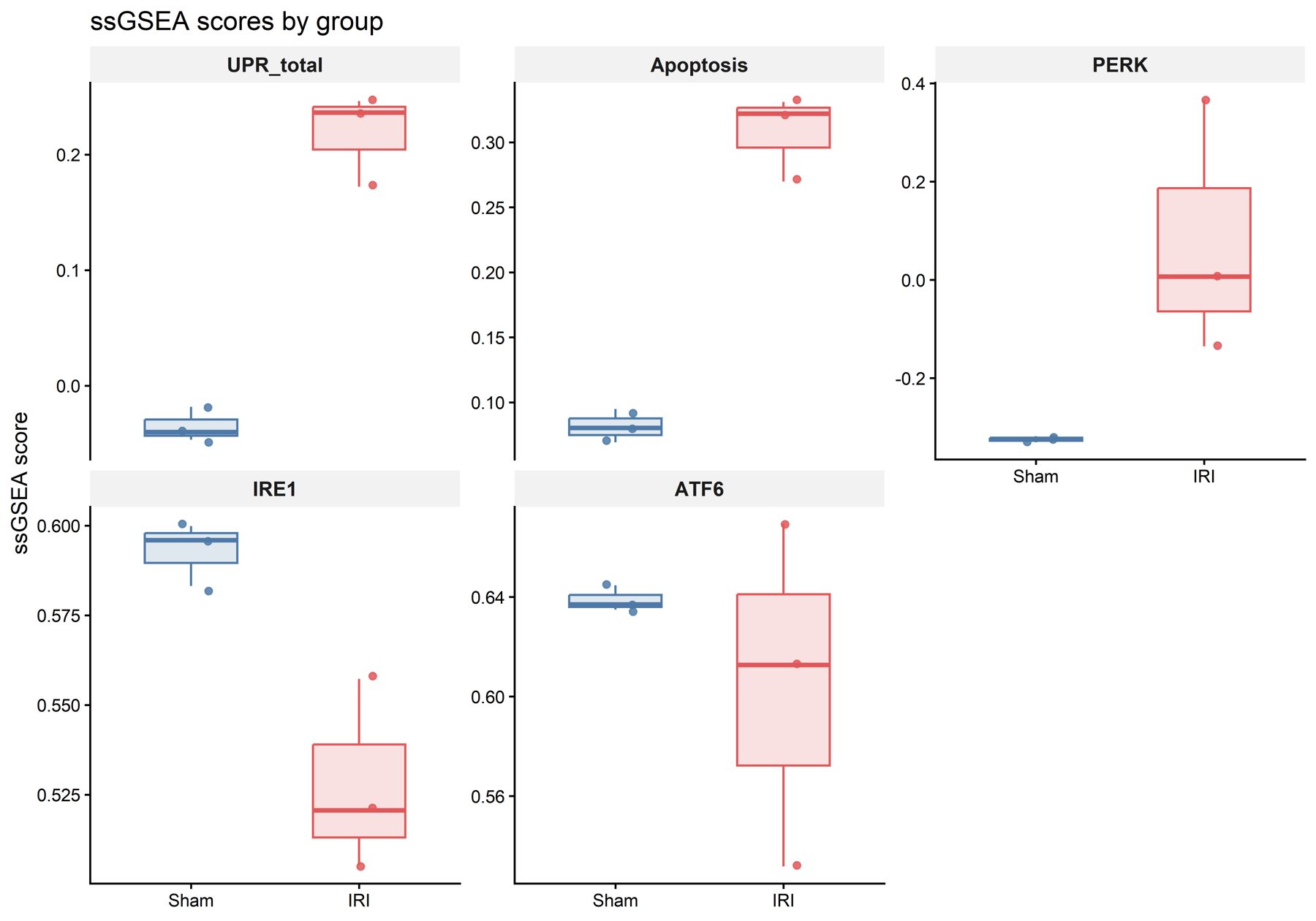


**Figure S2-023. Bulk transcriptome analysis, step8: bulk 08 ssgsea boxplot by group**


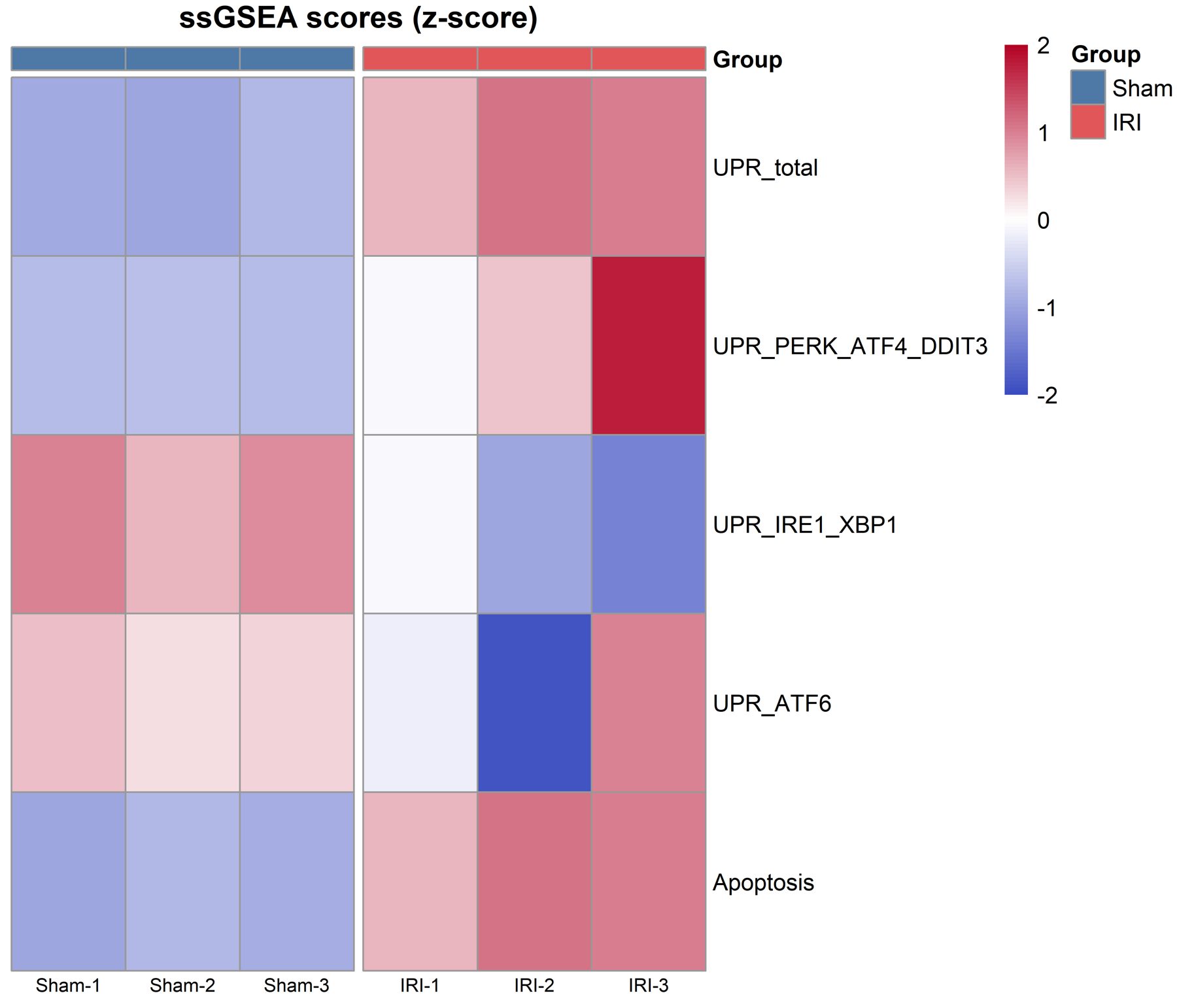


**Figure S2-024. Bulk transcriptome analysis, step8: bulk 08 ssgsea heatmap samples**

# Section: step9


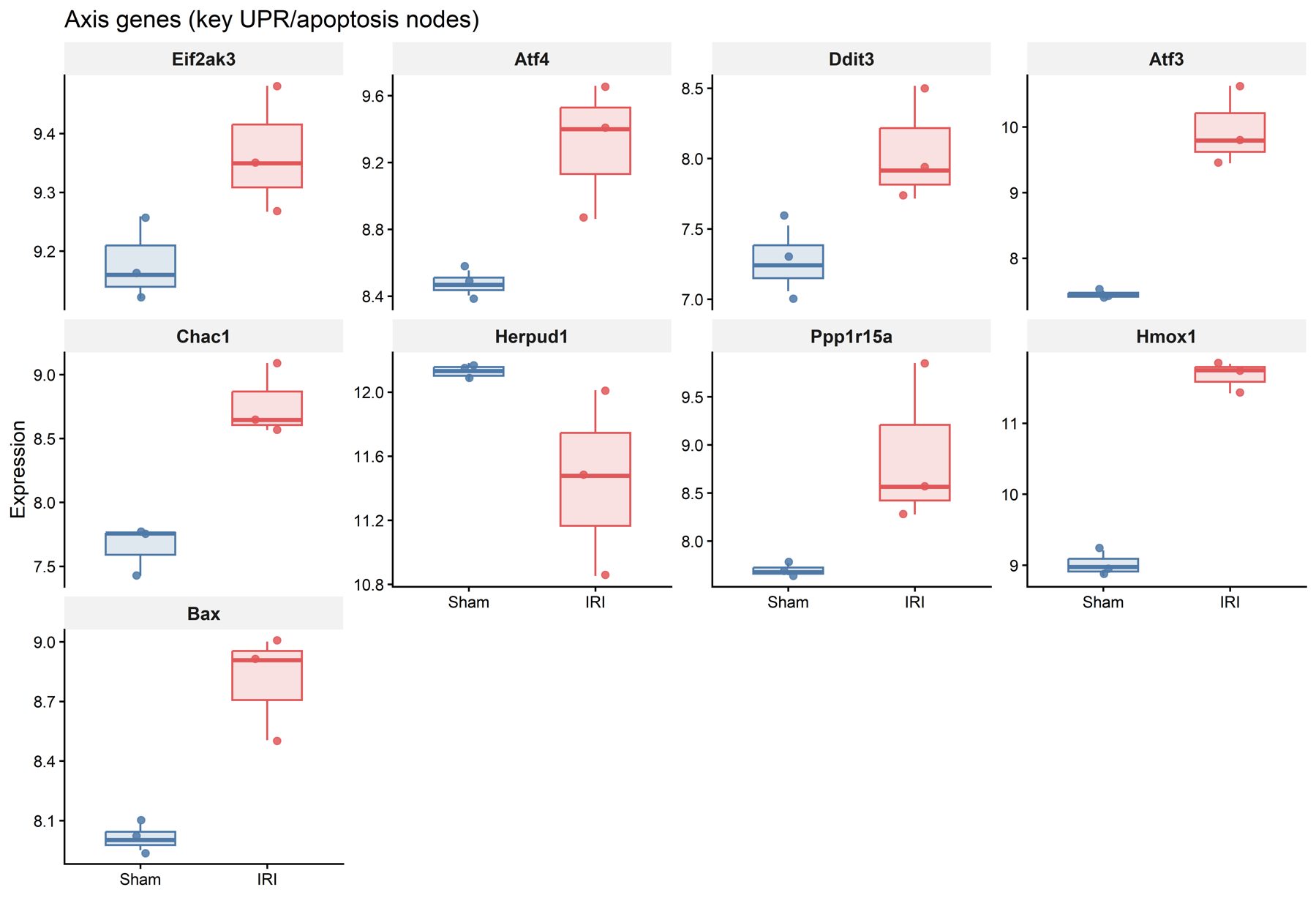


**Figure S2-025. Bulk transcriptome analysis, step9: bulk 09 axis genes boxplot**


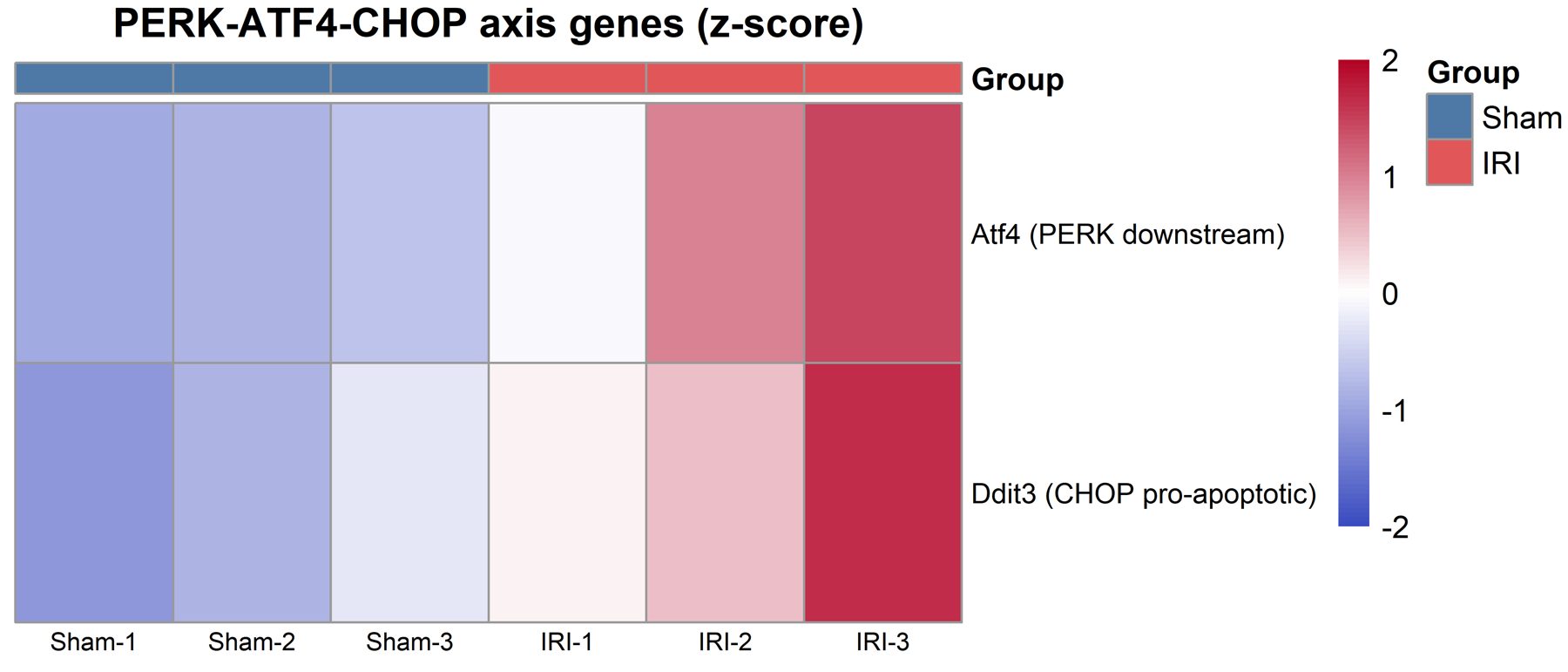


**Figure S2-026. Bulk transcriptome analysis, step9: bulk 09 axis genes heatmap**
