# Supplementary material for: Integrated transcriptomics identifies ER stress–associated apoptosis in post-resuscitation AKI and supports early Dl-3-n-butylphthalide–associated renoprotection in a porcine TCA model
Source: Front Pharmacol. 2026 Jun 4;17:1841271. doi: 10.3389/fphar.2026.1841271 (PMC13275486; doi:10.3389/fphar.2026.1841271)
Supplement: Supplementary file 7 [file DataSheet1.docx]

# Supplementary Methods

## 2. Bioinformatics Analysis (Pathway Retrieval and Cellular Localization)

### 2.1 Data Sources and Software Environment

We established a stepwise analytical framework of "bulk pathway screening -> single-cell cellular localization -> proximal tubule (PT) subset mechanistic convergence". Bulk microarray data were obtained from GEO (GSE52004; mouse kidney tissue; IRI 24 h vs Sham; GPL6246, Affymetrix Mouse Gene 1.0 ST Array). Single-cell data were obtained from GEO (GSE139107; Sham, IRI_4 h, and IRI_12 h).

The analysis environment was R 4.5.1 (Windows x64, ucrt). Major package versions were: Seurat 5.4.0, SeuratObject 5.3.0, harmony 1.2.4, scDblFinder 1.22.0, SingleCellExperiment 1.32.0, UCell 2.12.0, monocle3 1.4.26, edgeR 4.8.2, GSVA 2.2.0, fgsea 1.34.2, msigdbr 25.1.1, clusterProfiler 4.16.0, enrichplot 1.28.4, CellChat 1.6.1, oligo 1.71.7, GEOquery 2.76.0, and limma 3.64.3 (bulk runtime record: bulk_sessionInfo.txt).

### 2.2 Bulk Microarray Preprocessing and Differential Expression

Raw microarray data were processed using oligo::rma for background correction, quantile normalization, and log2 transformation. Probe-level signals were mapped to gene symbols and collapsed into a gene-level expression matrix. Differential expression analysis was performed using limma (lmFit + eBayes) for the primary contrast (IRI vs Sham), with Benjamini-Hochberg correction. Significance thresholds were FDR < 0.05 and |log2FC| > 1.

### 2.3 Bulk Pathway Enrichment and Sample-level Scoring

Hallmark GSEA was conducted using fgsea based on genes ranked by t-statistics (rank metric: t-stat), with parameters nperm = 10000, minSize = 15, and maxSize = 500. Hallmark gene sets were obtained from msigdbr (Mus musculus). Normalized enrichment score (NES) and FDR were used for pathway ranking and visualization.

Sample-level pathway quantification was performed using ssGSEA in GSVA with method = "ssgsea", kcdf = "Gaussian", and mx.diff = TRUE. UPR_total, Apoptosis, and three UPR branches (PERK, IRE1, and ATF6) were calculated.

### 2.4 Gene Set Definition and Traceability

Gene sets were version-controlled in YAML files. On the bulk side, gene sets were stored in bulk_gene_sets.yml. On the single-cell side, gene sets were stored in 08_gene_sets.yml (or equivalently named script-generated outputs). UPR_total and Apoptosis were derived from Hallmark gene sets. The PERK-ATF4-DDIT3, IRE1-XBP1, and ATF6 branch sets, together with extended ER stress/apoptosis sets, were literature-curated and fixed before downstream analyses.

### 2.5 Single-cell QC, Doublet Removal, Integration, and Annotation

Single-cell analysis followed the Seurat standard workflow. QC thresholds (recorded in 01_QC_thresholds_used.txt) were: min_features = 200, max_features = 6000, max_percent_mt = 20 (mitochondrial pattern ^mt-), nCount_RNA <= 99th percentile (recorded nCount_q99 = 3471), and min_nCount = 0 (no additional lower bound).

Doublet detection was performed using scDblFinder (v1.22.0). Integration was performed as SCTransform (regressing percent.mt) -> RunPCA (npcs = 30) -> RunHarmony (group.by.vars = "sample_id") -> FindNeighbors (dims = 1:30) -> FindClusters (resolution = 0.6) -> RunUMAP (dims = 1:30). No additional erythrocyte-marker-based exclusion was applied during preprocessing; cell types were annotated after integration.

### 2.6 PT Subset Re-analysis and State Definition

PT cells were extracted for re-analysis and re-clustered using Harmony integration (group.by.vars = "sample_id") and resolution = 0.6. PT states were defined by marker rules: PT_normal_like (Slc34a1/Lrp2/Slc5a2/Aqp1), PT_injured (Havcr1/Lcn2), and PT_repair (Sox9/Vcam1/Krt8/Krt18/Mki67/Top2a).

Pathway scores were computed using both AddModuleScore (including regression-adjusted versions) and UCell. All between-group statistical tests were performed at sample level to avoid pseudoreplication.

### 2.7 Pseudotime Analysis (Monocle3)

PT trajectory analysis was performed using monocle3. PT cells were converted to a cell_data_set and processed with learn_graph(use_partition = FALSE), order_cells(root_cells = ...), and graph_test(neighbor_graph = "principal_graph"). Pseudotime was used to compare group-level distributions (ridge plots) and to assess relationships with UPR/Apoptosis scores (cell-level smoothing and sample-level correlations). For sample-level pseudotime analyses, minimum cells per sample was set to min_cells_per_sample = 50 (as recorded in Step13 outputs).

### 2.8 PT Pseudobulk Differential Analysis and Pathway Convergence

PT counts were aggregated by sample to generate pseudobulk matrices. Inclusion threshold was min_cells_per_sample = 20 (see 09_pseudobulk_params.txt and 09_pseudobulk_design_matrix.txt). Differential analysis was performed using edgeR quasi-likelihood workflow: DGEList -> filterByExpr -> calcNormFactors (TMM, library-size normalization) -> estimateDisp -> glmQLFit -> glmQLFTest. The design matrix was model.matrix(~ group), and logFC direction was reported as group1 - group0. Hallmark GSEA was then applied to ranked differential results to form a closed validation loop linking pathways, differential signals, and axis genes.

### 2.9 Statistical Analysis

Pathway scores, state proportions, and compositional metrics were analyzed at sample level. Kruskal-Wallis tests were used for multi-group comparisons, and pairwise Wilcoxon rank-sum tests were used for post hoc analyses, with Benjamini-Hochberg correction. Where appropriate, effect sizes (e.g., Cliff's delta) and interval estimates were reported.
